# Identification of Binding Targets of a Pyrrole-Imidazole Polyamide KR12 in the LS180 Colorectal Cancer Genome

**DOI:** 10.1371/journal.pone.0165581

**Published:** 2016-10-31

**Authors:** Jason Lin, Kiriko Hiraoka, Takayoshi Watanabe, Tony Kuo, Yoshinao Shinozaki, Atsushi Takatori, Nobuko Koshikawa, Anandhakumar Chandran, Joe Otsuki, Hiroshi Sugiyama, Paul Horton, Hiroki Nagase

**Affiliations:** 1 Laboratory of Cancer Genetics, Chiba Cancer Center Research Institute, Chuo-ku, Chiba, Japan; 2 Artificial Intelligence Research Center, National Institute of Advanced Industrial Science and Technology, Koto-ku, Tokyo, Japan; 3 Department of Materials and Applied Chemistry, Nihon University, Chiyoda-ku, Tokyo, Japan; 4 Department of Chemistry, Kyoto University, Sakyo-ku, Kyoto, Japan; Lawrence Berkeley National Laboratory, University of California, Berkeley, UNITED STATES

## Abstract

Pyrrole-imidazole polyamides are versatile DNA minor groove binders and attractive therapeutic options against oncological targets, especially upon functionalization with an alkylating agent such as *seco*-CBI. These molecules also provide an alternative for oncogenes deemed “undruggable” at the protein level, where the absence of solvent-accessible pockets or structural crevices prevent the formation of protein-inhibitor ligands; nevertheless, the genome-wide effect of pyrrole-imidazole polyamide binding remain largely unclear to-date. Here we propose a next-generation sequencing-based workflow combined with whole genome expression arrays to address such issue using a candidate anti-cancer alkylating agent, KR12, against codon 12 mutant *KRAS*. Biotinylating KR12 enables the means to identify its genome-wide effects in living cells and possible biological implications via a coupled workflow of enrichment-based sequencing and expression microarrays. The subsequent computational pathway and expression analyses allow the identification of its genomic binding sites, as well as a route to explore a polyamide’s possible genome-wide effects. Among the 3,343 KR12 binding sites identified in the human LS180 colorectal cancer genome, the reduction of KR12-bound gene expressions was also observed. Additionally, the coupled microarray-sequencing analysis also revealed some insights about the effect of local chromatin structure on pyrrole-imidazole polyamide, which had not been fully understood to-date. A comparative analysis with KR12 in a different human colorectal cancer genome SW480 also showed agreeable agreements of KR12 binding affecting gene expressions. Combination of these analyses thus suggested the possibility of applying this approach to other pyrrole-imidazole polyamides to reveal further biological details about the effect of polyamide binding in a genome.

## Introduction

While protein-level inhibitors aimed to inhibit enzyme activity or disrupt binding enjoy a large degree of commercial success, pyrrole-imidazole polyamides (“PIPs”) and their accurate DNA base pair recognition [1–5] provide a promising option against a large class of so-called “undruggable” targets such as *KRAS* [6–8] which lack solvent-accessible surfaces or pockets for ligand binding. From paired heterocyclic amide building blocks of pyrrole and imidazole, PIPs can distinguish G/C and A/T (T/A) pairs via DNA minor groove binding with Im/Py and Py/Py pairs, respectively, down to the precision of a single hydrogen bond between the bases. Chemical functionalization provides versatility in regulating biological events [9–15] such as cell reprogramming, NF-*κ*B-dependent gene transcription, retinal development, and the inhibition of specific biological targets [16, 17]. Nevertheless, as PIPs principally bind typically well less than 20 bp, the impact of binding at genomic locations other than the intended design site remains unclear to-date. Without exploring other possible binding sites, it may be too premature to conclude that unanticipated binding to non-KRAS loci has no effect, since off-target effects could also contribute to cancer cell death and mask the true efficacy of a therapeutic candidate. In spite of recent developments in next-generation sequencing, PIP binding studies, especially those involving alkylating functional groups, remain few and far between at the genome level. Hopefully with the procedure discussed in this manuscript, we can introduce a useful addition in the design pipeline to understand whether off-target binding may have a large effect on the tumor before more costly studies, e.g. animal models. Sequencing studies involving bioactive DNA-targeting polyamides conjugated with a psoralen moiety for photo-crosslinking and biotin as affinity tag [18] so far have been largely restricted to chromatinized regions in the living cell genome until the latest report with a 6-bp alkylating PIP [19], albeit in an *ex vivo* setting. Following our previous study of a PIP with 9-bp recognition demonstrating toxicity against *KRAS* G12 mutant cell lines [20], we seek to evaluate, via a new sequencing procedure with the biotinylated KR12 to affinity capture nucleotides bearing binding sites for the polyamide *in vivo*, and computational workflow to determine whether other genomic binding sites in living cells may present an issue in the effectiveness of alkylating PIPs as therapeutic candidates. Corroborating findings here with our previous publication in which KR12 demonstrated distinguishable toxicities in *KRAS* G12D/G12V over wild-type cells suggested specific targeting of the mutant driver gene by the polyamide. Results here also revealed, for the first time at 9-bp precision, insights into the manner in which PIPs access the human genome in cells.

## Materials and Methods

### General Materials and Computational Tools

Chemicals and molecular biology grade reagents were purchased from the following manufacturers: PyBOP and Fmoc-*β*-alanine Wang resin, Novabiochem; HCTU, Peptide Institute Inc. (Osaka, Japan); HPLC grade acetonitrile and biotin, Aldrich Chemicals; TrisHCl, NP-40, SDS, EDTA, sodium bicarbonate, 25:24:1 phenol/chloroform/isoamyl alcohol, Nacalai Tesque; Triton-X 100, Complete Protease Inhibitor, Roche; HEPES, Gibco; pyrrole, imidazole, DIEA, sodium chloride, PIPES, sodium acetate, and solvents (unless otherwise specified), Wako Chemicals.

Computational utilities such as bedtools 2.25.0, samtools 1.3, bcftools 1.3, diffReps 1.55.4 and MACS 1.4.2 were used in conjunction with custom R 3.2 and Perl 5.16 scripts [21–29]. LS180 H3K27Ac ChIP-seq results were obtained from NCBI (GSM1890754). DNase I hypersensitivity (digital DNaseI hypersensitivity clusters, DHS) and chromatin state segmentation (Broad ChromHMM) data [30, 31], in hg19 coordinates, were obtained from ENCODE [32]. RefSeq annotations and hg19 reference sequences (chr1-22 and X, Y, M) were sourced from RefFlat information from University of California, Santa Cruz (UCSC) [33, 34]. The sequence file for the LS180 genome was reassembled from CCLE sequencing results (G41705) from CGHub [35] and reassembled by Picard 1.137 and GATK 3.4.46 to hg19 coordinates, with indels ignored during multi-allelic variant calling by bcftools.

### Synthesis of KR12

The backbone chain (ImPy-*β*-ImImPyIm-*γ*-PyIm-*β*-PyPy) for the (+) strand motif 5’-TGWWGGCGW-3’ was synthesized in a stepwise Fmoc solid-phase synthesis reaction on a PSSM-8 peptide synthesizer (Shimadzu) with a custom operating system on 10-μmol scale with Fmoc-*β*-alanine Wang resin. Cleavage from solid support was achieved with 0.5N LiOH/NMP at 55°C for 1 h. Purification of the cleavage product was performed using high-performance liquid chromatography on a LC-20 (Shimadzu), using a 10 mm × 150 mm Gemini-NX3u 5-ODS-H reverse-phase column (Phenomenex) using 0.1% acetic acid in Milli-Q water and a linear gradient of 0–66.7% acetonitrile over 20 m at a flow rate of 10 ml m^-1^ with detection at 310 nm. Collected fractions were analyzed by LC-MS2020 (Shimadzu). After purification, N-terminal biotinylation and C-terminal conjugation of *seco*-CBI to produce KR12 was achieved by incubating the cleavage product in the presence of biotin, PyBOP and DIEA for 2 h to allow reaction; after the conversion of the C-terminal carboxylic acid to the activated 1-hydroxybenzotriazole ester, the reaction was mixed with 5.1 μmol NH_2_-*seco*-CBI in 100 μL NMP [36] and stirred overnight to allow coupling. After solvent evaporation, the reaction product was characterized and purified by liquid chromatography and a linear gradient of 0.1% acetic acid in 30%– 75% acetonitrile and water over 0.5 h with detection at 310 nm. The final product (t_R_ = 16.87 m) after purification was lyophilized and stored. ESI-TOF-MS C_100_H_110_ClN_34_O_18_S m/z calcd [M+H]^2+^ 1071.95; found 1072.00, [M+3H]^3+^ 714.94; found 715.25.

### Cell Cultures and Viability Assay

Colorectal adenocarcinoma LS180 and SW480 cells were cultured and maintained at 37°C, 5% CO_2_ under humid conditions in Eagle’s Minimum Essential Medium (MEM, Gibco) and Dulbecco’s Modified Eagle’s Medium (DMEM, Gibco), respectively. Media were additionally supplemented with 10% fetal bovine serum (Gibco) and 1% penicillin/streptomycin (Gibco). For sequencing experiments, cultures were prepared in replicates at 6 × 10^5^ cells/dish prior to KR12 administration. For WST viability assays, cells were seeded at a final density of 10^3^ cells per sample in a 96-well plate for overnight attachment prior to treatment with 0.1% DMSO, 300 and 1000 nM KR12 as well as non-biotinylated variant (KR12 N/B) in 0.1% DMSO for 48 h to assess compound toxicity. After compound administration, WST-8 reagent (Dojindo) was added to each sample for incubation at 37°C for 2 h prior to absorbance measurement at 450 nm on a MTP-310 photospectrometer (Corona).

### Ion Torrent Sequencing

LS180 and SW480 cultures were treated with 500 nM KR12 in 0.05% DMSO for 6 h before washing, detachment in ice-cold phosphate-buffered saline and collection by centrifugation. Pellets were resuspended in a lysis buffer of 5 mM PIPES, 0.5% NP40 and 1% Complete Protease Inhibitor, incubated on ice for 10 m to extract nuclei by ultracentrifugation. After resuspension in nuclease lysis buffer of 50 mM Tris-HCl, 10 mM EDTA, 1% SDS and 1% Complete Protease Inhibitor at pH 8.1, samples were again incubated on ice for 10 m prior to overnight storage at -80°C. Since KR12 formed alkylation adducts with DNA, crosslinking was not performed during the DNA enrichment process. Genomic DNA was fragmented by sonication on a Covaris M220 for two 10 m runs of 200 cycles (peak power of 75, duty level of 26) before precipitation in a solution of 300 mM NaOAc in 70% ethanol (JIS Special Grade, Wako) at 30°C and isolation of genomic DNA by Micro-Vac evaporation (Tomy Digital Biology). Samples were resuspended in nuclease-free water (Millipore) for 15 m before collecting the supernatant for enrichment with Dynabeads MyOne streptavidin C1 beads (Invitrogen) over 15 m. Beads were washed twice in a buffer of 20 mM Tris-HCl, 0.1% SDS, 1.1% Triton X-100, 2 mM EDTA and 500 mM NaCl at pH 8.0, and five additional times in detergent-free buffer (5 mM Tris-HCl, 0.5 mM EDTA, 1 M NaCl at pH 7.5) prior to elution with 2% SDS, 0.1 mM NaHCO_3_ and 3 mM biotin at 65°C for 4 h under agitation.

Enriched DNA was purified by RNase A (5 μg, Invitrogen) for 10 m at 37°C, followed by proteinase K (50 μg, Merck Millipore) treatment at 55°C for 40 m. Samples were mixed with glycogen (Ambion), resuspended prior to phenol extraction. The supernatant was retained, from the phenol layer, after centrifugation in 1:1 TE:NaCl and stored as aliquots in absolute ethanol at -20°C prior to precipitation by centrifugation (17,000 × g, 4°C for 0.5 h). Samples were evaporated in open atmosphere, reconstituted in 20 μL TE and quantified by Qubit 2.0 Fluorometer (ThermoFisher). Samples were end-repaired by 30 m incubation in the supplied end-repair buffer and enzyme (2 μL) in Ion XpressTM Plus Fragment Library Preparation Kit; the reaction mixtures were suspended with AMPure XP Reagent Beads (Agencourt), washed in ethanol (70%) and eluted in Low TE buffer before adapter ligation for 30 m and again purified with AMPure beads. Nick repair and amplification with Platinum PCR SuperMix High Fidelity mix (ThermoFisher) proceeded at 72°C for nick repair (20 m), 95°C for denaturation (5 m), 18 cycles of 97°C denaturation, annealing at 60°C and 70°C extension (15 s, 15 s and 1 m, respectively). Library preparation proceeded with 1.68 ng of enriched DNA with Ion Plus Fragment Library Kit (ThermoFisher) and AMPure XP Reagent Beads (Agencourt) per manufacturer’s recommendations. Library sizes were validated on a Bioanalyzer 2100 (Agilent) and quantified using the Ion Library Quantitation Kit (ThermoFisher); after emulsion PCR of the prepared sample on Ion OneTouch 2 (ThermoFisher), a final quantity of 0.8 fmol DNA was used for sequencing on an Ion Proton sequencer (ThermoFisher). Data acquisition and alignment to hg19 genome were performed using Torrent Suite 5.0.4.

### Differential Identification and Statistical Validation of KR12 Sites from Sequencing Results

Ambiguous reads, i.e. mapping quality = 0, were removed from the BAM files before summarizing base calls of aligned reads (mpileup) with samtools, with BAQs recalculated on the fly and genotype likelihood output in uncompressed BCF format. Sequencing data (treatment and control) were then converted to BED format before differential calling by diffReps with hg19 as the genome size, method of chi-square, fragment size of 0 and no annotations; window sizes varied from run to run within the range of 400–1200 bp. Sequences were regenerated over such diffReps-assigned regions from the source FASTA file with indels ignored (see General Computational Section), by bedtools, ignoring strand information. Matches to the KR12 motif (“TGWWGGCGW” on the (+) strand), in both forward and reverse complementary orientations, were identified by searching against the reconstructed sequence files, followed by a back-transformation to hg19 coordinates. Positions were again inspected against pileup results for indels; sequences from such sites were reconstructed by variant calling with bcftools over given regions by multi-allelic calling, with compressed output containing only variant sites. Indel information was used to reconstruct a small region for motif searches and translated to hg19 coordinates. Transcript positions and gene symbols from hg19 RefFlat (last accessed Feb 2015) were used to generate an annotation including transcript locations and promoter regions, defined as 1000 bp region upstream of transcription start site (TSS), based on RefFlat strand information. KR12 candidates were subsequently annotated to produce a list of all positions of candidate KR12 sites from sequencing results annotated by their respective gene or promoter symbols.

For statistical validation, a list of nonbinding regions was generated by subtracting candidate KR12 sites from the list of hypothetical sites via bedtools (removing entire feature if any overlap was present). Randomizations were performed to generate null foreground and background positions in R (sampling without replacement, with an equal number of null and candidate sites). Per-base coverage was calculated over candidate regions as well as paired null foreground and background regions from sequencing data, before unity normalization of each region. A differential distribution was determined by subtracting per-base coverage between the input and pulldown groups; for the null differential distributions, shuffling was performed to pair randomly the null foreground with a background to increase randomness. For each of the candidate differential distribution, two-sample Kolmogorov-Smirnov test was performed for each of the null differential distribution under the hypothesis that there was no difference between the distributions; resultant *p*-values were adjusted for multiple comparisons by Benjamini-Hochberg corrections. Statistical significance was assessed based on the adjusted *p*-value at the 99.9^th^ percentile against a pre-defined *α*-level of 0.05; sites with a *p*-value of 0.05 < *p* < 0.055 were considered marginally significant and likewise annotated. Fold enrichment was calculated based on the log_2_ ratio of the maximum coverage within a given window in the pulldown and input samples. Results from multiple runs of varying window sizes for diffReps were compiled to produce the final output of 3,343 KR12 binding sites.

### Reverse-transcription Polymerase Chain Reaction

Cultures of 9.6 × 10^4^ LS180 cells/well were treated with 500 nM KR12 for 6 h before RNA extraction with RNAeasy plus mini kit (Qiagen) and reverse transcription of 500 ng RNA to cDNA by the SuperScript VILO cDNA Synthesis System (Invitrogen) for experiment as previously described [20]. Polymerase chain reactions were performed with temperature cycles as follows: 95°C, 2 m; (95°C, 30 s; 58°C, 30 s; 72°C, 30 s) over a number of optimized PCR cycles (*RPS18*: 23 cycles; *KRAS*, *PIK3CA*, *GUSB*: 28 cycles); 72°C, 5 m, ending with holding at 4°C. Primer sets used were as follows: *KRAS*, 5’-GGAGAGAGGCCTGCTGAA-3’ (sense) and 5’-TGACCTGCTGTGT CGAGAAT-3’ (antisense); *RPS18*, 5’-GAGGATGAGGTGGAACGTGT-3’ (sense) and 5’-TCTTCAGTCGCTCCAGGTCT-3’ (antisense); *PIK3CA*, 5’-AGTCGCCACCTACCACAGAG-3’ (sense) and 5’-GCTGACCCTCATGGCTGT-3’ (antisense); *GUSB*, 5’-GGTGGTTCATTGCTGCTGAC-3’ (sense) and 5’-TAGAACAGAGAGCGCCATTG-3’ (antisense).

### Expression Microarrays

LS180 and SW480 cultures at 9.6 × 10^4^ cells per sample were plated in a 6-well microtiter plate for overnight attachment prior to treatment with 500 nM KR12 or 0.05% DMSO for 6 h. After RNA extraction with RNeasy Plus Mini Kit (Qiagen), samples at 100 ng were labeled with RNA Spike-In Kit and analyzed on SurePrint G3 Human GE 8x60K V2 microarrays per Agilent Technologies’ recommendations. Arrays with sample replicates (2 × 2 for LS180, 3 for SW480) for each condition (DMSO, control; treatment, 500 nM KR12) were scanned on an Agilent SureScan microarray scanner, with differential expressions calculated by the LIMMA package [37] (background correction by the “normexp” method with an offset of 16, and scale-normalized for replicates between arrays). LIMMA calculated a linear model fit for each gene and utilized the method of empirical Bayes for statistical evaluations and differential expressions, with fold changes calculated from the difference between DMSO and KR12 treatments. Spots with matching RefSeq mRNA and ncRNA identifiers were filtered and retained for subsequent analyses. Statistical significance of gene expressions, unless otherwise specified, was assessed by two-sample *t*-test under the null hypothesis that there was no difference between their sample means against a predefined *α*-level of 0.05. For the purpose of comparison, genes with promoter region (defined as 1000 bp upstream of transcription start site) binding were also annotated with corresponding mRNA expressions.

### Nucleosome Occupancy Profiling

DHS, H3K27Ac and nucleosome structure information were obtained as described in the General Computational Tools Section. ChromHMM tracks from eight cell lines (GM12878, H1 hESC, HepG2, HMEC, HSMM, K562, Nhek and Nhlf) were combined to identify common overlaps, and heterochromatin regions with ≥ 5/8 overlaps were retained to create a consensus heterochromatin track for comparison. LS180 H3K27Ac data were reprocessed in MACS 1.4.2 to generate a region file of signature peaks with default parameters and an effective genome size of 2.7 × 10^9^. H3K27Ac and KR12 binding sites were annotated to the closest transcript features to facilitate the determination of two features occupying the same gene or otherwise, and for each KR12 binding site assigned to a gene (or a promoter region), distance to the closest H3K27Ac feature was determined similarly.

### Gene Set Enrichment and Network Analysis

Gene set enrichment analysis was performed with PANTHER 10.0 (May 15, 2015 release) statistical overrepresentation test, using the list generated from sequencing results as well as genes belonging to the Ras pathway (hsa04014, KEGG [38, 39]) under default settings (predefined *α*-level of 0.05, Bonferroni corrections against a reference list of Homo sapiens genes). A list of downregulated KR12-bound genes was selected by cross-referencing microarray expression results for negative log_2_FC values for interaction analysis by STRING 10.0 with the following parameters: highest confidence of 0.900, no additional nodes, custom limit of 0 interactors and all active prediction methods [40]. The resultant network was reconstructed in Cytoscape [41] using STRING-computed combined scores, and sub-networks with nodes less than or equal to four hidden for illustrative purposes.

### KEGG Pathway Significance

The KEGGREST package [42] in R was used to acquire pathway-specific information from the KEGG database. All 301 human pathways and corresponding gene lists were extracted from KEGG. The gene lists were then compared against KR12 binding sites from LS180 sequencing data to test for statistical significance in three different areas: 1) whether the mean gene expression in a given pathway compared to other genes was different; 2) whether mean expressions of KR12-bound genes in that pathway was different from non-KR12-binding genes, and 3) whether mean expressions of KR12-bound genes in the pathway were different from other KR12-bound genes. Pathways with less than 2 KR12-binding genes are excluded from tests of significance (81). For pathways satisfying all three criteria (9/220), they were considered candidates for off-target pathways of KR12. Common (duplicate) KR12-bound genes from candidate pathways were checked against all KEGG human pathways involving *KRAS* for possible involvement to assess off-target effects of KR12.

### Prediction of hg19 binding sites of scrambled KR12 motifs

The last eight bases of the KR12 motif (TGWWGGCGW on the (+) strand) were randomly scrambled for 100 permutations to check for genomic binding sites in hg19, with coding region annotations extracted from the Table Browser from UCSC. To simulate 3’ adenine alkylation by the conjugated *seco*-CBI moiety [43] for the scrambled 9-bp polyamides, the 5’ terminal thymine was fixed for all 100 permutations. Counts for each motif were divided by the corresponding KR12 value by the same metric.

### Sequence similarity between KR12-bound non-coding RNA and *KRAS*

Annotations for lncRNA (colon lincRNA RNA-Seq Reads [44]) and miRNA (snoRNA and miRNA [45]) were extracted from the Table Browser from UCSC and intersected with the list of KR12 binding sites. Regions with at least 1 base overlap were retained and their sequences, along with flanking up- and down-stream 1000 bp, were extracted from the LS180 genomic sequence to generate a fasta file containing those 2009 bp regions for 9-mer composition analysis by jellyfish [46] with an initial hash size of 3G. The *KRAS* transcript (chr12: 25357722–25403865) was also extracted for comparison. Tantan [47] was used to perform masking of simple repeats with the letter N.

### Data Availability

Sequence read datasets for the KR12-enriched ("pulldown") and unenriched ("input") LS180 and SW480 genomes are available in the NCBI Sequence Read Archive (SRA) under BioProject PRJNA342228; expression microarray datasets are available at the NCBI Gene Expression Omnibus (NCBI GEO) database (accession number GSE86599).

## Results and Discussion

### Synthesis of KR12 and Cell Toxicity

**Fig 1:**
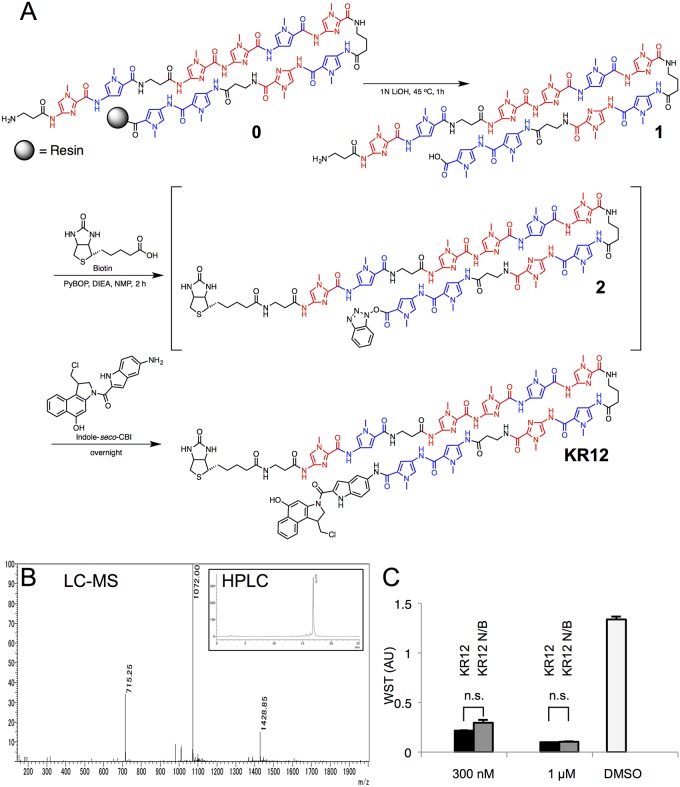
Next-generation sequencing with biotinylated KR12. (A) Synthetic scheme of KR12 from the non-biotinylated precursor KR12 N/B, with pyrroles and imidazoles colored in blue and red, respectively. (B) HPLC retention time diagram (upper right) and mass spectrum (LC-MS) of KR12. (C) WST assay of LS180 cells at 300 and 1000 nM dosage of KR12 (black) compared to the non-biotinylated precursor (KR12 N/B, gray) and DMSO only (white); error bars indicate ±1 SEM; n.s., no significance by two-sample Welch’s *t*-test.

We functionalized the original PIP (**KR12 N/B**) with biotin to create KR12 (Fig 1A) from a modified Fmoc solid-phase peptide synthesis procedure [20]. The one-pot preparation of KR12 via the use of excess PyBOP to allow simultaneous activation of the polyamide backbone C-terminus and biotin yielded the biotinylated intermediate **2**, and the subsequent *seco*-CBI coupling and liquid chromatography successfully afforded the final product (Fig 1B). Since KR12 exhibited similar cell toxicity as the original PIP in LS180 cell lines (Fig 1C), we proceeded with ligand affinity-captured DNA from LS180 6 h after KR12 (500 nM) administration for sequencing.

### Design of Sequencing Pipeline

We then devised a method to enrich KR12-bound nucleotides to sequence for possible KR12 binding sites in the genome of LS180, a human colorectal cancer cell lines with KRAS G12V mutation. Upon DNA extraction and fragmentation by sonication, KR12-alkylated nucleotides underwent streptavidin enrichment and Ion Torrent sequencing to yield ~ 12 Gb per run of sequencing data. Initial inspection showed a mixture of broad and narrow peaks that deviated from typical TFBS ChIP-seq or histone modification, e.g. H3K36me3, experiments, and were also uncharacteristic of DNase-seq [25, 48–50]. Since PIP-DNA interactions would have a smaller binding surface than their protein-DNA counterparts, it was unclear whether popular methods such as MACS or ZINBA would perform optimally with this dataset.

Additionally, our decision to omit formaldehyde crosslinking also attributed to the uncharacteristic peak distributions observed in the sequencing results. As the purpose of crosslinking was to preserve the interaction of DNA with protein targets, e.g. transcription factors, chromatin, etc., via the formation of a methylene bridge between a nucleoside and a nucleophilic nitrogen or sulfur of an amino acid, this additional step was unnecessary with alkylating PIPs due to the presence of an alkylating agent capable of forming a covalent linkage with the template 3’ terminal adenine upon binding. Since concerns about the effectiveness of crosslinking to preserve all protein-DNA ligands in certain cases had also been raised, e.g. [51], it was unclear whether the added step will provide additional fidelity of securing polyamide-DNA interactions. The use of formaldehyde would most likely crosslink DNA to chromatin and other transcription factors, complicating analysis with alkylating polyamides. As previous methods, e.g., COSMIC [18], did not describe whether it was possible to isolate polyamide binding sites outside chromatinized regions, we decided to omit chemical crosslinking in this current procedure.

**Fig 2:**
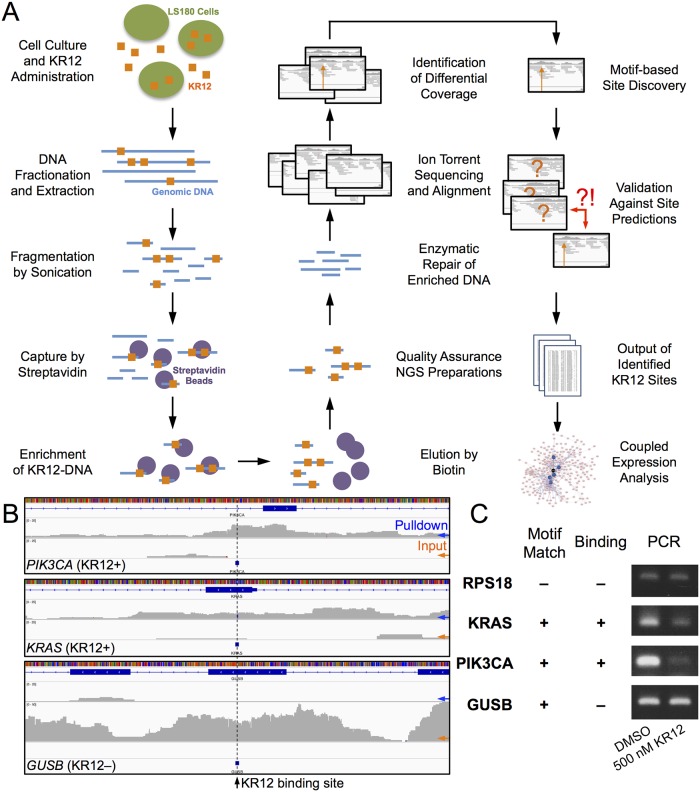
KR12 binding in the human colorectal carcinoma LS180 genome. (A) Workflow to identify KR12 binding sites in the LS180 genome by IonTorrent sequencing. Cells are administered with KR12 (500 nM, 6 h) prior to genomic DNA extraction and fragmentation by sonication. Enrichment by streptavidin allows the capture of KR12-bound nucleotides. A computational routine maps candidate regions in sequencing data, followed by site calling via motif matching and statistical validation. Subsequent microarray analyses provide the means to confirm binding data with genome-level changes. (B) Sample sequencing coverage of *PIK3CA* and *KRAS*, genes with KR12 binding sites (“KR12+”), and for reference, a predicted site by motif matching but non-binding (“KR12–”) in *GUSB*; windows centered around a KR12 site (black arrow, dashed line) are within –500 to +500 bp; blue and orange arrows indicate cumulative coverage for the pulldown and input tracks, respectively. (C) Semi-quantitative PCR of *RPS18*, *KRAS*, *PIK3CA* and *GUSB* in the presence of 500 nM KR12 or DMSO as control. “Motif match” and “binding” indicate whether a particular gene contains a computationally predetermined match to the KR12 motif in the hg19 genome or a KR12 binding site determined by sequencing analysis, respectively.

While ChIP-seq methods utilizing chemical crosslinking had been relatively mature and widely adopted, PIP or non-crosslinking sequencing experiments were relatively rare and thus less understood; as such, the extent of noise and peak qualities remained to be elucidated. Since the effect of omitting chemical crosslinking on sequencing results was unclear, we devised a different approach to identify genomic regions enriched by KR12, initially deploying a sliding-window comparison by diffReps [24] followed by a PIP-specific filtering process to find sites in differentially enriched regions of the LS180 genome (Fig 2A). We reassembled genomic sequences in the enriched peaks for matches to 5’-TGWWGGCGW-3’ (+), the KR12 9-bp motif previously found to be a full-match binder by Bind-n-Seq analysis [19]. Following sequence reassembly over indels from multi-allelic variant calling, we designed an additional statistical validation routine to determine whether candidates from peak calling did possess sufficient experimental evidence to suggest enrichment over non-binding KR12 sites. While the extra rigor might appear to be unnecessarily conservative and complicate the peak discovery process, the added safeguard allowed us to approach the exclusion of a traditionally important step more cautiously. We constructed a null background population consisting of randomly sampled predicted KR12 sites based on motif matches in the hg19 genome for differential coverage comparison by two-sample Kolmogorov-Smirnov test, a non-parametric method to account for possible deviations from normality in the heterogeneous peak distributions. After Benjamini-Hochberg corrections for multiple comparisons, we assessed statistical significance for each candidate by their 99.9^th^-percentile adjusted *p*-value.

### KR12 Binding in the Human Colorectal Cancer LS180 Genome

In total, we identified 3,343 KR12 binding sites (S1 Appendix; sample spectra in Fig 2B) in the LS180 genome, compared to 29,263 hypothetical positions in hg19. Among those, 1,556 and 23 were located within the transcripts and promoter regions (defined here as 1,000 bp upstream of transcription start site), respectively; 65 were on the list of 409 cancer-related genes [52]. These numbers were in sharp contrast to a mere list of 20 sites by MACS (S1 Table); this level of underestimation also suggested the possibility of encountering an elevated level of false negatives when adopting typical ChIP-seq procedures for PIP-based applications without modifications. Presumably, this would become more apparent with longer PIPs when their longer base-pair recognition and stronger interaction with DNA further reduced the number of possible sites.

As part of the validation, we performed PCR to check whether gene expressions of candidate genes such as *KRAS* and *PIK3CA* were disrupted by KR12 binding compared to sites that could potentially be alkylated yet not found to do so, e.g. *GUSB* (Fig 2C). Reduced transcript levels were indeed observed in *KRAS* and *PIK3CA* while *GUSB* and *RPS18* failed to show discernible differences. These results also indicated that the enzymatic repair step, performed during sequencing but not PCR, did seem to restore the covalently modified nucleotides to a sequencer-compatible stage; this was evident from the presence of differential coverage and in a previous report [19].

### Effect of KR12 Binding on Gene Expressions and Implications on *KRAS*

**Fig 3:**
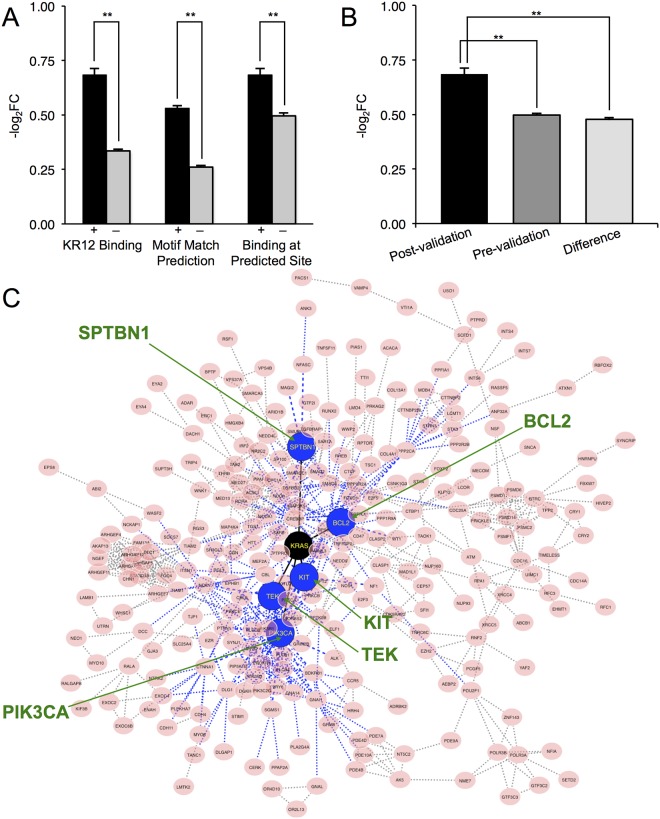
Genome-wide effect of KR12 binding and implications of mutant codon 12 *KRAS* as a driver gene. (A) Mean expressions (vertical axis,–log_2_FC) from RNA microarray analysis. Left: genome-wide KR12 binding (KR12-bound ‘+’, black; otherwise ‘–’, gray); middle: genes with computationally predetermined motif matches (‘+’) and those without (‘–’); right: expressions of genes with identified sites (‘+’) compared to genes with motif matches but no binding (‘–’) as determined by the workflow. **, *p* < .01 from two-sample Welch’s *t*-test; error bars indicate ±1 SEM. (B) Mean expressions (–log_2_FC) of identified KR12-bound genes (“Post-validation”) compared to candidate genes following sliding-window determination (“Pre-validation”); “Difference” indicates pre-validation candidates not found in the Post-validation group. Error bars, ±1 SEM; **, *p* < .01 from two-sample Welch’s *t*-test. (C) Interaction network of down-regulated KR12-bound genes. *KRAS* (black) and its first neighbors (blue, marked with green arrows) are linked by solid black edges. Blue dashed edges indicate direct interactions with first neighbors of *KRAS*.

Gene expression microarrays showed a significant difference in the mean expression of KR12-bound genes (–log_2_FC of 0.658) and those without (0.339, Fig 3A left), echoing our previous report that KR12 was capable of inducing toxicity in cancer cells. A significant decrease over the nonbinding sites was also observed (Fig 3A right). Comparing expressions of KR12-bound genes with all possible candidates prior to statistical validation also expectedly showed distinguishable reductions (Fig 3B). Coupled with *a priori* knowledge from previous Bind-n-Seq results [19], the workflow proposed here appeared to eliminate noise and false-positives by combining gene expressions. Further optimizations should allow us to address and reduce the level of false-negatives in the form of rare mismatch binding events. While gene expressions decreased upon KR12 binding, changes at the protein and subsequently the phenotypic levels were otherwise minimal due to the small fraction (~10%) of binding sites within protein coding regions. Comparing 9-mer frequencies of noncoding sections in the genome (2,000 bp centered around KR12 sites within lncRNA) with the KRAS transcript showed a relative low number of sites (138) contained with stretches of lncRNA and suggested that the possibility of KR12 affecting gene transcription via lncRNA interference was low, even given sequence similarities at the 9-mer resolution between lncRNA and the *KRAS* transcript (S2 Appendix). Coupled with our previous observations that KR12 treatment led to a reduction in Ras-GTP levels *in vivo* as well as phenotypic changes that were strongly Ras-dependent [20], it appeared that while KR12 did have a number of possible genomic targets, cellular responses to the polyamide remained overwhelmingly due to the transcriptional disruption of *KRAS* mutant codon 12. Previous results in which KR12 demonstrated appreciable toxicities in cell lines bearing *KRAS* G12D/G12V mutations over cells with wild-type or non-D/V mutations inferred the possibility of KR12 effectively targeting the mutant driver gene. We tried to confirm our analytical procedure with the SW480 genome, in which *KRAS* carried a homozygous codon 12 G12V mutation (S3 Appendix). Under the same criteria, site characterization results showed degrees of similarity between the makeup of KR12-bound genes in the two genomes and significance between the expression of KR12-bound and unbound genes. Considering that the transcriptomes of SW480 and LS180 were dynamic and thus understandably different, agreement between the two mutant *KRAS* colorectal cancer genomes at the same time infers that the approach can be extended to other genomes to identify binding sites by alkylating polyamides.

We then sought to understand if additional data exploration via interaction-association pathway analyses and a series of statistical assessments could substantiate results obtained from the workflow. From gene set enrichment analysis, we observed no statistical significance in the mean expressions of KR12-bound genes or otherwise (Table 1; S1 Fig), indicating the absence of specific effect by KR12 in those pathways despite enrichment. At the same time, KR12-bound genes exhibited an entirely different makeup of pathway overrepresentation compared to members of the Ras pathway (hsa04014 from KEGG). Expressions of KR12-bound genes in other pathways were also evaluated (S2 Table), and while some pathways did exhibit above-baseline changes in mean expressions, they frequently contained several common KR12-bound genes (S3 Table) either associated with *KRAS*; e.g. *SMURF2* [53], or participated in the same pathways as *KRAS*. As such, expression changes in those pathways could also be the consequence of *KRAS* inhibition. As such, these observations would suggest that while KR12 did bind non-*KRAS* sites non-systematically, most binding events appeared to be relatively random and did not as a whole contribute to phenotypic changes to the extent that binding to *KRAS* mutant codon 12. Network analysis of down-regulated genes (Fig 3C; S2 Fig) revealed only a small number of direct interactions stemmed from *KRAS*, namely *SPTBN1*, *BCL2*, *TEK*, *KIT* and *PIK3CA*. However, these first neighbors were heavily associated with other down-regulated genes in the same network, corroborating previous findings that KR12 binding to *KRAS* would then induce changes along the downstream pathway cascades. From these results, it appeared possible to use binding data with pathway information to generate statistically based criteria to elucidate the extent of non-systematic binding of a PIP.

**Table 1:** Statistically overrepresented pathways for KR12-bound genes, compared to members in the Ras pathway (hsa04014) alone. Fold enrichment between two groups have a *χ*^*2*^ statistic of 30.958 (*p* ~ 1.43 × 10^−4^).

Pathway	KR12	Fold	hsa04014	Fold
Histamine H1 receptor medicated signaling	9/1.75	5.15	6/0.31	19.22
VEGF signaling	13/3.55	3.66	31/0.63	48.82
Endothelin signaling	17/5.24	3.24	23/0.94	25.34
FGF signaling	23/7.41	3.10	58/1.32	43.82
Cadherin signaling	32/10.23	3.13	1/1.83^[e]^	0.55*
EGF receptor signaling	22/7.83	2.81	45/1.40	32.16
Gqα/Goα-mediated signaling	18/6.81	2.64	24/1.22	19.74
Wnt signaling	46/18.62	2.47	16/3.33	4.81
Unclassified	1012/1099.83	0.92	63/196.46	0.32

*Pathway*, overrepresented pathways determined using list of genes with KR12 binding sites via PANTHER, statistical criterion: *p* < .05 after Bonferroni correction; *KR12*, Ratio of found/expected number of genes for those with KR12 binding sites compared to human genome; *Fold*, computed enrichment fold; *hsa04014*, ratio of found/expected number of genes in the Ras pathway compared to human genome, irrespective of KR12 binding;

* Statistically insignificant (*p* ~ 1).

### Chromatin Accessibility and Other Implications

**Fig 4:**
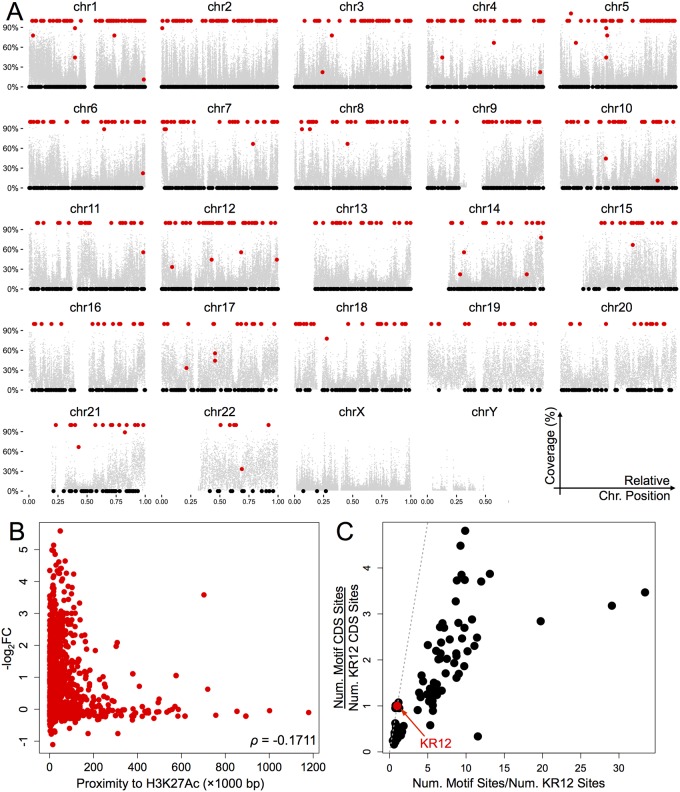
Chromatin accessibility of KR12. (A) Position-coverage plot of KR12 binding (9 bp per point, black and red) in relation to DHS regions (10,000 bp per point, gray) per chromosome. Horizontal axes, relative position along chromosome; vertical axes, relative coverage of a feature; for DHS regions, a coverage of 100% indicates that all 10,000 bp of a genomic region have DHS, while for KR12 sites, black spots (0%) indicate that a particular site is outside the boundaries of a DHS region, in contrast to the red sites (> 0%). (B) Gene expressions (vertical,–log_2_FC) as a function of KR12 site proximity (horizontal, 1000 bp) to the histone modification feature H3K27Ac. Spearman’s correlation coefficient (*ρ* = –0.171) suggests a weak correlation with histone modification. (C) Comparison of predicted binding site counts in coding regions (CDS) and in the hg19 genome for KR12 (red) against 100 randomly selected motifs (black, see S4 Table); horizontal axis, ratio of binding sites per motif/KR12 binding sites; vertical axis, ratio of binding sites in CDS per motif/KR12. Dashed line with slope of 1 (gray) is provided as reference.

The broadened search space in our pipeline led us to explore chromatin accessibility in the context of KR12 binding. Comparing site overlaps with DHS and heterochromatin mappings suggested some propensity for KR12 to access condensed chromatin (Fig 4A; ~80% DHS and 65% heterochromatin), a surprising departure from the earlier hypothesis of preferential binding in loose chromatin [54]. A weak correlation between gene expression and the relative distance from H3K27Ac peaks for intragenic KR12 binding sites (Fig 4B) also suggested some degree of binding independence from chromatin structure. Upon examining the expressions of 161 transcription factors as listed in the ENCODE clustered transcription factor binding site data, we found no significant differences in the mean or variance of expressions between KR12-bound transcription factors or otherwise (*p* = 0.142 and *p* = 0.121 from two-sample *t*- and *F*-tests, respectively); this further suggested that KR12 did not have a direct role in influencing large-scale gene transcription. Furthermore, out of the 161 transcription factors, only 67 contained predicted KR12 binding sites, and only 12 contained sites not located in the introns (S4 Table). These observations echoed a recent proposal that sufficiently small transcription factors (~ 5 nm in diameter, or < 50 kDa) could penetrate and reorganize local chromatin structure to enable transcription [55]. PIPs in general have molecular weights an order of magnitude lower than the smallest transcription factors, and thus are able to bind its targets in condensed chromatin; nonetheless, their high affinity to DNA may still induce steric hindrance to hamper transcription, leading to reduced expressions (Table 2). While such common measures to predict chromatin accessibility were sufficiently reliable, the dynamic nature behind nucleosome arrangement would still require further experimentation to verify so. Considering that previous reports of chromatin accessibility experiments only utilized synthetic or purified nucleotides in *ex vivo* settings, results here could potentially be one of the first sequencing- and expression-based evidence of the genome-wide accessibility of PIP in living cells, which further improved their attractiveness as therapeutic candidates than previously imagined.

**Table 2:** Expressions and distributions of KR12 binding in local chromatin structures.

DHS	LS180	hg19	*p*-value	Heterochromatin	LS180	hg19	*p*-value
Chromatin Ratio	0.245	0.353	4.33×10^−189^	Chromatin Ratio	0.529	0.692	5.34×10^−138^
LC+	-0.768	-0.508	6.10×10^−5^	LC+	-1.029	-0.672	9.40×10^−15^
LC–	-0.653	-0.579	3.77×10^−2^	LC–	-0.250	-0.212	*n*.*s*.

*Chromatin ratio*, odds of loose/condensed chromatin for DHS (within/outside) and heterochromatin (outside/inside); *LC+* and *LC*–, mean expressions of KR12-bound genes in loose and condensed chromatin, respectively; corresponding values from hg19 predictions are listed as reference. Statistical significance between experiment (*LS180*) and predictions (*hg19*) is assessed by Welch’s two-sample *t*-test. *p*-value is estimated from 1000 bootstrapping trials of *n* = 100; *n*.*s*., not significant.

As PIP binding appeared to be chromatin-independent and only weakly correlated with transcriptional regulation, the disconnection between gene- and protein-level changes still remained to be explored. A possibility raised in the earlier section was the surprisingly small number of binding sites in coding regions leading to fewer disruptions of changes at the protein level. We then compared the total number of possible binding sites in the hg19 genome for KR12 against 100 randomly scrambled motifs revealed a relatively small number of binding sites (~ 30 percentile) and a small fraction of coding regions (~ 43 percentile, Fig 4C); KR12’s relatively infrequent binding in the coding region could attribute to some extent the discriminating toxicity it had on cell lines with G12D/G12V mutations over wild-type.

The concept of “oncogenic addiction,” in which tumors relied on one dominant oncogene, e.g. *KRAS*, for growth and survival [56], has been advocated as possible approaches to molecular therapy; as such, the use of KR12 presents a viable option for treating mutant *KRAS* genotypes. While alkylation of occupied sites by KR12 did contribute to DNA damage and reduced gene expressions, the relatively few binding sites in promoter and coding regions perhaps explained the highly specific and directed suppression of mutant *KRAS* codon 12 by the polyamide. The relatively small subset of genomic binding by KR12 in contrast to the nonspecific alkylation of guanines and adenines by conventional alkylating neoplastic agents such as melphalan or mechlorethamine [57] could also lead to a potential reduction in the amount of adverse side effects. As more applications of PIP are explored, the need for similar sequencing and expression-driven exploratory analyses will also rise; incorporation of the workflow herein could then enhance the throughput of designing and optimizing new and existing polyamide designs, especially with further optimizations in the enrichment procedure and computational peak identification.

## Conclusion

The use of biotinylated alkylating polyamides in next-generation sequencing, followed by Kolmogorov-Smirnov-validated sliding-window enrichment identification and statistical assessment of coupled site-expression microarray data, provides a workflow to characterize possible genome-wide effect of a PIP. Assessment of statistically significant pathways based on the impact of PIP binding, intra-association of genes with binding sites in a network analysis as well as chromatin accessibility and coding-region binding frequencies also provide a basis of deciding whether a polyamide will have large-scale genetic level effects as well. The analytical steps proposed in this workflow are sufficiently modular and implementable in-line with an existing PIP design protocol.

While the identification of KR12 binding sites in the LS180 genome suggested that PIP binding was not unique only to the oncogene, evaluation of gene expressions and pathway analyses revealed that such binding events appeared to be non-systematic. In conjunction to previous reports where KR12 exerted toxicity against specific mutant cancer cell lines and demonstrate changes in cell cycle, it also suggested that non-*KRAS* binding overall had little contribution to changes at the phenotypic level.

## Supporting Information

S1 Appendix The list of identified KR12 binding sites in the LS180 genome, organized by RefSeq gene symbols and genomic positions in hg19 coordinates. (PDF)

S2 Appendix Sequence similarity among KR12-bound lncRNAs and the *KRAS* transcript. (GZ)

S3 Appendix The list of identified KR12 binding sites in the SW480 genome, organized by RefSeq gene symbols and genomic positions in hg19 coordinates. (PDF)

S4 Appendix The list of predicted KR12 binding sites in the hg19 genome found within the transcripts of common transcription factors (161 TF). (PDF)

S1 Fig Mean expressions of KR12-bound vs. unbound genes in statistically overrepresented pathways. (TIFF)

S2 Fig Vector graphics of the interaction network of downregulated KR12-bound genes (from Fig 3C). (SVG)

S1 Table List of Enriched peaks containing KR12 motifs in the LS180 dataset, using MACS 1.4.2 under default parameters for peak calling and cross-referenced to predicted KR12 binding sites in the hg19 genome. (DOCX)

S2 Table List of KEGG human pathways in which KR12 binding significantly affects the expression and has observable specific effect compared to KR12-bound genes otherwise. (DOCX)

S3 Table List of KR12-bound genes that participate in one or more KEGG pathways deemed to have statistically significant changes (from S2 Table) upon KR12 binding, and their relations to *KRAS* via the frequency of co-occurrence in *KRAS*-implicated pathways. (DOCX)

S4 Table List of Scrambled KR12 Motifs. (DOCX)

## References

[1] Meier JL, Yu AS, Korf I, Segal DJ, Dervan PB. (2012) Guiding the Design of Synthetic DNA-Binding Molecules with Massively Parallel Sequencing. J Am Chem Soc 134(42): 17814–17822. 10.1021/ja308888c 23013524 PMC3483022

[2] Taylor RD, Chandran A, Kashiwazaki G, Hashiya K, Bando T, Nagase H et al (2015) Selective Targeting of the KRAS Codon 12 Mutation Sequence by Pyrrole—Imidazole Polyamide seco-CBI Conjugates. Chem Eur J 21(42): 14996–15003. 10.1002/chem.201501870 26306751

[3] Anandhakumar C, Li Y, Kizaki S, Pandian GN, Hashiya K, Bando T et al (2014) Next-Generation Sequencing Studies Guide the Design of Pyrrole-Imidazole Polyamides with Improved Binding Specificity by the Addition of β-Alanine. ChemBioChem 15(18): 2647–2651. 10.1002/cbic.201402497 25371287

[4] Kang JS, Meier JL, Dervan PB. (2014) Design of Sequence-Specific DNA Binding Molecules for DNA Methyltransferase Inhibition. J Am Chem Soc 136(9): 3687–3694. 10.1021/ja500211z 24502234 PMC3985849

[5] Dervan PB, Edelson BS. (2003) Recognition of the DNA minor groove by pyrrole-imidazole polyamides. Curr Opin Struct Biol 13(3): 284–299. 12831879 10.1016/s0959-440x(03)00081-2

[6] Makley LN, Gestwicki JE (2013) Expanding the number of 'druggable' targets: non-enzymes and protein-protein interactions. Chem Biol Drug Des 81(1): 22–32. 10.1111/cbdd.12066 23253128 PMC3531880

[7] Ledford H. (2015) Cancer: The Ras renaissance. Nature 520(7547): 278–80. 10.1038/520278a 25877186

[8] Cox AD, Fesik SW, Kimmelman AC, Luo J, Der CJ. (2014) Drugging the undruggable RAS: Mission possible? Nat Rev Drug Discov 13(11): 828–851. 10.1038/nrd4389 25323927 PMC4355017

[9] Pandian GN, Nakano Y, Sato S, Morinaga H, Bando T, Nagase H et al (2012) A synthetic small molecule for rapid induction of multiple pluripotency genes in mouse embryonic fibroblasts. Sci Rep 2:544 10.1038/srep00544 22848790 PMC3408130

[10] Pandian GN, Sato S, Anandhakumar C, Taniguchi J, Takashima K, Junetha S et al (2014) Identification of a small molecule that turns ON the pluripotency gene circuitry in human fibroblasts. ACS Chem Biol 9(12): 2729–2736. 10.1021/cb500724t 25366962

[11] Raskatov JA, Meier JL, Puckett JW, Yang F, Ramakrishnan P, Dervan PB. (2012) Modulation of NF-*κ*B-dependent gene transcription using programmable DNA minor groove binders. Proc Natl Acad Sci U S A 109(4): 1023–1028. 10.1073/pnas.1118506109 22203967 PMC3268328

[12] Mishra R, Watanabe T, Kimura MT, Koshikawa N, Ikeda M, Uekusa S et al (2015) Identification of a novel E-box binding pyrrole-imidazole polyamide inhibiting MYC-driven cell proliferation. Cancer Sci 106(4): 421–429. 10.1111/cas.12610 25611295 PMC4406810

[13] Syed J, Chandran A, Pandian GN, Taniguchi J, Sato S, Hashiya K et al (2015) A Synthetic Transcriptional Activator of Genes Associated with the Retina in Human Dermal Fibroblasts. ChemBioChem 16(10):1497–501. 10.1002/cbic.201500140 25900774

[14] Obinata D, Ito A, Fujiwara K, Takayama K, Ashikari D, Murata Y et al (2014) Pyrrole-imidazole polyamide targeted to break fusion sites in TMPRSS2 and ERG gene fusion represses prostate tumor growth. Cancer Sci 105(10): 1272–1278. 10.1111/cas.12493 25088707 PMC4462350

[15] Chen M, Matsuda H, Wang L, Watanabe T, Kimura MT, Igarashi J et al (2010) Pretranscriptional regulation of Tgf-β1 by PI polyamide prevents scarring and accelerates wound healing of the cornea after exposure to alkali. Mol Ther 18(3): 519–527. 10.1038/mt.2009.263 19920805 PMC2839444

[16] Hargrove AE, Martinez TF, Hare AA, Kurmis AA, Phillips JW, Sud S et al (2015) Tumor Repression of VCaP Xenografts by a Pyrrole-Imidazole Polyamide. PLoS One 10(11): e0143161 10.1371/journal.pone.0143161 26571387 PMC4646452

[17] Kang JS, Meier JL, Dervan PB. (2014) Design of sequence-specific DNA binding molecules for DNA methyltransferase inhibition. J Am Chem Soc. 136(9): 3687–3694. 10.1021/ja500211z 24502234 PMC3985849

[18] Erwin GS, Bhimsaria D, Eguchi A, Ansari AZ. (2014) Mapping polyamide-DNA interactions in human cells reveals a new design strategy for effective targeting of genomic sites. Angew Chem Int Ed Engl 53(38): 10124–10128. 10.1002/anie.201405497 25066383 PMC4160732

[19] Chandran A, Syed J, Taylor RD, Kashiwazaki G, Sato S, Hashiya K et al (2016) Deciphering the genomic targets of alkylating polyamide conjugates using high-throughput sequencing. Nucleic Acids Res. 44(9): 4014–4024. 10.1093/nar/gkw283 27098039 PMC4872120

[20] Hiraoka K, Inoue T, Taylor RD, Watanabe T, Koshikawa N, Yoda H et al (2015) Inhibition of KRAS codon 12 mutants using a novel DNA-alkylating pyrrole-imidazole polyamide conjugate. Nat Commun 6: 6706 10.1038/ncomms7706 25913614

[21] Quinlan AR, Hall IM. (2010) BEDTools: a flexible suite of utilities for comparing genomic features. Bioinformatics 26(6): 841–842. 10.1093/bioinformatics/btq033 20110278 PMC2832824

[22] Li H, Handsaker B, Wysoker A, Fennell T, Ruan J, Homer N et al (2009) The Sequence Alignment/Map format and SAMtools. Bioinformatics 25(16): 2078–2079. 10.1093/bioinformatics/btp352 19505943 PMC2723002

[23] Li H. (2011) A statistical framework for SNP calling, mutation discovery, association mapping and population genetical parameter estimation from sequencing data. Bioinformatics 27(21): 2987–2993. 10.1093/bioinformatics/btr509 21903627 PMC3198575

[24] Shen L, Shao NY, Liu X, Maze I, Feng J, Nestler EJ. (2013) diffReps: detecting differential chromatin modification sites from ChIP-seq data with biological replicates. PLoS One 8(6): e65598 10.1371/journal.pone.0065598 23762400 PMC3677880

[25] Zhang Y, Liu T, Meyer CA, Eeckhoute J, Johnson DS, Bernstein BE et al (2008) Model-based analysis of ChIP-Seq (MACS). Genome Biol 9(9): R137 10.1186/gb-2008-9-9-r137 18798982 PMC2592715

[26] Broad Institute, Picard tools. Available at broadinstitute.github.io/picard

[27] McKenna A, Hanna M, Banks E, Sivachenko A, Cibulskis K, Kernytsky A et al (2010) The Genome Analysis Toolkit: a MapReduce framework for analyzing next-generation DNA sequencing data. Genome Res 20(9): 1297–1303. 10.1101/gr.107524.110 20644199 PMC2928508

[28] DePristo MA, Banks E, Poplin R, Garimella KV, Maguire JR, Hartl C et al (2011) A framework for variation discovery and genotyping using next-generation DNA sequencing data. Nat Genet 43(5): 491–498. 10.1038/ng.806 21478889 PMC3083463

[29] R Core Team (2016). R Foundation for Statistical Computing. Available at www.R-project.org

[30] Ernst J, Kellis M. (2010) Discovery and characterization of chromatin states for systematic annotation of the human genome. Nat Biotechnol 28(8): 817–825. 10.1038/nbt.1662 20657582 PMC2919626

[31] Ernst J, Kheradpour P, Mikkelsen TS, Shoresh N, Ward LD, Epstein CB et al (2011) Mapping and analysis of chromatin state dynamics in nine human cell types. Nature 473(7345): 43–49. 10.1038/nature09906 21441907 PMC3088773

[32] Rosenbloom KR, Sloan CA, Malladi VS, Dreszer TR, Learned K, Kirkup VM et al (2013) ENCODE data in the UCSC Genome Browser: year 5 update. Nucleic Acids Res 41(Database issue): D56–63. 10.1093/nar/gks1172 23193274 PMC3531152

[33] Karolchik D, Hinrichs AS, Furey TS, Roskin KM, Sugnet CW, Haussler D et al (2004) The UCSC Table Browser data retrieval tool. Nucleic Acids Res 32(Database issue): D493–496. 10.1093/nar/gkh103 14681465 PMC308837

[34] The Genome Sequencing Consortium. (2001) Initial sequencing and analysis of the human genome. Nature 409(6822): 860–921. 10.1038/35057062 11237011

[35] Wilks C, Cline MS, Weiler E, Diehkans M, Craft B, Martin C et al (2014) The Cancer Genomics Hub (CGHub): overcoming cancer through the power of torrential data. Database. 10.1093/database/bau093 25267794 PMC4178372

[36] Lajiness JP, Boger DL. (2011) Asymmetric synthesis of 1,2,9,9a-tetrahydrocyclopropa[c]benzo[e]indol-4-one (CBI). J Org Chem 76(2): 583–587. 10.1021/jo102136w 21192653 PMC3079324

[37] Ritchie ME, Phipson B, Wu D, Hu Y, Law CW, Shi W et al (2015) limma powers differential expression analyses for RNA-sequencing and microarray studies. Nucleic Acids Res 43(7): e47 10.1093/nar/gkv007 25605792 PMC4402510

[38] Kanehisa M, Sato Y, Kawashima M, Furumichi M, Tanabe M. (2016) KEGG as a reference resource for gene and protein annotation. Nucleic Acids Res 44: D457–D462. 10.1093/nar/gkv1070 26476454 PMC4702792

[39] Kanehisa M, Goto S. (2000) KEGG: Kyoto Encyclopedia of Genes and Genomes. Nucleic Acids Res 28: 27–30. 10592173 10.1093/nar/28.1.27 PMC102409

[40] Jensen LJ, Kuhn M, Stark M, Chaffron S, Creevey C, Muller J et al (2009) STRING 8—a global view on proteins and their functional interactions in 630 organisms. Nucleic Acids Res 37(Database issue): D412–D416. 10.1093/nar/gkn760 18940858 PMC2686466

[41] Shannon P, Markiel A, Ozier O, Baliga NS, Wang JT, Ramage D et al (2003) Cytoscape: a software environment for integrated models of biomolecular interaction networks. Genome Res 13(11): 2498–2504. 10.1101/gr.1239303 14597658 PMC403769

[42] Tenenbaum D (2016). KEGGREST 1.12.2: Client-side REST access to KEGG.

[43] Chang AY, Dervan PB. (2000) Strand selective cleavage of DNA by diastereomers of hairpin polyamide-*seco*-CBI conjugates. J Am Chem Soc 122(20): 4856–4864.

[44] Cabili MN, Trapnell C, Goff L, Koziol M, Tazon-Vega B, Regev A et al (2011) Integrative annotation of human large intergenic noncoding RNAs reveals global properties and specific subclasses. Genes Dev 25(18): 1915–1927. 10.1101/gad.17446611 21890647 PMC3185964

[45] Griffiths-Jones S, Grocock RJ, van Dongen S, Bateman A, Enright AJ. (2006) miRBase: microRNA sequences, targets and gene nomenclature. Nucleic Acids Res 34(Database issue): D140–144. 10.1093/nar/gkj112 16381832 PMC1347474

[46] Guillaume M, Kingsford C. (2011) A fast, lock-free approach for efficient parallel counting of occurrences of k-mers. Bioinformatics 27(6): 764–770. 10.1093/bioinformatics/btr011 21217122 PMC3051319

[47] Frith MC. (2011) A new repeat-masking method enables specific detection of homologous sequences. Nucleic Acids Res 39(4): e23 10.1093/nar/gkq1212 21109538 PMC3045581

[48] Rashid NU, Giresi PG, Ibrahim JG, Sun W, Lieb JD. (2011) ZINBA integrates local covariates with DNA-seq data to identify broad and narrow regions of enrichment, even within amplified genomic regions. Genome Biol 12(7): R67 10.1186/gb-2011-12-7-r67 21787385 PMC3218829

[49] Baek S, Sung MH, Hager GL. (2012) Quantitative analysis of genome-wide chromatin remodeling. Methods Mol Biol 833: 433–441. 10.1007/978-1-61779-477-3_26 22183609 PMC6391053

[50] Koohy H, Down TA, Spivakov M, Hubbard T. (2014) A comparison of peak callers used for DNase-Seq data. PLoS One 9(5):e96303 10.1371/journal.pone.0096303 24810143 PMC4014496

[51] Tian B, Yang J, Brasier AR. (2012) Two-step Crosslinking for Analysis of Protein-Chromatin Interactions. Methods Mol Biol 809: 105–120. 10.1007/978-1-61779-376-9_7 22113271 PMC4148016

[52] ThermoFisher Scientific. Ion AmpliSeq Comprehensive Cancer Panel. Available at thermofisher.com/order/catalog/product/4477685, last accessed Jan 2016.

[53] Shukla S, Allam US, Ahsan A, Chen G, Krishnamurthy PM, Marsh K et al (2014) KRAS protein stability is regulated through SMURF2: UBCH5 complex-mediated beta-TrCP1 degradation. Neoplasia 16(2): 115–128. 10.1593/neo.14184 24709419 PMC3978392

[54] Jespersen C, Soragni E, James Chou C, Arora PS, Dervan PB, Gottesfeld JM. (2012) Chromatin structure determines accessibility of a hairpin polyamide-chlorambucil conjugate at histone H4 genes in pancreatic cancer cells. Bioorg Med Chem Lett 22(12): 4068–4071. 10.1016/j.bmcl.2012.04.090 22607671 PMC3362666

[55] Maeshima K, Kaizu K, Tamura S, Nozaki T, Kokubo T, Takahashi K. (2015) The physical size of transcription factors is key to transcriptional regulation in chromatin domains. J Phys Condens Matter 27(6): 064116 10.1088/0953-8984/27/6/064116 25563431

[56] Weinstein B, Joe A. (2008) Oncogene Addiction. Cancer Res 68(9): 3077–3080. 10.1158/0008-5472.CAN-07-3293 18451130

[57] Polavarapu A, Stillabower JA, Stubblefield SG, Taylor WM, Baik MH. (2012) The mechanism of guanine alkylation by nitrogen mustards: a computational study. J Org Chem 77(14): 5914–5921. 10.1021/jo300351g 22681226

