# Supplementary material for: Identification of Binding Targets of a Pyrrole-Imidazole Polyamide KR12 in the LS180 Colorectal Cancer Genome
Source: PLoS One. 2016 Oct 31;11(10):e0165581. doi: 10.1371/journal.pone.0165581 (PMC5087912; doi:10.1371/journal.pone.0165581)
Supplement: S1 Appendix — (PDF) [file pone.0165581.s001.pdf]

S1 Appendix. The list of identified KR12 binding sites in the LS180 genome, organized by RefSeq gene symbols and genomic positions in hg19 coordinates. "Pattern" indicates motif of the binding site on the (+) strand. Fold enrichments ("FE"), changes in gene expressions ("FC") and *p*-values ("p_{BH99}") are listed in $\log_2$ scales. Fold enrichments are defined as the ratio of the maximum per-base coverage within a 309 bp window centered at the KR12 binding site for the pulldown and input datasets, and capped at a maximum value of 5. Gene expressions are experimentally determined fold changes for KR12 vs. DMSO (see "Expression Microarrays") for sites residing within the transcript or promoter (defined as within 1000 bp upstream of the transcription start site) of a particular gene, and NA otherwise. The 99th percentile p-value, after Benjamini-Hochberg corrections for multiple Kolmogorov-Smirnov comparisons, is listed for a given KR12 binding site. Under "Significance", if $p_{BH99} < 0.05$ for a particular site, it is considered significant; for sites with $0.05 < p_{BH99} < 0.055$, they are considered marginal. Sites with $p_{BH99} \geq 0.055$ are deemed statistically insignificant and consequently omitted.

Symbol	Chromosome	Site Position	Pattern	log_2 FE	log_2 FC	log_2 p_{BH99}	Significance
Intergenic	chr1	943635-943643	TGATGGCGA	2.1699	NA	-7.6316	Significant
C1orf159	chr1	1031151-1031159	TCGCCATCA	1.5850	-1.3106	-6.8473	Significant
TTLL10	chr1	1122014-1122022	TCGCCAACA	-1.1375	0.0084	-7.0772	Significant
Intergenic	chr1	4335306-4335314	TGAAGGCGA	2.0000	NA	-8.4534	Significant
Intergenic	chr1	4600121-4600129	TGTTGGCGA	5.0000	NA	-9.3627	Significant
Intergenic	chr1	4601578-4601586	TGTTGGCGT	5.0000	NA	-21.9498	Significant
CAMTA1	chr1	7149870-7149878	ACGCCATCA	5.0000	0.1408	-5.7411	Significant
CAMTA1	chr1	7443243-7443251	ACGCCATCA	3.1699	0.1408	-6.4691	Significant
CAMTA1	chr1	7486697-7486705	TGATGGCGA	1.3219	0.1408	-7.4699	Significant
CAMTA1	chr1	7813079-7813087	TCGCCAACA	3.7004	0.1408	-6.8990	Significant
RERE	chr1	8774469-8774477	ACGCCTTCA	5.0000	-1.0811	-7.0772	Significant
RERE	chr1	8836504-8836512	ACGCCATCA	5.0000	-1.0811	-8.0891	Significant
SLC25A33	chr1	9617620-9617628	TGTTGGCGT	5.0000	-0.8973	-7.0772	Significant
PIK3CD	chr1	9740887-9740895	ACGCCAACA	2.3219	0.3511	-6.3198	Significant
VPS13D	chr1	12484010-12484018	TGAAGGCGA	5.0000	-0.1975	-7.4701	Significant
Promoter_HNRNPCL1	chr1	12909238-12909246	TCGCCTTCA	-0.5305	-0.0202	-5.2534	Significant
PDPN	chr1	13918848-13918856	TGTTGGCGT	2.3219	-0.2445	-8.0891	Significant
PRDM2	chr1	14088539-14088547	TGTAGGCGT	-1.2479	-1.8767	-4.3961	Significant
PRDM2	chr1	14124599-14124607	TCGCCAACA	1.0000	-1.8767	-9.1342	Significant
KAZN	chr1	15187447-15187455	TCGCCTTCA	5.0000	-0.1223	-17.2082	Significant
FHAD1	chr1	15589452-15589460	TCGCCAACA	3.3219	-0.0285	-11.4243	Significant
NBPF1	chr1	16896439-16896447	TCGCCATCA	-0.5850	NA	-4.4469	Significant
Intergenic	chr1	17515573-17515581	TCGCCAACA	-1.8745	NA	-6.8473	Significant
ACTL8	chr1	18112718-18112726	ACGCCATCA	0.3219	0.1052	-6.1002	Significant
ACTL8	chr1	18112836-18112844	TCGCCTTCA	-0.4150	0.1052	-5.3906	Significant
Intergenic	chr1	20456701-20456709	TGTAGGCGT	0.2224	NA	-9.3124	Significant
EIF4G3	chr1	21322812-21322820	TGATGGCGA	0.2630	-0.4371	-6.1002	Significant
TCEA3	chr1	23725407-23725415	ACGCCATCA	0.0000	0.2010	-6.6938	Significant
MYOM3	chr1	24415013-24415021	ACGCCAACA	1.4594	-0.3133	-6.1006	Significant
Intergenic	chr1	24591519-24591527	TCGCCTTCA	1.4150	NA	-6.4691	Significant
Intergenic	chr1	24724986-24724994	ACGCCTACA	3.9069	NA	-4.9350	Significant
TMEM57	chr1	25799292-25799300	TGATGGCGA	2.3219	-2.0821	-19.6929	Significant
Intergenic	chr1	26239258-26239265	TGAAGGCGA	-0.7105	NA	-7.2864	Significant
TMEM222	chr1	27653850-27653858	TGTTGGCGT	2.0000	0.4426	-10.9476	Significant
WASF2	chr1	27786900-27786908	TGTTGGCGA	0.0000	-1.0013	-6.6938	Significant
PUM1	chr1	31429837-31429845	ACGCCTACA	5.0000	-4.9829	-6.6938	Significant
CSMD2	chr1	34470135-34470143	ACGCCATCA	2.5850	0.2179	-5.9549	Significant
Intergenic	chr1	35588288-35588296	ACGCCAACA	3.7004	NA	-10.0223	Significant
ZMYM4	chr1	35738848-35738856	TGAAGGCGT	-1.0000	-1.1433	-5.0497	Significant
Intergenic	chr1	37240884-37240892	ACGCCAACA	2.4594	NA	-16.1337	Significant
PPIEL	chr1	40000306-40000314	ACGCCATCA	3.5850	-0.0963	-4.5890	Significant
TRIT1	chr1	40310687-40310695	TGATGGCGA	-0.4475	-1.0311	-6.1002	Significant
SCMH1	chr1	41526569-41526577	TGTTGGCGA	2.5850	-0.4557	-10.6711	Significant
Intergenic	chr1	42598022-42598030	TCGCCAACA	5.0000	NA	-6.8990	Significant
ST3GAL3	chr1	44253425-44253433	TGAAGGCGA	2.4150	-0.2537	-7.2352	Significant
ERI3	chr1	44724079-44724087	ACGCCTTCA	3.3219	0.0356	-10.4804	Significant
RNF220	chr1	44935651-44935659	TGAAGGCGT	5.0000	-1.1893	-13.6405	Significant
RNF220	chr1	44941492-44941500	TCGCCAACA	5.0000	-1.1893	-7.6831	Significant
C1orf228	chr1	45173803-45173811	ACGCCTTCA	0.0000	0.0095	-6.4695	Significant
EIF2B3	chr1	45333062-45333070	TCGCCTACA	-1.4330	-0.6451	-4.9165	Significant
ZSWIM5	chr1	45661599-45661607	ACGCCATCA	1.0000	-2.5865	-9.5736	Significant
PIK3R3	chr1	46510205-46510213	TCGCCTACA	0.4150	-1.5321	-7.6316	Significant
Intergenic	chr1	47373588-47373596	TCGCCATCA	-0.0995	NA	-7.6831	Significant
ELAVL4	chr1	50593125-50593133	TGATGGCGA	1.4594	0.0302	-7.4699	Significant
OSBPL9	chr1	52110703-52110711	ACGCCATCA	1.5850	-0.8341	-8.4534	Significant
Intergenic	chr1	52604747-52604755	TCGCCATCA	0.1255	NA	-6.4691	Significant
ZCCHC11	chr1	52923346-52923354	ACGCCTACA	0.7370	-3.3061	-10.6711	Significant
GLIS1	chr1	54198236-54198244	TGAAGGCGA	1.7004	0.0269	-14.4800	Significant
Intergenic	chr1	55387636-55387644	TGTTGGCGT	2.3219	NA	-7.2348	Significant
Intergenic	chr1	56119938-56119945	TGAAGGCGA	0.0000	NA	-8.0378	Significant
DAB1	chr1	58192719-58192727	TGATGGCGA	2.7004	0.1412	-6.8990	Significant
Intergenic	chr1	59506155-59506163	ACGCCAACA	2.4594	NA	-20.9539	Significant
Intergenic	chr1	59552467-59552475	TCGCCTTCA	-1.2016	NA	-7.8720	Significant
Intergenic	chr1	59699385-59699393	TCGCCATCA	1.9260	NA	-10.2581	Significant

Intergenic	chr1	60719021-60719029	TCGCCTTCA	3.7004	NA	-6.6938	Significant
Intergenic	chr1	60756335-60756343	TGAAGGCGA	5.0000	NA	-15.0219	Significant
NFIA	chr1	61606692-61606700	ACGCCAACA	3.5850	-1.3424	-5.2533	Significant
NFIA	chr1	61869204-61869212	TGAAGGCGA	2.4594	-1.3424	-4.7186	Significant
Intergenic	chr1	61979547-61979555	TCGCCAACA	2.7004	NA	-10.0223	Significant
Intergenic	chr1	62010787-62010795	TGTAGGCGT	5.0000	NA	-4.5890	Significant
INADL	chr1	62457247-62457255	TCGCCATCA	5.0000	-0.5878	-13.6405	Significant
INADL	chr1	62482401-62482409	TGATGGCGT	0.4594	-0.5878	-5.3906	Significant
ROR1	chr1	64258959-64258967	TGATGGCGA	1.0000	-0.1527	-10.0223	Significant
ROR1	chr1	64304592-64304600	TCGCCTACA	5.0000	-0.1527	-4.5890	Significant
ROR1	chr1	64329312-64329320	TGATGGCGT	-0.8845	-0.1527	-6.3196	Significant
DNAJC6	chr1	65754230-65754238	TGTAGGCGA	5.0000	-3.4095	-8.2802	Significant
DNAJC6	chr1	65796618-65796626	TGAAGGCGA	1.1155	-3.4095	-9.3124	Significant
PDE4B	chr1	66736590-66736598	TCGCCTTCA	5.0000	-0.9363	-8.9289	Significant
Intergenic	chr1	66924841-66924849	TGATGGCGA	5.0000	NA	-4.7567	Significant
GNG12	chr1	68275450-68275457	TGATGGCGA	5.0000	-0.0033	-4.5684	Significant
Intergenic	chr1	68521171-68521179	TGAAGGCGT	2.3219	NA	-8.0378	Significant
SRSF11	chr1	70677124-70677132	TCGCCTACA	1.4594	0.1652	-7.6833	Significant
Intergenic	chr1	71157672-71157680	TGATGGCGT	0.4150	NA	-12.3712	Significant
Intergenic	chr1	71206974-71206982	TGTTGGCGT	0.2895	NA	-4.3965	Significant
NEGR1	chr1	72366619-72366627	TCGCCATCA	5.0000	0.2130	-8.6980	Significant
NEGR1	chr1	72375881-72375889	TGTTGGCGA	2.7004	0.2130	-9.1342	Significant
NEGR1	chr1	72614522-72614530	TCGCCTTCA	-0.6674	0.2130	-6.8473	Significant
Intergenic	chr1	72948416-72948424	TCGCCTACA	1.4594	NA	-9.1249	Significant
Intergenic	chr1	73232553-73232561	ACGCCATCA	3.5850	NA	-10.0064	Significant
Intergenic	chr1	75088356-75088364	TGTAGGCGA	5.0000	NA	-6.8990	Significant
Intergenic	chr1	75477253-75477261	TCGCCTTCA	3.4594	NA	-7.2352	Significant
SLC44A5	chr1	75835666-75835674	ACGCCATCA	5.0000	0.2148	-6.1002	Significant
Intergenic	chr1	77144757-77144765	TGATGGCGT	5.0000	NA	-6.1002	Significant
Intergenic	chr1	77181738-77181746	ACGCCTTCA	1.5850	NA	-13.9006	Significant
ST6GALNAC5	chr1	77460346-77460354	TCGCCAACA	1.3219	0.2198	-8.0382	Significant
AK5	chr1	77781374-77781382	TGTAGGCGA	5.0000	-0.0576	-11.4244	Significant
USP33	chr1	78194069-78194077	TCGCCTTCA	1.4594	-0.8278	-14.2110	Significant
Intergenic	chr1	79299117-79299125	ACGCCATCA	-0.2996	NA	-6.3196	Significant
ELTD1	chr1	79470418-79470426	TGAAGGCGA	-0.1375	0.0712	-7.0774	Significant
Intergenic	chr1	79797178-79797186	TGTTGGCGA	3.0000	NA	-5.9549	Significant
Intergenic	chr1	80750527-80750535	TGTAGGCGA	1.7004	NA	-7.2352	Significant
Intergenic	chr1	81069991-81069999	ACGCCAACA	-1.6521	NA	-9.8058	Significant
Intergenic	chr1	81745635-81745643	TCGCCTTCA	5.0000	NA	-6.1522	Significant
Intergenic	chr1	82052706-82052714	TCGCCAACA	1.5025	NA	-13.6409	Significant
Intergenic	chr1	82526174-82526182	TCGCCATCA	4.0000	NA	-8.4534	Significant
Intergenic	chr1	82835619-82835627	ACGCCTTCA	5.0000	NA	-5.9549	Significant
MCOLN3	chr1	85502427-85502435	ACGCCTTCA	5.0000	0.1557	-5.1011	Significant
COL24A1	chr1	86353023-86353031	TGAAGGCGA	1.5850	0.2348	-7.6316	Significant
Intergenic	chr1	87576089-87576097	ACGCCAACA	2.0000	NA	-8.2834	Significant
Intergenic	chr1	87576415-87576423	TGAAGGCGT	0.1375	NA	-5.7926	Significant
LMO4	chr1	87808345-87808353	TGTTGGCGT	1.4854	-0.8934	-5.7407	Significant
Intergenic	chr1	88022485-88022493	TGTAGGCGA	1.1699	NA	-6.6941	Significant
Intergenic	chr1	88039436-88039444	TGTTGGCGT	5.0000	NA	-7.2348	Significant
Intergenic	chr1	89114065-89114073	TGAAGGCGT	1.5850	NA	-9.9762	Significant
LRRC8B	chr1	90037794-90037802	TGTAGGCGA	2.8074	-2.6588	-8.0378	Significant
LRRC8D	chr1	90367743-90367751	TGAAGGCGT	-0.7370	-1.6934	-8.0378	Significant
Intergenic	chr1	91306488-91306496	ACGCCAACA	0.3626	NA	-5.7411	Significant
HFM1	chr1	91814909-91814917	TCGCCTTCA	1.8745	-0.0123	-8.0378	Significant
Intergenic	chr1	91955537-91955545	TCGCCAACA	0.3785	NA	-8.2807	Significant
BTBD8	chr1	92548917-92548924	TCGCCATCA	1.7370	-0.4417	-9.5610	Significant
FAM69A	chr1	93378394-93378402	ACGCCTACA	2.4594	-1.7336	-6.8473	Significant
BCAR3	chr1	94070371-94070379	ACGCCAACA	0.8931	-1.9412	-7.2348	Significant
Intergenic	chr1	94727157-94727165	TCGCCAACA	5.0000	NA	-10.7198	Significant
ABCD3	chr1	94928395-94928403	TGATGGCGA	0.8931	-0.3822	-9.7560	Significant
Intergenic	chr1	95131606-95131614	TGTTGGCGA	2.7004	NA	-7.4701	Significant
Intergenic	chr1	96048317-96048325	TGATGGCGA	2.1699	NA	-15.2920	Significant
MIR137HG	chr1	98455588-98455596	TGTTGGCGT	5.0000	NA	-8.6938	Significant
Intergenic	chr1	98743737-98743744	TCGCCAACA	1.1699	NA	-8.8782	Significant
Intergenic	chr1	98743737-98743740	TCGCCAACA	2.2224	NA	-8.9289	Significant

Intergenic	chr1	99618609-99618617	ACGCCAACA	2.4594	NA	-4.2708	Marginal
Intergenic	chr1	99659062-99659070	TCGCCAACA	-0.3219	NA	-5.7407	Significant
Intergenic	chr1	99659412-99659420	TGAAGGCGT	3.4594	NA	-7.2348	Significant
CDC14A	chr1	100951069-100951077	TGATGGCGT	5.0000	-0.3481	-15.0219	Significant
Intergenic	chr1	105469779-105469787	ACGCCAACA	3.3219	NA	-8.9289	Significant
Intergenic	chr1	106288046-106288054	TGTTGGCGT	-1.3923	NA	-9.3124	Significant
Intergenic	chr1	106333425-106333433	TGAAGGCGT	0.4854	NA	-7.4699	Significant
C1orf194	chr1	109654229-109654237	ACGCCTACA	5.0000	-0.0472	-8.4534	Significant
SLC16A4	chr1	110924478-110924486	TGATGGCGA	5.0000	0.2004	-7.8720	Significant
Intergenic	chr1	111295340-111295348	TGTTGGCGT	-0.4150	NA	-5.4421	Significant
Intergenic	chr1	111401005-111401013	TGTAGGCGA	0.8931	NA	-10.0223	Significant
Intergenic	chr1	111411691-111411699	TGTTGGCGA	1.5850	NA	-6.3196	Significant
Intergenic	chr1	111812058-111812066	ACGCCTTCA	1.7004	NA	-4.9166	Significant
TMIGD3	chr1	112063017-112063025	TCGCCAACA	1.1699	0.0958	-6.3196	Significant
CTTNBP2NL	chr1	112991668-112991676	TGATGGCGA	2.3219	-2.4232	-7.0774	Significant
Intergenic	chr1	116466300-116466308	TGATGGCGT	-0.1520	NA	-7.2352	Significant
Intergenic	chr1	116775019-116775027	TGAAGGCGA	1.0875	NA	-6.8477	Significant
Intergenic	chr1	117580193-117580201	ACGCCATCA	-1.1375	NA	-8.4534	Significant
TBX15	chr1	119430972-119430980	TCGCCAACA	5.0000	0.0158	-15.0219	Significant
Intergenic	chr1	142659510-142659518	TCGCCTTCA	5.0000	NA	-8.2848	Significant
Intergenic	chr1	142678510-142678518	ACGCCTTCA	0.1699	NA	-5.7407	Significant
Intergenic	chr1	142866428-142866436	TGAAGGCGA	1.5850	NA	-19.3678	Significant
Intergenic	chr1	143156603-143156611	ACGCCATCA	0.1699	NA	-6.1520	Significant
Intergenic	chr1	143174980-143174988	ACGCCTTCA	5.0000	NA	-8.0348	Significant
Intergenic	chr1	143177291-143177299	ACGCCTTCA	2.5850	NA	-11.6709	Significant
Intergenic	chr1	146645292-146645300	TGTTGGCGT	5.0000	NA	-6.4691	Significant
LOC645166	chr1	148938777-148938785	TCGCCTTCA	5.0000	0.2132	-7.2348	Significant
LOC645166	chr1	148943896-148943904	TCGCCAACA	0.2224	0.2132	-8.4534	Significant
LOC645166	chr1	148952528-148952536	TGTTGGCGA	2.4594	0.2132	-7.4699	Significant
Promoter_CGN	chr1	151483156-151483164	TGTAGGCGT	0.7655	-1.3017	-5.2699	Significant
Intergenic	chr1	151582185-151582193	TCGCCTTCA	2.1699	NA	-7.2348	Significant
Intergenic	chr1	152985472-152985480	TGTAGGCGT	-1.1155	NA	-10.0223	Significant
Intergenic	chr1	153325255-153325263	ACGCCTTCA	0.6521	NA	-7.6320	Significant
Intergenic	chr1	153577011-153577019	TCGCCTACA	-0.3785	NA	-5.5994	Significant
Intergenic	chr1	153688899-153688907	TCGCCAACA	0.2224	NA	-5.0497	Significant
ADAR	chr1	154582428-154582436	TCGCCTTCA	-1.0000	-0.0788	-7.8722	Significant
Intergenic	chr1	154652057-154652065	TGAAGGCGT	5.0000	NA	-4.5681	Significant
Intergenic	chr1	154658413-154658421	TCGCCATCA	5.0000	NA	-6.3196	Significant
DAP3	chr1	155660286-155660294	ACGCCATCA	5.0000	-0.0513	-8.5043	Significant
ARHGEF11	chr1	156998299-156998307	TGATGGCGA	0.3785	-1.7921	-5.0497	Significant
Intergenic	chr1	157041020-157041028	TGTTGGCGA	5.0000	NA	-6.5211	Significant
Intergenic	chr1	159076962-159076970	TCGCCATCA	0.4854	NA	-5.3906	Significant
CD84	chr1	160538935-160538943	TGATGGCGT	5.0000	-0.1148	-6.4695	Significant
Intergenic	chr1	160843484-160843492	TCGCCAACA	-1.0000	NA	-5.2534	Significant
ATF6	chr1	161764786-161764794	TGATGGCGA	0.7776	-0.5035	-4.5893	Significant
NOS1AP	chr1	162256395-162256403	ACGCCAACA	2.0000	-0.6212	-13.9011	Significant
Intergenic	chr1	162463578-162463584	TGATGGCGT	2.2224	NA	-7.6320	Significant
DDR2	chr1	162703650-162703658	TCGCCTACA	-0.5850	-0.0759	-8.0382	Significant
Intergenic	chr1	163907733-163907741	TCGCCATCA	5.0000	NA	-5.4423	Significant
Intergenic	chr1	164111908-164111916	TCGCCAACA	-0.2801	NA	-12.9106	Significant
Intergenic	chr1	164939694-164939702	TGATGGCGA	3.5850	NA	-7.4701	Significant
Intergenic	chr1	166630359-166630367	TGAAGGCGA	3.7004	NA	-7.2424	Significant
Intergenic	chr1	166992519-166992529	TGTTGGCGT	2.3219	NA	-4.2573	Marginal
POU2F1	chr1	167226300-167226308	TGTTGGCGA	5.0000	-2.1300	-10.9249	Significant
DCAF6	chr1	168040650-168040658	TCGCCAACA	5.0000	-0.1205	-11.6236	Significant
Intergenic	chr1	168231000-168231008	TGAAGGCGT	5.0000	NA	-4.3965	Significant
Intergenic	chr1	168244905-168244913	TGTAGGCGT	1.8745	NA	-9.3124	Significant
Intergenic	chr1	168892190-168892198	TGATGGCGA	3.9069	NA	-8.7041	Significant
Intergenic	chr1	168940136-168940144	ACGCCTTCA	0.5305	NA	-10.2089	Significant
Intergenic	chr1	168970840-168970848	TGAAGGCGA	0.0000	NA	-6.6938	Significant
Intergenic	chr1	168977394-168977401	TCGCCATCA	2.0000	NA	-6.4691	Significant
Intergenic	chr1	169070151-169070159	TGAAGGCGT	1.2630	NA	-7.0772	Significant
NME7	chr1	169195629-169195637	TGTTGGCGA	5.0000	-0.3815	-7.6316	Significant
SELP	chr1	169588211-169588219	TGATGGCGT	-0.1375	0.2950	-8.2834	Significant
Intergenic	chr1	170367836-170367844	TGAAGGCGA	0.4150	NA	-11.1427	Significant

Intergenic	chr1 170744571-170744579	ACGCCTACA	5.0000	NA	-19.9815	Significant
Intergenic	chr1 170835702-170835710	ACGCCAACA	1.1699	NA	-5.9550	Significant
Intergenic	chr1 170962012-170962020	TGTAGGCGT	2.0000	NA	-6.3196	Significant
VAMP4	chr1 171683720-171683728	TCGCCAACA	5.0000	-0.3935	-15.1398	Significant
DNM3OS	chr1 172110819-172110827	TGTAGGCGA	5.0000	-0.1745	-10.4804	Significant
DNM3	chr1 172110819-172110827	TGTAGGCGA	3.0000	-1.3406	-5.9549	Significant
Intergenic	chr1 172839802-172839810	TGATGGCGT	1.4150	NA	-7.6324	Significant
Intergenic	chr1 173148987-173148995	TCGCCTTCA	1.7370	NA	-6.8990	Significant
RABGAP1L	chr1 174293238-174293246	TGTAGGCGA	3.4594	-1.1298	-6.1520	Significant
KIAA0040	chr1 175136871-175136879	TGATGGCGT	5.0000	-0.8302	-7.3789	Significant
Intergenic	chr1 176183366-176183374	TCGCCTTCA	5.0000	NA	-9.3124	Significant
Intergenic	chr1 177253234-177253242	TGATGGCGT	1.1926	NA	-5.0497	Significant
Intergenic	chr1 177538898-177538906	TCGCCATCA	-0.1375	NA	-8.0378	Significant
Intergenic	chr1 178487518-178487526	ACGCCATCA	2.4594	NA	-6.4691	Significant
CEP350	chr1 180080369-180080377	TCGCCATCA	0.0000	-3.0502	-12.6134	Significant
XPR1	chr1 180832551-180832559	ACGCCTTCA	5.0000	-0.3142	-16.3722	Significant
CACNA1E	chr1 181484914-181484922	TGAAGGCGA	2.1155	-0.1961	-5.6142	Significant
CACNA1E	chr1 181611496-181611504	TGATGGCGA	2.4594	-0.1961	-5.0501	Significant
CACNA1E	chr1 181680484-181680492	TGATGGCGT	1.3785	-0.1961	-7.4731	Significant
LAMC2	chr1 183156344-183156352	TGATGGCGT	1.9260	0.1365	-5.5994	Significant
Intergenic	chr1 183524538-183524546	TGTTGGCGA	5.0000	NA	-7.2864	Significant
RGL1	chr1 183770848-183770856	ACGCCAACA	5.0000	-2.8021	-10.9476	Significant
RGL1	chr1 183883986-183883994	TGAAGGCGA	3.3219	-2.8021	-9.8058	Significant
C1orf21	chr1 184505388-184505396	TGTTGGCGT	-0.4854	-1.1991	-7.6320	Significant
RNF2	chr1 185060543-185060551	TGTAGGCGA	5.0000	-2.4214	-5.0027	Significant
SWT1	chr1 185186186-185186194	TGAAGGCGA	0.4150	-1.0792	-6.8473	Significant
IVNS1ABP	chr1 185270896-185270904	ACGCCTACA	3.1699	-2.0161	-7.0774	Significant
Intergenic	chr1 185468886-185468894	TGTAGGCGA	-0.5850	NA	-5.0497	Significant
Intergenic	chr1 185519075-185519083	TGATGGCGT	2.5850	NA	-5.2533	Significant
Intergenic	chr1 185587812-185587820	ACGCCATCA	5.0000	NA	-10.9476	Significant
Intergenic	chr1 186467983-186467991	TGTTGGCGT	2.4594	NA	-6.3196	Significant
Intergenic	chr1 186625972-186625980	ACGCCAACA	-0.8845	NA	-10.8753	Significant
PLA2G4A	chr1 186867657-186867665	ACGCCATCA	1.3219	-0.8515	-5.7678	Significant
Intergenic	chr1 187085561-187085569	TGTAGGCGA	-1.0995	NA	-6.5213	Significant
Intergenic	chr1 187581046-187581054	TGATGGCGT	1.7370	NA	-19.6933	Significant
Intergenic	chr1 191962832-191962840	TCGCCATCA	-0.2895	NA	-5.0497	Significant
Intergenic	chr1 191984005-191984013	TGATGGCGT	3.7004	NA	-6.6938	Significant
Intergenic	chr1 192200104-192200112	ACGCCAACA	3.1699	NA	-8.8782	Significant
Intergenic	chr1 194264082-194264090	TGTAGGCGA	1.8074	NA	-4.9360	Significant
Intergenic	chr1 196099568-196099576	TGATGGCGA	0.4150	NA	-5.0497	Significant
CFHR5	chr1 196965726-196965734	TGATGGCGA	5.0000	-0.3488	-12.1138	Significant
CRB1	chr1 197266291-197266299	ACGCCATCA	-1.4594	0.2895	-6.6938	Significant
Intergenic	chr1 198579089-198579097	ACGCCTTCA	5.0000	NA	-5.4421	Significant
Intergenic	chr1 198905742-198905750	TCGCCTTCA	2.4594	NA	-7.0775	Significant
Intergenic	chr1 204563376-204563384	ACGCCTACA	5.0000	NA	-15.0219	Significant
NFASC	chr1 204852089-204852097	ACGCCTACA	2.5850	-0.0181	-5.4213	Significant
DSTYK	chr1 205144781-205144789	ACGCCAACA	3.4594	-2.1683	-7.0772	Significant
RASSF5	chr1 206684582-206684590	TGATGGCGA	3.3219	-0.4489	-6.8477	Significant
EIF2D	chr1 206765306-206765314	ACGCCTACA	1.1699	0.0284	-8.7043	Significant
EIF2D	chr1 206768335-206768343	ACGCCTTCA	5.0000	0.0284	-6.8990	Significant
C4BPA	chr1 207290190-207290198	ACGCCAACA	5.0000	0.2457	-4.3961	Significant
CR1	chr1 207770947-207770955	TGTTGGCGA	2.4594	0.0418	-10.6711	Significant
Intergenic	chr1 208644886-208644894	TCGCCATCA	5.0000	NA	-4.9360	Significant
Intergenic	chr1 208785542-208785550	TCGCCTTCA	0.3219	NA	-11.4243	Significant
Intergenic	chr1 209007256-209007264	TGTTGGCGA	1.1699	NA	-8.2834	Significant
Intergenic	chr1 209119164-209119172	TCGCCTACA	5.0000	NA	-5.7407	Significant
Intergenic	chr1 209233385-209233393	TGTTGGCGA	5.0000	NA	-5.5994	Significant
TRAF3IP3	chr1 209947039-209947047	TGATGGCGT	5.0000	0.2671	-10.9476	Significant
Intergenic	chr1 210492541-210492549	TCGCCAACA	2.0000	NA	-8.4538	Significant
INTS7	chr1 212161252-212161260	TGATGGCGA	5.0000	-1.9140	-23.8795	Significant
Intergenic	chr1 213792299-213792307	TGATGGCGA	2.7004	NA	-7.2352	Significant
Intergenic	chr1 214278875-214278883	ACGCCATCA	3.5850	NA	-9.3136	Significant
Intergenic	chr1 214332685-214332693	ACGCCTACA	-0.1926	NA	-7.4699	Significant
PTPN14	chr1 214564987-214564995	ACGCCTTCA	1.4594	-2.8435	-5.0497	Significant
PTPN14	chr1 214684866-214684874	TGTTGGCGA	-0.8931	-2.8435	-5.7407	Significant

KCNK2	chr1 215318169-215318177	ACGCCTTCA	1.8074	0.2122	-10.2093	Significant
USH2A	chr1 216367748-216367756	TCGCCATCA	3.0000	0.0053	-6.1227	Significant
USH2A	chr1 216395769-216395777	ACGCCAACA	3.7004	0.0053	-5.7407	Significant
USH2A	chr1 216506813-216506821	ACGCCATCA	3.8074	0.0053	-4.7182	Significant
ESRRG	chr1 217184414-217184422	ACGCCATCA	-1.0000	0.2479	-6.1520	Significant
Intergenic	chr1 219452681-219452689	ACGCCATCA	3.3219	NA	-6.3297	Significant
Intergenic	chr1 219684911-219684919	TCGCCAACA	1.1155	NA	-7.2348	Significant
RAB3GAP2	chr1 220409976-220409984	ACGCCTTCA	5.0000	-1.8571	-6.1224	Significant
MARK1	chr1 220831141-220831149	TGAAGGCGA	0.5406	-0.1263	-7.6831	Significant
Intergenic	chr1 222080258-222080266	TCGCCAACA	0.7370	NA	-8.6982	Significant
Intergenic	chr1 224059745-224059753	TCGCCTTCA	3.7004	NA	-10.2581	Significant
CNIH4	chr1 224563736-224563744	ACGCCAACA	3.5850	0.0189	-4.9168	Significant
Intergenic	chr1 224768831-224768838	TGTTGGCGA	1.7370	NA	-12.1138	Significant
ENAH	chr1 225729941-225729949	ACGCCAACA	5.0000	-0.4574	-8.9289	Significant
Intergenic	chr1 226207542-226207550	TCGCCAACA	-0.3479	NA	-8.2838	Significant
Intergenic	chr1 226328335-226328343	ACGCCTACA	3.5850	NA	-6.8473	Significant
Intergenic	chr1 226378429-226378437	TGATGGCGT	3.0875	NA	-13.1671	Significant
PARP1	chr1 226552830-226552838	TCGCCTTCA	3.4594	0.1499	-5.7407	Significant
Intergenic	chr1 227023058-227023066	TCGCCTTCA	3.8074	NA	-6.8990	Significant
Intergenic	chr1 228185969-228185977	ACGCCAACA	1.2224	NA	-9.1342	Significant
Intergenic	chr1 231144554-231144561	TCGCCTTCA	0.0000	NA	-8.0378	Significant
Intergenic	chr1 231146758-231146766	TGTTGGCGT	0.8074	NA	-8.2802	Significant
Intergenic	chr1 232193619-232193627	ACGCCTTCA	-0.5850	NA	-7.8720	Significant
Intergenic	chr1 232869729-232869737	TGATGGCGT	1.8074	NA	-5.2533	Significant
Intergenic	chr1 233036816-233036824	TGAAGGCGA	5.0000	NA	-6.8473	Significant
KIAA1804	chr1 233520418-233520426	TGTTGGCGT	1.1375	-1.8313	-5.3906	Significant
Intergenic	chr1 233721533-233721541	TGTAGGCGT	3.3219	NA	-13.6846	Significant
KCNK1	chr1 233800769-233800777	TGTTGGCGT	2.5850	-1.4016	-4.5681	Significant
Intergenic	chr1 234485070-234485078	ACGCCTACA	5.0000	NA	-7.8720	Significant
Intergenic	chr1 235703670-235703678	TGAAGGCGT	0.4150	NA	-8.0378	Significant
LGALS8	chr1 236712269-236712277	ACGCCTACA	5.0000	-0.5778	-12.6134	Significant
Intergenic	chr1 238841887-238841895	TCGCCTTCA	2.1699	NA	-7.5855	Significant
Intergenic	chr1 239345489-239345497	TGTTGGCGA	5.0000	NA	-8.1717	Significant
Intergenic	chr1 239453163-239453171	TGTTGGCGA	0.2224	NA	-7.6831	Significant
Intergenic	chr1 240220838-240220846	ACGCCTTCA	1.2224	NA	-8.5043	Significant
FMN2	chr1 240482556-240482564	TGATGGCGT	1.5850	0.1658	-6.6938	Significant
FMN2	chr1 240560407-240560415	TCGCCAACA	5.0000	0.1658	-10.2581	Significant
Intergenic	chr1 243111084-243111092	ACGCCAACA	5.0000	NA	-9.3124	Significant
Intergenic	chr1 244383620-244383628	TGTTGGCGT	2.9386	NA	-18.1227	Significant
HNRNPU	chr1 245016664-245016672	TGATGGCGA	5.0000	-0.6305	-8.4534	Significant
KIF26B	chr1 245664073-245664081	TGATGGCGA	1.0000	0.1384	-8.2834	Significant
SMYD3	chr1 246245535-246245543	TGTAGGCGT	1.4150	-0.1507	-10.2089	Significant
SMYD3	chr1 246291369-246291377	TGTAGGCGT	2.5850	-0.1507	-6.8473	Significant
OR2L13	chr1 248101902-248101910	ACGCCTACA	3.0000	-0.0727	-8.2834	Significant
Intergenic	chr1 248721190-248721198	ACGCCATCA	-0.4594	NA	-7.2348	Significant
Intergenic	chr2 32604-32612	TCGCCTTCA	-1.2224	NA	-6.8477	Significant
SNTG2	chr2 1121119-1121126	TCGCCTTCA	0.5475	-0.1053	-11.1907	Significant
PXDN	chr2 1702563-1702571	ACGCCAACA	0.4594	-0.1723	-5.9672	Significant
MYT1L	chr2 1881362-1881370	ACGCCATCA	5.0000	-0.0503	-10.4803	Significant
MYT1L	chr2 1909905-1909913	TCGCCTACA	2.3219	-0.0503	-7.6316	Significant
Intergenic	chr2 2447516-2447523	ACGCCATCA	-2.0780	NA	-6.3198	Significant
Intergenic	chr2 2593074-2593081	TGATGGCGT	5.0000	NA	-6.1002	Significant
Intergenic	chr2 4117393-4117401	TCGCCTTCA	3.1699	NA	-7.4724	Significant
Intergenic	chr2 4761020-4761028	TCGCCATCA	0.1375	NA	-8.2836	Significant
Intergenic	chr2 6729531-6729539	TCGCCATCA	5.0000	NA	-7.0823	Significant
Intergenic	chr2 6918486-6918494	TGAAGGCGA	2.3219	NA	-11.6236	Significant
Intergenic	chr2 11036424-11036432	ACGCCAACA	3.1699	NA	-6.7014	Significant
Intergenic	chr2 11502716-11502724	TCGCCTTCA	1.5850	NA	-16.7036	Significant
Intergenic	chr2 12223744-12223752	ACGCCAACA	5.0000	NA	-13.6405	Significant
Intergenic	chr2 12545920-12545928	TGTTGGCGA	-0.6781	NA	-6.1002	Significant
Intergenic	chr2 12768746-12768754	TCGCCTTCA	3.1699	NA	-7.4699	Significant
Intergenic	chr2 13910792-13910800	ACGCCTACA	-0.1375	NA	-5.2534	Significant
NBAS	chr2 15447409-15447417	TGATGGCGA	2.8074	-0.7008	-9.1342	Significant
Intergenic	chr2 16696561-16696569	ACGCCATCA	5.0000	NA	-11.1427	Significant
Intergenic	chr2 18271992-18272000	TGTAGGCGA	5.0000	NA	-11.1427	Significant

Intergenic	chr2 18359952-18359960	TCGCCTTCA	0.6781	NA	-4.5891	Significant
Intergenic	chr2 18611652-18611660	TGATGGCGT	1.5850	NA	-4.9165	Significant
Intergenic	chr2 18612007-18612015	TGTTGGCGT	2.8074	NA	-5.9677	Significant
Intergenic	chr2 20275837-20275845	ACGCCATCA	1.0000	NA	-6.4691	Significant
Intergenic	chr2 23068930-23068938	TGTTGGCGT	2.2224	NA	-5.3910	Significant
Intergenic	chr2 23547781-23547789	TGATGGCGT	-0.1155	NA	-5.3906	Significant
KLHL29	chr2 23699018-23699026	TGTAGGCGT	5.0000	-4.2540	-5.7924	Significant
KLHL29	chr2 23868295-23868303	TGATGGCGT	1.1155	-4.2540	-9.8058	Significant
Intergenic	chr2 24677370-24677378	TGTAGGCGA	4.2479	NA	-18.4692	Significant
NCOA1	chr2 24856375-24856383	TGAAGGCGA	1.1375	-0.6197	-6.8473	Significant
ASXL2	chr2 26001307-26001315	ACGCCAACA	1.4150	-3.6145	-8.2836	Significant
ASXL2	chr2 26087113-26087121	ACGCCTTCA	5.0000	-3.6145	-6.5209	Significant
Intergenic	chr2 27493959-27493967	TGAAGGCGT	2.8074	NA	-7.6320	Significant
GTF3C2	chr2 27564339-27564347	TCGCCTACA	2.7004	-1.4356	-6.8473	Significant
EIF2B4	chr2 27590377-27590385	TGATGGCGT	2.5850	-1.1223	-4.2710	Marginal
BRE	chr2 28115419-28115427	TGTTGGCGA	1.8074	0.0527	-5.9549	Significant
BRE	chr2 28328498-28328506	TGTTGGCGA	5.0000	0.0527	-11.4244	Significant
BRE	chr2 28359576-28359584	TGTTGGCGT	5.0000	0.0527	-8.0893	Significant
CLIP4	chr2 29366438-29366446	TGTAGGCGT	0.5850	0.3247	-7.0774	Significant
ALK	chr2 29924536-29924544	TGAAGGCGT	2.7004	-0.1108	-7.8722	Significant
Intergenic	chr2 31542834-31542842	ACGCCTACA	3.4594	NA	-4.2708	Marginal
Intergenic	chr2 31871329-31871337	TCGCCTTCA	5.0000	NA	-11.9103	Significant
MEMO1	chr2 32213424-32213432	TCGCCAACA	1.7370	-0.1986	-5.7407	Significant
SPAST	chr2 32298287-32298295	TGATGGCGT	-0.0995	-2.3642	-4.9165	Significant
SPAST	chr2 32314613-32314621	ACGCCTTCA	5.0000	-2.3642	-8.9289	Significant
Intergenic	chr2 32571065-32571073	TCGCCATCA	3.8074	NA	-5.3906	Significant
Intergenic	chr2 34484896-34484904	ACGCCTTCA	1.1699	NA	-5.2533	Significant
Intergenic	chr2 36262806-36262814	TCGCCATCA	0.6521	NA	-5.7407	Significant
CEBPZ	chr2 37458323-37458331	TCGCCTACA	0.5146	-1.9741	-6.8477	Significant
Intergenic	chr2 37682071-37682079	TGTTGGCGT	0.4594	NA	-5.5996	Significant
CDKL4	chr2 39406329-39406337	TGTTGGCGT	1.8074	0.2782	-8.9291	Significant
Intergenic	chr2 41607165-41607173	TCGCCTTCA	0.8480	NA	-15.0219	Significant
Intergenic	chr2 42190831-42190839	ACGCCTACA	3.7004	NA	-6.8473	Significant
Intergenic	chr2 42205259-42205267	TCGCCAACA	-1.2801	NA	-5.7411	Significant
MTA3	chr2 42935500-42935508	TGAAGGCGT	3.7004	-1.3294	-7.8720	Significant
THADA	chr2 43543700-43543708	TGAAGGCGA	5.0000	-1.0702	-13.1671	Significant
Intergenic	chr2 45363290-45363298	TCGCCAACA	1.5025	NA	-10.7200	Significant
PRKCE	chr2 46062213-46062221	ACGCCTACA	5.0000	-1.3397	-13.4241	Significant
PRKCE	chr2 46088804-46088812	TGTTGGCGA	5.0000	-1.3397	-8.9289	Significant
Intergenic	chr2 47501067-47501075	TCGCCATCA	-0.6781	NA	-6.6938	Significant
Intergenic	chr2 47588567-47588575	TCGCCATCA	3.4594	NA	-15.8069	Significant
Intergenic	chr2 47825428-47825436	TCGCCAACA	5.0000	NA	-10.9476	Significant
Intergenic	chr2 47914277-47914285	ACGCCATCA	2.1699	NA	-4.7182	Significant
Intergenic	chr2 48279665-48279673	TGTAGGCGA	2.4594	NA	-5.0497	Significant
Intergenic	chr2 48490088-48490096	TCGCCTTCA	5.0000	NA	-9.7560	Significant
FSHR	chr2 49258101-49258109	ACGCCATCA	5.0000	0.2854	-11.9103	Significant
Intergenic	chr2 49750744-49750752	TGTTGGCGA	3.3219	NA	-8.0891	Significant
NRXN1	chr2 50570518-50570526	TGTTGGCGT	3.5850	0.1895	-12.4057	Significant
NRXN1	chr2 50821577-50821585	TGTAGGCGT	1.8745	0.1895	-16.9461	Significant
Intergenic	chr2 51495518-51495526	TGAAGGCGA	5.0000	NA	-7.2866	Significant
Intergenic	chr2 53212801-53212809	TCGCCAACA	3.5850	NA	-13.1671	Significant
Intergenic	chr2 53518324-53518332	TGAAGGCGA	1.0000	NA	-6.3198	Significant
Intergenic	chr2 54327079-54327087	TCGCCAACA	2.3219	NA	-7.4727	Significant
SPTBN1	chr2 54731700-54731708	TGTAGGCGT	0.2895	-0.4659	-8.6980	Significant
SPTBN1	chr2 54813966-54813974	TGTTGGCGA	0.3626	-0.4659	-7.2348	Significant
EML6	chr2 54958056-54958064	ACGCCAACA	5.0000	0.1912	-9.7564	Significant
EML6	chr2 55077837-55077845	TGATGGCGA	2.1155	0.1912	-8.9289	Significant
Intergenic	chr2 55410489-55410497	ACGCCAACA	-0.7004	NA	-6.6938	Significant
VRK2	chr2 58302671-58302679	TCGCCAACA	5.0000	-1.1709	-6.3198	Significant
VRK2	chr2 58309856-58309864	TGAAGGCGA	2.3219	-1.1709	-6.3196	Significant
Intergenic	chr2 60577567-60577575	ACGCCAACA	0.4594	NA	-6.4691	Significant
BCL11A	chr2 60734116-60734124	TGTTGGCGT	-0.1155	-2.4553	-8.4534	Significant
COMMD1	chr2 62338401-62338409	ACGCCAACA	2.2224	-0.1452	-6.6938	Significant
COMMD1	chr2 62344562-62344570	TGATGGCGT	2.5850	-0.1452	-10.0224	Significant
Intergenic	chr2 64283706-64283714	TGTTGGCGT	5.0000	NA	-5.7411	Significant

AFTPH	chr2 64772017-64772025	TCGCCATCA	5.0000	-2.4466	-7.8724	Significant
Intergenic	chr2 65162761-65162769	TGTTGGCGA	3.8074	NA	-7.2348	Significant
SPRED2	chr2 65542516-65542524	TCGCCATCA	-1.5850	-2.0671	-6.6939	Significant
Intergenic	chr2 66052318-66052326	TGATGGCGT	5.0000	NA	-4.4471	Significant
Intergenic	chr2 66399948-66399956	TGTAGGCGA	5.0000	NA	-8.2834	Significant
PROKR1	chr2 68876022-68876030	TCGCCTACA	3.1699	-0.0889	-4.2708	Marginal
ANXA4	chr2 70052756-70052764	ACGCCTACA	5.0000	0.2939	-8.2834	Significant
Intergenic	chr2 70589757-70589765	ACGCCATCA	-0.5146	NA	-6.3198	Significant
Intergenic	chr2 70849878-70849886	TCGCCTTCA	4.0000	NA	-8.2834	Significant
EXOC6B	chr2 72724618-72724626	TGAAGGCGT	5.0000	-0.1369	-4.4380	Significant
SLC4A5	chr2 74543761-74543769	ACGCCTACA	5.0000	-0.0479	-7.6831	Significant
Intergenic	chr2 75157933-75157941	ACGCCAACA	1.4150	NA	-7.8720	Significant
Intergenic	chr2 76209036-76209044	TGTAGGCGA	5.0000	NA	-10.4804	Significant
Intergenic	chr2 76313980-76313988	TGATGGCGA	5.0000	NA	-4.5890	Significant
Intergenic	chr2 76912645-76912653	ACGCCATCA	5.0000	NA	-7.2864	Significant
LRRTM4	chr2 77034166-77034174	TGTAGGCGA	3.0000	-0.0055	-12.9102	Significant
Intergenic	chr2 77807549-77807557	TCGCCTTCA	1.0000	NA	-5.9672	Significant
Intergenic	chr2 77949305-77949313	TGTAGGCGT	2.5850	NA	-7.2866	Significant
Intergenic	chr2 79256356-79256364	ACGCCTTCA	-0.2410	NA	-4.7182	Significant
CTNNA2	chr2 80591400-80591408	TGAAGGCGT	5.0000	0.1148	-7.2424	Significant
CTNNA2	chr2 80656706-80656714	TGTTGGCGT	2.7004	0.1148	-9.8074	Significant
CTNNA2	chr2 80871499-80871507	ACGCCATCA	0.5305	0.1148	-8.0382	Significant
Intergenic	chr2 82691879-82691887	TCGCCTTCA	-0.4854	NA	-9.5736	Significant
Intergenic	chr2 83186617-83186625	TCGCCAACA	5.0000	NA	-11.6709	Significant
Intergenic	chr2 83848377-83848385	TGAAGGCGA	5.0000	NA	-10.2581	Significant
Intergenic	chr2 83974560-83974568	ACGCCTTCA	0.0000	NA	-5.2533	Significant
DNAH6	chr2 84744018-84744026	TGAAGGCGT	5.0000	-0.4819	-8.0382	Significant
TCF7L1	chr2 85420315-85420323	TGATGGCGA	5.0000	-0.1341	-8.4534	Significant
Intergenic	chr2 85691105-85691113	TGTAGGCGA	3.8074	NA	-6.6938	Significant
MAT2A	chr2 85770922-85770930	TGTTGGCGT	0.1699	-1.5483	-10.7198	Significant
Intergenic	chr2 86021115-86021123	TCGCCAACA	-0.7105	NA	-13.4243	Significant
REEP1	chr2 86545205-86545213	TGAAGGCGT	5.0000	0.1876	-8.4462	Significant
ANKRD36BP2	chr2 89102401-89102409	ACGCCAACA	-1.0875	-0.8396	-4.9165	Significant
Intergenic	chr2 89228459-89228467	TGTTGGCGA	0.1699	NA	-5.3910	Significant
Intergenic	chr2 89612730-89612738	TGAAGGCGA	5.0000	NA	-6.3297	Significant
Intergenic	chr2 90241396-90241404	ACGCCTTCA	2.8074	NA	-7.8717	Significant
Intergenic	chr2 91799417-91799425	TCGCCTTCA	5.0000	NA	-4.4709	Significant
Intergenic	chr2 91823784-91823792	TCGCCAACA	2.4594	NA	-6.6938	Significant
LOC654342	chr2 91837528-91837536	TGAAGGCGA	3.5850	NA	-10.4803	Significant
Intergenic	chr2 92059982-92059990	TGTTGGCGA	1.6630	NA	-8.0378	Significant
KCNIP3	chr2 96046710-96046718	TGATGGCGA	-0.8480	-0.2305	-7.2864	Significant
CNNM4	chr2 97453331-97453339	ACGCCTACA	5.0000	-1.6589	-9.5736	Significant
Intergenic	chr2 98313756-98313764	ACGCCTTCA	5.0000	NA	-10.4806	Significant
REV1	chr2 100030822-100030830	TGAAGGCGT	5.0000	-1.7958	-7.6833	Significant
Intergenic	chr2 101067948-101067956	TCGCCTTCA	-1.0995	NA	-7.8720	Significant
RNF149	chr2 101904234-101904242	ACGCCTTCA	5.0000	-1.5614	-4.7569	Significant
MAP4K4	chr2 102333789-102333797	TGAAGGCGA	5.0000	-0.8621	-7.0772	Significant
SLC9A4	chr2 103116556-103116564	TGAAGGCGA	-0.2630	-0.1691	-8.4534	Significant
Intergenic	chr2 103541479-103541487	TCGCCTACA	5.0000	NA	-5.5996	Significant
Intergenic	chr2 103646983-103646991	TGTTGGCGA	-0.5475	NA	-9.8060	Significant
Intergenic	chr2 103970643-103970651	ACGCCATCA	2.0000	NA	-5.7407	Significant
Intergenic	chr2 104906575-104906583	TGATGGCGT	-1.1375	NA	-9.3627	Significant
LOC100287010	chr2 105021741-105021749	TCGCCATCA	5.0000	0.2158	-4.2708	Marginal
Intergenic	chr2 105456230-105456238	ACGCCATCA	5.0000	NA	-12.9102	Significant
MRPS9	chr2 105664226-105664234	TGAAGGCGA	3.5850	-0.7757	-4.2929	Marginal
TGFBRAP1	chr2 105887190-105887198	ACGCCTTCA	5.0000	-1.5277	-4.5890	Significant
Intergenic	chr2 106080554-106080562	TGATGGCGT	1.0995	NA	-8.2802	Significant
UXS1	chr2 106783261-106783269	ACGCCATCA	1.4594	-0.3904	-9.3629	Significant
Intergenic	chr2 107290087-107290095	TCGCCAACA	5.0000	NA	-11.2314	Significant
Intergenic	chr2 107620587-107620595	TGATGGCGT	0.2630	NA	-7.6316	Significant
Intergenic	chr2 107620778-107620786	ACGCCAACA	2.5850	NA	-8.9291	Significant
Intergenic	chr2 107712953-107712961	TGTTGGCGT	1.8480	NA	-6.1520	Significant
SH3RF3	chr2 110102080-110102088	ACGCCTTCA	5.0000	-0.4573	-6.3297	Significant
ACOXL	chr2 111763292-111763300	TGTTGGCGA	0.8074	0.3754	-7.8722	Significant
TMEM87B	chr2 112847271-112847279	TGAAGGCGT	0.0000	-0.3695	-6.6939	Significant

ZC3H8	chr2 112983729-112983737	ACGCCAACA	5.0000	-0.3225	-7.9758	Significant
Intergenic	chr2 113031152-113031160	ACGCCATCA	0.4594	NA	-16.3718	Significant
Intergenic	chr2 113611283-113611291	ACGCCATCA	5.0000	NA	-6.6221	Significant
Intergenic	chr2 114562923-114562931	TGATGGCGT	2.0000	NA	-23.7456	Significant
Intergenic	chr2 114644738-114644746	TGAAGGCGT	0.4150	NA	-6.1002	Significant
Intergenic	chr2 114646120-114646128	TCGCCTTCA	5.0000	NA	-5.9550	Significant
Intergenic	chr2 114835977-114835985	TCGCCATCA	2.7004	NA	-4.2708	Marginal
Intergenic	chr2 114925345-114925353	TGATGGCGT	2.5850	NA	-4.2708	Marginal
Intergenic	chr2 115167482-115167490	TGATGGCGT	-0.4475	NA	-5.0497	Significant
DPP10	chr2 115300180-115300188	TCGCCTTCA	1.2479	0.0618	-6.1520	Significant
Intergenic	chr2 117598587-117598595	TCGCCTACA	3.1699	NA	-17.8714	Significant
Intergenic	chr2 119786945-119786953	TGATGGCGA	5.0000	NA	-20.3187	Significant
SCTR	chr2 120268641-120268649	TGATGGCGA	5.0000	0.1368	-17.8714	Significant
CLASP1	chr2 122100701-122100709	TCGCCATCA	2.8074	-1.9902	-5.9014	Significant
Intergenic	chr2 124103848-124103856	TGTAGGCGT	3.8074	NA	-11.4243	Significant
CNTNAP5	chr2 125158881-125158889	ACGCCTTCA	3.9696	0.0450	-8.7041	Significant
Intergenic	chr2 125837011-125837019	TGTTGGCGT	1.7370	NA	-6.3196	Significant
Intergenic	chr2 125932189-125932197	TGTTGGCGA	3.9069	NA	-7.4705	Significant
Intergenic	chr2 126318923-126318931	ACGCCATCA	-0.6521	NA	-4.2710	Marginal
Intergenic	chr2 126737200-126737208	TGAAGGCGT	0.7370	NA	-8.7043	Significant
Intergenic	chr2 126805855-126805863	ACGCCATCA	5.0000	NA	-5.3906	Significant
Intergenic	chr2 128696291-128696299	TGTTGGCGA	5.0000	NA	-8.4534	Significant
Intergenic	chr2 131062423-131062431	TGTTGGCGA	5.0000	NA	-11.6709	Significant
Intergenic	chr2 131534366-131534374	ACGCCAACA	2.7004	NA	-6.6938	Significant
ARHGEF4	chr2 131753035-131753043	ACGCCATCA	2.5850	-0.5880	-5.1011	Significant
Intergenic	chr2 132893601-132893609	TCGCCATCA	5.0000	NA	-8.7043	Significant
Intergenic	chr2 133142897-133142905	TGTTGGCGT	-0.4854	NA	-6.6939	Significant
GPR39	chr2 133260117-133260125	TGTAGGCGA	5.0000	-3.4394	-17.5297	Significant
Intergenic	chr2 134824549-134824557	ACGCCATCA	1.3219	NA	-7.0825	Significant
Intergenic	chr2 135801114-135801122	TCGCCATCA	3.3219	NA	-4.3969	Significant
Intergenic	chr2 137562548-137562556	TGATGGCGA	2.3219	NA	-5.9549	Significant
Intergenic	chr2 140642005-140642013	TGTAGGCGA	5.0000	NA	-12.1138	Significant
LRP1B	chr2 142846131-142846139	TCGCCTACA	2.5850	0.1885	-9.3124	Significant
Intergenic	chr2 142914557-142914565	TGTTGGCGA	3.3219	NA	-7.0772	Significant
Intergenic	chr2 144582666-144582674	TCGCCATCA	3.4594	NA	-18.9864	Significant
GTDC1	chr2 144906389-144906397	ACGCCAACA	5.0000	-1.2233	-12.6591	Significant
GTDC1	chr2 144979269-144979277	TGAAGGCGT	2.5850	-1.2233	-12.6593	Significant
Intergenic	chr2 145331134-145331142	TCGCCTACA	2.0000	NA	-5.9672	Significant
Intergenic	chr2 147706771-147706779	TCGCCATCA	5.0000	NA	-7.8720	Significant
Intergenic	chr2 148460826-148460834	ACGCCTTCA	3.5850	NA	-10.6711	Significant
Intergenic	chr2 149353484-149353492	ACGCCAACA	2.1699	NA	-7.6831	Significant
EPC2	chr2 149444279-149444287	TCGCCATCA	5.0000	-4.6151	-7.2424	Significant
Intergenic	chr2 150556918-150556926	TGAAGGCGT	5.0000	NA	-4.3961	Significant
Intergenic	chr2 150620050-150620058	TGTTGGCGT	5.0000	NA	-5.7673	Significant
Intergenic	chr2 150828650-150828658	TCGCCAACA	5.0000	NA	-14.1681	Significant
Intergenic	chr2 150984622-150984630	TCGCCTTCA	2.0000	NA	-7.2352	Significant
NEB	chr2 152385738-152385746	TGTAGGCGT	5.0000	0.0248	-9.1342	Significant
NEB	chr2 152487565-152487573	ACGCCTTCA	3.3219	0.0248	-6.8477	Significant
NEB	chr2 152517244-152517252	ACGCCAACA	5.0000	0.0248	-11.9104	Significant
CACNB4	chr2 152813270-152813278	TGTAGGCGA	2.0000	0.1475	-7.0772	Significant
Intergenic	chr2 155443380-155443388	TGAAGGCGA	5.0000	NA	-9.1344	Significant
Intergenic	chr2 156393032-156393040	TCGCCATCA	1.3219	NA	-9.3128	Significant
Intergenic	chr2 156404586-156404594	TGTTGGCGA	1.4594	NA	-12.4056	Significant
Intergenic	chr2 156859980-156859988	TCGCCTTCA	-1.3949	NA	-7.2424	Significant
GPD2	chr2 157342027-157342035	TGTAGGCGA	3.4594	-0.5190	-7.0772	Significant
Intergenic	chr2 157882691-157882699	TGTTGGCGA	5.0000	NA	-6.6939	Significant
Intergenic	chr2 158078986-158078994	ACGCCATCA	1.8745	NA	-9.5736	Significant
UPP2	chr2 158971761-158971769	TCGCCATCA	5.0000	0.0177	-5.3906	Significant
Intergenic	chr2 159572865-159572873	TCGCCTTCA	1.0995	NA	-8.8786	Significant
TANC1	chr2 159896809-159896817	TGTTGGCGT	5.0000	-4.6421	-7.6316	Significant
LY75	chr2 160694573-160694581	ACGCCAACA	5.0000	-0.0342	-7.8720	Significant
Intergenic	chr2 160770904-160770912	TGAAGGCGT	5.0000	NA	-4.3965	Significant
ITGB6	chr2 160997559-160997567	TGTTGGCGT	2.0000	-0.1156	-7.2348	Significant
Intergenic	chr2 161644912-161644920	ACGCCAACA	0.1155	NA	-11.1427	Significant
Intergenic	chr2 162102946-162102954	ACGCCATCA	1.7370	NA	-5.2699	Significant

PSMD14	chr2 162185552-162185560	TGAAGGCGA	5.0000	-1.3464	-13.4241	Significant
SLC4A10	chr2 162638040-162638048	TGTTGGCGA	0.0000	0.1327	-8.0893	Significant
SLC4A10	chr2 162696394-162696402	TGTTGGCGT	2.1155	0.1327	-7.6320	Significant
KCNH7	chr2 163641230-163641238	TCGCCTACA	1.2630	0.0721	-5.2533	Significant
Intergenic	chr2 163770412-163770420	TGTTGGCGA	-0.9569	NA	-6.6939	Significant
Intergenic	chr2 165074027-165074035	TCGCCTACA	-0.1375	NA	-9.0010	Significant
TTC21B	chr2 166788276-166788284	TGAAGGCGA	-3.0255	-0.5756	-10.0066	Significant
SCN1A	chr2 166987856-166987864	TGAAGGCGT	2.3219	0.1678	-6.7014	Significant
Intergenic	chr2 168239965-168239973	ACGCCTACA	1.8074	NA	-5.5116	Significant
Intergenic	chr2 168604304-168604312	TGAAGGCGT	5.0000	NA	-5.2533	Significant
STK39	chr2 168892365-168892373	ACGCCATCA	-0.1375	-0.7983	-11.0946	Significant
Intergenic	chr2 169225362-169225370	TGTTGGCGA	3.3219	NA	-11.1427	Significant
ABCB11	chr2 169800628-169800636	ACGCCTTCA	-0.1255	0.2412	-5.5996	Significant
ABCB11	chr2 169867505-169867513	TCGCCTTCA	1.5850	0.2412	-7.8720	Significant
Intergenic	chr2 169966072-169966080	TCGCCATCA	5.0000	NA	-14.2114	Significant
UBR3	chr2 170927590-170927598	TGTTGGCGA	-0.7162	-1.0173	-13.6405	Significant
METAP1D	chr2 172885038-172885046	ACGCCATCA	5.0000	-1.1658	-10.9476	Significant
Intergenic	chr2 174420398-174420406	TCGCCTTCA	-0.3219	NA	-5.0501	Significant
OLA1	chr2 175039413-175039421	TCGCCTTCA	1.8074	0.1525	-9.7560	Significant
GPR155	chr2 175337865-175337873	TGAAGGCGA	5.0000	0.2282	-5.0497	Significant
CHN1	chr2 175751860-175751868	TGATGGCGA	2.0000	-0.0595	-14.4800	Significant
KIAA1715	chr2 176821518-176821526	TCGCCTACA	5.0000	-0.7291	-10.9478	Significant
Intergenic	chr2 177383592-177383600	TCGCCATCA	2.2224	NA	-7.2866	Significant
Intergenic	chr2 177632501-177632509	TGTTGGCGA	1.8074	NA	-6.1520	Significant
Promoter_TTC30A	chr2 178484236-178484244	TGTTGGCGT	0.8745	-0.1328	-8.7041	Significant
TTN	chr2 179468695-179468703	ACGCCTTCA	-1.6699	0.2578	-9.5615	Significant
Intergenic	chr2 180187699-180187707	ACGCCAACA	1.5850	NA	-6.6939	Significant
ZNF385B	chr2 180602862-180602870	ACGCCTTCA	2.1699	-0.0511	-8.0378	Significant
CWC22	chr2 180830487-180830495	ACGCCATCA	2.3219	-2.2796	-6.5209	Significant
Intergenic	chr2 181019508-181019516	TGTTGGCGA	1.3219	NA	-5.3906	Significant
Intergenic	chr2 181152523-181152531	TCGCCAACA	3.0000	NA	-7.4699	Significant
Intergenic	chr2 181371785-181371793	TGTAGGCGA	3.5850	NA	-11.4243	Significant
UBE2E3	chr2 181902820-181902828	ACGCCTTCA	-2.2630	-1.0121	-8.5043	Significant
NEUROD1	chr2 182543665-182543673	ACGCCTTCA	3.5850	0.2377	-6.3196	Significant
Intergenic	chr2 182643633-182643641	TGATGGCGT	-0.4594	NA	-4.7693	Significant
PDE1A	chr2 183200184-183200192	ACGCCAACA	1.4150	0.0514	-7.0823	Significant
DNAJC10	chr2 183609693-183609701	TCGCCTTCA	5.0000	0.1803	-8.0382	Significant
NCKAP1	chr2 183830037-183830045	ACGCCATCA	5.0000	-0.5126	-5.2533	Significant
Intergenic	chr2 185131213-185131221	ACGCCTTCA	-0.5025	NA	-7.6316	Significant
Intergenic	chr2 185191002-185191010	TGTTGGCGA	5.0000	NA	-8.0382	Significant
Intergenic	chr2 186798771-186798779	ACGCCTTCA	5.0000	NA	-17.8714	Significant
Intergenic	chr2 187943479-187943487	TGTAGGCGT	2.4594	NA	-7.0772	Significant
Intergenic	chr2 189521259-189521267	TGTTGGCGA	1.4594	NA	-9.1342	Significant
Promoter_C2orf88	chr2 191002319-191002327	TCGCCATCA	5.0000	0.2134	-7.4727	Significant
Intergenic	chr2 191467123-191467131	ACGCCTTCA	-0.2630	NA	-9.5613	Significant
Intergenic	chr2 191629744-191629752	TGATGGCGA	5.0000	NA	-8.2802	Significant
Intergenic	chr2 192055088-192055096	TGTAGGCGT	0.6521	NA	-9.8058	Significant
MYO1B	chr2 192255131-192255139	ACGCCTTCA	3.4594	-1.2965	-6.3196	Significant
MYO1B	chr2 192278382-192278390	TGAAGGCGT	5.0000	-1.2965	-16.9461	Significant
Intergenic	chr2 192718611-192718619	TGAAGGCGA	5.0000	NA	-8.0891	Significant
Intergenic	chr2 192744449-192744457	TGAAGGCGT	5.0000	NA	-7.0774	Significant
Intergenic	chr2 193719900-193719908	TGTTGGCGT	5.0000	NA	-9.3124	Significant
HECW2	chr2 197177362-197177370	TGATGGCGT	-0.7655	0.1757	-5.3910	Significant
HECW2	chr2 197423355-197423363	TGTTGGCGT	5.0000	0.1757	-5.4421	Significant
GTF3C3	chr2 197645350-197645358	ACGCCATCA	5.0000	-0.4827	-10.2089	Significant
ANKRD44	chr2 198055075-198055083	TCGCCTTCA	3.4594	-0.2954	-4.9168	Significant
ANKRD44	chr2 198131940-198131948	TCGCCATCA	5.0000	-0.2954	-15.8069	Significant
COQ10B	chr2 198319366-198319374	ACGCCATCA	3.3219	-0.9041	-5.3914	Significant
MOB4	chr2 198399809-198399817	TGATGGCGT	3.9069	-2.7783	-9.5615	Significant
SATB2	chr2 200247476-200247484	TGTAGGCGA	-0.1699	-4.4036	-8.2834	Significant
Intergenic	chr2 200443770-200443778	TGTTGGCGA	1.4150	NA	-6.3196	Significant
Intergenic	chr2 201063518-201063526	TGATGGCGT	2.3219	NA	-5.9549	Significant
SPATS2L	chr2 201182064-201182072	ACGCCTACA	0.1520	-0.4662	-5.7924	Significant
ALS2CR11	chr2 202469242-202469250	ACGCCATCA	-0.9069	0.1809	-9.8058	Significant
MPP4	chr2 202559961-202559969	ACGCCTACA	1.2630	0.0418	-10.6715	Significant

Intergenic	chr2 202774767-202774775	TCGCCAACA	-0.2224	NA	-8.7041	Significant
Intergenic	chr2 203804037-203804045	TGATGGCGA	2.8074	NA	-10.7198	Significant
NBEAL1	chr2 204022254-204022262	TCGCCATCA	1.9069	-0.6680	-9.3124	Significant
ABI2	chr2 204205932-204205940	ACGCCTACA	2.0000	-0.7274	-6.6938	Significant
Intergenic	chr2 205384344-205384352	ACGCCATCA	-0.2630	NA	-9.5736	Significant
PARD3B	chr2 205546240-205546248	TGAAGGCGT	0.2630	0.0912	-6.8473	Significant
PARD3B	chr2 206177838-206177846	TGATGGCGA	1.3219	0.0912	-7.8720	Significant
Promoter_NRP2	chr2 206546412-206546420	ACGCCTTCA	1.7370	0.1781	-7.0825	Significant
Intergenic	chr2 207590096-207590104	TCGCCAACA	2.4594	NA	-5.9549	Significant
PLEKHM3	chr2 208843497-208843505	TCGCCTACA	5.0000	-2.2509	-7.6833	Significant
PTH2R	chr2 209339102-209339110	ACGCCATCA	0.0000	0.1847	-5.2534	Significant
MYL1	chr2 211164163-211164171	ACGCCATCA	3.7004	0.0753	-9.5736	Significant
MYL1	chr2 211164171-211164171	ACGCCATCA	3.4594	0.0753	-8.0378	Significant
Intergenic	chr2 213771442-213771450	TGAAGGCGA	5.0000	NA	-4.7693	Significant
Intergenic	chr2 216668712-216668720	TCGCCTTCA	5.0000	NA	-11.5686	Significant
XRCC5	chr2 217023073-217023081	ACGCCATCA	3.3219	-0.0739	-11.1427	Significant
Intergenic	chr2 219778641-219778649	TGATGGCGT	-0.3219	NA	-5.4423	Significant
Intergenic	chr2 222584657-222584665	TGAAGGCGA	1.0000	NA	-6.3196	Significant
Intergenic	chr2 224040997-224041005	TGTTGGCGT	5.0000	NA	-12.1138	Significant
Intergenic	chr2 224538455-224538463	TCGCCAACA	1.8074	NA	-10.0223	Significant
Intergenic	chr2 225503476-225503484	TGTAGGCGT	-0.3785	NA	-7.2348	Significant
DOCK10	chr2 225779561-225779569	ACGCCTACA	5.0000	0.0402	-6.3198	Significant
DOCK10	chr2 225830837-225830845	ACGCCTTCA	0.2895	0.0402	-6.4695	Significant
Intergenic	chr2 226881073-226881081	TCGCCTACA	5.0000	NA	-6.6938	Significant
Intergenic	chr2 227187931-227187939	ACGCCAACA	5.0000	NA	-14.2112	Significant
LOC654841	chr2 228134119-228134127	TCGCCAACA	3.3219	0.0334	-9.5736	Significant
COL4A3	chr2 228134119-228134127	TCGCCAACA	5.0000	0.1300	-12.4985	Significant
Intergenic	chr2 228255453-228255461	TGAAGGCGT	5.0000	NA	-10.4803	Significant
Intergenic	chr2 228788877-228788885	TGATGGCGA	-1.8260	NA	-6.8992	Significant
PID1	chr2 230088901-230088909	TGTTGGCGA	-0.1155	-0.0020	-5.0505	Significant
SP140	chr2 231152135-231152143	ACGCCTTCA	3.3219	-0.4065	-11.9103	Significant
SP100	chr2 231378588-231378596	TCGCCATCA	3.0000	-0.8280	-8.0891	Significant
Intergenic	chr2 231886408-231886416	TCGCCATCA	1.7004	NA	-9.3124	Significant
Intergenic	chr2 232762072-232762080	TCGCCAACA	0.3626	NA	-11.1909	Significant
DIS3L2	chr2 232841005-232841013	TGATGGCGT	5.0000	-0.3062	-4.5681	Significant
DIS3L2	chr2 233122884-233122892	ACGCCTTCA	1.0000	-0.3062	-9.5736	Significant
EIF4E2	chr2 233433787-233433795	TGTTGGCGT	-0.1375	-0.3819	-6.8473	Significant
GIGYF2	chr2 233676981-233676989	TGAAGGCGT	5.0000	-4.8521	-6.6938	Significant
NGEF	chr2 233826056-233826064	TCGCCTTCA	5.0000	-1.5072	-8.8782	Significant
USP40	chr2 234400842-234400850	TCGCCTTCA	-0.5146	-0.5983	-9.7560	Significant
AGAP1	chr2 236850517-236850525	TGAAGGCGA	0.5850	-1.3564	-9.3124	Significant
ASB18	chr2 237135304-237135312	TGTTGGCGT	-0.6374	0.5797	-7.4701	Significant
LRRFIP1	chr2 238635330-238635338	TGATGGCGT	0.7370	-0.4946	-14.1685	Significant
Intergenic	chr2 239627665-239627673	ACGCCAACA	1.8745	NA	-8.4534	Significant
ANKMY1	chr2 241472269-241472277	ACGCCATCA	1.9069	-0.8226	-9.3124	Significant
Intergenic	chr3 536475-536483	ACGCCTTCA	1.7370	NA	-8.0382	Significant
Intergenic	chr3 961691-961699	ACGCCATCA	0.4150	NA	-12.6591	Significant
Intergenic	chr3 1051921-1051929	TGATGGCGT	0.3219	NA	-9.3627	Significant
Intergenic	chr3 4260453-4260461	ACGCCTTCA	1.8074	NA	-7.2864	Significant
Intergenic	chr3 5812480-5812488	TGAAGGCGA	5.0000	NA	-11.7050	Significant
GRM7	chr3 7048806-7048814	TGATGGCGT	0.4594	0.2026	-6.6938	Significant
RAD18	chr3 8972359-8972367	ACGCCTTCA	1.7370	-2.7185	-8.0382	Significant
ATG7	chr3 11589979-11589987	TCGCCTTCA	5.0000	-0.8654	-7.4699	Significant
PPARG	chr3 12432946-12432954	TGAAGGCGA	2.2224	-1.7583	-4.2931	Marginal
Intergenic	chr3 12595240-12595248	TGATGGCGT	5.0000	NA	-4.7186	Significant
Intergenic	chr3 13469710-13469718	TCGCCAACA	-1.2479	NA	-7.6316	Significant
Intergenic	chr3 14697062-14697070	TGTAGGCGT	5.0000	NA	-8.7041	Significant
NR2C2	chr3 15043821-15043829	TCGCCATCA	3.3219	-2.2621	-8.6982	Significant
ANKRD28	chr3 15860647-15860655	TGATGGCGT	5.0000	-1.7231	-6.1520	Significant
PLCL2	chr3 17082883-17082891	TGAAGGCGT	1.7370	-1.9141	-8.0382	Significant
Intergenic	chr3 18779299-18779307	TCGCCAACA	3.1699	NA	-10.4803	Significant
EFHB	chr3 19945746-19945754	TGATGGCGT	1.7370	0.0885	-8.0382	Significant
Intergenic	chr3 20795759-20795767	TGTTGGCGA	5.0000	NA	-7.0828	Significant
Intergenic	chr3 21005512-21005520	ACGCCTTCA	1.4854	NA	-5.7411	Significant
Intergenic	chr3 22806460-22806468	TGATGGCGA	3.5850	NA	-7.8720	Significant

UBE2E2	chr3 23567133-23567141	TGTAGGCGT	5.0000	-0.0287	-11.9103	Significant
THRB	chr3 24272740-24272748	TGAAGGCGA	1.5850	-1.0776	-5.5994	Significant
RARB	chr3 25323137-25323145	TGTAGGCGT	5.0000	0.0536	-17.5656	Significant
Intergenic	chr3 26205492-26205500	TGAAGGCGA	3.4594	NA	-6.6939	Significant
LRRC3B	chr3 26744989-26744996	TGAAGGCGT	5.0000	0.0247	-10.4609	Significant
Intergenic	chr3 28093241-28093249	TCGCCAACA	0.7370	NA	-7.4727	Significant
TGFBR2	chr3 30712132-30712139	ACGCCAACA	3.0000	-3.3323	-4.5890	Significant
GADL1	chr3 30935417-30935425	ACGCCAACA	5.0000	0.0338	-9.5737	Significant
Intergenic	chr3 31527178-31527186	TGTAGGCGA	5.0000	NA	-9.1342	Significant
OSBPL10	chr3 32001017-32001025	TCGCCATCA	5.0000	-2.0256	-9.8058	Significant
CLASP2	chr3 33580881-33580889	TGATGGCGT	5.0000	-2.2580	-13.1223	Significant
CLASP2	chr3 33708975-33708983	TGATGGCGT	5.0000	-2.2580	-6.6938	Significant
Intergenic	chr3 34288185-34288193	TCGCCATCA	3.9069	NA	-8.7043	Significant
Intergenic	chr3 34463574-34463582	TCGCCAACA	0.2630	NA	-10.2089	Significant
ARPP21	chr3 35742957-35742965	TCGCCAACA	5.0000	0.0011	-6.8990	Significant
Intergenic	chr3 36204423-36204431	ACGCCTTCA	0.3219	NA	-7.8722	Significant
ITGA9	chr3 37805321-37805329	TCGCCTTCA	1.7370	0.3043	-8.0382	Significant
Intergenic	chr3 39672343-39672351	TGATGGCGA	3.4594	NA	-6.3300	Significant
Intergenic	chr3 39772910-39772918	TCGCCAACA	5.0000	NA	-4.7182	Significant
MYRIP	chr3 40048916-40048924	ACGCCAACA	2.8074	-2.3776	-5.3906	Significant
MYRIP	chr3 40187696-40187704	TGTTGGCGT	1.3219	-2.3776	-6.4691	Significant
Intergenic	chr3 40422777-40422785	ACGCCATCA	-0.0995	NA	-14.1681	Significant
ENTPD3	chr3 40443090-40443098	TGATGGCGA	-1.7776	0.2002	-9.1342	Significant
ENTPD3-AS1	chr3 40443090-40443098	TGATGGCGA	1.0000	0.0642	-5.9677	Significant
Intergenic	chr3 40762008-40762016	TCGCCAACA	3.8074	NA	-10.9476	Significant
Intergenic	chr3 42104360-42104368	TGAAGGCGT	5.0000	NA	-8.9293	Significant
Intergenic	chr3 43004383-43004391	ACGCCAACA	1.7370	NA	-8.0382	Significant
ANO10	chr3 43526032-43526040	TCGCCTTCA	0.5146	0.1486	-10.4609	Significant
Intergenic	chr3 43874490-43874498	TGTAGGCGT	2.5850	NA	-9.7560	Significant
Intergenic	chr3 44076705-44076713	TCGCCAACA	5.0000	NA	-6.6938	Significant
Intergenic	chr3 44705248-44705256	TGATGGCGA	5.0000	NA	-7.4699	Significant
Promoter_CCR9	chr3 45927096-45927104	TGTTGGCGA	1.1699	0.0144	-8.2834	Significant
LZTFL1	chr3 45927096-45927104	TGTTGGCGA	5.0000	-0.1772	-10.4804	Significant
CCR5	chr3 46412332-46412340	TCGCCTTCA	5.0000	-0.4432	-17.8716	Significant
LRRC2	chr3 46563062-46563070	TGTTGGCGA	1.1375	0.1731	-6.8473	Significant
SETD2	chr3 47156177-47156185	TCGCCAACA	0.2410	-2.3356	-6.1520	Significant
KLHL18	chr3 47349153-47349161	TGTAGGCGT	1.4594	-3.1265	-7.6324	Significant
SMARCC1	chr3 47634014-47634022	TGTAGGCGA	5.0000	-0.1867	-12.3714	Significant
CDC25A	chr3 48202718-48202726	TGATGGCGA	0.7370	-2.5708	-15.3693	Significant
DOCK3	chr3 50989259-50989267	TGATGGCGT	1.7370	0.1736	-8.0382	Significant
SFMBT1	chr3 52991187-52991195	ACGCCAACA	2.8074	-3.1727	-7.6320	Significant
IL17RB	chr3 53894345-53894353	TGTTGGCGT	5.0000	1.1072	-8.0378	Significant
Intergenic	chr3 53948274-53948282	TGAAGGCGT	5.0000	NA	-5.4421	Significant
CACNA2D3	chr3 54415783-54415791	TCGCCTTCA	5.0000	0.7616	-10.0064	Significant
CACNA2D3	chr3 55019499-55019507	ACGCCATCA	1.2224	0.7616	-5.9549	Significant
Intergenic	chr3 55286391-55286399	TGAAGGCGA	1.7370	NA	-8.0382	Significant
FAM208A	chr3 56663455-56663463	ACGCCTTCA	5.0000	-2.6541	-6.3198	Significant
Intergenic	chr3 57953540-57953548	TGTTGGCGT	5.0000	NA	-5.0505	Significant
Intergenic	chr3 58437951-58437959	ACGCCTTCA	5.0000	NA	-10.3119	Significant
Intergenic	chr3 59729944-59729952	TGTAGGCGA	3.9069	NA	-6.5209	Significant
FHIT	chr3 60859958-60859966	ACGCCAACA	3.5850	0.3380	-9.3627	Significant
Intergenic	chr3 61431302-61431310	TGTAGGCGT	1.7370	NA	-8.0382	Significant
PTPRG	chr3 61691208-61691216	TCGCCATCA	5.0000	-0.3126	-6.5209	Significant
PTPRG	chr3 61766562-61766570	TGATGGCGT	0.8480	-0.3126	-7.0772	Significant
PTPRG	chr3 61843431-61843439	TGTTGGCGT	5.0000	-0.3126	-8.0378	Significant
PTPRG	chr3 62221928-62221936	ACGCCTACA	0.6781	-0.3126	-6.4869	Significant
PTPRG	chr3 62242630-62242638	TCGCCTACA	5.0000	-0.3126	-6.5209	Significant
CADPS	chr3 62402261-62402269	ACGCCTTCA	5.0000	-0.1335	-6.6938	Significant
CADPS	chr3 62850164-62850171	TGTAGGCGT	5.0000	-0.1335	-10.0223	Significant
SYNPR	chr3 63355368-63355376	TGAAGGCGA	0.5146	-0.0275	-5.7928	Significant
SYNPR	chr3 63506384-63506392	TCGCCATCA	3.1699	-0.0275	-9.3124	Significant
SNTN	chr3 63645913-63645921	TGATGGCGT	2.5850	0.2956	-5.9550	Significant
Intergenic	chr3 63752800-63752808	TGTTGGCGT	-0.3219	NA	-6.8473	Significant
PSMD6	chr3 64003673-64003680	TGAAGGCGA	5.0000	-0.5593	-15.2517	Significant
Intergenic	chr3 66040617-66040625	TCGCCAACA	3.7004	NA	-5.9675	Significant

Intergenic	chr3 66567012-66567020	ACGCCAACA	-1.9475	NA	-4.4471	Significant
Intergenic	chr3 67036518-67036526	TCGCCAACA	5.0000	NA	-6.8990	Significant
FAM19A1	chr3 68335501-68335508	TGTTGGCGT	0.5850	-0.0892	-6.1002	Significant
Intergenic	chr3 68624942-68624950	ACGCCAACA	0.1255	NA	-15.8069	Significant
Intergenic	chr3 72537803-72537811	TGATGGCGT	1.5850	NA	-12.4056	Significant
SHQ1	chr3 72798770-72798778	ACGCCAACA	5.0000	-1.4369	-7.6831	Significant
Intergenic	chr3 74991657-74991665	TGAAGGCGA	1.0000	NA	-5.4421	Significant
Intergenic	chr3 75900768-75900776	ACGCCTACA	2.0000	NA	-5.2170	Significant
Intergenic	chr3 76680488-76680496	TGTTGGCGT	-0.2630	NA	-4.7186	Significant
ROBO2	chr3 77097473-77097481	TCGCCTACA	3.9069	0.0888	-10.9476	Significant
ROBO2	chr3 77471353-77471361	TGAAGGCGA	3.3219	0.0888	-4.9166	Significant
Intergenic	chr3 78465000-78465008	ACGCCAACA	-1.0000	NA	-5.4216	Significant
Intergenic	chr3 80439973-80439981	ACGCCATCA	3.3219	NA	-8.9289	Significant
Intergenic	chr3 81471060-81471068	TGATGGCGA	3.0000	NA	-4.2710	Marginal
Intergenic	chr3 82006035-82006043	TGATGGCGA	0.2630	NA	-8.5043	Significant
Intergenic	chr3 84456069-84456077	TCGCCAACA	5.0000	NA	-16.1339	Significant
Intergenic	chr3 84456069-84456077	ACGCCAACA	0.7655	NA	-7.2864	Significant
Intergenic	chr3 84764278-84764286	ACGCCAACA	3.0000	NA	-5.0497	Significant
Intergenic	chr3 84818139-84818147	TGAAGGCGT	5.0000	NA	-6.8477	Significant
CADM2	chr3 86039601-86039609	ACGCCATCA	2.2224	-0.2873	-10.2089	Significant
Intergenic	chr3 86207352-86207359	TCGCCTTCA	5.0000	NA	-6.5209	Significant
Intergenic	chr3 86215435-86215442	TGAAGGCGT	3.5850	NA	-5.9549	Significant
Intergenic	chr3 87154761-87154769	TGTTGGCGT	5.0000	NA	-9.3124	Significant
Intergenic	chr3 89638211-89638219	TGATGGCGA	5.0000	NA	-6.5209	Significant
Intergenic	chr3 94208034-94208042	TGTTGGCGT	1.0780	NA	-7.0772	Significant
Intergenic	chr3 94291704-94291712	TGATGGCGA	2.7004	NA	-9.2913	Significant
Intergenic	chr3 94949617-94949625	TCGCCATCA	5.0000	NA	-10.4803	Significant
Intergenic	chr3 95214955-95214963	TCGCCATCA	5.0000	NA	-7.2866	Significant
Intergenic	chr3 95706578-95706586	TGTAGGCGT	0.4594	NA	-7.8722	Significant
EPHA6	chr3 97381327-97381335	TGAAGGCGA	1.4150	0.0413	-7.8720	Significant
EPHA6	chr3 97413115-97413123	TGTTGGCGT	1.3785	0.0413	-6.8473	Significant
EPHA6	chr3 97439145-97439153	TCGCCTACA	3.0000	0.0413	-8.8782	Significant
Intergenic	chr3 97916813-97916821	TCGCCTTCA	-0.4695	NA	-8.9291	Significant
COL8A1	chr3 99434804-99434812	ACGCCTTCA	5.0000	1.0053	-9.3124	Significant
TBC1D23	chr3 100010941-100010949	TGTAGGCGT	-0.6919	-1.8762	-12.6134	Significant
TMEM45A	chr3 100252978-100252986	TGTTGGCGA	5.0000	0.2329	-9.1342	Significant
Intergenic	chr3 101898406-101898414	TGAAGGCGT	5.0000	NA	-5.7407	Significant
Intergenic	chr3 104175445-104175453	TCGCCATCA	3.0000	NA	-5.9552	Significant
CBLB	chr3 105451254-105451262	TCGCCAACA	-1.7843	-3.3252	-5.9672	Significant
Intergenic	chr3 105908456-105908464	ACGCCTTCA	1.9069	NA	-6.1002	Significant
Intergenic	chr3 106881805-106881813	ACGCCTTCA	0.8480	NA	-7.4699	Significant
CD47	chr3 107808356-107808364	TGAAGGCGT	5.0000	-0.1208	-7.4724	Significant
MYH15	chr3 108154007-108154015	TGTTGGCGT	1.8074	0.0342	-6.3196	Significant
Intergenic	chr3 108879922-108879930	ACGCCATCA	5.0000	NA	-6.6939	Significant
Intergenic	chr3 109460012-109460020	TGTAGGCGT	5.0000	NA	-4.5890	Significant
Intergenic	chr3 111973197-111973205	TCGCCTACA	0.1255	NA	-8.5043	Significant
Intergenic	chr3 113014292-113014300	TCGCCAACA	2.0000	NA	-4.3961	Significant
SPICE1	chr3 113187974-113187982	TGATGGCGT	1.0000	-0.1163	-5.5994	Significant
ZBTB20	chr3 114347157-114347165	TGAAGGCGT	2.7004	-0.4022	-7.4701	Significant
ZBTB20	chr3 114522362-114522370	TGTTGGCGA	2.8074	-0.4022	-10.3119	Significant
ZBTB20	chr3 114615851-114615859	TGATGGCGA	0.3219	-0.4022	-7.4699	Significant
LSAMP	chr3 115918472-115918480	TGTAGGCGA	3.8074	-0.1275	-10.9476	Significant
Intergenic	chr3 116533549-116533557	ACGCCTACA	1.3219	NA	-5.0847	Significant
Intergenic	chr3 118218208-118218216	TCGCCATCA	0.2895	NA	-6.8473	Significant
Intergenic	chr3 119437624-119437632	TGAAGGCGA	3.7004	NA	-5.6140	Significant
Intergenic	chr3 119485512-119485520	TCGCCATCA	3.1699	NA	-6.8990	Significant
GPR156	chr3 119901967-119901975	ACGCCATCA	2.3219	0.2292	-7.0774	Significant
PARP15	chr3 122325574-122325582	TGTAGGCGA	2.5025	-0.1197	-10.4803	Significant
HACD2	chr3 123249156-123249164	ACGCCATCA	1.3219	0.0160	-6.3198	Significant
HEG1	chr3 124725611-124725619	TGAAGGCGA	1.2630	-0.3777	-7.8720	Significant
Intergenic	chr3 125584638-125584646	TCGCCTTCA	5.0000	NA	-12.1603	Significant
RAB7A	chr3 128448758-128448766	TGAAGGCGA	1.3219	-0.0231	-6.1002	Significant
Intergenic	chr3 129086262-129086270	TCGCCAACA	3.7004	NA	-11.9103	Significant
Intergenic	chr3 129869615-129869623	TGAAGGCGA	2.4150	NA	-10.6301	Significant
Promoter_COL6A6	chr3 130278779-130278787	TGATGGCGA	3.3219	0.1483	-12.4056	Significant

ATP2C1	chr3 130697426-130697434	TGATGGCGT	2.1699	0.0215	-6.6939	Significant
Intergenic	chr3 131887012-131887020	TCGCCTTCA	5.0000	NA	-13.9474	Significant
DNAJC13	chr3 132236984-132236992	TGTTGGCGT	-0.3479	-0.4843	-10.2089	Significant
NPHP3	chr3 132408625-132408633	TGTAGGCGA	5.0000	-1.2651	-9.7284	Significant
Intergenic	chr3 134421664-134421672	TGAAGGCGA	5.0000	NA	-4.5681	Significant
EPHB1	chr3 134781183-134781191	ACGCCAACA	5.0000	-0.0854	-9.7560	Significant
NCK1	chr3 136606314-136606322	TGAAGGCGA	1.5850	-3.5455	-6.3196	Significant
Intergenic	chr3 140304866-140304874	TCGCCTACA	2.1155	NA	-6.6941	Significant
Intergenic	chr3 141407223-141407231	TGATGGCGA	1.4594	NA	-7.0772	Significant
XRN1	chr3 142049703-142049711	TGATGGCGT	5.0000	-1.1999	-8.2836	Significant
U2SURP	chr3 142770197-142770205	ACGCCAACA	1.4150	-1.3224	-5.7407	Significant
SLC9A9	chr3 143455588-143455596	TGAAGGCGA	-1.7776	-0.1423	-4.9165	Significant
Intergenic	chr3 144307050-144307058	TGTAGGCGT	1.0000	NA	-8.2836	Significant
Intergenic	chr3 144759513-144759521	ACGCCAACA	-0.7105	NA	-6.4691	Significant
Intergenic	chr3 145598555-145598563	TCGCCATCA	1.5850	NA	-13.6846	Significant
Intergenic	chr3 146023413-146023421	ACGCCATCA	1.8745	NA	-7.0772	Significant
Intergenic	chr3 146295159-146295167	TGAAGGCGA	3.1699	NA	-5.9549	Significant
PLSCR5	chr3 146310371-146310379	TCGCCATCA	5.0000	0.2326	-9.5613	Significant
Intergenic	chr3 147984364-147984372	TCGCCATCA	5.0000	NA	-8.5043	Significant
Intergenic	chr3 148989185-148989193	ACGCCATCA	5.0000	NA	-5.1011	Significant
Intergenic	chr3 149754740-149754748	TGATGGCGT	-0.4854	NA	-7.6316	Significant
Intergenic	chr3 149860146-149860154	TGTAGGCGT	-1.7004	NA	-6.1006	Significant
Intergenic	chr3 150627319-150627327	TGAAGGCGA	2.3219	NA	-19.0766	Significant
Intergenic	chr3 150630420-150630428	TGTTGGCGT	5.0000	NA	-5.4423	Significant
AADACL2	chr3 151461939-151461947	TGTAGGCGT	2.7004	0.0552	-10.2089	Significant
Intergenic	chr3 152771856-152771864	ACGCCTTCA	5.0000	NA	-7.0774	Significant
Intergenic	chr3 152828965-152828973	TGTTGGCGT	5.0000	NA	-11.9103	Significant
Intergenic	chr3 153178051-153178059	ACGCCAACA	5.0000	NA	-7.8844	Significant
GPR149	chr3 154118562-154118570	TGAAGGCGA	3.0000	0.0698	-8.8782	Significant
MME	chr3 154798656-154798664	TGAAGGCGT	5.0000	0.1394	-5.2536	Significant
PLCH1	chr3 155208869-155208877	ACGCCAACA	3.4594	-2.5208	-6.8473	Significant
KCNAB1	chr3 155860811-155860819	TCGCCTACA	1.3219	0.0568	-8.0346	Significant
Intergenic	chr3 156386930-156386938	TCGCCAACA	2.1699	NA	-8.2834	Significant
Intergenic	chr3 156899242-156899250	TCGCCTTCA	5.0000	NA	-6.1520	Significant
Intergenic	chr3 156971661-156971669	TGTAGGCGT	3.4594	NA	-5.9549	Significant
VEPH1	chr3 157062909-157062917	ACGCCTTCA	2.4594	0.1655	-5.1017	Significant
Intergenic	chr3 157640946-157640954	TGTTGGCGA	0.6781	NA	-5.2533	Significant
IQCJ-SCHIP1	chr3 159052021-159052029	TGTTGGCGA	-0.7885	0.0846	-4.7182	Significant
SCHIP1	chr3 159052021-159052029	TGTTGGCGA	3.4594	-0.2278	-6.5211	Significant
Intergenic	chr3 159819484-159819492	ACGCCTTCA	0.8480	NA	-6.3297	Significant
Intergenic	chr3 159843566-159843574	ACGCCAACA	5.0000	NA	-4.8879	Significant
IFT80	chr3 160060561-160060569	TGAAGGCGT	1.8074	-0.4893	-8.4534	Significant
Intergenic	chr3 160288024-160288032	ACGCCATCA	1.2224	NA	-11.1427	Significant
Intergenic	chr3 160424727-160424735	TGATGGCGT	3.7004	NA	-10.0223	Significant
PPM1L	chr3 160626444-160626452	TGTAGGCGT	2.7004	-0.6099	-7.6316	Significant
Intergenic	chr3 161335786-161335794	TGAAGGCGA	1.3219	NA	-10.2089	Significant
Intergenic	chr3 162082072-162082080	TCGCCAACA	0.5146	NA	-8.0346	Significant
SI	chr3 164780442-164780450	ACGCCAACA	1.8074	0.1476	-6.4866	Significant
Intergenic	chr3 165567780-165567788	TCGCCAACA	-0.5305	NA	-10.2089	Significant
EGFEM1P	chr3 168428124-168428132	TGTAGGCGA	5.0000	0.2306	-10.4803	Significant
MECOM	chr3 169272126-169272134	TCGCCTACA	3.3219	-2.0819	-5.4423	Significant
SPATA16	chr3 172823954-172823962	TGAAGGCGT	1.3219	-0.0082	-8.2834	Significant
NLGN1	chr3 173228901-173228909	TGAAGGCGT	-0.3870	0.1432	-11.6236	Significant
NLGN1	chr3 173248408-173248416	TGTTGGCGT	0.7004	0.1432	-6.3196	Significant
NLGN1	chr3 173960649-173960657	TGTTGGCGA	2.2224	0.1432	-7.2352	Significant
NLGN1	chr3 173960671-173960679	TGTTGGCGA	-0.5305	0.1432	-6.6938	Significant
Intergenic	chr3 174062947-174062955	TGAAGGCGT	5.0000	NA	-5.0497	Significant
NAALADL2	chr3 174966503-174966511	TGAAGGCGT	5.0000	-0.1319	-4.9352	Significant
Intergenic	chr3 176695445-176695453	ACGCCAACA	1.5850	NA	-4.7693	Significant
Intergenic	chr3 176991196-176991204	TCGCCAACA	5.0000	NA	-5.9675	Significant
PIK3CA	chr3 178943684-178943692	TGATGGCGT	5.0000	-1.6245	-7.8720	Significant
Intergenic	chr3 181918065-181918073	TGAAGGCGA	2.3219	NA	-5.2701	Significant
Intergenic	chr3 183288068-183288076	TGTAGGCGT	0.0000	NA	-7.2348	Significant
TRA2B	chr3 185639828-185639836	TGTTGGCGT	2.5850	-1.2298	-11.6236	Significant
TBCCD1	chr3 186268991-186268999	TGTTGGCGA	0.7370	-1.8736	-5.9677	Significant

Intergenic	chr3 187075122-187075130	TGAAGGCGA	5.0000	NA	-8.7041	Significant
LPP	chr3 187981644-187981652	TGTTGGCGT	1.7370	-0.3880	-10.4806	Significant
Intergenic	chr3 189288272-189288280	TCGCCATCA	2.7004	NA	-8.6982	Significant
Intergenic	chr3 189344550-189344558	ACGCCTACA	0.2630	NA	-7.4699	Significant
CCDC50	chr3 191064083-191064091	TCGCCTTCA	3.1699	-0.9289	-8.8786	Significant
Intergenic	chr3 191228564-191228572	TGTTGGCGA	1.7370	NA	-7.2348	Significant
FGF12	chr3 192187682-192187690	ACGCCTACA	1.1375	0.1254	-12.4056	Significant
FGF12	chr3 192442630-192442638	TGTTGGCGA	1.1155	0.1254	-6.3196	Significant
Intergenic	chr3 192734035-192734043	ACGCCATCA	2.8074	NA	-6.1520	Significant
Intergenic	chr3 192954524-192954532	TGTAGGCGA	3.4594	NA	-5.2536	Significant
Intergenic	chr3 193279806-193279813	TGTTGGCGT	0.4150	NA	-7.4699	Significant
LOC100507391	chr3 194430431-194430439	TGATGGCGT	5.0000	-0.2698	-7.2348	Significant
Intergenic	chr3 194508615-194508623	TGATGGCGA	1.3219	NA	-8.0382	Significant
ACAP2	chr3 195027954-195027962	TGTTGGCGA	2.5850	-1.3837	-7.8720	Significant
Intergenic	chr3 195907578-195907586	TGATGGCGA	3.3219	NA	-7.8717	Significant
SENP5	chr3 196652515-196652523	TCGCCTTCA	2.0000	-1.8624	-14.0742	Significant
DLG1	chr3 196861097-196861105	TCGCCATCA	2.4594	-0.9618	-7.4699	Significant
DLG1	chr3 196874016-196874024	TGTTGGCGT	0.1069	-0.9618	-9.7560	Significant
DLG1	chr3 196991772-196991780	TGTAGGCGT	2.4594	-0.9618	-7.0823	Significant
LRCH3	chr3 197529636-197529644	TGTTGGCGT	5.0000	-0.3844	-11.1907	Significant
Intergenic	chr4 296383-296391	TCGCCTACA	3.1699	NA	-5.7407	Significant
RNF212	chr4 1097742-1097750	TGTTGGCGT	5.0000	0.2543	-11.4243	Significant
CTBP1	chr4 1212533-1212541	TGAAGGCGT	3.1699	-0.1703	-8.7041	Significant
WHSC1	chr4 1949435-1949443	TCGCCATCA	5.0000	-0.6196	-4.8879	Significant
POLN	chr4 2185728-2185736	ACGCCATCA	1.4854	0.0032	-6.8473	Significant
HTT	chr4 3206141-3206149	ACGCCTACA	5.0000	-0.4111	-7.7711	Significant
TBC1D14	chr4 6966284-6966292	TGATGGCGA	0.8745	-1.5376	-6.4691	Significant
SORCS2	chr4 7583898-7583906	TGAAGGCGT	3.5850	0.2313	-6.6941	Significant
AFAP1	chr4 7819942-7819950	ACGCCTTCA	0.5850	-1.2808	-7.4703	Significant
SLC2A9	chr4 9946730-9946738	TCGCCATCA	0.5850	-0.4049	-7.4703	Significant
CLNK	chr4 10644730-10644738	TCGCCTTCA	5.0000	0.1977	-7.8720	Significant
Intergenic	chr4 11037260-11037268	ACGCCAACA	1.3785	NA	-8.7041	Significant
Intergenic	chr4 12970526-12970534	ACGCCATCA	5.0000	NA	-8.8782	Significant
Intergenic	chr4 13189953-13189961	ACGCCAACA	5.0000	NA	-13.6886	Significant
Intergenic	chr4 13909789-13909797	TGTAGGCGA	5.0000	NA	-4.2708	Marginal
Intergenic	chr4 14353971-14353979	TGTAGGCGT	5.0000	NA	-6.4695	Significant
CC2D2A	chr4 15554039-15554047	TCGCCTTCA	3.9069	0.1296	-6.1002	Significant
CD38	chr4 15812857-15812865	TGATGGCGT	5.0000	0.0998	-8.7041	Significant
PROM1	chr4 15998356-15998364	TGATGGCGA	5.0000	0.0964	-11.6236	Significant
LDB2	chr4 16818641-16818649	TCGCCAACA	2.1155	0.2025	-9.1342	Significant
Intergenic	chr4 17434712-17434720	TGTTGGCGT	5.0000	NA	-9.8058	Significant
Intergenic	chr4 18090700-18090708	TGAAGGCGT	-0.8625	NA	-7.4699	Significant
Intergenic	chr4 18279648-18279656	TGATGGCGT	2.0000	NA	-10.0223	Significant
Intergenic	chr4 18756812-18756820	ACGCCAACA	5.0000	NA	-5.2533	Significant
Intergenic	chr4 18987073-18987081	ACGCCAACA	-1.3219	NA	-6.3196	Significant
Intergenic	chr4 19102061-19102069	TGAAGGCGT	2.4150	NA	-7.6316	Significant
Intergenic	chr4 20040413-20040421	TCGCCATCA	4.0875	NA	-12.4059	Significant
SLIT2	chr4 20581303-20581311	TGTTGGCGA	1.6781	0.0633	-6.4691	Significant
Intergenic	chr4 20635528-20635536	TGAAGGCGA	0.8074	NA	-5.7407	Significant
KCNIP4	chr4 21281551-21281559	TCGCCATCA	-0.7004	-0.7608	-5.7676	Significant
Intergenic	chr4 22326171-22326179	TGAAGGCGA	5.0000	NA	-4.2708	Marginal
Intergenic	chr4 22637726-22637734	TGATGGCGT	1.4594	NA	-6.1006	Significant
Intergenic	chr4 22639426-22639434	TCGCCATCA	1.8745	NA	-7.0772	Significant
Intergenic	chr4 24433192-24433200	TCGCCAACA	-1.3219	NA	-5.7924	Significant
Intergenic	chr4 24452061-24452069	TCGCCATCA	-0.8301	NA	-5.0497	Significant
CCDC149	chr4 24980139-24980147	TGTTGGCGT	2.1155	-1.2625	-7.6316	Significant
Intergenic	chr4 25597682-25597690	TGATGGCGT	-0.9511	NA	-7.0772	Significant
Intergenic	chr4 25728406-25728414	TCGCCTACA	2.5850	NA	-8.7043	Significant
TBC1D19	chr4 26590705-26590713	TCGCCAACA	3.0000	-0.3861	-8.4534	Significant
TBC1D19	chr4 26706538-26706546	TCGCCAACA	5.0000	-0.3861	-10.4803	Significant
Intergenic	chr4 26828342-26828350	TGTTGGCGA	5.0000	NA	-9.8058	Significant
Intergenic	chr4 31978148-31978156	TGATGGCGA	5.0000	NA	-10.9478	Significant
Intergenic	chr4 33643787-33643795	TCGCCAACA	-0.3479	NA	-8.4534	Significant
Intergenic	chr4 34126294-34126302	TGTTGGCGA	0.2895	NA	-7.4703	Significant
Intergenic	chr4 34169657-34169665	TGAAGGCGA	1.3219	NA	-6.8477	Significant

Intergenic	chr4 36640791-36640799	ACGCCTACA	5.0000	NA	-10.0223	Significant
Intergenic	chr4 37033979-37033987	TGAAGGCGT	2.1699	NA	-4.9168	Significant
Intergenic	chr4 37066734-37066742	TCGCCAACA	0.8480	NA	-5.7407	Significant
Intergenic	chr4 37728850-37728858	ACGCCTACA	1.0000	NA	-8.8786	Significant
Intergenic	chr4 38290571-38290579	ACGCCTTCA	0.6781	NA	-7.6316	Significant
RFC1	chr4 39306482-39306490	TCGCCTTCA	-0.8745	-2.3287	-9.3124	Significant
RFC1	chr4 39318061-39318069	ACGCCATCA	1.0000	-2.3287	-6.4691	Significant
APBB2	chr4 41204534-41204542	ACGCCAACA	3.4594	-1.2712	-8.7041	Significant
ATP8A1	chr4 42518683-42518691	ACGCCTTCA	5.0000	-0.5252	-15.5732	Significant
Intergenic	chr4 43900573-43900581	TCGCCAACA	1.0000	NA	-6.1520	Significant
Intergenic	chr4 44536862-44536870	TGTTGGCGA	2.1155	NA	-15.5732	Significant
Intergenic	chr4 45308529-45308537	TGAAGGCGT	5.0000	NA	-10.2089	Significant
Intergenic	chr4 45885546-45885554	TGTTGGCGA	-0.8931	NA	-6.3200	Significant
Intergenic	chr4 46399906-46399914	TGAAGGCGT	5.0000	NA	-10.4803	Significant
GABRA4	chr4 46966771-46966779	TCGCCTACA	5.0000	0.0815	-7.4699	Significant
SLAIN2	chr4 48354177-48354185	TGTAGGCGA	3.1699	-0.4373	-8.4534	Significant
Intergenic	chr4 48941589-48941597	TGAAGGCGA	0.7370	NA	-5.0497	Significant
Intergenic	chr4 48965795-48965803	ACGCCATCA	0.7370	NA	-8.5043	Significant
FIP1L1	chr4 54302584-54302592	TGTAGGCGT	1.4150	-4.0808	-12.4057	Significant
LNX1	chr4 54396127-54396135	TGTAGGCGA	0.3479	-1.7439	-6.4691	Significant
Intergenic	chr4 54584253-54584261	TCGCCATCA	0.0000	NA	-8.2836	Significant
Intergenic	chr4 55446908-55446916	TCGCCAACA	5.0000	NA	-12.6591	Significant
KIT	chr4 55585681-55585689	TGTTGGCGA	5.0000	-1.5484	-9.3627	Significant
KDR	chr4 55955591-55955599	TCGCCTACA	2.4594	0.1978	-7.0774	Significant
Intergenic	chr4 56556529-56556537	TGTTGGCGT	3.3219	NA	-7.3789	Significant
Intergenic	chr4 58221458-58221466	TGTTGGCGT	1.0000	NA	-6.4691	Significant
Intergenic	chr4 59219324-59219332	ACGCCTACA	1.2630	NA	-5.9549	Significant
Intergenic	chr4 59257808-59257816	TGAAGGCGA	1.8074	NA	-9.5736	Significant
Intergenic	chr4 60125917-60125925	TCGCCATCA	3.4594	NA	-6.6939	Significant
Intergenic	chr4 62018918-62018926	TCGCCATCA	2.1699	NA	-6.8473	Significant
LPHN3	chr4 62466935-62466943	TGTAGGCGA	5.0000	0.1962	-6.1132	Significant
Intergenic	chr4 68877024-68877032	TCGCCATCA	5.0000	NA	-8.8782	Significant
YTHDC1	chr4 69185920-69185928	ACGCCTACA	5.0000	0.0614	-5.9672	Significant
UGT2B11	chr4 70071698-70071706	TGTTGGCGA	1.7004	0.2199	-6.6938	Significant
Intergenic	chr4 70592190-70592198	TGTAGGCGT	1.0704	NA	-7.6831	Significant
RUFY3	chr4 71607007-71607015	TCGCCTACA	-0.1375	-0.2665	-7.6316	Significant
Intergenic	chr4 71706872-71706880	TGATGGCGT	1.4594	NA	-5.2534	Significant
SLC4A4	chr4 72244800-72244808	ACGCCTTCA	0.7370	0.1472	-16.9469	Significant
GC	chr4 72613314-72613322	TGTTGGCGT	5.0000	0.1452	-5.9672	Significant
Intergenic	chr4 73696962-73696970	ACGCCTTCA	5.0000	NA	-9.1249	Significant
Intergenic	chr4 74520307-74520315	TCGCCATCA	2.9069	NA	-10.0223	Significant
Intergenic	chr4 74532188-74532196	TCGCCATCA	5.0000	NA	-7.6316	Significant
Intergenic	chr4 74604366-74604374	TCGCCTACA	1.4594	NA	-9.1344	Significant
USO1	chr4 76665619-76665627	TCGCCAACA	-1.3923	-0.6068	-4.3965	Significant
SHROOM3	chr4 77551854-77551862	TGTAGGCGA	0.7370	-2.6532	-16.9469	Significant
SHROOM3	chr4 77628367-77628375	ACGCCTACA	2.5850	-2.6532	-7.2348	Significant
Intergenic	chr4 77799162-77799170	ACGCCAACA	5.0000	NA	-4.9166	Significant
Intergenic	chr4 78143301-78143309	TGATGGCGT	2.3219	NA	-9.3124	Significant
Intergenic	chr4 78609418-78609426	TGAAGGCGA	3.3219	NA	-8.0378	Significant
Intergenic	chr4 78880214-78880222	TGATGGCGA	5.0000	NA	-19.9812	Significant
Intergenic	chr4 81093334-81093342	TCGCCTACA	0.7370	NA	-7.2864	Significant
Intergenic	chr4 82677909-82677917	TCGCCATCA	5.0000	NA	-22.2523	Significant
SCD5	chr4 83575678-83575686	TGAAGGCGT	0.0000	0.1422	-7.0772	Significant
SCD5	chr4 83624238-83624246	TGAAGGCGA	0.3219	0.1422	-7.6316	Significant
SCD5	chr4 83693241-83693249	TGATGGCGA	3.4594	0.1422	-8.8782	Significant
Intergenic	chr4 84150752-84150760	TGATGGCGA	5.0000	NA	-10.9247	Significant
Intergenic	chr4 84163064-84163072	TCGCCAACA	5.0000	NA	-10.1747	Significant
Intergenic	chr4 84448540-84448548	TCGCCATCA	-0.4475	NA	-5.7411	Significant
MAPK10	chr4 87166612-87166620	ACGCCATCA	5.0000	0.0321	-7.6831	Significant
MAPK10	chr4 87263531-87263539	TGTAGGCGT	0.3219	0.0321	-8.2836	Significant
MAPK10	chr4 87320408-87320416	TCGCCAACA	2.4594	0.0321	-6.8473	Significant
Intergenic	chr4 87479715-87479723	TGTTGGCGT	0.6781	NA	-13.6849	Significant
Intergenic	chr4 88071800-88071808	TGTAGGCGT	2.0000	NA	-4.7567	Significant
FAM13A	chr4 89673845-89673853	ACGCCAACA	3.0875	-2.1126	-7.4699	Significant
SNCA	chr4 90656738-90656746	TGAAGGCGA	-0.9329	-0.0001	-6.3297	Significant

Intergenic	chr4 91140663-91140671	TCGCCTTCA	5.0000	NA	-9.5736	Significant
Intergenic	chr4 92793805-92793813	TGATGGCGT	3.7004	NA	-6.6938	Significant
Intergenic	chr4 92872565-92872573	ACGCCAACA	5.0000	NA	-5.2533	Significant
GRID2	chr4 93919941-93919949	TGATGGCGT	3.5850	0.6394	-5.7407	Significant
GRID2	chr4 94120902-94120910	TGTTGGCGT	1.1375	0.6394	-7.6316	Significant
Intergenic	chr4 94959224-94959232	ACGCCATCA	-0.5619	NA	-7.0775	Significant
PDLIM5	chr4 95427366-95427374	ACGCCATCA	1.5850	-0.2987	-19.6929	Significant
PDLIM5	chr4 95552607-95552615	ACGCCTTCA	5.0000	-0.2987	-16.1339	Significant
Intergenic	chr4 97068815-97068823	TCGCCTTCA	-0.3219	NA	-7.2348	Significant
Intergenic	chr4 97508745-97508753	ACGCCATCA	5.0000	NA	-7.4699	Significant
Intergenic	chr4 97597816-97597824	TCGCCAACA	5.0000	NA	-5.5546	Significant
Intergenic	chr4 97943424-97943432	TCGCCTTCA	5.0000	NA	-10.2089	Significant
Intergenic	chr4 98199474-98199482	TGTTGGCGT	5.0000	NA	-9.3627	Significant
Intergenic	chr4 98268245-98268253	TGTTGGCGT	5.0000	NA	-5.3317	Significant
Intergenic	chr4 98430236-98430244	TCGCCTTCA	5.0000	NA	-10.6715	Significant
MTTP	chr4 100505711-100505719	TCGCCTACA	-0.7370	-0.1399	-9.3124	Significant
EMCN	chr4 101403802-101403810	TGAAGGCGA	5.0000	0.0519	-5.2533	Significant
BANK1	chr4 102906515-102906523	ACGCCTTCA	1.5850	-0.2032	-6.4691	Significant
Intergenic	chr4 103549949-103549957	TCGCCATCA	5.0000	NA	-8.2834	Significant
BDH2	chr4 104020954-104020962	TGATGGCGT	5.0000	0.1508	-7.9758	Significant
Intergenic	chr4 105327273-105327281	TGAAGGCGA	-0.1375	NA	-8.0378	Significant
Intergenic	chr4 105351869-105351877	TCGCCTTCA	3.4594	NA	-14.4800	Significant
TET2	chr4 106151848-106151856	TGTAGGCGT	5.0000	-1.9562	-11.3976	Significant
PPA2	chr4 106332845-106332853	ACGCCATCA	5.0000	0.2199	-7.2348	Significant
Intergenic	chr4 109110292-109110300	TGTAGGCGA	2.5850	NA	-6.1002	Significant
Intergenic	chr4 109192627-109192635	ACGCCATCA	5.0000	NA	-7.2864	Significant
Intergenic	chr4 109286818-109286826	TGTAGGCGA	5.0000	NA	-5.7669	Significant
Intergenic	chr4 113618469-113618477	TGAAGGCGT	2.2224	NA	-6.6941	Significant
ANK2	chr4 114163211-114163219	TCGCCATCA	0.5850	0.1155	-12.4056	Significant
Intergenic	chr4 117625215-117625223	TGTAGGCGT	2.4594	NA	-6.4695	Significant
Intergenic	chr4 118896970-118896978	TCGCCATCA	3.7004	NA	-4.9168	Significant
Intergenic	chr4 120374270-120374278	TCGCCAACA	5.0000	NA	-4.7182	Significant
PRDM5	chr4 121705412-121705420	TGTAGGCGT	5.0000	-0.0653	-6.3196	Significant
Intergenic	chr4 122473424-122473432	TGATGGCGA	1.0000	NA	-7.0823	Significant
Intergenic	chr4 122989164-122989172	TGAAGGCGT	2.3219	NA	-4.7572	Significant
Intergenic	chr4 123699753-123699761	TGAAGGCGA	5.0000	NA	-24.5658	Significant
FGF2	chr4 123775383-123775391	TCGCCTACA	3.7004	0.0798	-9.5736	Significant
Intergenic	chr4 125561314-125561322	ACGCCTTCA	2.3219	NA	-5.3910	Significant
Intergenic	chr4 126472823-126472831	TGTAGGCGT	-0.8074	NA	-7.6316	Significant
Intergenic	chr4 126540735-126540743	TCGCCTTCA	5.0000	NA	-7.4699	Significant
C4orf29	chr4 128917545-128917553	TGATGGCGA	0.7105	-0.9412	-5.3906	Significant
Intergenic	chr4 129287471-129287479	ACGCCTTCA	5.0000	NA	-10.4804	Significant
Intergenic	chr4 129292551-129292559	TGTTGGCGA	5.0000	NA	-14.1681	Significant
Intergenic	chr4 131393851-131393859	ACGCCATCA	1.7370	NA	-7.6833	Significant
Intergenic	chr4 131721244-131721252	TCGCCAACA	2.2224	NA	-7.8720	Significant
Intergenic	chr4 134706462-134706470	ACGCCAACA	4.0875	NA	-7.8720	Significant
Intergenic	chr4 134852578-134852586	TCGCCTTCA	5.0000	NA	-10.4804	Significant
Intergenic	chr4 135151092-135151100	TGTTGGCGT	0.0000	NA	-6.1002	Significant
Intergenic	chr4 137896315-137896323	TCGCCATCA	1.4854	NA	-6.3196	Significant
Intergenic	chr4 138498459-138498467	ACGCCTACA	1.0000	NA	-8.2838	Significant
Intergenic	chr4 139576108-139576116	ACGCCATCA	-0.5850	NA	-9.1342	Significant
Intergenic	chr4 139826132-139826140	ACGCCTTCA	3.0000	NA	-18.7577	Significant
Intergenic	chr4 140330599-140330607	TGAAGGCGA	2.4594	NA	-8.0378	Significant
MAML3	chr4 140952353-140952361	ACGCCATCA	0.0000	-0.5067	-10.4609	Significant
CLGN	chr4 141322980-141322988	TGAAGGCGA	5.0000	0.2075	-12.4056	Significant
RNF150	chr4 141861320-141861328	TCGCCAACA	1.2630	0.2221	-8.7041	Significant
RNF150	chr4 142022175-142022183	TGAAGGCGT	2.4150	0.2221	-8.0378	Significant
Intergenic	chr4 142215561-142215569	ACGCCATCA	5.0000	NA	-16.7038	Significant
Intergenic	chr4 142271306-142271314	TGAAGGCGA	1.6630	NA	-12.1606	Significant
Intergenic	chr4 142399512-142399520	TGTTGGCGT	2.4150	NA	-8.9293	Significant
Intergenic	chr4 142777100-142777108	TCGCCATCA	2.8074	NA	-5.6140	Significant
GAB1	chr4 144322994-144323002	TCGCCTTCA	1.3219	0.1399	-5.7407	Significant
Promoter_SMARCA5	chr4 144433840-144433848	TGTAGGCGA	5.0000	-0.5622	-12.3712	Significant
Intergenic	chr4 145196868-145196876	TGTTGGCGA	1.0000	NA	-8.7043	Significant
Intergenic	chr4 145563263-145563271	TGTTGGCGT	2.8074	NA	-9.7560	Significant

HHIP	chr4	145620376-145620384	TCGCCTTCA	1.1699	-0.0703	-8.4534	Significant
Intergenic	chr4	146121397-146121405	TGTTGGCGT	-0.0995	NA	-10.0224	Significant
TTC29	chr4	147692642-147692650	TGTTGGCGA	1.0000	0.1509	-6.4691	Significant
Intergenic	chr4	148506466-148506474	ACGCCTTCA	1.1155	NA	-9.7560	Significant
NR3C2	chr4	149290955-149290963	TGAAGGCGA	5.0000	-2.3433	-15.0219	Significant
Intergenic	chr4	149924085-149924093	ACGCCTTCA	5.0000	NA	-10.9476	Significant
LRBA	chr4	151522127-151522135	TCGCCTTCA	0.0000	-0.0771	-6.6938	Significant
FAM160A1	chr4	152536268-152536276	ACGCCATCA	1.2224	-2.2999	-16.1339	Significant
FBXW7	chr4	153402758-153402766	ACGCCTTCA	5.0000	-4.0183	-10.1750	Significant
Intergenic	chr4	157463700-157463708	TGAAGGCGA	0.4854	NA	-5.9549	Significant
Intergenic	chr4	157514146-157514154	TCGCCAACA	3.0000	NA	-5.7407	Significant
PDGFC	chr4	157851290-157851298	TCGCCTACA	-2.2479	-3.2077	-5.9672	Significant
Intergenic	chr4	158666346-158666354	TGTTGGCGA	3.4594	NA	-7.2352	Significant
Intergenic	chr4	158886526-158886534	TCGCCTTCA	5.0000	NA	-8.2836	Significant
FNIP2	chr4	159756136-159756144	TCGCCTACA	0.1375	-1.8057	-5.7407	Significant
Intergenic	chr4	160029366-160029374	TCGCCTTCA	1.5850	NA	-8.4534	Significant
Intergenic	chr4	160629328-160629336	TGTTGGCGT	5.0000	NA	-5.7407	Significant
Intergenic	chr4	161555998-161556006	TGAAGGCGA	1.5850	NA	-8.5820	Significant
FSTL5	chr4	163022212-163022220	TCGCCTTCA	1.2224	-2.0869	-11.4243	Significant
NPY5R	chr4	164270151-164270159	ACGCCAACA	2.3219	0.0644	-8.7041	Significant
1-Mar	chr4	164693550-164693558	TGTAGGCGT	5.0000	0.0920	-5.0501	Significant
Intergenic	chr4	166639588-166639596	TCGCCATCA	5.0000	NA	-7.0772	Significant
DDX60L	chr4	169343986-169343994	ACGCCATCA	0.6521	0.0716	-5.7407	Significant
SH3RF1	chr4	170090094-170090102	TCGCCTACA	2.9069	-4.4126	-7.6831	Significant
NEK1	chr4	170324306-170324314	ACGCCTACA	3.9069	-0.5064	-8.0378	Significant
Intergenic	chr4	171030183-171030191	TGTAGGCGT	-1.1844	NA	-9.1344	Significant
Intergenic	chr4	171923982-171923990	TGAAGGCGA	2.4594	NA	-9.1249	Significant
Intergenic	chr4	172322294-172322302	TGTTGGCGT	5.0000	NA	-6.2573	Significant
GALNTL6	chr4	173605376-173605384	TGATGGCGT	5.0000	-0.0579	-19.9576	Significant
GALNTL6	chr4	173809715-173809723	TGTAGGCGT	2.4594	-0.0579	-13.4245	Significant
Intergenic	chr4	174481101-174481109	TGTTGGCGT	2.5850	NA	-13.4243	Significant
Intergenic	chr4	174701052-174701060	TGATGGCGA	0.1375	NA	-7.2866	Significant
FBXO8	chr4	175202293-175202301	TGATGGCGT	5.0000	-2.0264	-7.6320	Significant
FBXO8	chr4	175202701-175202709	TGATGGCGT	5.0000	-2.0264	-13.9474	Significant
Intergenic	chr4	175819157-175819165	TCGCCATCA	5.0000	NA	-10.6711	Significant
Intergenic	chr4	179073106-179073114	TCGCCAACA	5.0000	NA	-7.0772	Significant
Intergenic	chr4	180230891-180230899	TCGCCAACA	2.3219	NA	-6.8473	Significant
Intergenic	chr4	180437735-180437742	TCGCCAACA	5.0000	NA	-10.0064	Significant
LINC00290	chr4	182011602-182011610	TCGCCTTCA	3.8074	-0.0687	-9.7560	Significant
LINC00290	chr4	182030485-182030493	ACGCCATCA	3.0000	-0.0687	-5.7411	Significant
Intergenic	chr4	182573528-182573536	TCGCCTACA	1.7370	NA	-5.7411	Significant
Intergenic	chr4	182607264-182607272	TGTAGGCGA	-0.5850	NA	-6.6941	Significant
Intergenic	chr4	183687426-183687434	TCGCCATCA	2.0000	NA	-8.4538	Significant
WWC2	chr4	184064427-184064435	TGATGGCGT	-0.1255	-0.8749	-5.1013	Significant
Intergenic	chr4	184805747-184805755	TGAAGGCGT	5.0000	NA	-11.6236	Significant
IRF2	chr4	185319034-185319042	TGTTGGCGA	0.5146	-0.3688	-5.2534	Significant
Intergenic	chr4	185445412-185445420	TGTTGGCGT	1.0000	NA	-7.2348	Significant
ACSL1	chr4	185745868-185745876	TGATGGCGA	5.0000	-0.2664	-8.7041	Significant
SLC25A4	chr4	186065086-186065094	ACGCCTTCA	5.0000	-0.1168	-4.7693	Significant
SORBS2	chr4	186666711-186666719	TGATGGCGA	1.0000	-0.6460	-14.4800	Significant
SORBS2	chr4	186776380-186776388	TGAAGGCGT	5.0000	-0.6460	-5.7924	Significant
SORBS2	chr4	186847521-186847529	TCGCCTACA	1.6781	-0.6460	-6.4691	Significant
FAT1	chr4	187512888-187512896	TGTAGGCGT	5.0000	-1.0567	-10.0066	Significant
Intergenic	chr4	187702338-187702345	TGAAGGCGA	3.7004	NA	-5.9672	Significant
LOC339975	chr4	188236948-188236956	TGTTGGCGT	1.8745	0.0828	-7.4699	Significant
Intergenic	chr4	189419155-189419163	TGATGGCGA	5.0000	NA	-12.9102	Significant
Intergenic	chr4	189798377-189798385	TCGCCTTCA	-0.6781	NA	-7.4699	Significant
Intergenic	chr4	190109555-190109563	ACGCCAACA	5.0000	NA	-5.4421	Significant
Intergenic	chr4	190194556-190194564	ACGCCTACA	0.1699	NA	-5.6140	Significant
Intergenic	chr4	190472272-190472280	ACGCCATCA	1.2224	NA	-7.8720	Significant
Intergenic	chr4	190476346-190476354	ACGCCATCA	0.2895	NA	-7.8720	Significant
Intergenic	chr5	30067-30075	TGATGGCGA	2.4594	NA	-11.9104	Significant
Intergenic	chr5	3685489-3685497	ACGCCATCA	5.0000	NA	-4.2931	Marginal
Intergenic	chr5	4481552-4481560	TGATGGCGA	3.3219	NA	-7.4699	Significant
Intergenic	chr5	5440443-5440451	TGAAGGCGA	1.7004	NA	-5.2533	Significant

Intergenic	chr5 5543155-5543163	TCGCCTTCA	3.5850	NA	-7.2348	Significant
Intergenic	chr5 8180969-8180977	TGTTGGCGA	2.0000	NA	-5.3906	Significant
Intergenic	chr5 8812068-8812076	TGTTGGCGA	3.5850	NA	-15.0219	Significant
LOC285692	chr5 9740381-9740389	TGTAGGCGA	0.8074	0.0069	-8.7041	Significant
Intergenic	chr5 10090637-10090645	ACGCCTACA	2.1699	NA	-11.4243	Significant
6-Mar	chr5 10411991-10411999	TGTAGGCGA	5.0000	-0.8487	-7.8720	Significant
CTNND2	chr5 11120313-11120321	ACGCCTACA	5.0000	0.0513	-6.6938	Significant
CTNND2	chr5 11814413-11814421	TGAAGGCGT	5.0000	0.0513	-12.9104	Significant
Intergenic	chr5 13198774-13198782	TGATGGCGT	3.1699	NA	-5.5994	Significant
DNAH5	chr5 13766505-13766513	TGTTGGCGA	1.8745	0.2887	-6.6938	Significant
FBXL7	chr5 15906229-15906237	TCGCCAACA	2.0000	0.2067	-6.3196	Significant
MYO10	chr5 16713930-16713939	TCGCCTTCA	3.4594	-0.5466	-6.8990	Significant
LOC285696	chr5 17176442-17176450	TGTAGGCGT	1.0000	0.2212	-6.6939	Significant
Intergenic	chr5 17642140-17642148	TGAAGGCGT	2.7004	NA	-7.2352	Significant
Intergenic	chr5 17721799-17721807	TGTTGGCGT	-0.8480	NA	-6.8477	Significant
Intergenic	chr5 17784522-17784530	ACGCCATCA	5.0000	NA	-9.1342	Significant
Intergenic	chr5 18286914-18286922	ACGCCATCA	1.1375	NA	-8.8782	Significant
CDH18	chr5 19836853-19836861	ACGCCTACA	5.0000	0.1134	-8.8782	Significant
GUSBP1	chr5 21556645-21556653	TCGCCAACA	1.3219	-0.7795	-6.4691	Significant
Intergenic	chr5 24380270-24380278	TGATGGCGT	5.0000	NA	-10.6711	Significant
Intergenic	chr5 24380522-24380530	TGAAGGCGT	5.0000	NA	-7.2348	Significant
Intergenic	chr5 25703985-25703993	TGTTGGCGT	-0.3785	NA	-7.0774	Significant
Intergenic	chr5 25860453-25860461	TGAAGGCGA	-0.4594	NA	-6.1002	Significant
Intergenic	chr5 30475837-30475845	TGATGGCGT	3.1699	NA	-6.6939	Significant
CDH6	chr5 31301340-31301348	ACGCCTACA	5.0000	0.1545	-5.9549	Significant
PDZD2	chr5 32110479-32110487	TCGCCAACA	1.4594	0.0600	-9.3128	Significant
ADAMTS12	chr5 33554440-33554448	ACGCCATCA	5.0000	0.3965	-5.6140	Significant
RAI14	chr5 34748739-34748747	TGTTGGCGT	2.4594	-0.7597	-12.4057	Significant
Intergenic	chr5 37754852-37754860	TCGCCTTCA	2.3219	NA	-6.8473	Significant
Intergenic	chr5 38041904-38041912	ACGCCATCA	5.0000	NA	-6.6938	Significant
C7	chr5 40923477-40923485	ACGCCTTCA	-1.0995	0.0258	-7.8724	Significant
Intergenic	chr5 41042908-41042916	TGTTGGCGA	2.3219	NA	-7.2864	Significant
Intergenic	chr5 42243474-42243482	TGAAGGCGA	3.3219	NA	-9.3124	Significant
NNT	chr5 43703186-43703194	TCGCCTTCA	5.0000	0.1069	-18.7250	Significant
Intergenic	chr5 43888253-43888261	ACGCCTACA	2.1155	NA	-11.9103	Significant
Intergenic	chr5 50419056-50419064	ACGCCATCA	-1.4406	NA	-5.3906	Significant
PPAP2A	chr5 54798764-54798772	ACGCCTTCA	2.3219	-1.7289	-5.9552	Significant
Intergenic	chr5 56281463-56281471	TGATGGCGT	5.0000	NA	-6.3196	Significant
Intergenic	chr5 58170537-58170545	TCGCCTTCA	2.4594	NA	-6.6938	Significant
Intergenic	chr5 58260401-58260409	TGATGGCGA	1.3219	NA	-5.9549	Significant
PDE4D	chr5 58994126-58994134	TGAAGGCGA	5.0000	-1.1468	-9.3627	Significant
PDE4D	chr5 59261986-59261994	TCGCCAACA	5.0000	-1.1468	-10.1266	Significant
PDE4D	chr5 59690799-59690807	TCGCCTTCA	5.0000	-1.1468	-15.0221	Significant
PDE4D	chr5 59746757-59746765	TCGCCTACA	5.0000	-1.1468	-9.3627	Significant
ADAMTS6	chr5 64474841-64474849	ACGCCTACA	0.0000	0.2577	-10.9478	Significant
SREK1	chr5 65449351-65449359	TGTTGGCGT	0.5146	0.0710	-10.9247	Significant
MAST4	chr5 65921474-65921482	TGTTGGCGA	5.0000	-1.1095	-6.5209	Significant
MAST4	chr5 66367042-66367050	TGAAGGCGA	0.8074	-1.1095	-6.4691	Significant
Intergenic	chr5 67106267-67106275	TGTTGGCGT	5.0000	NA	-8.6982	Significant
PIK3R1	chr5 67523638-67523646	TCGCCTTCA	-0.4406	-3.1045	-6.8477	Significant
Intergenic	chr5 70872350-70872358	TCGCCTTCA	5.0000	NA	-9.7560	Significant
MRPS27	chr5 71537667-71537674	TCGCCATCA	1.6781	-0.6258	-5.9550	Significant
Intergenic	chr5 71846785-71846793	ACGCCTACA	5.0000	NA	-7.2348	Significant
Intergenic	chr5 72058655-72058663	ACGCCTTCA	2.0000	NA	-8.0378	Significant
Intergenic	chr5 72478864-72478872	TGATGGCGA	5.0000	NA	-9.1249	Significant
Intergenic	chr5 72950951-72950959	ACGCCTTCA	3.0000	NA	-7.8720	Significant
Intergenic	chr5 73059244-73059252	TGTAGGCGA	2.9069	NA	-8.2805	Significant
Intergenic	chr5 73744215-73744223	TGAAGGCGT	5.0000	NA	-5.7924	Significant
GFM2	chr5 74018027-74018035	TCGCCAACA	5.0000	-0.2907	-4.2929	Marginal
Intergenic	chr5 74541011-74541019	TCGCCAACA	1.9260	NA	-7.8717	Significant
POLK	chr5 74890167-74890175	TCGCCTTCA	-0.0780	-1.0706	-6.4691	Significant
Intergenic	chr5 77602921-77602929	TGTAGGCGT	1.3219	NA	-6.3196	Significant
LHFPL2	chr5 77872384-77872392	TGTAGGCGT	3.1699	-3.7837	-9.1251	Significant
Intergenic	chr5 79107564-79107572	TGAAGGCGA	5.0000	NA	-13.6844	Significant
SSBP2	chr5 80724444-80724452	TCGCCATCA	3.0000	-1.2294	-6.4691	Significant

ATG10	chr5 81506524-81506532	TGATGGCGA	0.5305	-0.5705	-10.2089	Significant
Intergenic	chr5 81700413-81700421	TGTTGGCGT	-1.1375	NA	-5.2533	Significant
XRCC4	chr5 82547385-82547393	TGTAGGCGT	1.2630	-1.1821	-8.2836	Significant
VCAN	chr5 82786615-82786623	TGAAGGCGA	0.3785	-0.1279	-10.2089	Significant
Intergenic	chr5 84538946-84538954	TGATGGCGA	5.0000	NA	-8.4534	Significant
Intergenic	chr5 84763212-84763220	TGAAGGCGA	0.5850	NA	-6.8481	Significant
Intergenic	chr5 85662466-85662474	TGTTGGCGA	2.4594	NA	-5.2533	Significant
Intergenic	chr5 86374938-86374946	TCGCCATCA	5.0000	NA	-4.4469	Significant
Intergenic	chr5 89271279-89271287	TCGCCTTCA	3.4594	NA	-6.1924	Significant
Intergenic	chr5 89674727-89674735	TGAAGGCGA	3.3219	NA	-11.4197	Significant
GPR98	chr5 90442167-90442175	TGTAGGCGA	5.0000	-0.7729	-16.9461	Significant
Intergenic	chr5 90869710-90869718	TGATGGCGA	5.0000	NA	-13.4241	Significant
Intergenic	chr5 92100075-92100083	ACGCCAACA	0.7004	NA	-8.4534	Significant
Intergenic	chr5 92579992-92580000	TCGCCTTCA	5.0000	NA	-11.6236	Significant
Intergenic	chr5 92666527-92666535	TGTTGGCGA	3.1699	NA	-9.1251	Significant
FAM172A	chr5 93280109-93280117	TCGCCAACA	-1.2801	-0.1991	-7.6316	Significant
MCTP1	chr5 94188308-94188316	TGTTGGCGT	1.5850	-0.7644	-6.8477	Significant
MCTP1	chr5 94274916-94274924	ACGCCAACA	3.4594	-0.7644	-6.3196	Significant
MCTP1	chr5 94462070-94462078	TGAAGGCGA	2.9069	-0.7644	-9.5736	Significant
Intergenic	chr5 95840910-95840917	ACGCCTACA	5.0000	NA	-9.7560	Significant
CAST	chr5 96077027-96077035	TCGCCTACA	2.1155	-0.3042	-6.4691	Significant
Intergenic	chr5 96911448-96911456	TGATGGCGT	3.1699	NA	-7.8722	Significant
Intergenic	chr5 97582057-97582065	TGTAGGCGT	5.0000	NA	-4.7693	Significant
Intergenic	chr5 98155028-98155036	ACGCCTACA	1.0000	NA	-4.2708	Marginal
Intergenic	chr5 99426151-99426159	TGAAGGCGA	3.4594	NA	-5.7676	Significant
PAM	chr5 102298467-102298475	ACGCCTACA	1.0000	0.0760	-10.2581	Significant
Intergenic	chr5 104348556-104348564	TCGCCTTCA	5.0000	NA	-4.9165	Significant
Intergenic	chr5 106276470-106276478	TCGCCTTCA	5.0000	NA	-9.7560	Significant
Intergenic	chr5 106406257-106406264	TGAAGGCGT	1.5850	NA	-13.4241	Significant
FBXL17	chr5 107679064-107679072	TGAAGGCGA	3.5850	-0.4480	-12.9104	Significant
Intergenic	chr5 109529314-109529322	TGTAGGCGT	5.0000	NA	-8.6984	Significant
MCC	chr5 112609835-112609843	ACGCCATCA	2.5025	0.4010	-12.3714	Significant
Intergenic	chr5 113388880-113388888	TGTAGGCGT	3.1699	NA	-14.7469	Significant
Intergenic	chr5 115072421-115072429	TCGCCTTCA	1.8745	NA	-6.1002	Significant
Intergenic	chr5 115136034-115136042	ACGCCTTCA	2.8074	NA	-5.3906	Significant
AP3S1	chr5 115236242-115236250	TGTTGGCGA	3.5850	0.2359	-11.7047	Significant
Intergenic	chr5 115364171-115364179	TGAAGGCGT	0.3219	NA	-9.7560	Significant
Intergenic	chr5 115405976-115405984	TCGCCAACA	1.5850	NA	-4.2708	Marginal
Intergenic	chr5 117920945-117920953	TGTTGGCGA	0.1699	NA	-7.6316	Significant
Intergenic	chr5 118946345-118946353	TGATGGCGA	5.0000	NA	-8.4534	Significant
PRR16	chr5 119884438-119884446	TGAAGGCGT	0.4854	0.2270	-14.4288	Significant
Intergenic	chr5 120754404-120754412	TCGCCAACA	0.0000	NA	-5.4423	Significant
Intergenic	chr5 121584034-121584042	TGATGGCGA	5.0000	NA	-5.6140	Significant
Intergenic	chr5 121629838-121629846	TCGCCAACA	2.0704	NA	-6.8473	Significant
SNX2	chr5 122113992-122114000	TCGCCAACA	0.4594	-1.0029	-7.6833	Significant
CSNK1G3	chr5 122868324-122868332	TGATGGCGT	-0.3219	-3.2948	-12.4056	Significant
Intergenic	chr5 123773394-123773402	ACGCCAACA	2.0000	NA	-9.7560	Significant
Intergenic	chr5 124518445-124518453	TGTTGGCGA	2.0000	NA	-9.7560	Significant
Intergenic	chr5 124672646-124672654	TGTTGGCGA	-0.4150	NA	-5.1011	Significant
Intergenic	chr5 126178860-126178868	TGAAGGCGA	2.5850	NA	-4.4471	Significant
3-Mar	chr5 126248631-126248639	TGTAGGCGA	1.7370	-2.8645	-7.4729	Significant
MEGF10	chr5 126660665-126660673	TCGCCATCA	2.0000	0.1458	-6.8992	Significant
Intergenic	chr5 126806187-126806195	TGATGGCGT	3.4594	NA	-8.7041	Significant
Intergenic	chr5 127179504-127179512	TGTAGGCGA	1.0000	NA	-8.4534	Significant
FBN2	chr5 127817604-127817612	ACGCCTTCA	-0.2895	0.2362	-7.2352	Significant
CHSY3	chr5 129503887-129503895	ACGCCTTCA	5.0000	0.3168	-12.1603	Significant
Intergenic	chr5 129862644-129862652	TCGCCATCA	5.0000	NA	-13.9006	Significant
Intergenic	chr5 129982733-129982741	TGATGGCGT	1.4594	NA	-4.5890	Significant
PPP2CA	chr5 133536692-133536700	TGTAGGCGA	1.1699	-1.2448	-7.2348	Significant
Intergenic	chr5 134478610-134478618	TCGCCATCA	-1.5305	NA	-4.9165	Significant
Intergenic	chr5 134578306-134578314	TGAAGGCGT	1.2224	NA	-7.8720	Significant
Intergenic	chr5 134765128-134765136	TCGCCAACA	-1.4150	NA	-6.4699	Significant
Intergenic	chr5 135431614-135431622	TCGCCAACA	0.8074	NA	-11.1427	Significant
Intergenic	chr5 135840835-135840843	TCGCCAACA	0.7370	NA	-8.0378	Significant
Intergenic	chr5 135995291-135995299	ACGCCATCA	2.5850	NA	-6.5211	Significant

SPOCK1	chr5 136346436-136346444	ACGCCAACA	2.8074	-0.1184	-12.6134	Significant
SPOCK1	chr5 136557817-136557825	ACGCCATCA	0.4594	-0.1184	-7.2348	Significant
CTNNA1	chr5 138244216-138244224	TGATGGCGT	1.8074	-0.3095	-19.5976	Significant
PCDHA1	chr5 140356903-140356911	TGATGGCGA	0.5146	0.1270	-7.0823	Significant
PCDHA2	chr5 140356903-140356911	TGATGGCGA	0.5146	0.0765	-7.0823	Significant
PCDHA3	chr5 140356903-140356911	TGATGGCGA	1.7370	0.2515	-6.3198	Significant
PCDHA4	chr5 140356903-140356911	TGATGGCGA	5.0000	0.1822	-17.8714	Significant
PCDHA5	chr5 140356903-140356911	TGATGGCGA	-0.2630	0.0362	-7.0772	Significant
PCDHA7	chr5 140356903-140356911	TGATGGCGA	0.0000	-0.0176	-7.4699	Significant
PCDHA8	chr5 140356903-140356911	TGATGGCGA	-0.4854	-0.0952	-6.6939	Significant
PCDHA9	chr5 140356903-140356911	TGATGGCGA	2.5850	-0.0054	-5.5994	Significant
PCDHA10	chr5 140356903-140356911	TGATGGCGA	0.4594	0.0243	-9.3128	Significant
PCDHA11	chr5 140356903-140356911	TGATGGCGA	5.0000	0.0440	-8.2836	Significant
PCDHA12	chr5 140356903-140356911	TGATGGCGA	0.9069	0.1304	-8.8782	Significant
PCDHA13	chr5 140356903-140356911	TGATGGCGA	5.0000	-0.1098	-11.6236	Significant
PCDHAC1	chr5 140356903-140356911	TGATGGCGA	1.8745	0.0720	-7.8717	Significant
PCDHAC2	chr5 140356903-140356911	TGATGGCGA	2.0000	0.0713	-6.8473	Significant
Intergenic	chr5 140545821-140545829	TCGCCTTCA	1.3219	NA	-4.9166	Significant
ARHGAP26	chr5 142327318-142327326	TGAAGGCGA	5.0000	-2.0144	-8.7043	Significant
Intergenic	chr5 143115444-143115452	ACGCCTACA	5.0000	NA	-7.8720	Significant
Intergenic	chr5 144546056-144546064	ACGCCTTCA	-1.5850	NA	-7.2348	Significant
PRELID2	chr5 145184389-145184397	TGATGGCGA	-0.3219	-1.1711	-6.6938	Significant
SH3RF2	chr5 145440562-145440570	TCGCCATCA	5.0000	-1.1562	-6.6938	Significant
PPP2R2B	chr5 146084864-146084872	TCGCCTTCA	5.0000	-0.2299	-9.3124	Significant
PPP2R2B	chr5 146417561-146417569	TGTTGGCGA	5.0000	-0.2299	-5.0497	Significant
DPYSL3	chr5 146818013-146818021	TCGCCTTCA	0.6521	0.0614	-9.7560	Significant
JAKMIP2	chr5 146970331-146970339	TGTTGGCGT	1.5850	-0.0243	-6.0167	Significant
Intergenic	chr5 147621592-147621600	TGATGGCGT	5.0000	NA	-11.3382	Significant
Intergenic	chr5 150155665-150155673	ACGCCATCA	1.8074	NA	-10.9476	Significant
SLC36A3	chr5 150664181-150664189	ACGCCTTCA	0.8745	0.4974	-8.4534	Significant
Intergenic	chr5 153867049-153867057	TCGCCATCA	-0.7655	NA	-9.7560	Significant
Promoter_MRPL22	chr5 154319674-154319682	TGAAGGCGT	1.4150	-0.4377	-10.9247	Significant
SGCD	chr5 155873379-155873387	TGTAGGCGT	-0.7105	0.0681	-5.9550	Significant
ITK	chr5 156632708-156632716	ACGCCTTCA	0.5850	0.0730	-10.0064	Significant
ITK	chr5 156676195-156676203	TGAAGGCGA	5.0000	0.0730	-10.4806	Significant
EBF1	chr5 158128845-158128853	TCGCCTTCA	5.0000	0.2918	-10.0223	Significant
EBF1	chr5 158281750-158281758	ACGCCATCA	1.7162	0.2918	-4.3961	Significant
EBF1	chr5 158343085-158343092	TGTTGGCGT	1.7004	0.2918	-8.8782	Significant
Intergenic	chr5 158821865-158821873	TGATGGCGT	2.3219	NA	-6.4691	Significant
Intergenic	chr5 158971420-158971428	TGTTGGCGT	1.2224	NA	-7.6831	Significant
GABRB2	chr5 160826936-160826944	TGATGGCGA	5.0000	0.1757	-8.7041	Significant
Intergenic	chr5 161236310-161236318	TGAAGGCGT	1.5850	NA	-5.9550	Significant
Intergenic	chr5 161705992-161706000	TGAAGGCGA	2.5850	NA	-5.7407	Significant
Intergenic	chr5 162096921-162096929	ACGCCTACA	1.2630	NA	-13.4241	Significant
Intergenic	chr5 163220597-163220605	TGATGGCGT	3.5850	NA	-6.6938	Significant
Intergenic	chr5 164267201-164267209	TCGCCATCA	2.4594	NA	-8.2834	Significant
Intergenic	chr5 164761430-164761438	TGTAGGCGT	2.3219	NA	-5.9549	Significant
Intergenic	chr5 166259762-166259770	TGATGGCGA	1.1375	NA	-9.1342	Significant
Intergenic	chr5 167029411-167029419	ACGCCTTCA	5.0000	NA	-7.2864	Significant
Intergenic	chr5 167292171-167292179	ACGCCTTCA	1.7370	NA	-7.6831	Significant
Intergenic	chr5 168082393-168082401	TGAAGGCGA	-0.4854	NA	-6.8992	Significant
SLIT3	chr5 168371414-168371422	TGAAGGCGT	1.1699	0.1085	-6.1002	Significant
Intergenic	chr5 168757511-168757519	TCGCCTACA	3.0000	NA	-4.2708	Marginal
KCNIP1	chr5 169986334-169986342	TCGCCTACA	2.3219	-0.0947	-12.6134	Significant
Intergenic	chr5 171173472-171173480	TGAAGGCGT	1.7004	NA	-9.7560	Significant
Intergenic	chr5 172827347-172827355	TGTAGGCGA	0.6781	NA	-5.9550	Significant
Intergenic	chr5 174150280-174150288	TGTAGGCGA	3.3219	NA	-6.6938	Significant
UIMC1	chr5 176381234-176381242	ACGCCATCA	5.0000	-3.0093	-9.7560	Significant
NSD1	chr5 176674239-176674247	TCGCCAACA	-1.5406	-3.0718	-6.1522	Significant
CLK4	chr5 178051880-178051888	ACGCCATCA	1.2224	-1.6432	-13.1227	Significant
RNF130	chr5 179444261-179444269	TGATGGCGT	2.5850	-0.2096	-5.9017	Significant
EXOC2	chr6 625562-625570	TGAAGGCGT	5.0000	-0.8550	-5.1011	Significant
Intergenic	chr6 770558-770566	TCGCCTACA	1.1699	NA	-5.2533	Significant
GMDS	chr6 1636526-1636534	TGTTGGCGT	3.3219	-0.0553	-7.6831	Significant
GMDS	chr6 1734573-1734581	ACGCCTACA	1.6781	-0.0553	-8.0891	Significant

GMDS	chr6 1865273-1865281	ACGCCATCA	3.7004	-0.0553	-15.2922	Significant
GMDS	chr6 2004463-2004471	ACGCCTACA	0.0000	-0.0553	-5.7415	Significant
Intergenic	chr6 2498570-2498578	TGAAGGCGT	5.0000	NA	-10.4609	Significant
SERPINB9	chr6 2900028-2900036	TGAAGGCGT	5.0000	-0.0540	-8.0382	Significant
SLC22A23	chr6 3326128-3326136	TGATGGCGT	5.0000	-0.6563	-10.4803	Significant
Intergenic	chr6 6845007-6845015	ACGCCATCA	1.7004	NA	-6.4691	Significant
Intergenic	chr6 6966923-6966931	TGATGGCGA	1.2630	NA	-11.9104	Significant
Intergenic	chr6 7489672-7489680	ACGCCAACA	1.3785	NA	-14.7050	Significant
Intergenic	chr6 7683887-7683895	TGTTGGCGA	5.0000	NA	-7.2864	Significant
Intergenic	chr6 8036636-8036644	ACGCCAACA	-0.4406	NA	-8.4534	Significant
Intergenic	chr6 8398775-8398783	ACGCCTACA	5.0000	NA	-6.1520	Significant
LOC100506207	chr6 8747042-8747050	TGAAGGCGT	3.4594	0.0127	-21.8765	Significant
Intergenic	chr6 10004560-10004568	TGAAGGCGA	3.1699	NA	-6.5209	Significant
Intergenic	chr6 11132507-11132515	TGTTGGCGA	5.0000	NA	-13.6405	Significant
NEDD9	chr6 11303701-11303709	TCGCCATCA	-1.8260	-0.5629	-6.1520	Significant
Intergenic	chr6 11473012-11473020	TGTTGGCGA	0.6521	NA	-6.6938	Significant
Intergenic	chr6 11922227-11922235	TGTTGGCGT	5.0000	NA	-5.2533	Significant
PHACTR1	chr6 12851618-12851626	TGATGGCGT	5.0000	0.4214	-10.0223	Significant
PHACTR1	chr6 13237471-13237479	TGATGGCGT	1.0000	0.4214	-5.3906	Significant
PHACTR1	chr6 13273964-13273972	TCGCCAACA	1.7004	0.4214	-6.1006	Significant
Intergenic	chr6 13342406-13342414	ACGCCAACA	1.8745	NA	-8.0378	Significant
GFOD1	chr6 13486000-13486008	TGAAGGCGA	5.0000	-2.6247	-16.4103	Significant
RANBP9	chr6 13655566-13655574	TGATGGCGT	-0.1520	-1.9173	-5.3906	Significant
Intergenic	chr6 13857952-13857960	TGTTGGCGT	3.9069	NA	-19.6929	Significant
RNF182	chr6 13961291-13961299	TGTTGGCGT	3.8074	0.1444	-5.3906	Significant
Intergenic	chr6 14087132-14087140	TGATGGCGA	5.0000	NA	-5.0497	Significant
Intergenic	chr6 14282150-14282158	TGTAGGCGA	5.0000	NA	-6.9960	Significant
ATXN1	chr6 16513339-16513347	ACGCCAACA	0.4594	-1.2637	-9.3124	Significant
ATXN1	chr6 16675757-16675765	TGAAGGCGA	5.0000	-1.2637	-8.2834	Significant
Intergenic	chr6 17379311-17379319	TCGCCATCA	-0.1699	NA	-11.4244	Significant
Intergenic	chr6 19635877-19635885	TGATGGCGT	4.1699	NA	-14.1681	Significant
E2F3	chr6 20438202-20438210	TGATGGCGA	1.3219	-4.3333	-5.3906	Significant
CDKAL1	chr6 20770916-20770924	TGAAGGCGT	1.2630	-0.9919	-6.4691	Significant
CDKAL1	chr6 20927492-20927499	TGAAGGCGA	3.3219	-0.9919	-9.3124	Significant
CDKAL1	chr6 21067583-21067591	TGTTGGCGA	2.4594	-0.9919	-9.1342	Significant
Intergenic	chr6 21335084-21335092	TGAAGGCGT	3.3219	NA	-10.2581	Significant
Intergenic	chr6 21409900-21409908	TGTTGGCGA	0.8074	NA	-11.1431	Significant
ACOT13	chr6 24697623-24697631	TGTTGGCGT	2.0000	-0.2447	-5.5996	Significant
ACOT13	chr6 24697797-24697805	TGTTGGCGT	0.3626	-0.2447	-6.4691	Significant
SLC17A1	chr6 25819064-25819072	TCGCCTACA	5.0000	0.1250	-14.4800	Significant
Intergenic	chr6 28694218-28694226	TGTAGGCGT	5.0000	NA	-6.1520	Significant
MUC22	chr6 30979615-30979623	ACGCCAACA	3.5850	0.2267	-8.6982	Significant
Intergenic	chr6 31300314-31300322	TCGCCTTCA	2.0000	NA	-7.2348	Significant
Intergenic	chr6 32245830-32245838	TGAAGGCGA	3.4594	NA	-9.3124	Significant
Intergenic	chr6 32415674-32415682	ACGCCTTCA	1.8745	NA	-10.9478	Significant
Intergenic	chr6 33094823-33094831	TGTAGGCGA	0.2801	NA	-7.8720	Significant
Intergenic	chr6 33877797-33877805	TCGCCTTCA	5.0000	NA	-6.4866	Significant
Intergenic	chr6 33948806-33948814	ACGCCATCA	-0.9260	NA	-8.0378	Significant
C6orf106	chr6 34573522-34573530	ACGCCAACA	1.4150	-0.9549	-8.8782	Significant
C6orf106	chr6 34595310-34595318	TGTTGGCGT	0.1926	-0.9549	-10.9478	Significant
C6orf106	chr6 34636419-34636427	ACGCCTTCA	1.1699	-0.9549	-6.1002	Significant
Intergenic	chr6 34751002-34751010	TGTTGGCGA	0.7776	NA	-6.6938	Significant
FKBP5	chr6 35544803-35544811	TGTTGGCGT	0.4150	-0.5971	-5.7673	Significant
FKBP5	chr6 35587588-35587596	TCGCCAACA	2.2224	-0.5971	-9.8060	Significant
PNPLA1	chr6 36254883-36254891	TCGCCTTCA	5.0000	-0.2517	-7.0772	Significant
TBC1D22B	chr6 37269557-37269565	TGTTGGCGT	0.8745	-2.1133	-10.9476	Significant
BTBD9	chr6 38380497-38380505	TGATGGCGT	5.0000	-0.9290	-5.7407	Significant
USP49	chr6 41800451-41800459	TCGCCTACA	0.7370	-0.1620	-8.0891	Significant
Intergenic	chr6 43811986-43811994	TGTTGGCGA	5.0000	NA	-11.0946	Significant
SUPT3H	chr6 45052206-45052214	TGTTGGCGA	0.5850	-0.2445	-5.1015	Significant
RUNX2	chr6 45481563-45481571	TCGCCTTCA	1.7370	-0.4190	-6.6941	Significant
Intergenic	chr6 46066324-46066332	TGTTGGCGT	5.0000	NA	-13.1227	Significant
Intergenic	chr6 46943481-46943489	TGTTGGCGA	2.3219	NA	-7.4701	Significant
TNFRSF21	chr6 47204502-47204510	ACGCCAACA	1.0000	-2.2794	-6.5211	Significant
Intergenic	chr6 48913180-48913188	ACGCCAACA	5.0000	NA	-9.8058	Significant

Intergenic	chr6 50187210-50187218	ACGCCTTCA	2.2224	NA	-5.2536	Significant
PKHD1	chr6 51543566-51543574	TCGCCATCA	5.0000	0.1237	-6.1002	Significant
PAQR8	chr6 52245464-52245472	ACGCCTTCA	3.3219	-0.8559	-11.9103	Significant
MLIP	chr6 53892657-53892665	TGATGGCGA	5.0000	0.2776	-7.6316	Significant
MLIP	chr6 54083464-54083472	ACGCCATCA	0.0000	0.2776	-5.3906	Significant
Intergenic	chr6 54428922-54428930	ACGCCTACA	3.7004	NA	-6.3198	Significant
GFRAL	chr6 55253099-55253107	TGAAGGCGA	0.6781	0.2426	-8.2802	Significant
Intergenic	chr6 56234986-56234994	TCGCCTACA	2.1699	NA	-5.4421	Significant
DST	chr6 56571852-56571860	ACGCCATCA	5.0000	-0.3896	-7.4724	Significant
DST	chr6 56761408-56761416	ACGCCAACA	1.4150	-0.3896	-9.7560	Significant
ZNF451	chr6 56968720-56968728	TGATGGCGT	2.1699	-1.5497	-5.4421	Significant
Intergenic	chr6 57581331-57581338	TCGCCAACA	3.1699	NA	-4.2708	Marginal
Intergenic	chr6 57869435-57869443	ACGCCAACA	5.0000	NA	-4.5890	Significant
Intergenic	chr6 58427456-58427464	ACGCCTTCA	3.5850	NA	-6.6938	Significant
Intergenic	chr6 63374751-63374759	ACGCCAACA	1.1375	NA	-5.9549	Significant
EYS	chr6 64524158-64524166	TCGCCAACA	1.2630	0.1338	-7.4701	Significant
Intergenic	chr6 67411891-67411899	TCGCCTTCA	3.0000	NA	-7.2424	Significant
Intergenic	chr6 68095137-68095145	ACGCCTTCA	5.0000	NA	-11.1427	Significant
Intergenic	chr6 68489764-68489772	TGTTGGCGA	5.0000	NA	-11.6709	Significant
COL19A1	chr6 70836650-70836658	TGAAGGCGT	1.5850	0.2868	-5.7924	Significant
FAM135A	chr6 71146307-71146315	TGTTGGCGA	0.6374	-2.0117	-10.2583	Significant
SMAP1	chr6 71518493-71518501	TCGCCTACA	5.0000	-0.3433	-20.3187	Significant
Intergenic	chr6 71803039-71803047	ACGCCTACA	1.1375	NA	-6.1006	Significant
RIMS1	chr6 72606523-72606531	TGTTGGCGT	4.0000	0.1178	-13.1223	Significant
Intergenic	chr6 74981067-74981075	TCGCCTTCA	-1.0000	NA	-6.6941	Significant
Intergenic	chr6 75263618-75263626	ACGCCTTCA	5.0000	NA	-5.0497	Significant
Intergenic	chr6 75272585-75272593	TCGCCATCA	-0.2065	NA	-5.3906	Significant
MYO6	chr6 76540140-76540148	TCGCCAACA	2.5850	-0.2213	-8.6980	Significant
Intergenic	chr6 76929334-76929342	ACGCCAACA	4.0000	NA	-14.7469	Significant
Intergenic	chr6 78178903-78178911	ACGCCTTCA	5.0000	NA	-13.9474	Significant
Intergenic	chr6 79978937-79978945	TGTAGGCGA	-0.1375	NA	-5.1013	Significant
Intergenic	chr6 80421732-80421740	TCGCCAACA	5.0000	NA	-8.7041	Significant
ELOVL4	chr6 80639105-80639113	ACGCCAACA	5.0000	0.1725	-10.0223	Significant
Intergenic	chr6 81229710-81229718	ACGCCATCA	0.4475	NA	-8.4538	Significant
Intergenic	chr6 82358488-82358496	TCGCCTACA	-1.4288	NA	-9.3627	Significant
CYB5R4	chr6 84617848-84617856	TCGCCAACA	2.1699	-0.1324	-7.4699	Significant
Intergenic	chr6 85644896-85644904	TCGCCAACA	3.4594	NA	-6.1002	Significant
SYNCRIP	chr6 86331631-86331639	TGTAGGCGA	2.1699	-0.6873	-7.6316	Significant
Intergenic	chr6 88450783-88450790	ACGCCATCA	2.2224	NA	-7.6320	Significant
Intergenic	chr6 88898822-88898830	ACGCCTACA	2.7004	NA	-8.7041	Significant
RNGTT	chr6 89534420-89534428	ACGCCAACA	0.5146	-2.7998	-6.3196	Significant
RNGTT	chr6 89572057-89572065	ACGCCTACA	1.3785	-2.7998	-7.4701	Significant
RNGTT	chr6 89626142-89626150	TGATGGCGT	5.0000	-2.7998	-20.5879	Significant
ANKRD6	chr6 90197384-90197392	TCGCCATCA	3.0000	0.0945	-6.4691	Significant
BACH2	chr6 90930419-90930427	TGATGGCGT	1.0000	0.0538	-8.0891	Significant
Intergenic	chr6 92219924-92219932	ACGCCAACA	5.0000	NA	-8.2834	Significant
Intergenic	chr6 95379676-95379683	TGAAGGCGA	0.3219	NA	-6.1002	Significant
Intergenic	chr6 95379682-95379683	TGAAGGCGA	5.0000	NA	-8.2834	Significant
Intergenic	chr6 97064767-97064775	TGTAGGCGA	0.3626	NA	-5.5996	Significant
Intergenic	chr6 98738930-98738938	TCGCCTACA	-0.3626	NA	-6.3196	Significant
GRIK2	chr6 102224867-102224875	TCGCCATCA	0.8301	0.1168	-5.7411	Significant
Intergenic	chr6 102698870-102698878	TCGCCAACA	3.8074	NA	-9.3128	Significant
Intergenic	chr6 104857707-104857715	TGAAGGCGA	5.0000	NA	-6.8990	Significant
Intergenic	chr6 104978688-104978696	ACGCCTTCA	3.1699	NA	-4.3969	Significant
Intergenic	chr6 105688942-105688950	TGTTGGCGT	-0.3626	NA	-10.0224	Significant
Intergenic	chr6 106388907-106388915	TCGCCTTCA	5.0000	NA	-9.3128	Significant
PRDM1	chr6 106543584-106543592	ACGCCAACA	3.0000	0.6803	-8.5043	Significant
PDSS2	chr6 107609484-107609492	ACGCCTTCA	5.0000	-1.1259	-7.0774	Significant
PDSS2	chr6 107641261-107641269	TCGCCTTCA	5.0000	-1.1259	-7.0772	Significant
SOBP	chr6 107872671-107872679	TGATGGCGT	5.0000	0.1026	-8.8782	Significant
SCML4	chr6 108109953-108109961	TCGCCATCA	5.0000	0.2332	-10.4803	Significant
ARMC2	chr6 109226422-109226430	TGTTGGCGT	5.0000	0.0470	-6.1002	Significant
Intergenic	chr6 110672491-110672499	TGTAGGCGT	2.5850	NA	-9.1347	Significant
Intergenic	chr6 110680568-110680575	ACGCCTACA	5.0000	NA	-9.5737	Significant
Intergenic	chr6 112327488-112327496	TGTAGGCGT	0.1699	NA	-6.4699	Significant

Intergenic	chr6 112823969-112823977	TGATGGCGT	2.8074	NA	-13.1671	Significant
Intergenic	chr6 113065993-113066001	TGTTGGCGA	5.0000	NA	-4.4469	Significant
Intergenic	chr6 113101966-113101974	ACGCCTTCA	5.0000	NA	-6.6938	Significant
Intergenic	chr6 113878544-113878552	TGATGGCGA	5.0000	NA	-14.4802	Significant
Intergenic	chr6 115378752-115378760	ACGCCTACA	2.0000	NA	-5.3906	Significant
Intergenic	chr6 115697076-115697084	ACGCCTTCA	2.8074	NA	-5.3910	Significant
Intergenic	chr6 116072486-116072494	TCGCCATCA	1.5850	NA	-6.8992	Significant
Intergenic	chr6 116917897-116917905	ACGCCTACA	2.8074	NA	-9.7560	Significant
Intergenic	chr6 116918094-116918102	ACGCCAACA	3.3219	NA	-6.3196	Significant
ROS1	chr6 117618887-117618895	TGTTGGCGA	5.0000	-0.0218	-12.9102	Significant
Intergenic	chr6 118107314-118107322	ACGCCTACA	-0.5475	NA	-4.9166	Significant
Intergenic	chr6 118830935-118830943	ACGCCAACA	5.0000	NA	-21.2279	Significant
Intergenic	chr6 122334903-122334911	TGTTGGCGT	3.3219	NA	-7.4727	Significant
Intergenic	chr6 122443182-122443190	ACGCCATCA	0.5406	NA	-5.9550	Significant
Intergenic	chr6 122606257-122606265	TCGCCTACA	2.8074	NA	-7.0774	Significant
TPD52L1	chr6 125555090-125555098	TGTTGGCGT	5.0000	-0.2745	-5.0497	Significant
Intergenic	chr6 125662587-125662595	ACGCCTTCA	0.1520	NA	-4.7186	Significant
Intergenic	chr6 126367202-126367210	TGAAGGCGA	5.0000	NA	-8.7041	Significant
Intergenic	chr6 126386522-126386530	ACGCCTTCA	4.0000	NA	-6.1520	Significant
Intergenic	chr6 127886681-127886689	TGATGGCGA	3.1699	NA	-5.4421	Significant
THEMIS	chr6 128178238-128178246	TGTTGGCGA	1.1375	0.3412	-4.3961	Significant
Intergenic	chr6 128882043-128882051	TGAAGGCGA	1.4150	NA	-6.1014	Significant
Intergenic	chr6 130294930-130294938	TCGCCATCA	-0.8301	NA	-5.1015	Significant
Intergenic	chr6 131156038-131156046	TCGCCAACA	5.0000	NA	-6.6938	Significant
Intergenic	chr6 132439044-132439052	TGTAGGCGT	5.0000	NA	-7.8720	Significant
Intergenic	chr6 132559818-132559826	ACGCCTTCA	0.8074	NA	-9.1344	Significant
Intergenic	chr6 133214879-133214887	TCGCCTACA	5.0000	NA	-6.8599	Significant
EYA4	chr6 133845401-133845409	TGTTGGCGA	2.5850	-0.0487	-6.6938	Significant
Intergenic	chr6 134419827-134419835	ACGCCTTCA	2.3219	NA	-6.7370	Significant
Intergenic	chr6 135133111-135133119	TGTTGGCGT	3.4594	NA	-10.4803	Significant
MAP3K5	chr6 137109893-137109901	TGTTGGCGA	2.5850	-3.2973	-6.6938	Significant
Intergenic	chr6 137502250-137502258	TGAAGGCGA	3.0000	NA	-13.6851	Significant
Intergenic	chr6 137654091-137654099	TGTTGGCGA	1.0000	NA	-8.2802	Significant
ARFGEF3	chr6 138657273-138657281	ACGCCTACA	3.7004	-0.6744	-8.7043	Significant
NHSL1	chr6 138769327-138769335	TGTTGGCGA	0.4150	-1.3530	-10.0223	Significant
LOC100132735	chr6 140139381-140139389	TCGCCTTCA	0.8745	0.1642	-8.2834	Significant
Intergenic	chr6 142192921-142192929	TCGCCATCA	1.1699	NA	-8.8782	Significant
Intergenic	chr6 142810834-142810842	TGTTGGCGT	5.0000	NA	-16.4101	Significant
LOC153910	chr6 142893409-142893416	TCGCCTTCA	5.0000	0.1558	-12.0511	Significant
LOC153910	chr6 142893409-142893415	TCGCCTTCA	1.4150	0.1558	-9.5396	Significant
Intergenic	chr6 142997346-142997354	TGATGGCGA	5.0000	NA	-9.8058	Significant
HIVEP2	chr6 143098077-143098085	TGATGGCGT	2.5850	-3.6763	-11.6240	Significant
AIG1	chr6 143649628-143649636	ACGCCTACA	5.0000	0.2895	-10.9476	Significant
UTRN	chr6 145115160-145115168	TGTTGGCGA	1.8745	-2.6579	-17.5297	Significant
Intergenic	chr6 147054959-147054967	TGAAGGCGT	1.8745	NA	-11.1427	Significant
SAMD5	chr6 147879062-147879070	TCGCCATCA	2.0000	-0.5021	-7.6316	Significant
Intergenic	chr6 148490630-148490638	TCGCCTTCA	5.0000	NA	-7.4699	Significant
SASH1	chr6 148779120-148779128	TGTTGGCGA	1.4150	-2.3245	-6.3302	Significant
Intergenic	chr6 149477546-149477554	TCGCCAACA	3.7549	NA	-4.7182	Significant
TAB2	chr6 149577958-149577966	TCGCCTACA	5.0000	-2.7033	-6.1002	Significant
Intergenic	chr6 150252987-150252995	TGATGGCGA	0.3479	NA	-10.2093	Significant
ESR1	chr6 152116024-152116032	ACGCCTTCA	5.0000	0.1974	-9.1342	Significant
ESR1	chr6 152161134-152161142	TGTAGGCGT	1.1699	0.1974	-7.6339	Significant
ESR1	chr6 152368207-152368215	ACGCCTTCA	2.1699	0.1974	-7.6833	Significant
OPRM1	chr6 154401434-154401442	ACGCCTTCA	-1.6781	0.1585	-5.0497	Significant
Intergenic	chr6 154986260-154986268	TGTTGGCGA	2.4594	NA	-7.0775	Significant
TIAM2	chr6 155538832-155538840	TGTAGGCGT	3.0000	-1.1867	-6.3200	Significant
Intergenic	chr6 155843613-155843621	TCGCCAACA	5.0000	NA	-8.4534	Significant
Intergenic	chr6 155852379-155852387	TGATGGCGA	-0.2224	NA	-13.6409	Significant
Intergenic	chr6 156462472-156462480	TCGCCATCA	5.0000	NA	-7.4724	Significant
Intergenic	chr6 157050505-157050513	TCGCCAACA	5.0000	NA	-10.0223	Significant
ARID1B	chr6 157106041-157106049	ACGCCATCA	5.0000	-2.2010	-7.8720	Significant
EZR	chr6 159225137-159225145	TGATGGCGT	3.1699	-0.5953	-7.8720	Significant
Intergenic	chr6 159826604-159826612	TGTTGGCGT	0.8480	NA	-10.6711	Significant
Intergenic	chr6 160377803-160377811	TCGCCATCA	5.0000	NA	-14.1681	Significant

MAP3K4	chr6 161485863-161485871	ACGCCTACA	0.2895	-2.4536	-4.7186	Significant
PARK2	chr6 161843928-161843936	ACGCCAACA	5.0000	0.2156	-10.9478	Significant
PARK2	chr6 162564861-162564868	TGAAGGCGA	5.0000	0.2156	-12.1603	Significant
PARK2	chr6 162915987-162915995	TGATGGCGA	1.3219	0.2156	-8.2834	Significant
Intergenic	chr6 164208724-164208732	TGATGGCGA	5.0000	NA	-4.3965	Significant
C6orf118	chr6 165701120-165701128	ACGCCTACA	3.1699	0.0388	-7.6831	Significant
PDE10A	chr6 165802590-165802598	TGAAGGCGA	5.0000	-0.1931	-11.9103	Significant
Intergenic	chr6 167784799-167784807	TCGCCTACA	0.5850	NA	-4.7182	Significant
KIF25	chr6 168431334-168431342	TGATGGCGT	5.0000	0.6617	-8.2844	Significant
THBS2	chr6 169625964-169625972	TGAAGGCGT	0.5850	0.2098	-6.5209	Significant
MAD1L1	chr7 1966309-1966317	ACGCCAACA	0.3219	-1.8725	-5.3906	Significant
CARD11	chr7 2992928-2992936	ACGCCTACA	1.0000	0.1794	-4.5890	Significant
SDK1	chr7 3838668-3838676	TCGCCTTCA	4.1699	0.0780	-6.1002	Significant
SDK1	chr7 4086740-4086748	TGTAGGCGT	-0.7370	0.0780	-6.8473	Significant
RNF216	chr7 5699047-5699054	TGAAGGCGT	3.5850	-2.3834	-7.2348	Significant
RNF216	chr7 5800338-5800346	ACGCCTTCA	2.4594	-2.3834	-10.9476	Significant
USP42	chr7 6146741-6146749	TGAAGGCGA	3.4594	-3.4481	-10.9476	Significant
Intergenic	chr7 7217878-7217886	TGTTGGCGA	5.0000	NA	-8.8782	Significant
COL28A1	chr7 7481690-7481698	TCGCCAACA	5.0000	0.2197	-4.5890	Significant
Intergenic	chr7 7935460-7935468	TGAAGGCGT	5.0000	NA	-7.0772	Significant
Intergenic	chr7 7949031-7949039	TGATGGCGA	2.3219	NA	-5.9672	Significant
Intergenic	chr7 8875417-8875425	TGAAGGCGA	5.0000	NA	-4.5890	Significant
Intergenic	chr7 9729314-9729322	TGTAGGCGT	3.4594	NA	-7.6831	Significant
Intergenic	chr7 10903289-10903297	ACGCCATCA	5.0000	NA	-5.7926	Significant
Intergenic	chr7 12283764-12283772	TCGCCTACA	5.0000	NA	-5.9550	Significant
Intergenic	chr7 12997325-12997333	ACGCCATCA	1.9069	NA	-6.6938	Significant
ETV1	chr7 13942814-13942822	ACGCCTTCA	1.1699	-0.2976	-9.3128	Significant
DGKB	chr7 14529656-14529664	ACGCCAACA	2.3219	0.1624	-8.8790	Significant
Intergenic	chr7 16991700-16991708	TGATGGCGT	1.9069	NA	-7.6324	Significant
Intergenic	chr7 17111773-17111781	TCGCCTACA	5.0000	NA	-12.4057	Significant
HDAC9	chr7 18938132-18938140	TGAAGGCGT	3.1699	0.2105	-8.0378	Significant
Intergenic	chr7 20162313-20162321	TCGCCTTCA	2.1699	NA	-4.5891	Significant
MACC1	chr7 20216497-20216505	TGATGGCGA	2.0000	-1.1014	-6.8473	Significant
Intergenic	chr7 20336345-20336353	TCGCCAACA	2.3219	NA	-9.7560	Significant
Intergenic	chr7 22017545-22017553	TGTTGGCGA	5.0000	NA	-8.8782	Significant
Intergenic	chr7 22647577-22647585	TGAAGGCGT	0.8480	NA	-8.9289	Significant
Intergenic	chr7 25709180-25709188	ACGCCTTCA	3.3219	NA	-4.5891	Significant
LOC441204	chr7 26480086-26480094	TGATGGCGT	5.0000	0.3459	-5.0497	Significant
SKAP2	chr7 26877743-26877751	ACGCCTTCA	5.0000	0.1054	-8.9289	Significant
JAZF1	chr7 27980711-27980719	ACGCCTTCA	5.0000	0.1000	-6.5209	Significant
JAZF1	chr7 28030438-28030446	TGAAGGCGT	1.0000	0.1000	-10.9476	Significant
JAZF1	chr7 28201518-28201526	TCGCCTACA	2.3219	0.1000	-7.0772	Significant
Intergenic	chr7 28246167-28246175	ACGCCTACA	5.0000	NA	-7.2348	Significant
SCRN1	chr7 30016599-30016607	TGAAGGCGT	3.7004	-0.6410	-10.0223	Significant
Intergenic	chr7 31023081-31023089	TCGCCTACA	0.2630	NA	-4.2708	Marginal
PDE1C	chr7 31929674-31929682	TGTAGGCGA	1.7370	0.0983	-8.0378	Significant
Intergenic	chr7 32941888-32941896	TCGCCAACA	3.9069	NA	-12.4056	Significant
BBS9	chr7 33253738-33253746	TGATGGCGA	0.5146	-0.0481	-8.7041	Significant
BBS9	chr7 33567827-33567835	TCGCCTTCA	5.0000	-0.0481	-8.2802	Significant
BBS9	chr7 33631475-33631483	TCGCCATCA	2.3219	-0.0481	-7.0774	Significant
Intergenic	chr7 33821438-33821446	TCGCCATCA	0.9069	NA	-5.2536	Significant
Intergenic	chr7 33936769-33936777	TCGCCTTCA	-0.8074	NA	-4.9166	Significant
Intergenic	chr7 35111710-35111718	TCGCCTTCA	0.4594	NA	-7.2348	Significant
DPY19L2P1	chr7 35125447-35125455	TGATGGCGT	0.3219	-0.1466	-5.2533	Significant
DPY19L2P1	chr7 35223055-35223063	TGATGGCGA	0.5850	-0.1466	-8.8786	Significant
Intergenic	chr7 36031913-36031921	TGAAGGCGT	-0.9260	NA	-18.7250	Significant
AOAH	chr7 36562989-36562996	TGTTGGCGT	5.0000	-0.2065	-9.5736	Significant
AOAH	chr7 36627582-36627590	TCGCCTACA	-0.5850	-0.2065	-8.8782	Significant
ELMO1	chr7 37048437-37048445	TGATGGCGA	-1.5146	0.0327	-6.1002	Significant
Intergenic	chr7 37708952-37708960	TGATGGCGT	2.4594	NA	-12.1603	Significant
Intergenic	chr7 37934581-37934589	TCGCCAACA	-0.4475	NA	-5.5996	Significant
Intergenic	chr7 38973397-38973405	TGATGGCGT	5.0000	NA	-4.7693	Significant
RALA	chr7 39711498-39711506	TGATGGCGT	5.0000	-1.1760	-8.2834	Significant
Intergenic	chr7 40336999-40337007	TGATGGCGT	3.3219	NA	-6.4691	Significant
Intergenic	chr7 40802617-40802625	TGTTGGCGA	2.8074	NA	-16.3718	Significant

Intergenic	chr7 40952879-40952887	ACGCCATCA	0.6781	NA	-5.0847	Significant
Intergenic	chr7 41706037-41706045	TGATGGCGT	-1.0000	NA	-9.5736	Significant
Intergenic	chr7 41716248-41716256	TGATGGCGA	5.0000	NA	-4.5891	Significant
Intergenic	chr7 41937267-41937275	TCGCCAACA	3.1699	NA	-5.0497	Significant
HECW1	chr7 43442686-43442694	TGTTGGCGT	-0.2224	0.2087	-6.6938	Significant
Intergenic	chr7 43773240-43773248	TGATGGCGT	0.6781	NA	-6.4691	Significant
URGCP	chr7 43920882-43920890	TGAAGGCGT	1.1375	-1.6667	-6.8990	Significant
Intergenic	chr7 45810175-45810183	TGTTGGCGA	5.0000	NA	-9.3124	Significant
ABCA13	chr7 48329564-48329572	TCGCCAACA	-0.7776	0.0656	-4.7182	Significant
ABCA13	chr7 48620847-48620855	TCGCCTTCA	3.0000	0.0656	-4.5891	Significant
VWC2	chr7 49947865-49947873	ACGCCTACA	3.7004	0.1371	-8.2834	Significant
COBL	chr7 51364111-51364119	TGAAGGCGT	5.0000	-0.9320	-7.8720	Significant
Intergenic	chr7 51443056-51443064	TCGCCATCA	2.3219	NA	-4.7182	Significant
Intergenic	chr7 51936474-51936482	ACGCCATCA	3.0000	NA	-6.8477	Significant
Intergenic	chr7 52194040-52194048	TCGCCTTCA	3.3219	NA	-8.7041	Significant
Intergenic	chr7 53269889-53269897	TGATGGCGT	3.5850	NA	-7.2348	Significant
Intergenic	chr7 54032359-54032367	TCGCCTACA	0.4854	NA	-5.3910	Significant
Intergenic	chr7 54407420-54407428	TGTTGGCGT	5.0000	NA	-5.4421	Significant
Intergenic	chr7 57231578-57231586	TGTAGGCGT	5.0000	NA	-6.8990	Significant
Intergenic	chr7 57696603-57696611	TGATGGCGA	2.1699	NA	-9.3627	Significant
Intergenic	chr7 57696642-57696650	TGATGGCGA	1.2224	NA	-5.3906	Significant
Intergenic	chr7 57888303-57888311	TCGCCATCA	5.0000	NA	-13.6844	Significant
Intergenic	chr7 57888342-57888350	TCGCCATCA	-0.0995	NA	-5.5994	Significant
Intergenic	chr7 64960955-64960963	ACGCCTACA	2.3219	NA	-6.8473	Significant
Intergenic	chr7 65472200-65472208	TGTAGGCGT	5.0000	NA	-11.9103	Significant
Intergenic	chr7 65875848-65875856	ACGCCTTCA	3.5850	NA	-6.1002	Significant
LOC493754	chr7 66050974-66050982	ACGCCATCA	0.2065	NA	-5.3910	Significant
Intergenic	chr7 66357722-66357730	TGTAGGCGT	0.4854	NA	-21.5984	Significant
Intergenic	chr7 68520504-68520512	TGTTGGCGT	3.3219	NA	-10.0223	Significant
Intergenic	chr7 68690865-68690873	TGTAGGCGT	0.5146	NA	-7.4724	Significant
Intergenic	chr7 69040286-69040294	ACGCCTTCA	5.0000	NA	-11.3979	Significant
AUTS2	chr7 69115461-69115469	TGAAGGCGT	0.1375	-1.9713	-9.7560	Significant
AUTS2	chr7 69340628-69340636	TGTTGGCGA	1.1375	-1.9713	-6.6939	Significant
CALN1	chr7 71794059-71794067	ACGCCAACA	5.0000	0.2997	-11.9103	Significant
GTF2I	chr7 74121328-74121336	ACGCCATCA	2.4150	-0.6104	-7.8722	Significant
Intergenic	chr7 75755125-75755133	TGAAGGCGT	4.0875	NA	-12.1138	Significant
Intergenic	chr7 76430180-76430188	TGTTGGCGA	2.8074	NA	-7.8720	Significant
CCDC146	chr7 76909772-76909780	ACGCCAACA	3.5850	-0.5928	-18.7254	Significant
Intergenic	chr7 77412496-77412504	ACGCCAACA	0.1069	NA	-6.8473	Significant
MAGI2	chr7 78171516-78171524	TGTTGGCGA	-0.5850	-0.0566	-7.4703	Significant
MAGI2	chr7 78987503-78987511	ACGCCAACA	1.1375	-0.0566	-6.6938	Significant
MAGI2	chr7 79046874-79046882	TGTAGGCGT	1.6781	-0.0566	-6.7016	Significant
MAGI2-AS3	chr7 79084452-79084460	TGAAGGCGA	-0.2630	0.0459	-4.5891	Significant
Intergenic	chr7 79447159-79447167	TGAAGGCGA	5.0000	NA	-19.6931	Significant
GNAI1	chr7 79778595-79778603	TCGCCTACA	-1.3219	-2.1911	-8.9291	Significant
Intergenic	chr7 83417677-83417685	TGATGGCGT	0.5850	NA	-7.2348	Significant
SEMA3A	chr7 83637057-83637065	ACGCCTTCA	5.0000	-1.0900	-4.6093	Significant
SEMA3A	chr7 83650700-83650708	TCGCCTTCA	5.0000	-1.0900	-8.7041	Significant
Intergenic	chr7 84119041-84119049	TGTTGGCGT	3.0000	NA	-4.9165	Significant
SEMA3D	chr7 84742401-84742409	TGATGGCGA	5.0000	0.2851	-9.7560	Significant
ABCB1	chr7 87270594-87270602	TCGCCAACA	5.0000	-0.2433	-6.6938	Significant
RUNDC3B	chr7 87270594-87270602	TCGCCAACA	3.4594	-0.4580	-5.2699	Significant
Intergenic	chr7 88381499-88381507	TGTTGGCGA	1.7004	NA	-8.5043	Significant
Intergenic	chr7 89185311-89185319	TGAAGGCGA	0.4150	NA	-5.9675	Significant
Intergenic	chr7 89347098-89347106	TCGCCTTCA	5.0000	NA	-14.1681	Significant
STEAP1	chr7 89793277-89793285	TCGCCTTCA	1.8745	-0.8706	-10.4611	Significant
CDK14	chr7 90603011-90603019	TGATGGCGA	-0.4594	-0.1301	-11.6240	Significant
Intergenic	chr7 93007827-93007835	TGATGGCGA	2.0000	NA	-6.4695	Significant
CALCR	chr7 93195848-93195856	TCGCCTTCA	0.8480	0.1850	-8.4538	Significant
PPP1R9A	chr7 94638733-94638741	TCGCCATCA	5.0000	-1.5230	-8.0378	Significant
PPP1R9A	chr7 94892883-94892891	ACGCCATCA	-0.2630	-1.5230	-5.7411	Significant
PDK4	chr7 95224405-95224413	TGTTGGCGA	1.8745	-1.4833	-7.0774	Significant
Intergenic	chr7 95360604-95360611	TGTTGGCGT	5.0000	NA	-9.5610	Significant
SLC25A13	chr7 95753788-95753796	TGATGGCGT	0.7776	-1.1430	-6.3196	Significant
SLC25A13	chr7 95860229-95860237	TCGCCAACA	2.8074	-1.1430	-6.3302	Significant

Intergenic	chr7	96999973-96999981	TGTTGGCGT	1.0000	NA	-5.0497	Significant
Intergenic	chr7	97096675-97096683	TGATGGCGA	5.0000	NA	-7.8717	Significant
LMTK2	chr7	97768574-97768582	ACGCCATCA	-1.8074	-3.2880	-7.2348	Significant
LMTK2	chr7	97791920-97791928	TCGCCATCA	0.6781	-3.2880	-6.1002	Significant
Intergenic	chr7	98378920-98378928	TGATGGCGT	5.0000	NA	-10.0223	Significant
CYP3A5	chr7	99253255-99253263	TGATGGCGA	5.0000	0.7149	-6.1002	Significant
Intergenic	chr7	99576098-99576106	TGATGGCGA	0.3626	NA	-5.2534	Significant
STAG3	chr7	99801966-99801974	ACGCCAACA	3.8074	0.0288	-8.7043	Significant
GATS	chr7	99801966-99801974	ACGCCAACA	0.8480	-0.4297	-6.3300	Significant
Intergenic	chr7	100311410-100311418	TGAAGGCGA	5.0000	NA	-7.4701	Significant
DPY19L2P2	chr7	102819965-102819973	TGATGGCGT	5.0000	0.1609	-9.5610	Significant
Intergenic	chr7	102924859-102924867	TGAAGGCGT	1.4854	NA	-9.3124	Significant
PSMC2	chr7	102998617-102998625	TGAAGGCGT	1.7370	-1.0855	-9.1344	Significant
SLC26A5	chr7	102998617-102998625	TGAAGGCGT	0.8745	0.1023	-5.9672	Significant
RELN	chr7	103489101-103489109	ACGCCTTCA	5.0000	0.5989	-19.0764	Significant
LHFPL3	chr7	103970628-103970636	TGATGGCGA	5.0000	0.1094	-12.0516	Significant
Intergenic	chr7	104601464-104601472	ACGCCAACA	0.2895	NA	-8.4534	Significant
EFCAB10	chr7	105221403-105221411	ACGCCTACA	5.0000	0.0588	-9.5737	Significant
Intergenic	chr7	105719946-105719954	TGTAGGCGT	3.8074	NA	-13.3821	Significant
NAMPT	chr7	105914426-105914434	ACGCCAACA	5.0000	-1.3024	-7.8720	Significant
COG5	chr7	107023321-107023329	TCGCCTACA	5.0000	-0.5225	-8.0891	Significant
LAMB1	chr7	107622986-107622994	ACGCCAACA	5.0000	-1.3136	-7.6831	Significant
Intergenic	chr7	110020628-110020636	TCGCCATCA	1.2801	NA	-10.4803	Significant
IMMP2L	chr7	110631514-110631522	ACGCCATCA	0.2224	-0.0866	-4.7190	Significant
Intergenic	chr7	111254541-111254549	ACGCCATCA	2.8074	NA	-8.4534	Significant
DOCK4	chr7	111550790-111550798	ACGCCTTCA	5.0000	-1.1261	-9.4287	Significant
IFRD1	chr7	112086330-112086338	TGAAGGCGA	5.0000	-2.3858	-5.1013	Significant
FOXP2	chr7	113836122-113836130	TGATGGCGT	-0.5146	-0.1782	-7.6316	Significant
FOXP2	chr7	114290041-114290049	TCGCCTTCA	2.2224	-0.1782	-8.7043	Significant
Intergenic	chr7	114832078-114832086	TCGCCAACA	2.1699	NA	-7.7711	Significant
Intergenic	chr7	115308952-115308960	TGAAGGCGA	1.8074	NA	-7.2424	Significant
Intergenic	chr7	115772096-115772104	TGATGGCGA	1.4594	NA	-5.6142	Significant
Intergenic	chr7	116114901-116114909	TGTTGGCGT	5.0000	NA	-7.2864	Significant
Intergenic	chr7	116273372-116273380	ACGCCTTCA	3.8074	NA	-17.5297	Significant
Intergenic	chr7	117343697-117343705	TCGCCTTCA	3.5850	NA	-14.6024	Significant
CTTNBP2	chr7	117417612-117417620	TGTTGGCGT	2.1699	-2.8224	-6.5209	Significant
Intergenic	chr7	117771535-117771543	TGTAGGCGT	5.0000	NA	-5.9549	Significant
Intergenic	chr7	118522047-118522053	TGAAGGCGA	5.0000	NA	-8.2834	Significant
Intergenic	chr7	121065827-121065835	TCGCCATCA	5.0000	NA	-9.3124	Significant
SPAM1	chr7	123568596-123568604	TGTAGGCGA	2.9069	0.0837	-6.1522	Significant
Intergenic	chr7	123624239-123624247	ACGCCATCA	3.1699	NA	-7.2348	Significant
Intergenic	chr7	123949807-123949815	TGATGGCGA	5.0000	NA	-6.5209	Significant
Intergenic	chr7	124933213-124933221	TCGCCTTCA	-1.0000	NA	-7.8720	Significant
Intergenic	chr7	125791783-125791791	TGATGGCGT	0.9069	NA	-8.7043	Significant
GRM8	chr7	126775780-126775788	TCGCCTACA	5.0000	-0.2224	-10.0223	Significant
GRM8	chr7	126832827-126832835	TGTTGGCGA	3.4594	-0.2224	-5.7924	Significant
Intergenic	chr7	126969315-126969323	TCGCCAACA	1.4594	NA	-5.5994	Significant
Intergenic	chr7	128009139-128009147	TGATGGCGT	1.5850	NA	-7.8720	Significant
UBE2H	chr7	129544161-129544169	TGTTGGCGA	1.0000	-2.6901	-4.3965	Significant
Intergenic	chr7	130554306-130554314	TGAAGGCGA	2.5850	NA	-6.8990	Significant
LOC100506860	chr7	130603264-130603272	ACGCCAACA	5.0000	NA	-8.0891	Significant
MKLN1	chr7	130871690-130871698	TGTAGGCGT	0.5850	-1.6050	-4.5891	Significant
MKLN1	chr7	131044709-131044717	TGTTGGCGA	1.7370	-1.6050	-6.4691	Significant
FLJ40288	chr7	132340837-132340845	TGAAGGCGT	5.0000	0.0289	-5.7407	Significant
CHCHD3	chr7	132758683-132758691	ACGCCTACA	1.2630	-1.0205	-13.4241	Significant
Intergenic	chr7	132880365-132880373	TGAAGGCGA	-0.2895	NA	-11.1427	Significant
Intergenic	chr7	132914508-132914516	TGATGGCGA	5.0000	NA	-7.6339	Significant
EXOC4	chr7	133432553-133432561	ACGCCAACA	5.0000	-0.2095	-13.1223	Significant
LRGUK	chr7	133940497-133940505	TCGCCTACA	2.4594	-0.0523	-7.4699	Significant
Intergenic	chr7	133949237-133949245	TCGCCAACA	5.0000	NA	-5.7924	Significant
Intergenic	chr7	134096101-134096109	TGAAGGCGA	3.4594	NA	-11.1427	Significant
Intergenic	chr7	135038107-135038115	TGAAGGCGT	3.1699	NA	-7.4727	Significant
Promoter_PTN	chr7	137029386-137029394	TGATGGCGT	2.4594	0.1117	-8.8782	Significant
TRIM24	chr7	138233274-138233282	TGAAGGCGT	2.3219	-4.9619	-22.2522	Significant
ZC3HAV1	chr7	138748448-138748456	TGATGGCGT	0.7370	-1.0671	-10.0223	Significant

PARP12	chr7 139733771-139733779	TCGCCTTCA	5.0000	-0.7843	-8.8782	Significant
SLC37A3	chr7 140074032-140074040	ACGCCTACA	0.8931	-2.0260	-5.9550	Significant
DENND2A	chr7 140237741-140237749	TGAAGGCGA	-0.5850	0.4902	-8.9291	Significant
Intergenic	chr7 140851492-140851500	ACGCCATCA	-0.7655	NA	-6.3198	Significant
Intergenic	chr7 141974022-141974030	TGAAGGCGA	5.0000	NA	-11.1427	Significant
Intergenic	chr7 142363576-142363584	TGAAGGCGT	-0.1255	NA	-6.5209	Significant
Intergenic	chr7 143788346-143788354	TGATGGCGT	2.4150	NA	-6.8481	Significant
Intergenic	chr7 144143007-144143013	TGTAGGCGT	3.7004	NA	-15.8069	Significant
TPK1	chr7 144442485-144442493	ACGCCATCA	3.5850	-2.0311	-5.2534	Significant
CNTNAP2	chr7 147085875-147085883	TGTTGGCGA	1.0000	-0.3180	-6.1006	Significant
CNTNAP2	chr7 147538394-147538402	TCGCCAACA	2.1699	-0.3180	-12.1138	Significant
EZH2	chr7 148523589-148523597	TCGCCTACA	5.0000	-2.5431	-5.5996	Significant
ACTR3C	chr7 150009183-150009191	ACGCCAACA	1.5850	-0.3818	-5.5997	Significant
GIMAP2	chr7 150386830-150386838	TGTTGGCGT	5.0000	0.1558	-5.9550	Significant
Intergenic	chr7 150992022-150992030	TGTTGGCGT	3.4594	NA	-9.3627	Significant
RHEB	chr7 151209551-151209559	TCGCCATCA	1.2224	-0.5492	-5.0509	Significant
PRKAG2	chr7 151327087-151327095	ACGCCAACA	1.8074	-0.6918	-7.8717	Significant
Intergenic	chr7 152256876-152256884	ACGCCATCA	-0.8301	NA	-5.1013	Significant
Intergenic	chr7 153184030-153184038	TCGCCTACA	3.9069	NA	-7.0772	Significant
DPP6	chr7 153615261-153615269	TGATGGCGT	1.2224	0.0655	-5.0509	Significant
DPP6	chr7 154201307-154201315	TGATGGCGA	5.0000	0.0655	-16.3722	Significant
DPP6	chr7 154468954-154468962	ACGCCAACA	5.0000	0.0655	-9.2913	Significant
CNPY1	chr7 155297503-155297511	TCGCCTTCA	5.0000	0.3571	-11.6709	Significant
Intergenic	chr7 156823274-156823282	TGATGGCGA	2.7004	NA	-9.5737	Significant
PTPRN2	chr7 158035867-158035875	ACGCCTTCA	0.5850	0.2611	-8.2802	Significant
ESYT2	chr7 158556922-158556930	TGAAGGCGT	2.7004	-0.1305	-9.5737	Significant
Intergenic	chr8 1401648-1401656	TGTTGGCGT	3.0000	NA	-10.4804	Significant
Intergenic	chr8 1664182-1664190	ACGCCTTCA	0.3219	NA	-4.7186	Significant
Intergenic	chr8 2139992-2140001	TGAAGGCGT	1.0000	NA	-5.4423	Significant
Intergenic	chr8 2216801-2216809	ACGCCTTCA	5.0000	NA	-5.3906	Significant
CSMD1	chr8 3468441-3468449	TGATGGCGA	-0.1375	0.2215	-7.2352	Significant
CSMD1	chr8 4460144-4460152	TGAAGGCGT	0.3219	0.2215	-4.7186	Significant
CSMD1	chr8 4653290-4653298	ACGCCTTCA	-1.1699	0.2215	-5.4421	Significant
Intergenic	chr8 4892038-4892046	ACGCCTTCA	5.0000	NA	-7.8722	Significant
Intergenic	chr8 5829412-5829420	TGAAGGCGT	2.5850	NA	-7.0772	Significant
Intergenic	chr8 5981160-5981168	ACGCCTTCA	0.1699	NA	-6.8473	Significant
MCPH1	chr8 6408871-6408879	ACGCCTTCA	0.6521	-2.5246	-6.8473	Significant
ANGPT2	chr8 6408871-6408879	ACGCCTTCA	3.7004	-0.0708	-4.2929	Marginal
Intergenic	chr8 8505712-8505720	TCGCCTTCA	0.3219	NA	-7.4699	Significant
Intergenic	chr8 8543148-8543156	TGATGGCGA	5.0000	NA	-15.5732	Significant
Intergenic	chr8 8791052-8791060	TGATGGCGT	2.3219	NA	-7.0774	Significant
LOC157273	chr8 9186939-9186947	TGTTGGCGA	1.5850	0.1812	-8.0378	Significant
Intergenic	chr8 9307886-9307893	TGAAGGCGA	5.0000	NA	-8.0348	Significant
Intergenic	chr8 9320613-9320621	ACGCCATCA	5.0000	NA	-5.0027	Significant
MSRA	chr8 10035213-10035221	TGTTGGCGT	2.3219	0.3336	-10.9247	Significant
DEFB135	chr8 11841974-11841982	TGTTGGCGA	0.0000	-0.1637	-6.7014	Significant
DLC1	chr8 13029523-13029531	ACGCCATCA	5.0000	-0.8021	-8.8782	Significant
Intergenic	chr8 13582236-13582244	ACGCCTTCA	0.5850	NA	-5.3906	Significant
SGCZ	chr8 14368099-14368107	ACGCCATCA	3.0000	0.1324	-13.9474	Significant
Intergenic	chr8 15260010-15260018	TGAAGGCGT	1.0000	NA	-8.0378	Significant
VPS37A	chr8 17143385-17143393	TGTAGGCGA	5.0000	-2.7215	-5.4423	Significant
PDGFRL	chr8 17476481-17476489	TGATGGCGT	5.0000	-0.1528	-7.8720	Significant
MTUS1	chr8 17563684-17563692	TCGCCATCA	5.0000	-1.3519	-11.4243	Significant
MTUS1	chr8 17647078-17647086	ACGCCAACA	0.5146	-1.3519	-8.7041	Significant
Intergenic	chr8 18350484-18350492	TCGCCAACA	1.1699	NA	-8.0378	Significant
Intergenic	chr8 19149181-19149189	ACGCCAACA	2.0000	NA	-6.3196	Significant
CSGALNACT1	chr8 19315491-19315498	TGAAGGCGA	5.0000	-0.2324	-17.2828	Significant
Intergenic	chr8 19917429-19917437	TGTAGGCGT	-0.3479	NA	-5.9549	Significant
XPO7	chr8 21786055-21786063	TCGCCAACA	3.5850	-2.1755	-7.2866	Significant
LOXL2	chr8 23230765-23230773	TCGCCTACA	3.0000	-0.2109	-5.3910	Significant
Intergenic	chr8 23874029-23874037	TCGCCTTCA	2.1155	NA	-7.8720	Significant
Intergenic	chr8 25685697-25685705	TCGCCTTCA	5.0000	NA	-14.4288	Significant
PPP2R2A	chr8 26218026-26218034	ACGCCATCA	2.5850	-2.0823	-4.9165	Significant
Intergenic	chr8 26899557-26899565	TGAAGGCGA	1.2224	NA	-6.4866	Significant
PTK2B	chr8 27222741-27222749	TGATGGCGA	3.5850	-1.2743	-8.0346	Significant

FBXO16	chr8 28326227-28326235	TGTTGGCGT	3.9069	-2.6184	-6.8473	Significant
HMBOX1	chr8 28772053-28772061	ACGCCTTCA	0.9175	-1.6844	-8.0378	Significant
LEPROTL1	chr8 29964932-29964940	TCGCCATCA	2.4594	-0.3512	-10.0064	Significant
NRG1	chr8 31904815-31904823	TGTAGGCGA	0.6781	0.3413	-10.6711	Significant
Intergenic	chr8 34381025-34381033	TCGCCAACA	2.0000	NA	-6.4691	Significant
KCNU1	chr8 36782776-36782784	TCGCCTTCA	3.1699	0.3269	-14.7350	Significant
Intergenic	chr8 37036188-37036196	TGAAGGCGA	3.5850	NA	-11.1431	Significant
Intergenic	chr8 37090098-37090106	TCGCCTTCA	-0.4150	NA	-9.1344	Significant
FGFR1	chr8 38269042-38269050	TCGCCAACA	5.0000	0.0910	-7.0772	Significant
Intergenic	chr8 39717854-39717862	TCGCCTTCA	0.5850	NA	-5.0497	Significant
Intergenic	chr8 40984770-40984778	ACGCCAACA	-0.4150	NA	-6.4695	Significant
SLC20A2	chr8 42352138-42352146	ACGCCATCA	1.8745	-2.9571	-5.9549	Significant
Intergenic	chr8 43622925-43622933	ACGCCTACA	5.0000	NA	-7.6831	Significant
Intergenic	chr8 48053257-48053265	TCGCCTTCA	5.0000	NA	-5.9549	Significant
Intergenic	chr8 48361111-48361119	TGTAGGCGT	3.1699	NA	-8.2834	Significant
PRKDC	chr8 48789084-48789092	TGTTGGCGT	0.3219	0.2237	-8.9289	Significant
Intergenic	chr8 50058945-50058953	TGTAGGCGA	-1.4150	NA	-6.3198	Significant
PXDNL	chr8 52661548-52661556	TGAAGGCGT	2.1155	0.0088	-7.4699	Significant
Intergenic	chr8 52723008-52723016	TGTAGGCGA	1.1375	NA	-5.7415	Significant
ST18	chr8 53317942-53317950	ACGCCAACA	5.0000	-0.1495	-4.9165	Significant
Intergenic	chr8 53336788-53336796	TCGCCAACA	3.1699	NA	-5.2533	Significant
Intergenic	chr8 55306250-55306258	ACGCCAACA	1.2801	NA	-6.8473	Significant
Intergenic	chr8 55750171-55750179	TGATGGCGA	3.9069	NA	-8.2838	Significant
Intergenic	chr8 55909812-55909820	ACGCCTTCA	5.0000	NA	-12.1603	Significant
XKR4	chr8 56128465-56128473	TGATGGCGA	5.0000	0.1173	-6.7014	Significant
XKR4	chr8 56238793-56238801	TGTTGGCGA	5.0000	0.1173	-8.9289	Significant
TMEM68	chr8 56685135-56685143	ACGCCTACA	1.5850	-0.9146	-8.8782	Significant
Promoter_TGS1	chr8 56685135-56685143	ACGCCTACA	-0.3626	-3.5468	-6.1520	Significant
PLAG1	chr8 57104938-57104946	TCGCCAACA	0.4854	-3.2530	-6.1006	Significant
Intergenic	chr8 57433530-57433538	TGTAGGCGT	5.0000	NA	-8.9289	Significant
TOX	chr8 60016273-60016281	ACGCCAACA	-0.2895	-2.6773	-4.9165	Significant
Intergenic	chr8 61088341-61088349	TCGCCATCA	-0.2895	NA	-5.7924	Significant
CA8	chr8 61172378-61172386	ACGCCTTCA	1.8745	-1.8380	-7.6316	Significant
RAB2A	chr8 61441162-61441170	TGTTGGCGT	5.0000	-0.4298	-5.7924	Significant
NKAIN3	chr8 63568676-63568684	TCGCCTTCA	-0.3479	0.3182	-5.7924	Significant
Intergenic	chr8 64409233-64409241	TGAAGGCGA	2.0000	NA	-9.5736	Significant
LINC00251	chr8 66086309-66086317	TGATGGCGA	5.0000	0.1322	-15.8466	Significant
PDE7A	chr8 66639589-66639597	ACGCCATCA	5.0000	-1.0480	-4.7693	Significant
Promoter_PDE7A	chr8 66702218-66702226	ACGCCAACA	2.3219	-1.0480	-6.1002	Significant
PDE7A	chr8 66702218-66702226	ACGCCAACA	5.0000	-1.0480	-20.5887	Significant
Intergenic	chr8 67097397-67097405	TGAAGGCGA	0.4150	NA	-6.6941	Significant
Intergenic	chr8 70122595-70122603	TCGCCAACA	1.3219	NA	-5.5996	Significant
Intergenic	chr8 70988508-70988516	ACGCCTTCA	5.0000	NA	-6.3196	Significant
Intergenic	chr8 71367187-71367195	TGATGGCGA	2.4594	NA	-8.7041	Significant
Intergenic	chr8 71429913-71429921	ACGCCTACA	2.4594	NA	-5.9549	Significant
Intergenic	chr8 72080287-72080295	TGTAGGCGT	0.8074	NA	-7.2864	Significant
Intergenic	chr8 72608154-72608162	TCGCCTTCA	0.2895	NA	-8.8786	Significant
STAU2	chr8 74493647-74493655	ACGCCTACA	5.0000	-1.0375	-10.4803	Significant
UBE2W	chr8 74749316-74749324	ACGCCATCA	1.7655	-2.5087	-10.0223	Significant
Intergenic	chr8 76715421-76715429	TGTAGGCGA	5.0000	NA	-5.1011	Significant
Intergenic	chr8 77561520-77561528	TGTAGGCGA	2.6630	NA	-24.9608	Significant
ZFHX4	chr8 77742405-77742413	TGATGGCGA	1.5850	0.2024	-5.7924	Significant
Intergenic	chr8 80340800-80340808	ACGCCTTCA	0.1069	NA	-9.3128	Significant
TPD52	chr8 81020284-81020292	TGATGGCGT	5.0000	-0.2436	-11.1907	Significant
Intergenic	chr8 82641904-82641911	TGTAGGCGA	-1.2224	NA	-7.4699	Significant
Intergenic	chr8 83125221-83125229	TCGCCAACA	3.8074	NA	-7.8720	Significant
E2F5	chr8 86116851-86116859	ACGCCAACA	0.3479	-1.9970	-5.3910	Significant
C8orf59	chr8 86131572-86131580	ACGCCAACA	0.2895	0.4654	-4.2711	Marginal
Intergenic	chr8 87028276-87028284	TCGCCATCA	1.8745	NA	-11.6240	Significant
Intergenic	chr8 87292006-87292014	TCGCCTTCA	1.3219	NA	-8.2834	Significant
Intergenic	chr8 89025168-89025176	ACGCCATCA	0.5850	NA	-8.4534	Significant
Intergenic	chr8 89891172-89891180	ACGCCTTCA	3.3219	NA	-4.9165	Significant
Intergenic	chr8 91168411-91168419	TGATGGCGT	0.1375	NA	-7.2424	Significant
Intergenic	chr8 91304693-91304700	TCGCCTTCA	1.0000	NA	-5.1011	Significant
NECAB1	chr8 91886677-91886685	TGTAGGCGT	2.5850	-0.0091	-5.9550	Significant

NECAB1	chr8 91936898-91936906	TGTTGGCGA	5.0000	-0.0091	-6.4695	Significant
Intergenic	chr8 91987697-91987705	ACGCCATCA	3.0000	NA	-10.2583	Significant
RUNX1T1	chr8 93034338-93034346	TGATGGCGA	3.7004	0.2937	-4.3961	Significant
Intergenic	chr8 94316594-94316602	ACGCCAACA	1.5850	NA	-5.4421	Significant
Intergenic	chr8 94491156-94491164	TGTAGGCGA	2.5850	NA	-17.2828	Significant
Intergenic	chr8 95103356-95103364	TGTAGGCGT	5.0000	NA	-4.3965	Significant
Promoter_RAD54B	chr8 95449215-95449223	ACGCCTACA	1.7370	-2.2835	-8.0382	Significant
RAD54B	chr8 95449215-95449223	ACGCCTACA	2.7370	-2.2835	-12.4056	Significant
Intergenic	chr8 96122469-96122477	TCGCCTACA	0.7370	NA	-11.1427	Significant
Intergenic	chr8 96437814-96437822	TGTAGGCGA	0.6521	NA	-8.0893	Significant
Intergenic	chr8 96571615-96571623	ACGCCTTCA	1.8745	NA	-13.9478	Significant
Intergenic	chr8 98162856-98162864	TCGCCTTCA	1.4594	NA	-5.5994	Significant
Intergenic	chr8 98563017-98563025	TCGCCATCA	0.7004	NA	-10.2089	Significant
MATN2	chr8 98891124-98891132	TCGCCTACA	1.0000	0.0961	-8.0346	Significant
Intergenic	chr8 99422527-99422535	TCGCCTTCA	5.0000	NA	-6.3196	Significant
STK3	chr8 99902462-99902470	ACGCCAACA	0.1155	-1.9959	-7.0823	Significant
STK3	chr8 99944087-99944095	TCGCCTTCA	5.0000	-1.9959	-5.3910	Significant
VPS13B	chr8 100572715-100572723	TCGCCATCA	5.0000	-0.3960	-5.4421	Significant
RGS22	chr8 100997825-100997833	ACGCCTACA	0.6781	-0.0505	-5.2534	Significant
Intergenic	chr8 101399746-101399754	TCGCCTACA	3.1699	NA	-4.8879	Significant
SNX31	chr8 101626754-101626762	ACGCCATCA	-1.0000	0.1411	-7.6316	Significant
YWHAZ	chr8 101952397-101952405	TGAAGGCGT	2.3219	0.3781	-5.9549	Significant
UBR5	chr8 103353764-103353772	TCGCCTTCA	3.0000	-0.9208	-9.1342	Significant
UBR5	chr8 103391523-103391531	TCGCCATCA	0.1375	-0.9208	-6.5209	Significant
Intergenic	chr8 103448211-103448219	TCGCCATCA	5.0000	NA	-11.6236	Significant
Intergenic	chr8 103983229-103983237	TGTTGGCGT	4.0000	NA	-6.8990	Significant
RIMS2	chr8 105116070-105116078	ACGCCATCA	1.3219	0.2483	-5.3914	Significant
Intergenic	chr8 105828266-105828274	TGATGGCGT	5.0000	NA	-4.5890	Significant
Intergenic	chr8 108080759-108080767	TCGCCTTCA	2.1155	NA	-10.0224	Significant
Intergenic	chr8 109647819-109647827	TCGCCAACA	3.9069	NA	-10.0224	Significant
Intergenic	chr8 109726765-109726773	TGTTGGCGA	0.0000	NA	-6.1520	Significant
Intergenic	chr8 109908578-109908586	TGAAGGCGA	5.0000	NA	-5.7407	Significant
SYBU	chr8 110636829-110636837	TCGCCAACA	3.4594	-3.2150	-4.5890	Significant
Intergenic	chr8 110805594-110805602	TGATGGCGT	5.0000	NA	-4.5890	Significant
Intergenic	chr8 110932589-110932596	ACGCCTTCA	-1.1926	NA	-5.7924	Significant
Intergenic	chr8 111400644-111400652	TGAAGGCGT	2.5850	NA	-4.9165	Significant
Intergenic	chr8 111880599-111880607	TCGCCAACA	2.5850	NA	-6.6941	Significant
Intergenic	chr8 115936855-115936863	TGTTGGCGA	5.0000	NA	-7.2348	Significant
Intergenic	chr8 116692229-116692237	TCGCCTACA	4.0875	NA	-6.8473	Significant
Intergenic	chr8 116707762-116707770	TGAAGGCGA	5.0000	NA	-13.4243	Significant
Intergenic	chr8 117062578-117062586	TGATGGCGT	2.4594	NA	-8.7041	Significant
EXT1	chr8 118819601-118819609	TGAAGGCGA	5.0000	-2.5874	-6.3196	Significant
EXT1	chr8 119026329-119026337	TGATGGCGA	1.8074	-2.5874	-6.6938	Significant
Intergenic	chr8 119858093-119858101	TGAAGGCGT	5.0000	NA	-6.8473	Significant
Intergenic	chr8 120167188-120167196	TGTAGGCGT	5.0000	NA	-7.8844	Significant
Intergenic	chr8 120407604-120407612	TGATGGCGA	-0.9475	NA	-5.4423	Significant
Intergenic	chr8 120676077-120676085	TGTAGGCGT	5.0000	NA	-5.0497	Significant
COL14A1	chr8 121306236-121306244	TGATGGCGT	1.7370	0.1957	-7.2348	Significant
Intergenic	chr8 122686269-122686277	TCGCCTTCA	1.0000	NA	-8.0378	Significant
Intergenic	chr8 122808768-122808776	TCGCCAACA	2.0000	NA	-8.2834	Significant
Intergenic	chr8 124646594-124646602	TCGCCATCA	5.0000	NA	-4.2710	Marginal
FER1L6	chr8 124891406-124891414	TGATGGCGT	0.5146	-0.1763	-6.4699	Significant
FER1L6	chr8 125079645-125079653	ACGCCAACA	1.4150	-0.1763	-7.6316	Significant
Intergenic	chr8 125389725-125389733	TCGCCAACA	-0.2895	NA	-6.4691	Significant
Intergenic	chr8 125432451-125432459	TCGCCATCA	5.0000	NA	-5.7926	Significant
Intergenic	chr8 125963968-125963976	ACGCCAACA	-0.9175	NA	-11.6240	Significant
NSMCE2	chr8 126131514-126131522	TGATGGCGT	1.4150	-1.3215	-8.0378	Significant
Intergenic	chr8 126563579-126563587	TGAAGGCGT	5.0000	NA	-11.1907	Significant
Intergenic	chr8 126686230-126686238	TGTAGGCGT	-0.4150	NA	-4.7567	Significant
Intergenic	chr8 127897925-127897933	TCGCCTTCA	0.5305	NA	-11.6236	Significant
Intergenic	chr8 128129262-128129270	TGAAGGCGA	3.3219	NA	-7.6316	Significant
Intergenic	chr8 128174381-128174389	ACGCCATCA	1.1699	NA	-13.1223	Significant
Intergenic	chr8 128601924-128601932	TGTTGGCGA	2.9069	NA	-8.0891	Significant
Intergenic	chr8 129527843-129527851	ACGCCTTCA	1.5850	NA	-9.3627	Significant
FAM49B	chr8 130857364-130857372	TCGCCATCA	1.0000	-1.4803	-4.3961	Significant

Intergenic	chr8 131476200-131476208	TGTTGGCGA	1.5236	NA	-4.9350	Significant
ADCY8	chr8 131908678-131908686	ACGCCTTCA	0.7370	0.1366	-5.7407	Significant
EFR3A	chr8 132995875-132995883	TCGCCTTCA	-0.2895	-1.0267	-7.0772	Significant
HHLA1	chr8 133076364-133076372	TCGCCAACA	1.4594	0.2306	-6.8477	Significant
ZFAT	chr8 135564844-135564852	TGATGGCGT	2.9069	-2.3252	-6.6941	Significant
Intergenic	chr8 135766417-135766425	TCGCCTACA	5.0000	NA	-5.2699	Significant
Intergenic	chr8 135917867-135917875	TGATGGCGA	5.0000	NA	-6.8315	Significant
KHDRBS3	chr8 136545311-136545319	TGATGGCGT	1.4594	-1.5724	-10.7198	Significant
Intergenic	chr8 137853835-137853843	TGTAGGCGT	-1.2410	NA	-7.6316	Significant
Intergenic	chr8 137910495-137910503	TCGCCTTCA	0.2224	NA	-7.4699	Significant
Intergenic	chr8 138445697-138445705	TGATGGCGA	5.0000	NA	-5.4421	Significant
Intergenic	chr8 138558851-138558859	TCGCCATCA	3.1699	NA	-6.4691	Significant
FAM135B	chr8 139329938-139329946	ACGCCTACA	5.0000	0.2574	-6.8473	Significant
TRAPPC9	chr8 140962387-140962395	TGATGGCGA	2.3219	0.0934	-6.8477	Significant
Intergenic	chr8 143768684-143768692	TGATGGCGT	3.7004	NA	-4.9350	Significant
Intergenic	chr8 145143435-145143443	TGATGGCGA	5.0000	NA	-6.5209	Significant
DOCK8	chr9 302196-302204	ACGCCTACA	1.8745	0.1565	-5.9549	Significant
DOCK8	chr9 328085-328093	ACGCCTTCA	-0.5025	0.1565	-5.5994	Significant
DMRT1	chr9 917297-917305	TGAAGGCGT	-1.0780	-0.0787	-5.3906	Significant
DMRT1	chr9 952870-952878	TGTTGGCGT	0.3479	-0.0787	-5.3906	Significant
Intergenic	chr9 1365420-1365427	TCGCCATCA	0.0000	NA	-12.6591	Significant
Intergenic	chr9 1375149-1375157	ACGCCAACA	3.7004	NA	-8.4534	Significant
Intergenic	chr9 1872496-1872504	TGATGGCGA	0.1255	NA	-10.4609	Significant
Intergenic	chr9 3759137-3759145	TGATGGCGA	3.3219	NA	-5.4421	Significant
Intergenic	chr9 4370915-4370923	ACGCCTTCA	0.4695	NA	-6.3300	Significant
Promoter_INSL6	chr9 5186581-5186589	TGTTGGCGT	2.8074	0.0080	-4.9165	Significant
Intergenic	chr9 5732838-5732846	ACGCCTTCA	3.0000	NA	-7.8720	Significant
Intergenic	chr9 5913930-5913938	ACGCCTACA	0.8931	NA	-5.2533	Significant
Intergenic	chr9 6022087-6022095	TCGCCATCA	5.0000	NA	-12.0511	Significant
Intergenic	chr9 6068442-6068450	TCGCCTTCA	-0.2814	NA	-4.7182	Significant
IL33	chr9 6240678-6240686	TGAAGGCGT	-0.8931	0.0598	-6.4699	Significant
UHRF2	chr9 6483134-6483142	TCGCCAACA	0.2895	-1.2170	-5.7924	Significant
GLDC	chr9 6561520-6561528	ACGCCTACA	-1.1699	0.0227	-10.9476	Significant
GLDC	chr9 6623918-6623926	TGTAGGCGA	1.7370	0.0227	-10.1750	Significant
Intergenic	chr9 8295966-8295974	ACGCCATCA	5.0000	NA	-15.2513	Significant
PTPRD	chr9 8679534-8679542	ACGCCTTCA	0.7776	-3.0021	-5.3906	Significant
PTPRD	chr9 8937042-8937050	TGATGGCGT	3.1699	-3.0021	-9.0007	Significant
Intergenic	chr9 13061221-13061229	TGAAGGCGT	1.3785	NA	-7.2864	Significant
Intergenic	chr9 13504918-13504926	TGTTGGCGA	5.0000	NA	-6.6938	Significant
Intergenic	chr9 18143585-18143593	TGTAGGCGT	2.5850	NA	-7.0775	Significant
Intergenic	chr9 18314056-18314064	ACGCCAACA	1.3219	NA	-6.6938	Significant
ADAMTSL1	chr9 18559559-18559567	TGATGGCGA	1.5850	-0.3552	-5.1015	Significant
FAM154A	chr9 19029171-19029179	TCGCCAACA	5.0000	-0.0537	-12.8718	Significant
HAUS6	chr9 19065647-19065655	TCGCCAACA	1.0000	-2.3633	-7.4699	Significant
ACER2	chr9 19422675-19422683	ACGCCTTCA	0.5850	0.3831	-4.3961	Significant
Intergenic	chr9 19876357-19876365	TGAAGGCGT	0.7370	NA	-8.2834	Significant
Intergenic	chr9 20255166-20255174	ACGCCATCA	3.5850	NA	-5.3910	Significant
Intergenic	chr9 23235016-23235024	TGAAGGCGT	5.0000	NA	-9.3124	Significant
Intergenic	chr9 24973484-24973492	TCGCCTTCA	-1.1375	NA	-4.7693	Significant
Intergenic	chr9 25975502-25975510	TGAAGGCGT	1.5025	NA	-7.0823	Significant
Intergenic	chr9 26319273-26319281	ACGCCTTCA	-1.2801	NA	-5.2534	Significant
TEK	chr9 27182620-27182628	TCGCCTACA	0.5305	-0.1674	-11.5686	Significant
LINGO2	chr9 27987932-27987940	TGTTGGCGT	3.0000	-0.7184	-4.7823	Significant
LINGO2	chr9 28507644-28507652	ACGCCTACA	-0.7004	-0.7184	-6.1002	Significant
Intergenic	chr9 29503206-29503214	TGATGGCGA	5.0000	NA	-4.2708	Marginal
Intergenic	chr9 30534020-30534028	TGTTGGCGA	0.2224	NA	-7.7708	Significant
Intergenic	chr9 32281008-32281016	TCGCCATCA	1.1155	NA	-8.8782	Significant
Intergenic	chr9 32798333-32798341	ACGCCATCA	0.8931	NA	-7.2864	Significant
Intergenic	chr9 34409445-34409453	TGAAGGCGT	5.0000	NA	-8.7043	Significant
Intergenic	chr9 35671781-35671789	TGATGGCGT	2.8074	NA	-6.8992	Significant
Intergenic	chr9 35790470-35790478	TCGCCTTCA	5.0000	NA	-7.4699	Significant
LOC642236	chr9 68435874-68435882	ACGCCAACA	3.0000	-1.1539	-5.0497	Significant
Intergenic	chr9 68497185-68497193	ACGCCATCA	1.2630	NA	-5.2533	Significant
PGM5	chr9 71046163-71046171	TCGCCAACA	-0.2224	-0.4766	-5.0497	Significant
PIP5K1B	chr9 71500250-71500258	TGTTGGCGA	3.0000	-2.1710	-6.6938	Significant

PTAR1	chr9 72360043-72360051	ACGCCATCA	3.0000	-3.1729	-4.9165	Significant
MAMDC2	chr9 72742548-72742556	TGATGGCGT	-1.1255	-0.1107	-4.9166	Significant
TRPM3	chr9 73170769-73170777	ACGCCAACA	2.5850	0.2225	-5.3906	Significant
TRPM3	chr9 73182375-73182383	TCGCCAACA	-0.2801	0.2225	-7.0772	Significant
TRPM3	chr9 73432961-73432969	TGTTGGCGT	5.0000	0.2225	-20.5879	Significant
TRPM3	chr9 73443733-73443741	TGTAGGCGA	-0.1926	0.2225	-5.7411	Significant
TRPM3	chr9 73458022-73458030	TCGCCAACA	2.7004	0.2225	-13.4243	Significant
TRPM3	chr9 73638167-73638175	TCGCCATCA	0.6521	0.2225	-7.6316	Significant
Intergenic	chr9 74141591-74141599	TGAAGGCGT	1.8745	NA	-11.6236	Significant
TMEM2	chr9 74362095-74362103	ACGCCTTCA	5.0000	-1.4386	-6.1002	Significant
TMEM2	chr9 74362095-74362103	TCGCCTTCA	5.0000	-1.4386	-6.4691	Significant
Intergenic	chr9 74597460-74597468	TCGCCAACA	3.0000	NA	-16.4101	Significant
TMC1	chr9 75310466-75310474	TCGCCATCA	2.7004	-0.1942	-11.9103	Significant
Intergenic	chr9 76973797-76973805	TCGCCTTCA	-0.6280	NA	-6.6224	Significant
Intergenic	chr9 76974040-76974048	TGTTGGCGT	0.8931	NA	-8.5043	Significant
RORB	chr9 77194808-77194816	TCGCCTTCA	-2.0000	0.1740	-7.2864	Significant
Intergenic	chr9 77519843-77519851	TGATGGCGT	3.7004	NA	-6.8473	Significant
OSTF1	chr9 77730355-77730363	ACGCCATCA	5.0000	-0.1657	-7.3789	Significant
Intergenic	chr9 78397984-78397992	TCGCCAACA	2.0000	NA	-5.9549	Significant
PCSK5	chr9 78751091-78751099	TGATGGCGA	2.4594	-0.1374	-7.8722	Significant
GNA14	chr9 80078168-80078176	TCGCCATCA	-0.7776	-0.7364	-12.4056	Significant
GNA14	chr9 80222647-80222655	TCGCCAACA	1.5850	-0.7364	-5.7411	Significant
GNA14	chr9 80229465-80229473	ACGCCAACA	5.0000	-0.7364	-10.0064	Significant
Intergenic	chr9 80893270-80893278	TCGCCTACA	5.0000	NA	-5.3906	Significant
Intergenic	chr9 81309123-81309131	TCGCCTTCA	1.8074	NA	-6.6938	Significant
Intergenic	chr9 83118555-83118563	TGTAGGCGA	5.0000	NA	-10.0223	Significant
Intergenic	chr9 83446473-83446481	TGAAGGCGT	1.2224	NA	-7.0772	Significant
Intergenic	chr9 83963702-83963710	TCGCCATCA	5.0000	NA	-11.1907	Significant
Intergenic	chr9 85229782-85229790	ACGCCATCA	-0.1699	NA	-8.2836	Significant
Intergenic	chr9 87169821-87169829	ACGCCTACA	2.7004	NA	-13.9476	Significant
NTRK2	chr9 87438146-87438154	TCGCCTACA	2.0000	-0.1164	-8.5043	Significant
NTRK2	chr9 87438561-87438569	TGTTGGCGT	0.0000	-0.1164	-8.0378	Significant
NTRK2	chr9 87468609-87468617	TGTTGGCGT	2.0000	-0.1164	-5.3906	Significant
Intergenic	chr9 89178854-89178861	TGATGGCGT	1.8745	NA	-4.4469	Significant
Intergenic	chr9 89435102-89435110	TCGCCAACA	-0.4854	NA	-9.3629	Significant
Intergenic	chr9 90883074-90883082	TGATGGCGA	0.5850	NA	-10.4803	Significant
Intergenic	chr9 91884794-91884802	TGATGGCGA	0.6781	NA	-6.4699	Significant
Intergenic	chr9 92183867-92183875	TGATGGCGT	3.3219	NA	-10.2089	Significant
Intergenic	chr9 93248322-93248330	TCGCCAACA	3.3219	NA	-8.5043	Significant
Intergenic	chr9 94375327-94375335	TCGCCATCA	5.0000	NA	-8.0378	Significant
Intergenic	chr9 94440296-94440304	TGATGGCGT	3.7004	NA	-7.4699	Significant
WNK2	chr9 96042651-96042659	TGTTGGCGA	3.7004	-0.2566	-5.3914	Significant
FAM120A	chr9 96313128-96313136	TCGCCTTCA	5.0000	0.0835	-20.6158	Significant
PTCH1	chr9 98239076-98239084	TGAAGGCGT	5.0000	-3.5757	-5.5994	Significant
PTCH1	chr9 98244024-98244032	ACGCCTACA	5.0000	-3.5757	-13.5543	Significant
Intergenic	chr9 99072417-99072425	TCGCCTTCA	3.5850	NA	-7.2348	Significant
CDC14B	chr9 99352912-99352920	TCGCCAACA	3.7004	-0.5397	-6.5209	Significant
Intergenic	chr9 101648062-101648070	TGTTGGCGT	1.7370	NA	-8.4534	Significant
Intergenic	chr9 101657967-101657975	TGAAGGCGT	3.0000	NA	-5.0497	Significant
Intergenic	chr9 103696054-103696062	TCGCCAACA	0.8480	NA	-6.1520	Significant
LPPR1	chr9 104015691-104015699	TGATGGCGT	1.5850	-0.7063	-7.6316	Significant
GRIN3A	chr9 104439917-104439925	TGATGGCGA	1.3219	0.3160	-8.5046	Significant
Intergenic	chr9 106966331-106966338	TCGCCTTCA	1.0000	NA	-10.4803	Significant
Intergenic	chr9 107349923-107349931	ACGCCTACA	0.1375	NA	-4.9165	Significant
Intergenic	chr9 107747139-107747147	TGAAGGCGA	-1.7370	NA	-5.0505	Significant
Intergenic	chr9 107805826-107805834	ACGCCTTCA	5.0000	NA	-5.9550	Significant
ZNF462	chr9 109629323-109629331	TGTTGGCGT	0.0000	-1.2481	-6.4691	Significant
ZNF462	chr9 109638940-109638948	ACGCCTTCA	1.2224	-1.2481	-7.0772	Significant
Intergenic	chr9 110401052-110401060	TCGCCAACA	3.7004	NA	-7.2348	Significant
Intergenic	chr9 110812111-110812119	TGTTGGCGT	5.0000	NA	-8.7041	Significant
Intergenic	chr9 110962901-110962909	TGAAGGCGA	0.4475	NA	-11.4244	Significant
Intergenic	chr9 111255280-111255288	TCGCCAACA	1.8745	NA	-6.6938	Significant
Intergenic	chr9 111877041-111877049	ACGCCATCA	3.5850	NA	-5.9550	Significant
Intergenic	chr9 112312800-112312807	TGTTGGCGT	2.7004	NA	-6.8992	Significant
PALM2	chr9 112478559-112478567	TGAAGGCGT	2.1155	1.1809	-8.5043	Significant

ZNF483	chr9 114291010-114291018	TCGCCATCA	3.7004	-0.3336	-5.0497	Significant
Intergenic	chr9 115882565-115882573	TGATGGCGA	0.7004	NA	-7.4699	Significant
FKBP15	chr9 115952702-115952710	TGATGGCGA	-2.1468	-2.4852	-6.6939	Significant
RGS3	chr9 116316733-116316741	ACGCCATCA	5.0000	-0.7691	-7.8720	Significant
Intergenic	chr9 116864210-116864218	TGTTGGCGA	-0.2996	NA	-10.2581	Significant
Intergenic	chr9 117282164-117282172	TGATGGCGT	1.7370	NA	-5.6696	Significant
1-Dec	chr9 118029907-118029915	ACGCCAACA	3.5850	0.0928	-8.9295	Significant
Intergenic	chr9 118695089-118695097	TCGCCATCA	2.0000	NA	-9.1342	Significant
PAPPA	chr9 118937330-118937338	TCGCCAACA	2.9069	-0.8646	-6.4691	Significant
Intergenic	chr9 122956607-122956615	ACGCCATCA	5.0000	NA	-9.5736	Significant
CDK5RAP2	chr9 123181872-123181880	TCGCCAACA	5.0000	-1.4723	-4.9166	Significant
CDK5RAP2	chr9 123301951-123301959	TGTTGGCGA	1.4854	-1.4723	-8.8782	Significant
Promoter_MRRF	chr9 125032485-125032493	TGTAGGCGA	3.1699	-0.2537	-5.7411	Significant
MRRF	chr9 125032485-125032493	TGTAGGCGA	0.8745	-0.2537	-6.3196	Significant
Intergenic	chr9 126915216-126915224	TCGCCTTCA	3.5850	NA	-9.8058	Significant
Intergenic	chr9 129035007-129035015	TGTAGGCGT	0.0000	NA	-7.4699	Significant
Intergenic	chr9 129255586-129255594	TGTTGGCGA	3.5850	NA	-9.3124	Significant
SET	chr9 131452950-131452958	TGTAGGCGT	5.0000	0.3574	-6.3196	Significant
Intergenic	chr9 132060607-132060615	TGAAGGCGT	0.2224	NA	-6.8473	Significant
C9orf78	chr9 132595499-132595507	ACGCCTACA	5.0000	0.5231	-7.3789	Significant
FUBP3	chr9 133472902-133472910	ACGCCATCA	0.7370	0.2814	-18.1227	Significant
MED27	chr9 134791468-134791476	TCGCCAACA	3.4594	-0.9631	-5.9549	Significant
TSC1	chr9 135816983-135816991	ACGCCTTCA	5.0000	-1.0765	-8.2836	Significant
Intergenic	chr9 136121256-136121264	TGATGGCGA	5.0000	NA	-6.7014	Significant
EHMT1	chr9 140544531-140544539	TCGCCAACA	0.1926	-1.9691	-4.9166	Significant
Intergenic	chr10 2815859-2815867	ACGCCTTCA	-0.7370	NA	-5.9675	Significant
Intergenic	chr10 2906392-2906400	TGAAGGCGT	5.0000	NA	-6.3196	Significant
Intergenic	chr10 4241233-4241241	TGTTGGCGT	5.0000	NA	-7.4724	Significant
Intergenic	chr10 4472820-4472828	ACGCCATCA	5.0000	NA	-16.1337	Significant
AKR1C1	chr10 5008682-5008690	ACGCCTACA	5.0000	-0.1180	-10.4803	Significant
Intergenic	chr10 5724416-5724424	ACGCCTACA	3.1699	NA	-9.4287	Significant
Intergenic	chr10 6913004-6913012	ACGCCTACA	-0.2410	NA	-8.0382	Significant
TAF3	chr10 7970592-7970600	TGTTGGCGA	0.4150	-3.1870	-4.9166	Significant
Intergenic	chr10 8613939-8613947	TGAAGGCGA	0.3479	NA	-5.5994	Significant
Intergenic	chr10 8972011-8972019	TGTTGGCGT	1.3785	NA	-10.1750	Significant
Intergenic	chr10 9235481-9235489	TCGCCATCA	1.7370	NA	-7.2348	Significant
CELF2	chr10 11056981-11056989	TGATGGCGT	0.8931	-0.7850	-5.2533	Significant
CELF2	chr10 11310062-11310070	TGATGGCGT	3.4594	-0.7850	-6.8473	Significant
CAMK1D	chr10 12487893-12487901	TGTTGGCGT	3.8074	-1.2312	-5.7407	Significant
BEND7	chr10 13534375-13534383	ACGCCATCA	2.8074	0.0334	-10.0223	Significant
Intergenic	chr10 15225511-15225519	TCGCCAACA	5.0000	NA	-7.4699	Significant
Intergenic	chr10 15225896-15225904	TGAAGGCGA	0.3626	NA	-7.2864	Significant
FAM171A1	chr10 15402812-15402820	ACGCCTTCA	5.0000	-1.2275	-11.0946	Significant
Intergenic	chr10 15436440-15436448	TCGCCTTCA	5.0000	NA	-7.8720	Significant
RSU1	chr10 16705592-16705600	ACGCCTACA	-1.7370	0.1998	-6.3196	Significant
CUBN	chr10 17063415-17063423	TCGCCTTCA	1.7370	0.1355	-6.6939	Significant
Intergenic	chr10 19107087-19107095	ACGCCTTCA	1.4594	NA	-12.1138	Significant
PLXDC2	chr10 20392036-20392044	TCGCCATCA	3.1699	-0.7241	-8.6982	Significant
Intergenic	chr10 20598865-20598873	TGTTGGCGA	3.5850	NA	-5.7411	Significant
Intergenic	chr10 20663185-20663193	TGTTGGCGT	0.0000	NA	-8.4534	Significant
Intergenic	chr10 20781657-20781665	TGTTGGCGT	-0.1255	NA	-7.0772	Significant
NEBL	chr10 21256702-21256710	TGATGGCGA	5.0000	-0.2378	-6.7367	Significant
NEBL	chr10 21297583-21297591	TGTTGGCGT	5.0000	-0.2378	-16.9831	Significant
Intergenic	chr10 21658461-21658469	TCGCCTTCA	5.0000	NA	-9.3202	Significant
Intergenic	chr10 30239756-30239764	TGATGGCGT	1.5850	NA	-6.4699	Significant
ZNF438	chr10 31308390-31308398	TGATGGCGT	5.0000	-3.8062	-5.2533	Significant
ZEB1	chr10 31786613-31786621	TCGCCATCA	5.0000	0.0896	-10.9476	Significant
Intergenic	chr10 31829480-31829488	ACGCCTTCA	0.2224	NA	-8.2836	Significant
ARHGAP12	chr10 32106115-32106123	TGATGGCGA	-0.2801	-5.5829	-8.2834	Significant
Intergenic	chr10 32288858-32288866	TCGCCTACA	5.0000	NA	-9.8058	Significant
CCNY	chr10 35640984-35640992	TCGCCTTCA	-0.7885	-0.2164	-7.0772	Significant
Intergenic	chr10 43235058-43235066	TCGCCTACA	3.7004	NA	-7.2348	Significant
Intergenic	chr10 44096349-44096357	TGATGGCGA	0.6374	NA	-7.8722	Significant
Intergenic	chr10 45575925-45575933	ACGCCTACA	1.4150	NA	-5.7411	Significant
Intergenic	chr10 47025432-47025440	TCGCCTTCA	5.0000	NA	-9.1342	Significant

Intergenic	chr10	47086720-47086728	TGTAGGCGT	2.0000	NA	-6.8992	Significant
SGMS1	chr10	52280469-52280477	ACGCCAACA	5.0000	-3.8130	-7.6316	Significant
PRKG1	chr10	53203187-53203195	TGAAGGCGT	3.8074	0.1428	-7.4699	Significant
PRKG1	chr10	53871844-53871852	ACGCCATCA	5.0000	0.1428	-8.2802	Significant
Intergenic	chr10	55027786-55027794	TGTAGGCGT	5.0000	NA	-10.6711	Significant
PCDH15	chr10	55734495-55734503	TGATGGCGA	3.4594	0.3932	-4.4469	Significant
Intergenic	chr10	57163998-57164006	ACGCCAACA	5.0000	NA	-7.2352	Significant
ANK3	chr10	61836040-61836048	TGATGGCGT	1.1375	-0.4350	-11.6711	Significant
ANK3	chr10	62042400-62042408	ACGCCTTCA	5.0000	-0.4350	-10.7671	Significant
ARID5B	chr10	63815368-63815376	TCGCCTTCA	5.0000	-5.1368	-9.3124	Significant
JMJD1C	chr10	65202004-65202012	ACGCCAACA	5.0000	-1.7621	-9.0007	Significant
Intergenic	chr10	65467861-65467869	TGTTGGCGA	3.0875	NA	-6.3297	Significant
Intergenic	chr10	66233662-66233670	TGTTGGCGT	5.0000	NA	-13.0441	Significant
Intergenic	chr10	66298868-66298876	TGAAGGCGA	5.0000	NA	-12.4056	Significant
Intergenic	chr10	66980146-66980154	TCGCCAACA	1.8745	NA	-8.2838	Significant
Intergenic	chr10	67027946-67027954	TGTTGGCGT	5.0000	NA	-7.0823	Significant
Intergenic	chr10	67629995-67630003	TGAAGGCGA	1.8074	NA	-4.9165	Significant
CTNNA3	chr10	68979278-68979286	ACGCCAACA	5.0000	0.0209	-6.5211	Significant
HERC4	chr10	69791157-69791165	TCGCCAACA	5.0000	-0.8537	-6.7016	Significant
HERC4	chr10	69833861-69833869	TGATGGCGA	2.3219	-0.8537	-5.5546	Significant
HK1	chr10	71108200-71108208	TGAAGGCGT	5.0000	-0.3201	-7.0772	Significant
COL13A1	chr10	71615097-71615105	ACGCCTTCA	0.4854	-0.7034	-5.7407	Significant
SAR1A	chr10	71918124-71918132	TGTAGGCGT	3.5850	-0.1808	-6.3196	Significant
Intergenic	chr10	72657748-72657756	TCGCCAACA	2.8074	NA	-4.7567	Significant
Intergenic	chr10	72902888-72902896	TGATGGCGA	2.3219	NA	-8.2834	Significant
MCU	chr10	74476298-74476306	TGTAGGCGT	5.0000	-0.5526	-14.7050	Significant
Intergenic	chr10	74729016-74729024	TGTAGGCGT	5.0000	NA	-6.1002	Significant
FAM149B1	chr10	74929486-74929494	TGTTGGCGT	1.8074	-0.3236	-7.0825	Significant
Intergenic	chr10	75336616-75336624	TGATGGCGA	5.0000	NA	-8.9289	Significant
ZNF503	chr10	77084358-77084366	TGTAGGCGT	5.0000	0.2238	-7.6831	Significant
ZNF503-AS1	chr10	77084358-77084366	TGTAGGCGT	1.5850	0.0791	-8.0893	Significant
C10orf11	chr10	77837013-77837021	TGATGGCGA	1.2224	0.0390	-6.4691	Significant
POLR3A	chr10	79749464-79749472	TGTTGGCGT	0.6781	-2.1459	-7.8722	Significant
NRG3	chr10	83877661-83877669	TCGCCTTCA	0.8931	0.0709	-5.5994	Significant
Intergenic	chr10	86213258-86213266	TCGCCAACA	0.4594	NA	-7.4701	Significant
Intergenic	chr10	87181981-87181989	TGATGGCGA	1.3219	NA	-5.7926	Significant
Intergenic	chr10	88694763-88694771	ACGCCAACA	2.0000	NA	-4.3961	Significant
Intergenic	chr10	89816169-89816177	TGATGGCGT	5.0000	NA	-9.3124	Significant
RNLS	chr10	90034735-90034743	TCGCCATCA	5.0000	-0.0351	-8.2834	Significant
Intergenic	chr10	91592371-91592379	ACGCCTTCA	1.5850	NA	-14.7469	Significant
Intergenic	chr10	91673671-91673679	ACGCCTTCA	0.5850	NA	-7.0772	Significant
Intergenic	chr10	91745425-91745433	ACGCCTACA	-0.5850	NA	-13.1227	Significant
PCGF5	chr10	92924541-92924549	TCGCCTTCA	5.0000	-0.5481	-10.0223	Significant
Intergenic	chr10	97333286-97333294	TGAAGGCGA	5.0000	NA	-13.4241	Significant
LCOR	chr10	98604537-98604545	ACGCCTACA	5.0000	-3.1965	-8.9289	Significant
LCOR	chr10	98637952-98637960	TGTAGGCGT	3.7004	-3.1965	-8.8782	Significant
SLIT1	chr10	98871290-98871298	ACGCCTACA	3.1699	0.3021	-17.2828	Significant
SLIT1	chr10	98890063-98890071	ACGCCTTCA	3.3219	0.3021	-9.8058	Significant
CNNM1	chr10	101104569-101104577	TGAAGGCGA	5.0000	-1.8549	-7.2864	Significant
Intergenic	chr10	101192967-101192975	TGTTGGCGA	1.4150	NA	-7.4701	Significant
Intergenic	chr10	101388318-101388326	ACGCCTACA	3.4594	NA	-5.7407	Significant
LINC00263	chr10	102143328-102143336	TGAAGGCGT	3.0000	0.0785	-8.0891	Significant
Intergenic	chr10	103008429-103008437	ACGCCATCA	3.3219	NA	-10.7200	Significant
BTRC	chr10	103252403-103252411	TGAAGGCGT	-0.4150	-1.8437	-7.8720	Significant
NT5C2	chr10	104852646-104852654	ACGCCAACA	-0.4150	-0.5787	-8.8782	Significant
COL17A1	chr10	105842892-105842900	TGATGGCGT	1.4150	0.4347	-8.2834	Significant
SORCS3	chr10	106893212-106893220	TGTAGGCGA	3.1699	0.1594	-7.1102	Significant
Intergenic	chr10	107323631-107323639	TCGCCATCA	5.0000	NA	-10.6711	Significant
Intergenic	chr10	107622643-107622651	TGAAGGCGA	3.0000	NA	-5.9549	Significant
SORCS1	chr10	108412273-108412281	ACGCCATCA	2.7004	0.2158	-7.6316	Significant
SORCS1	chr10	108689349-108689357	ACGCCTACA	1.2630	0.2158	-7.6324	Significant
Intergenic	chr10	109823596-109823604	TGATGGCGA	5.0000	NA	-7.0772	Significant
Intergenic	chr10	110286711-110286719	TCGCCAACA	3.8074	NA	-5.9550	Significant
Intergenic	chr10	110978403-110978411	TGAAGGCGT	5.0000	NA	-9.1342	Significant
ADD3	chr10	111811707-111811715	ACGCCATCA	2.9069	-0.2049	-8.0891	Significant

ADD3	chr10	111851849-111851857	TGTTGGCGA	0.4854	-0.2049	-7.2348	Significant
RBM20	chr10	112406772-112406780	TGATGGCGA	2.5850	-0.1128	-8.2834	Significant
RBM20	chr10	112425063-112425071	TGATGGCGT	1.5025	-0.1128	-6.8477	Significant
RBM20	chr10	112523560-112523568	TGAAGGCGT	5.0000	-0.1128	-5.4423	Significant
Intergenic	chr10	113167729-113167737	ACGCCATCA	1.0000	NA	-5.5994	Significant
VTI1A	chr10	114286859-114286867	TCGCCTACA	3.3219	-2.3098	-14.4800	Significant
VTI1A	chr10	114575108-114575116	TGATGGCGA	1.8074	-2.3098	-12.9104	Significant
Intergenic	chr10	115117522-115117530	TGATGGCGA	2.0000	NA	-6.8473	Significant
Intergenic	chr10	115303028-115303036	TCGCCAACA	0.1926	NA	-6.8990	Significant
NRAP	chr10	115373404-115373412	ACGCCTACA	3.7004	-0.1108	-9.5737	Significant
Intergenic	chr10	115518398-115518406	TCGCCTTCA	3.7004	NA	-8.0891	Significant
Intergenic	chr10	115551675-115551683	TCGCCATCA	1.3219	NA	-6.1520	Significant
TDRD1	chr10	115955818-115955826	ACGCCTTCA	2.3219	0.2463	-7.6835	Significant
Intergenic	chr10	116782726-116782734	TGAAGGCGT	0.5850	NA	-9.1344	Significant
Intergenic	chr10	118295508-118295516	TGTAGGCGT	0.3219	NA	-6.3198	Significant
Intergenic	chr10	121932986-121932994	ACGCCTACA	5.0000	NA	-16.9461	Significant
Intergenic	chr10	122189396-122189403	ACGCCATCA	1.5850	NA	-4.7182	Significant
Intergenic	chr10	122189396-122189400	ACGCCATCA	0.0000	NA	-7.0772	Significant
Intergenic	chr10	122710417-122710425	TCGCCTTCA	1.5850	NA	-9.7560	Significant
TACC2	chr10	124005145-124005153	TGTTGGCGA	3.3219	-3.2911	-12.1603	Significant
BTBD16	chr10	124043772-124043780	TCGCCAACA	3.1699	0.2014	-5.4421	Significant
Intergenic	chr10	124452859-124452867	TGATGGCGT	5.0000	NA	-5.0497	Significant
Intergenic	chr10	124723389-124723396	TGAAGGCGA	3.3219	NA	-5.0497	Significant
CPXM2	chr10	125608285-125608293	TCGCCTTCA	3.0000	-0.9038	-7.4701	Significant
METTL10	chr10	126450742-126450750	ACGCCATCA	2.3219	-0.6127	-6.6939	Significant
Intergenic	chr10	130579545-130579553	TGAAGGCGT	3.0000	NA	-7.2799	Significant
Intergenic	chr10	130976933-130976941	TGAAGGCGT	5.0000	NA	-10.2581	Significant
EBF3	chr10	131744895-131744903	TGTTGGCGT	5.0000	-0.4181	-9.7560	Significant
Intergenic	chr10	133380891-133380899	ACGCCAACA	-1.1155	NA	-6.4695	Significant
KNDC1	chr10	135005851-135005859	ACGCCTACA	5.0000	0.2573	-5.3910	Significant
Intergenic	chr11	2276846-2276854	TGAAGGCGT	3.3219	NA	-6.0164	Significant
KCNQ1	chr11	2779638-2779646	TGTTGGCGA	5.0000	0.1746	-5.7924	Significant
STIM1	chr11	3943668-3943676	TGTAGGCGT	5.0000	-0.4837	-6.1002	Significant
Intergenic	chr11	5220497-5220505	TCGCCATCA	0.7370	NA	-8.0891	Significant
Intergenic	chr11	6021767-6021775	ACGCCAACA	-0.3370	NA	-4.3965	Significant
Intergenic	chr11	6155105-6155113	TCGCCTACA	1.0000	NA	-8.7041	Significant
NRIP3	chr11	9019156-9019164	TCGCCATCA	2.1155	0.4099	-5.7411	Significant
LOC644656	chr11	9481734-9481742	ACGCCTTCA	-0.5850	0.0482	-9.3128	Significant
Promoter_ZNF143	chr11	9481734-9481742	ACGCCTTCA	0.4150	-2.4767	-20.5891	Significant
Intergenic	chr11	9646963-9646971	ACGCCATCA	5.0000	NA	-4.2573	Marginal
SWAP70	chr11	9715000-9715008	TGTTGGCGA	2.8074	-2.4738	-4.9166	Significant
SWAP70	chr11	9759804-9759812	ACGCCTTCA	5.0000	-2.4738	-6.1002	Significant
SBF2	chr11	10043048-10043056	TGAAGGCGA	-1.2224	-0.9222	-8.2834	Significant
RNF141	chr11	10536575-10536583	TGTAGGCGA	5.0000	-0.1756	-21.8765	Significant
Intergenic	chr11	10718488-10718496	TGAAGGCGA	0.1699	NA	-5.3910	Significant
Intergenic	chr11	10905757-10905765	TGATGGCGA	0.3219	NA	-8.8786	Significant
Intergenic	chr11	12593897-12593905	TGATGGCGA	1.3219	NA	-7.2352	Significant
Intergenic	chr11	15806354-15806362	ACGCCAACA	0.7776	NA	-12.9104	Significant
PLEKHA7	chr11	16925045-16925053	ACGCCTACA	1.4594	-2.1571	-7.4701	Significant
Intergenic	chr11	17056434-17056442	TGTAGGCGA	0.4854	NA	-8.8786	Significant
NELL1	chr11	20727347-20727355	ACGCCAACA	5.0000	0.3838	-7.6316	Significant
SLC17A6	chr11	22378244-22378252	TGTTGGCGT	1.0000	0.2783	-9.1342	Significant
Intergenic	chr11	24198569-24198577	TCGCCTTCA	-0.4150	NA	-6.6938	Significant
Intergenic	chr11	24392807-24392815	TGTAGGCGT	-1.1255	NA	-7.4699	Significant
BBOX1	chr11	27113627-27113635	ACGCCTACA	0.2630	0.0116	-5.9550	Significant
LGR4	chr11	27390161-27390169	TGATGGCGT	3.3219	-4.1261	-6.1002	Significant
LGR4	chr11	27477273-27477281	ACGCCTACA	2.5850	-4.1261	-6.6938	Significant
Intergenic	chr11	30109757-30109765	TGAAGGCGT	1.4594	NA	-6.4691	Significant
Intergenic	chr11	30197930-30197938	TCGCCTACA	3.1699	NA	-5.7415	Significant
IMMP1L	chr11	31479548-31479556	ACGCCATCA	1.8745	-1.2241	-9.5736	Significant
WT1	chr11	32426680-32426688	TGAAGGCGA	5.0000	-0.8536	-6.3196	Significant
CCDC73	chr11	32633598-32633606	TCGCCTACA	5.0000	0.1948	-5.5549	Significant
CD59	chr11	33734610-33734618	ACGCCATCA	5.0000	0.0981	-20.5987	Significant
CD59	chr11	33748996-33749004	ACGCCTTCA	3.8074	0.0981	-13.6413	Significant
Intergenic	chr11	33937360-33937368	TCGCCATCA	0.7370	NA	-6.6941	Significant

CAT	chr11	34465703-34465711	TGTTGGCGA	5.0000	0.2386	-9.2913	Significant
Intergenic	chr11	35643074-35643082	TGAAGGCGT	3.1699	NA	-8.2834	Significant
Intergenic	chr11	36794087-36794095	TCGCCATCA	-0.3626	NA	-8.0378	Significant
Intergenic	chr11	37509620-37509628	ACGCCAACA	-0.2630	NA	-8.4534	Significant
Intergenic	chr11	37963591-37963599	TGTAGGCGT	1.8074	NA	-5.9552	Significant
Intergenic	chr11	39674350-39674358	ACGCCTTCA	2.0000	NA	-8.2834	Significant
LRRC4C	chr11	40357669-40357677	TGATGGCGA	1.9260	0.0594	-6.8473	Significant
Intergenic	chr11	42942369-42942377	TCGCCTTCA	3.5850	NA	-10.9478	Significant
Intergenic	chr11	44084581-44084589	TCGCCTTCA	1.4594	NA	-6.8990	Significant
EXT2	chr11	44144012-44144020	TCGCCATCA	5.0000	-0.0298	-8.2836	Significant
Intergenic	chr11	44357708-44357716	TGATGGCGT	1.5850	NA	-8.0891	Significant
CRY2	chr11	45899428-45899436	TGTAGGCGA	1.2630	-2.4909	-8.7043	Significant
PHF21A	chr11	46045315-46045323	TCGCCAACA	0.3219	-0.6821	-5.7407	Significant
CREB3L1	chr11	46321042-46321050	TGTTGGCGT	5.0000	-0.0724	-7.6316	Significant
AMBRA1	chr11	46518524-46518532	TGATGGCGA	1.7370	-2.6242	-7.0772	Significant
C11orf49	chr11	47146901-47146909	ACGCCTACA	0.8931	-0.1878	-6.1522	Significant
CELF1	chr11	47573125-47573133	ACGCCTTCA	5.0000	-1.0366	-9.5737	Significant
NUP160	chr11	47825541-47825549	TCGCCAACA	5.0000	-2.2939	-10.0223	Significant
NUP160	chr11	47858535-47858543	TGAAGGCGA	5.0000	-2.2939	-10.0223	Significant
Intergenic	chr11	48349582-48349590	TGATGGCGA	-0.6781	NA	-4.4380	Significant
Intergenic	chr11	48355101-48355109	ACGCCAACA	5.0000	NA	-6.8990	Significant
Intergenic	chr11	58258558-58258566	TCGCCAACA	5.0000	NA	-4.9165	Significant
OR4D10	chr11	59245061-59245069	TCGCCTTCA	0.1255	-0.0013	-8.0378	Significant
Intergenic	chr11	59318996-59319004	TGAAGGCGT	-0.2224	NA	-12.6134	Significant
Intergenic	chr11	59520646-59520654	TGTAGGCGT	5.0000	NA	-7.6320	Significant
Intergenic	chr11	59643874-59643882	TGAAGGCGA	5.0000	NA	-8.0891	Significant
Intergenic	chr11	59783971-59783979	ACGCCTTCA	-0.1375	NA	-5.7407	Significant
MS4A3	chr11	59838320-59838328	TGTTGGCGA	3.0000	0.3434	-5.7407	Significant
DDB1	chr11	61088993-61089001	ACGCCTTCA	2.7004	0.1228	-7.6316	Significant
Intergenic	chr11	62813556-62813564	TGTTGGCGA	1.1155	NA	-6.1522	Significant
RTN3	chr11	63481242-63481250	TGATGGCGA	5.0000	0.2123	-12.6134	Significant
MALAT1	chr11	65266949-65266957	TGAAGGCGA	5.0000	-1.5933	-13.1223	Significant
MALAT1	chr11	65272599-65272607	TGTTGGCGT	-0.2410	-1.5933	-7.8720	Significant
PACS1	chr11	65932728-65932736	TGAAGGCGT	1.0000	-0.1225	-15.6865	Significant
RBM4	chr11	66415218-66415226	TGTTGGCGA	1.3219	-0.0024	-7.4701	Significant
KDM2A	chr11	66977694-66977702	TGATGGCGT	3.1699	-1.9954	-8.0382	Significant
Intergenic	chr11	67469213-67469221	TCGCCAACA	3.3219	NA	-7.6316	Significant
CPT1A	chr11	68532859-68532867	ACGCCATCA	3.0875	-0.1293	-9.3627	Significant
PPFIA1	chr11	70147171-70147179	TCGCCTACA	-0.1255	-1.8378	-6.3198	Significant
Intergenic	chr11	70304687-70304695	TCGCCATCA	2.3219	NA	-5.5999	Significant
SHANK2	chr11	70766935-70766943	TGAAGGCGA	0.4854	-0.2889	-8.5043	Significant
Intergenic	chr11	71218048-71218056	TCGCCATCA	3.0000	NA	-8.7041	Significant
NUMA1	chr11	71735145-71735152	TGAAGGCGA	3.5850	-0.9683	-9.3124	Significant
CLPB	chr11	72073708-72073716	ACGCCTTCA	5.0000	0.1049	-6.4695	Significant
P2RY6	chr11	72996263-72996271	ACGCCAACA	-0.4854	-0.1819	-4.5681	Significant
Intergenic	chr11	73652495-73652503	ACGCCATCA	1.5850	NA	-6.4691	Significant
Intergenic	chr11	74207968-74207976	ACGCCAACA	1.4150	NA	-5.5996	Significant
RSF1	chr11	77496444-77496452	TCGCCAACA	1.0000	-2.9853	-7.2348	Significant
INTS4	chr11	77676982-77676990	TCGCCAACA	5.0000	-0.4162	-9.5737	Significant
Intergenic	chr11	77794112-77794120	TCGCCTTCA	2.9069	NA	-6.6939	Significant
NARS2	chr11	78171226-78171234	TGTAGGCGA	3.1699	-0.2113	-8.2802	Significant
Intergenic	chr11	78992124-78992132	TGATGGCGT	-1.8365	NA	-8.8786	Significant
Intergenic	chr11	81196889-81196897	ACGCCATCA	0.5146	NA	-5.5996	Significant
RAB30	chr11	82728243-82728251	TGTTGGCGT	3.0000	-0.9892	-5.5996	Significant
Intergenic	chr11	82901809-82901817	TGTTGGCGA	1.7370	NA	-9.8058	Significant
DLG2	chr11	84369419-84369427	TGATGGCGT	5.0000	0.0758	-6.7014	Significant
DLG2	chr11	84600382-84600389	TGAAGGCGT	1.5850	0.0758	-10.9247	Significant
CCDC83	chr11	85572736-85572744	ACGCCTTCA	1.1375	0.0090	-5.5994	Significant
Intergenic	chr11	87189274-87189282	ACGCCAACA	1.7370	NA	-10.6711	Significant
CTSC	chr11	88033146-88033154	ACGCCATCA	2.4594	-0.2272	-13.6844	Significant
GRM5	chr11	88448250-88448258	TCGCCTTCA	3.0000	0.1504	-9.1249	Significant
GRM5	chr11	88486622-88486630	TGATGGCGA	5.0000	0.1504	-8.0378	Significant
NOX4	chr11	89321953-89321961	TGTAGGCGT	1.1699	0.0991	-7.6316	Significant
Intergenic	chr11	90463192-90463200	TGTTGGCGT	-1.0000	NA	-6.4691	Significant
Intergenic	chr11	94109929-94109937	TCGCCTACA	5.0000	NA	-8.0378	Significant

CEP57	chr11	95557272-95557280	TGTTGGCGT	5.0000	-1.6979	-6.6938	Significant
MTMR2	chr11	95612796-95612804	TCGCCAACA	2.8074	-0.3914	-10.9247	Significant
Intergenic	chr11	97876322-97876330	TCGCCAACA	5.0000	NA	-7.6316	Significant
CNTN5	chr11	99576713-99576721	ACGCCATCA	5.0000	0.0910	-15.8069	Significant
CNTN5	chr11	99828412-99828420	TGAAGGCGT	-1.3626	0.0910	-5.7407	Significant
CNTN5	chr11	100162994-100163002	TGATGGCGT	0.0000	0.0910	-6.7014	Significant
Intergenic	chr11	101490801-101490809	TGAAGGCGA	5.0000	NA	-6.2573	Significant
Intergenic	chr11	102151796-102151804	ACGCCAACA	5.0000	NA	-4.8879	Significant
Intergenic	chr11	102252655-102252663	TGTTGGCGT	5.0000	NA	-6.6221	Significant
MMP3	chr11	102707891-102707899	TGTAGGCGA	3.3219	0.1628	-5.3906	Significant
Intergenic	chr11	105381231-105381239	TGTTGGCGA	5.0000	NA	-10.0223	Significant
GRIA4	chr11	105806265-105806273	TGAAGGCGT	0.2410	-0.0225	-4.9168	Significant
Intergenic	chr11	106044866-106044874	ACGCCTTCA	3.9069	NA	-10.0224	Significant
Intergenic	chr11	106374287-106374295	TGATGGCGT	3.1699	NA	-5.2533	Significant
CWF19L2	chr11	107205498-107205506	TCGCCTTCA	1.7370	-1.3394	-10.0223	Significant
ATM	chr11	108126426-108126434	TGTAGGCGT	2.4594	-0.3591	-11.6236	Significant
C11orf65	chr11	108332493-108332501	TGTAGGCGT	1.4594	-0.3042	-13.6405	Significant
DDX10	chr11	108657948-108657956	TGATGGCGT	-0.4475	-4.2324	-7.0774	Significant
Intergenic	chr11	108829792-108829800	TCGCCTACA	1.8074	NA	-11.1907	Significant
Intergenic	chr11	109677997-109678005	TCGCCAACA	2.5850	NA	-6.7014	Significant
USP28	chr11	113702479-113702487	TGAAGGCGA	0.0000	-0.1226	-8.8633	Significant
Intergenic	chr11	114200491-114200499	TCGCCAACA	0.0000	NA	-8.8633	Significant
CADM1	chr11	115106330-115106338	ACGCCTACA	5.0000	-0.0037	-10.2583	Significant
Intergenic	chr11	116166103-116166111	TGTTGGCGT	5.0000	NA	-5.7924	Significant
SIK3	chr11	116817174-116817182	ACGCCTACA	5.0000	-2.6652	-9.5736	Significant
SIK3	chr11	116821746-116821754	TGAAGGCGT	5.0000	-2.6652	-7.8722	Significant
Intergenic	chr11	118711267-118711275	TGAAGGCGA	0.6781	NA	-10.9578	Significant
CBL	chr11	119137465-119137473	TGAAGGCGA	0.0000	-2.0313	-6.8992	Significant
CBL	chr11	119142440-119142448	TGTAGGCGA	1.4854	-2.0313	-7.8717	Significant
POU2F3	chr11	120162189-120162197	TGATGGCGA	0.2895	-0.0430	-10.2581	Significant
POU2F3	chr11	120189014-120189022	ACGCCATCA	1.8074	-0.0430	-6.1522	Significant
SORL1	chr11	121444686-121444694	TCGCCAACA	2.3219	0.0583	-7.2864	Significant
SORL1	chr11	121471483-121471491	TGAAGGCGA	3.3219	0.0583	-5.5994	Significant
Intergenic	chr11	121793333-121793341	TGATGGCGT	-0.5305	NA	-14.1681	Significant
Intergenic	chr11	121881841-121881849	ACGCCAACA	1.7370	NA	-7.6835	Significant
MIR100HG	chr11	121984018-121984026	TGATGGCGA	4.3219	0.1281	-7.4699	Significant
Intergenic	chr11	122119877-122119885	TCGCCTTCA	0.8301	NA	-6.4691	Significant
Intergenic	chr11	122176641-122176649	ACGCCTTCA	3.4594	NA	-6.8477	Significant
Intergenic	chr11	124272606-124272614	TGTAGGCGA	5.0000	NA	-15.5129	Significant
Intergenic	chr11	124391143-124391151	ACGCCAACA	5.0000	NA	-7.6316	Significant
CCDC15	chr11	124834826-124834834	TGATGGCGT	5.0000	-0.4950	-10.2581	Significant
ST3GAL4	chr11	126241207-126241215	ACGCCATCA	-0.4150	-1.6557	-7.0772	Significant
ZBTB44	chr11	130109773-130109781	ACGCCTTCA	2.7370	-0.6932	-14.4802	Significant
Intergenic	chr11	130800571-130800579	ACGCCATCA	1.5850	NA	-11.9103	Significant
Intergenic	chr11	130851630-130851638	TGAAGGCGT	5.0000	NA	-4.9937	Significant
Intergenic	chr11	131154756-131154764	ACGCCATCA	1.2895	NA	-6.6938	Significant
NTM	chr11	132099334-132099342	TGAAGGCGT	-1.0000	0.0188	-5.4423	Significant
Intergenic	chr11	132284579-132284587	TCGCCTACA	0.5850	NA	-8.2836	Significant
OPCML	chr11	132746486-132746494	ACGCCAACA	0.4594	0.0021	-6.8990	Significant
Intergenic	chr11	133659503-133659511	TGATGGCGA	2.0000	NA	-8.6980	Significant
IQSEC3	chr12	182431-182439	TCGCCTTCA	5.0000	0.1772	-11.9103	Significant
IQSEC3	chr12	215919-215927	TGTAGGCGT	3.0000	0.1772	-12.6591	Significant
WNK1	chr12	890361-890369	ACGCCTACA	0.1155	-1.0570	-6.8992	Significant
ERC1	chr12	1317841-1317849	TGTTGGCGT	5.0000	-2.1176	-10.2581	Significant
ERC1	chr12	1484169-1484177	TGTAGGCGT	2.5850	-2.1176	-9.1342	Significant
Intergenic	chr12	1777044-1777052	TGAAGGCGT	5.0000	NA	-8.2834	Significant
PRMT8	chr12	3510839-3510847	ACGCCAACA	-0.2224	-0.1102	-5.5996	Significant
C12orf4	chr12	4616049-4616057	TGTAGGCGT	5.0000	-2.4948	-14.2110	Significant
RAD51AP1	chr12	4667484-4667492	TGTAGGCGA	5.0000	-0.2386	-8.6982	Significant
DYRK4	chr12	4709969-4709977	TGAAGGCGT	0.8845	-0.4157	-4.5890	Significant
DYRK4	chr12	4720740-4720748	TGTAGGCGA	0.5146	-0.4157	-9.5736	Significant
Intergenic	chr12	5099618-5099626	TGTAGGCGT	0.8480	NA	-5.7924	Significant
Intergenic	chr12	5304590-5304598	ACGCCATCA	2.0000	NA	-5.9552	Significant
Intergenic	chr12	7903655-7903663	ACGCCTTCA	5.0000	NA	-5.3906	Significant
Intergenic	chr12	7909761-7909769	TGTAGGCGT	3.4594	NA	-6.8992	Significant

SLC2A3	chr12 8079859-8079867	TGATGGCGT	2.3219	0.1968	-12.9102	Significant
CLECL1	chr12 9884162-9884170	TGATGGCGA	2.1155	0.1636	-6.5211	Significant
Intergenic	chr12 10991886-10991894	TGAAGGCGT	2.3219	NA	-4.6093	Significant
ETV6	chr12 11939144-11939152	ACGCCATCA	2.7004	-3.3668	-6.4691	Significant
ETV6	chr12 11960248-11960256	ACGCCAACA	2.1699	-3.3668	-8.0378	Significant
DUSP16	chr12 12633197-12633205	TGTAGGCGA	4.7549	-1.2721	-15.0219	Significant
DUSP16	chr12 12640590-12640598	TGATGGCGT	2.8074	-1.2721	-7.2348	Significant
Intergenic	chr12 13388266-13388274	TGAAGGCGA	5.0000	NA	-4.7693	Significant
Intergenic	chr12 13426634-13426642	ACGCCAACA	5.0000	NA	-11.9103	Significant
GRIN2B	chr12 13922632-13922640	TCGCCTTCA	5.0000	-1.0129	-13.4241	Significant
PLBD1	chr12 14679945-14679953	ACGCCTTCA	2.2224	0.0588	-6.8473	Significant
PTPRO	chr12 15596775-15596783	TGTTGGCGA	2.1699	-1.5666	-4.7194	Significant
EPS8	chr12 15783660-15783668	TGATGGCGA	1.7370	-1.1610	-4.9166	Significant
SLC15A5	chr12 16404481-16404489	ACGCCAACA	-0.4854	0.1031	-7.2348	Significant
Intergenic	chr12 16935552-16935560	ACGCCATCA	5.0000	NA	-6.1002	Significant
Intergenic	chr12 17111069-17111077	TGATGGCGA	3.5850	NA	-9.1342	Significant
Intergenic	chr12 17785676-17785684	TCGCCAACA	5.0000	NA	-8.2836	Significant
Intergenic	chr12 18046005-18046013	TGTTGGCGA	3.5850	NA	-8.2838	Significant
Intergenic	chr12 18049266-18049274	TGTTGGCGT	1.8074	NA	-7.0772	Significant
PIK3C2G	chr12 18593950-18593958	ACGCCAACA	3.1699	-0.0249	-8.0891	Significant
Intergenic	chr12 19063230-19063238	TGTAGGCGA	-0.1926	NA	-8.2836	Significant
AEBP2	chr12 19608200-19608208	TGTAGGCGT	5.0000	-3.8858	-10.6711	Significant
Intergenic	chr12 19695500-19695508	TGTTGGCGT	5.0000	NA	-6.8473	Significant
LOC100506393	chr12 20242033-20242041	TGTTGGCGA	3.7004	0.3222	-9.3124	Significant
Intergenic	chr12 22191542-22191550	TCGCCAACA	3.3219	NA	-10.0223	Significant
ST8SIA1	chr12 22370317-22370325	TGATGGCGT	-0.1806	-0.0901	-7.6831	Significant
Intergenic	chr12 22648329-22648337	ACGCCAACA	5.0000	NA	-6.8990	Significant
Intergenic	chr12 22678999-22679007	ACGCCATCA	0.0000	NA	-5.7407	Significant
Intergenic	chr12 22857388-22857396	TGTTGGCGA	5.0000	NA	-6.1002	Significant
SOX5	chr12 23717965-23717973	TGTAGGCGA	5.0000	0.1796	-5.0845	Significant
SOX5	chr12 24443781-24443789	TGAAGGCGA	1.3785	0.1796	-7.0775	Significant
KRAS	chr12 25398278-25398286	ACGCCATCA	0.0000	-0.9000	-8.7041	Significant
ITPR2	chr12 26883162-26883170	ACGCCATCA	0.5850	-0.9298	-12.6138	Significant
ARNTL2	chr12 27524143-27524151	TGATGGCGT	5.0000	0.0735	-7.8720	Significant
PPFIBP1	chr12 27702812-27702820	TGAAGGCGA	5.0000	-1.4142	-9.1342	Significant
PPFIBP1	chr12 27776244-27776252	TCGCCTACA	1.4594	-1.4142	-7.2352	Significant
Intergenic	chr12 28096282-28096290	TGAAGGCGA	1.7370	NA	-5.5994	Significant
Intergenic	chr12 28282450-28282458	TGATGGCGT	5.0000	NA	-7.8720	Significant
CCDC91	chr12 28428063-28428071	TGAAGGCGA	5.0000	-0.7374	-13.6844	Significant
TMTC1	chr12 29664386-29664394	TGATGGCGA	2.7004	-1.0167	-5.7407	Significant
Intergenic	chr12 31299641-31299649	ACGCCTTCA	-0.3219	NA	-10.0224	Significant
AMN1	chr12 31836610-31836618	TCGCCTTCA	-0.7776	-0.0866	-11.6240	Significant
Intergenic	chr12 32258019-32258027	ACGCCATCA	4.0000	NA	-7.4699	Significant
FGD4	chr12 32715115-32715123	TCGCCTACA	0.4150	-3.0082	-5.0497	Significant
Intergenic	chr12 33210996-33211004	TGATGGCGA	5.0000	NA	-8.7041	Significant
CPNE8	chr12 39214015-39214023	TCGCCAACA	0.8480	0.0777	-5.7407	Significant
ABCD2	chr12 39965412-39965420	TGTAGGCGT	5.0000	-0.0522	-15.8069	Significant
Intergenic	chr12 42105318-42105326	TGTTGGCGA	3.5850	NA	-8.9293	Significant
YAF2	chr12 42570724-42570732	ACGCCATCA	1.7370	-1.1616	-6.8473	Significant
PRICKLE1	chr12 42962553-42962561	TGATGGCGA	5.0000	-0.0002	-4.3965	Significant
Intergenic	chr12 43993118-43993126	ACGCCAACA	4.0444	NA	-9.1345	Significant
Intergenic	chr12 45340576-45340584	ACGCCTACA	-0.9069	NA	-9.3128	Significant
Intergenic	chr12 46543202-46543210	TGTTGGCGT	0.7776	NA	-8.8782	Significant
Intergenic	chr12 47515018-47515026	TGTAGGCGA	2.0000	NA	-7.6320	Significant
Intergenic	chr12 48035993-48036001	TGTTGGCGT	1.8074	NA	-9.3128	Significant
Intergenic	chr12 48040402-48040410	TCGCCTTCA	-0.7370	NA	-5.1015	Significant
Intergenic	chr12 48327780-48327788	TGTTGGCGA	1.0000	NA	-6.5209	Significant
SPATS2	chr12 49874350-49874358	ACGCCTACA	0.5850	-1.4492	-4.7695	Significant
Intergenic	chr12 50704150-50704158	TCGCCATCA	5.0000	NA	-17.5653	Significant
SCN8A	chr12 52035907-52035915	TCGCCATCA	0.2895	0.3035	-5.7407	Significant
SCN8A	chr12 52157154-52157162	TGTAGGCGA	1.8074	0.3035	-6.8473	Significant
Intergenic	chr12 52746911-52746919	TGAAGGCGT	3.7004	NA	-11.1907	Significant
ATF7	chr12 53958698-53958706	TGTTGGCGT	1.7370	-0.2139	-6.8473	Significant
Intergenic	chr12 54166855-54166863	TGTTGGCGA	5.0000	NA	-10.6711	Significant
Promoter_HOXC12	chr12 54348326-54348334	TGTTGGCGA	0.8074	0.4113	-16.3718	Significant

FLJ12825	chr12 54491346-54491354	ACGCCATCA	3.5850	-0.1574	-5.7407	Significant
Intergenic	chr12 54559658-54559666	TCGCCTACA	5.0000	NA	-8.9289	Significant
Intergenic	chr12 54885891-54885899	TGAAGGCGA	5.0000	NA	-7.4727	Significant
TIMELESS	chr12 56837461-56837469	TCGCCATCA	-1.3219	-0.0383	-5.1011	Significant
TMEM194A	chr12 57458856-57458864	ACGCCTTCA	-0.1520	-0.8143	-9.3128	Significant
Promoter_TMEM194A	chr12 57472966-57472974	ACGCCTACA	5.0000	-0.8143	-12.4056	Significant
R3HDM2	chr12 57698036-57698044	TGAAGGCGT	5.0000	-0.8796	-7.6831	Significant
Intergenic	chr12 58898094-58898102	TGATGGCGT	3.4594	NA	-6.1006	Significant
Intergenic	chr12 61567556-61567564	TGAAGGCGA	0.5850	NA	-4.5890	Significant
Intergenic	chr12 61803718-61803726	ACGCCATCA	3.4594	NA	-13.4241	Significant
Intergenic	chr12 61983107-61983115	TCGCCTTCA	2.0000	NA	-6.3198	Significant
FAM19A2	chr12 62539672-62539680	TGTTGGCGA	2.5850	0.0052	-9.3124	Significant
PPM1H	chr12 63043815-63043823	TCGCCTTCA	2.3219	-0.9389	-10.2581	Significant
Intergenic	chr12 63400513-63400521	TGTTGGCGT	0.4475	NA	-10.6711	Significant
Intergenic	chr12 64221177-64221185	ACGCCTTCA	5.0000	NA	-7.0772	Significant
Intergenic	chr12 64995221-64995229	ACGCCATCA	2.1699	NA	-5.9550	Significant
Intergenic	chr12 65106949-65106957	TGAAGGCGA	-0.5850	NA	-4.9165	Significant
Intergenic	chr12 65181624-65181632	TCGCCAACA	1.8745	NA	-5.9549	Significant
TBC1D30	chr12 65271017-65271025	TCGCCATCA	5.0000	-0.4105	-8.0378	Significant
TBC1D30	chr12 65271399-65271407	ACGCCTACA	0.7370	-0.4105	-6.6938	Significant
WIF1	chr12 65445183-65445191	TGAAGGCGT	2.0000	0.1529	-4.5893	Significant
WIF1	chr12 65458630-65458638	ACGCCTACA	1.5850	0.1529	-5.3906	Significant
MSRB3	chr12 65806811-65806819	TGATGGCGA	2.8074	0.0508	-5.3910	Significant
IRAK3	chr12 66632317-66632325	TGTTGGCGT	1.8074	-0.6411	-7.4699	Significant
GRIP1	chr12 67015391-67015399	TGTAGGCGT	5.0000	0.1116	-6.1002	Significant
Intergenic	chr12 68206215-68206223	TGTAGGCGA	1.1155	NA	-7.6316	Significant
Intergenic	chr12 68331490-68331498	TGATGGCGA	5.0000	NA	-5.4213	Significant
Intergenic	chr12 69605822-69605829	ACGCCTACA	5.0000	NA	-9.1342	Significant
YEATS4	chr12 69771722-69771730	TGTTGGCGA	2.8074	-2.9761	-5.0501	Significant
CNOT2	chr12 70723295-70723303	ACGCCAACA	1.5850	-2.6417	-6.5209	Significant
Intergenic	chr12 72584023-72584031	TCGCCATCA	1.0000	NA	-8.7041	Significant
TRHDE	chr12 72794174-72794182	ACGCCTACA	5.0000	0.1120	-6.6939	Significant
Intergenic	chr12 73532216-73532224	TGAAGGCGT	5.0000	NA	-5.4421	Significant
Intergenic	chr12 75629543-75629551	TGTTGGCGT	5.0000	NA	-15.9822	Significant
Intergenic	chr12 76008729-76008737	ACGCCAACA	1.6781	NA	-8.0378	Significant
Intergenic	chr12 76625429-76625437	TGTAGGCGT	0.7370	NA	-5.2699	Significant
OSBPL8	chr12 76937759-76937767	TCGCCAACA	2.3219	0.1862	-7.4727	Significant
Intergenic	chr12 77740061-77740069	TGTTGGCGA	1.2224	NA	-5.0497	Significant
Intergenic	chr12 77860046-77860054	TGTTGGCGA	5.0000	NA	-10.2583	Significant
NAV3	chr12 78274266-78274274	ACGCCATCA	-1.1155	-0.9553	-7.8720	Significant
NAV3	chr12 78282914-78282922	TGAAGGCGT	2.3219	-0.9553	-8.2836	Significant
SYT1	chr12 79386400-79386409	TCGCCTACA	0.8480	-0.0822	-5.5996	Significant
SYT1	chr12 79612793-79612801	TCGCCAACA	5.0000	-0.0822	-11.6236	Significant
Intergenic	chr12 80163278-80163286	TGTAGGCGT	1.3219	NA	-6.8473	Significant
Intergenic	chr12 80384865-80384873	ACGCCAACA	-0.1255	NA	-4.7182	Significant
Intergenic	chr12 80594718-80594726	ACGCCTACA	1.2630	NA	-6.1520	Significant
PPFIA2	chr12 81676361-81676369	ACGCCAACA	2.4594	0.2627	-12.1138	Significant
PPFIA2	chr12 81691592-81691600	TCGCCTACA	5.0000	0.2627	-5.7924	Significant
Intergenic	chr12 82425339-82425347	TGATGGCGA	3.8074	NA	-21.9009	Significant
TMTC2	chr12 83178415-83178423	TGTAGGCGA	5.0000	-1.4868	-6.3300	Significant
Intergenic	chr12 85245187-85245195	ACGCCAACA	2.5850	NA	-10.6711	Significant
Intergenic	chr12 85978011-85978019	TGATGGCGA	1.5850	NA	-7.0774	Significant
Intergenic	chr12 88230928-88230936	TCGCCTACA	0.2224	NA	-6.6938	Significant
C12orf50	chr12 88383886-88383894	ACGCCATCA	3.4594	-0.1302	-7.2348	Significant
C12orf29	chr12 88442016-88442024	TCGCCATCA	3.7004	-0.4953	-17.5309	Significant
Intergenic	chr12 88673977-88673985	TGATGGCGA	3.5850	NA	-7.2348	Significant
Promoter_KITLG	chr12 88974915-88974923	TCGCCAACA	0.4594	-4.3448	-5.3906	Significant
Intergenic	chr12 89491742-89491750	TGATGGCGT	1.8745	NA	-13.6844	Significant
Intergenic	chr12 89715253-89715261	ACGCCTTCA	2.4150	NA	-5.7407	Significant
Intergenic	chr12 90548718-90548726	TCGCCTACA	-0.1699	NA	-4.2929	Marginal
Intergenic	chr12 90598991-90598999	ACGCCTTCA	-1.1844	NA	-4.5890	Significant
Intergenic	chr12 90732830-90732838	TGTTGGCGT	3.7004	NA	-10.4804	Significant
Intergenic	chr12 91352817-91352825	TGATGGCGT	1.0000	NA	-5.9549	Significant
Intergenic	chr12 92035232-92035240	TCGCCATCA	5.0000	NA	-14.4800	Significant
Intergenic	chr12 92070946-92070954	TCGCCTTCA	5.0000	NA	-10.2581	Significant

Intergenic	chr12	92978361-92978369	TCGCCAACA	5.0000	NA	-7.8720	Significant
EEA1	chr12	93261008-93261016	ACGCCATCA	3.0000	-2.7954	-13.6409	Significant
LOC643339	chr12	93419251-93419259	TGATGGCGA	2.5850	0.0835	-4.7695	Significant
NUDT4	chr12	93781657-93781665	ACGCCATCA	3.3219	-2.0638	-4.6093	Significant
Intergenic	chr12	94372669-94372677	TCGCCATCA	1.0995	NA	-5.2534	Significant
PLXNC1	chr12	94561835-94561843	TGTTGGCGA	5.0000	0.0861	-19.6931	Significant
Intergenic	chr12	94895108-94895116	TGAAGGCGA	3.1699	NA	-8.5043	Significant
VEZT	chr12	95625027-95625035	TGAAGGCGT	2.9069	-1.9560	-8.0382	Significant
AMDHD1	chr12	96355414-96355422	ACGCCATCA	5.0000	0.6804	-12.6595	Significant
AMDHD1	chr12	96359367-96359375	TGAAGGCGT	5.0000	0.6804	-13.9474	Significant
Intergenic	chr12	96891476-96891484	ACGCCTTCA	5.0000	NA	-9.5736	Significant
RMST	chr12	97859049-97859057	TCGCCTTCA	5.0000	-0.2862	-5.7924	Significant
ANKS1B	chr12	100279934-100279942	ACGCCAACA	5.0000	-0.1106	-7.7711	Significant
Intergenic	chr12	102325013-102325021	TCGCCAACA	-0.5305	NA	-4.7182	Significant
Intergenic	chr12	102657260-102657268	TGTAGGCGA	2.8074	NA	-7.4699	Significant
Intergenic	chr12	102987543-102987551	TGATGGCGA	5.0000	NA	-11.4243	Significant
C12orf42	chr12	103863803-103863811	TGAAGGCGA	5.0000	-0.2261	-10.2583	Significant
STAB2	chr12	103981600-103981608	TGTTGGCGT	2.4594	0.0810	-6.6939	Significant
STAB2	chr12	104081129-104081137	ACGCCTTCA	5.0000	0.0810	-7.6831	Significant
GLT8D2	chr12	104396959-104396967	TGTTGGCGT	1.0000	0.0308	-5.9549	Significant
Intergenic	chr12	104839266-104839274	TGTTGGCGT	5.0000	NA	-6.7014	Significant
ALDH1L2	chr12	105421347-105421355	TCGCCTTCA	5.0000	0.2410	-5.1011	Significant
Intergenic	chr12	106364999-106365007	TCGCCATCA	5.0000	NA	-6.2573	Significant
Intergenic	chr12	106586145-106586153	TCGCCATCA	3.4594	NA	-22.9153	Significant
Intergenic	chr12	106621960-106621968	TGTAGGCGT	5.0000	NA	-8.7041	Significant
Intergenic	chr12	106670802-106670810	TGTTGGCGA	1.0000	NA	-10.2581	Significant
POLR3B	chr12	106817022-106817030	TCGCCATCA	3.1699	-3.0135	-8.5046	Significant
RFX4	chr12	107049873-107049881	ACGCCTTCA	5.0000	0.4036	-8.7041	Significant
LOC100287944	chr12	107049873-107049881	ACGCCTTCA	5.0000	0.1415	-8.2834	Significant
RFX4	chr12	107052108-107052116	TCGCCTTCA	3.3219	0.4036	-7.2348	Significant
LOC100287944	chr12	107052108-107052116	TCGCCTTCA	1.1155	0.1415	-4.2710	Marginal
RFX4	chr12	107104019-107104027	TGAAGGCGA	5.0000	0.4036	-7.6831	Significant
LOC100287944	chr12	107104019-107104027	TGAAGGCGA	2.1699	0.1415	-6.3300	Significant
CRY1	chr12	107462979-107462987	TCGCCAACA	1.2630	-1.3061	-10.2581	Significant
Intergenic	chr12	107568552-107568560	ACGCCAACA	3.3219	NA	-10.1750	Significant
BTBD11	chr12	107810822-107810830	TGAAGGCGA	5.0000	0.0926	-7.4727	Significant
SART3	chr12	108929163-108929171	TGTTGGCGT	2.0000	-1.7986	-7.2348	Significant
PPTC7	chr12	110972718-110972726	TGTAGGCGT	5.0000	-2.7417	-15.2513	Significant
ACAD10	chr12	112189343-112189351	TCGCCAACA	0.0000	0.2443	-7.8717	Significant
NAA25	chr12	112488470-112488478	TCGCCAACA	1.0000	-1.4198	-5.9552	Significant
Intergenic	chr12	112666711-112666719	TGTTGGCGT	5.0000	NA	-8.0378	Significant
Intergenic	chr12	112737856-112737864	ACGCCTACA	0.8074	NA	-4.2710	Marginal
Intergenic	chr12	114447772-114447780	TGAAGGCGA	5.0000	NA	-5.3906	Significant
Intergenic	chr12	116036013-116036021	TGTAGGCGT	5.0000	NA	-12.9102	Significant
Intergenic	chr12	117119072-117119080	TGATGGCGT	5.0000	NA	-9.1121	Significant
NOS1	chr12	117761340-117761348	TCGCCATCA	3.5850	-0.0210	-10.7200	Significant
KSR2	chr12	118338747-118338755	ACGCCATCA	5.0000	-0.7581	-8.2836	Significant
KSR2	chr12	118340881-118340889	TGTAGGCGA	5.0000	-0.7581	-5.5994	Significant
KSR2	chr12	118346471-118346479	ACGCCAACA	-1.0000	-0.7581	-5.5996	Significant
WDR66	chr12	122420076-122420084	ACGCCATCA	2.5850	0.1626	-6.6938	Significant
Intergenic	chr12	123388497-123388505	ACGCCATCA	-0.3626	NA	-7.2866	Significant
Intergenic	chr12	125703720-125703728	TGATGGCGA	0.3219	NA	-5.2533	Significant
TMEM132B	chr12	125817242-125817250	TGAAGGCGT	5.0000	0.0890	-11.8798	Significant
Intergenic	chr12	126314686-126314694	TGATGGCGT	5.0000	NA	-9.3124	Significant
Intergenic	chr12	126957790-126957798	ACGCCTACA	5.0000	NA	-6.8990	Significant
Intergenic	chr12	128017461-128017469	TCGCCATCA	0.0000	NA	-4.5890	Significant
TMEM132D	chr12	130357980-130357988	TCGCCTACA	5.0000	0.1304	-7.8720	Significant
Intergenic	chr12	130530438-130530446	ACGCCTACA	5.0000	NA	-4.7567	Significant
EP400	chr12	132479452-132479460	TGAAGGCGT	5.0000	-0.6779	-8.0891	Significant
EP400	chr12	132495349-132495357	TGATGGCGT	5.0000	-0.6779	-4.9165	Significant
NOC4L	chr12	132631154-132631162	TCGCCAACA	3.4594	0.0688	-7.2864	Significant
Intergenic	chr13	20164487-20164495	TGATGGCGT	3.5850	NA	-6.1002	Significant
ZMYM2	chr13	20600537-20600545	TGAAGGCGA	1.0000	-2.7554	-6.6943	Significant
GJA3	chr13	20734488-20734496	TGTAGGCGA	1.7370	-0.0578	-12.1138	Significant
Intergenic	chr13	20863577-20863585	TCGCCAACA	5.0000	NA	-8.0378	Significant

IFT88	chr13 21244552-21244560	TGAAGGCGA	1.5850	-0.9637	-10.6711	Significant
N6AMT2	chr13 21310430-21310438	ACGCCTTCA	1.9260	-0.1526	-5.2533	Significant
Intergenic	chr13 22872376-22872384	TGTAGGCGT	0.7776	NA	-7.6316	Significant
Intergenic	chr13 23691398-23691406	TCGCCATCA	5.0000	NA	-10.4803	Significant
ATP8A2	chr13 26014229-26014237	TGTTGGCGT	5.0000	-0.2723	-12.6591	Significant
ATP8A2	chr13 26210815-26210823	TCGCCTTCA	3.9069	-0.2723	-5.5994	Significant
WASF3	chr13 27225267-27225275	TCGCCAACA	5.0000	0.1715	-5.2533	Significant
Intergenic	chr13 28042997-28043005	TGTAGGCGA	1.7370	NA	-6.6938	Significant
Intergenic	chr13 28357140-28357148	TGAAGGCGA	5.0000	NA	-15.0219	Significant
Intergenic	chr13 28464771-28464779	TCGCCAACA	1.8745	NA	-9.7560	Significant
FLT1	chr13 28998564-28998572	TCGCCTTCA	0.1375	0.0947	-6.6938	Significant
Intergenic	chr13 29102566-29102574	TGAAGGCGA	5.0000	NA	-5.9014	Significant
MTUS2	chr13 29672437-29672445	TGAAGGCGT	1.8745	0.1866	-8.6980	Significant
ALOX5AP	chr13 31328826-31328834	ACGCCTTCA	-0.6521	0.1425	-7.4727	Significant
Intergenic	chr13 31558735-31558743	TGTTGGCGA	2.4594	NA	-6.8599	Significant
B3GALTL	chr13 31892984-31892992	TGATGGCGT	5.0000	-0.4605	-8.9289	Significant
STARD13	chr13 33790206-33790214	TGTTGGCGT	2.2224	-1.0713	-4.5890	Significant
RFC3	chr13 34422040-34422048	TCGCCTACA	4.1699	-0.8018	-6.8473	Significant
RFC3	chr13 34483820-34483828	TGAAGGCGT	3.8074	-0.8018	-10.2093	Significant
Intergenic	chr13 34587733-34587741	ACGCCTACA	5.0000	NA	-9.7564	Significant
NBEA	chr13 35645839-35645847	TGATGGCGA	5.0000	-1.4893	-5.1013	Significant
DCLK1	chr13 36353101-36353109	TCGCCAACA	2.8074	0.0427	-9.1342	Significant
Intergenic	chr13 37661798-37661806	TGATGGCGA	1.4150	NA	-5.0497	Significant
Intergenic	chr13 37699273-37699281	TGTTGGCGA	5.0000	NA	-11.4248	Significant
Intergenic	chr13 38201617-38201625	TGTTGGCGA	0.8745	NA	-5.2699	Significant
Intergenic	chr13 38574329-38574337	TCGCCAACA	2.1699	NA	-5.1013	Significant
Intergenic	chr13 39903129-39903137	ACGCCAACA	5.0000	NA	-12.9106	Significant
Intergenic	chr13 41051973-41051981	TGAAGGCGA	1.3219	NA	-5.9549	Significant
FOXO1	chr13 41155074-41155082	TCGCCTACA	2.4594	-3.8508	-7.6316	Significant
ELF1	chr13 41576321-41576329	ACGCCTTCA	5.0000	-2.2463	-9.8058	Significant
ELF1	chr13 41581741-41581749	ACGCCTTCA	3.3219	-2.2463	-7.4703	Significant
Intergenic	chr13 42337518-42337526	TCGCCTACA	1.4854	NA	-10.0223	Significant
Intergenic	chr13 42466562-42466570	TCGCCTTCA	1.8074	NA	-5.9549	Significant
DGKH	chr13 42641726-42641734	ACGCCAACA	3.3219	-0.2016	-18.4692	Significant
Intergenic	chr13 43132151-43132159	ACGCCAACA	5.0000	NA	-11.3976	Significant
TNFSF11	chr13 43169783-43169791	TGATGGCGT	-2.3219	-0.0072	-8.4534	Significant
ENOX1	chr13 44218493-44218501	ACGCCTACA	-0.5850	0.2649	-6.3198	Significant
Intergenic	chr13 44643015-44643023	ACGCCAACA	3.7004	NA	-5.5996	Significant
Intergenic	chr13 45642269-45642277	ACGCCAACA	5.0000	NA	-16.9831	Significant
SLC25A30	chr13 45986703-45986711	TCGCCAACA	1.8074	-1.3669	-10.0223	Significant
Intergenic	chr13 46227424-46227432	ACGCCAACA	2.3219	NA	-7.8717	Significant
Intergenic	chr13 46899935-46899943	TCGCCTTCA	5.0000	NA	-18.1227	Significant
Intergenic	chr13 47071334-47071342	TGATGGCGT	1.1375	NA	-8.4534	Significant
Intergenic	chr13 47833796-47833803	TCGCCTTCA	0.5146	NA	-9.1344	Significant
Intergenic	chr13 48355019-48355027	TCGCCATCA	2.4594	NA	-5.9549	Significant
Intergenic	chr13 48582219-48582227	TGTTGGCGT	-0.8745	NA	-7.8722	Significant
Intergenic	chr13 49368997-49369005	TGATGGCGT	0.1155	NA	-7.8722	Significant
FNDC3A	chr13 49615323-49615331	TGTTGGCGA	0.4854	-1.4592	-5.2534	Significant
Intergenic	chr13 49814362-49814370	TGATGGCGA	2.9069	NA	-6.8990	Significant
Intergenic	chr13 49880868-49880876	TGATGGCGA	5.0000	NA	-4.2573	Marginal
KPNA3	chr13 50344631-50344639	TGATGGCGT	0.8745	-2.0355	-7.2348	Significant
DLEU1	chr13 50694694-50694702	TCGCCTTCA	3.1699	-1.7950	-5.3914	Significant
DLEU1	chr13 50863567-50863575	ACGCCATCA	5.0000	-1.7950	-13.4243	Significant
Intergenic	chr13 51269748-51269756	TGAAGGCGT	3.4594	NA	-5.3317	Significant
INTS6	chr13 51961525-51961533	TGTTGGCGA	0.4854	-0.6444	-10.6711	Significant
Intergenic	chr13 53550549-53550557	TCGCCTTCA	1.1699	NA	-8.0390	Significant
OLFM4	chr13 53624668-53624676	ACGCCTTCA	5.0000	0.2059	-13.4243	Significant
Intergenic	chr13 55071300-55071308	TCGCCTTCA	3.1699	NA	-8.7043	Significant
Intergenic	chr13 55095756-55095764	ACGCCTACA	0.0000	NA	-6.3196	Significant
Intergenic	chr13 56206083-56206091	TGTTGGCGA	2.1155	NA	-6.8473	Significant
Intergenic	chr13 57849844-57849852	TCGCCATCA	5.0000	NA	-6.4695	Significant
Intergenic	chr13 58597023-58597031	TGTAGGCGT	1.4150	NA	-5.3906	Significant
Intergenic	chr13 62563979-62563986	TCGCCAACA	-0.8074	NA	-6.4691	Significant
Intergenic	chr13 65308115-65308123	TGTAGGCGT	0.1699	NA	-4.9165	Significant
Intergenic	chr13 66119787-66119795	ACGCCTTCA	5.0000	NA	-8.2834	Significant

Intergenic	chr13 66264055-66264063	ACGCCTACA	0.2895	NA	-5.5996	Significant
Intergenic	chr13 66841991-66841999	TGTAGGCGT	-0.6781	NA	-5.7926	Significant
PCDH9	chr13 67702291-67702299	TCGCCTACA	5.0000	0.1430	-9.5736	Significant
Intergenic	chr13 69149329-69149337	TGTTGGCGA	3.3219	NA	-5.7407	Significant
KLHL1	chr13 70503472-70503480	ACGCCTTCA	2.8074	0.1023	-6.6941	Significant
DACH1	chr13 72410819-72410827	TCGCCTTCA	3.4594	-0.7844	-7.6316	Significant
DACH1	chr13 72414427-72414435	ACGCCTTCA	5.0000	-0.7844	-6.0167	Significant
Intergenic	chr13 72818425-72818433	TGATGGCGT	3.5850	NA	-8.9289	Significant
Intergenic	chr13 73745086-73745094	TGAAGGCGA	5.0000	NA	-4.9165	Significant
Intergenic	chr13 73917227-73917235	TGTAGGCGT	1.3219	NA	-5.2533	Significant
Intergenic	chr13 74150440-74150448	ACGCCTACA	1.7370	NA	-7.0823	Significant
Intergenic	chr13 74257990-74257998	TGTAGGCGT	5.0000	NA	-7.8720	Significant
KLF12	chr13 74625783-74625791	TCGCCATCA	5.0000	-0.6564	-11.1907	Significant
KLF12	chr13 74653099-74653107	TCGCCTTCA	5.0000	-0.6564	-7.8722	Significant
COMMD6	chr13 76108416-76108424	TGAAGGCGT	1.2630	0.4652	-7.2348	Significant
Intergenic	chr13 76863564-76863572	TGAAGGCGA	5.0000	NA	-8.2834	Significant
Intergenic	chr13 77103128-77103136	TGATGGCGA	5.0000	NA	-17.5297	Significant
Intergenic	chr13 77906169-77906177	TCGCCAACA	3.8074	NA	-13.1223	Significant
Intergenic	chr13 79686574-79686582	TCGCCATCA	0.5146	NA	-6.3196	Significant
Intergenic	chr13 80164953-80164961	TGTTGGCGT	1.0000	NA	-7.4699	Significant
Intergenic	chr13 80253306-80253314	TGTTGGCGT	5.0000	NA	-9.1342	Significant
Intergenic	chr13 81146679-81146687	ACGCCTACA	5.0000	NA	-11.1909	Significant
Intergenic	chr13 83399923-83399931	TGTTGGCGT	1.5850	NA	-4.7182	Significant
Intergenic	chr13 84463285-84463293	TGATGGCGA	5.0000	NA	-5.6140	Significant
Intergenic	chr13 85095172-85095180	TGTAGGCGT	1.0995	NA	-8.0378	Significant
Intergenic	chr13 85098185-85098193	TCGCCTTCA	4.3219	NA	-10.6711	Significant
Intergenic	chr13 85508086-85508094	TGTAGGCGA	1.4594	NA	-7.6316	Significant
Intergenic	chr13 87012778-87012786	TCGCCTTCA	5.0000	NA	-7.2348	Significant
Intergenic	chr13 87764357-87764365	TCGCCATCA	0.7370	NA	-16.4101	Significant
Intergenic	chr13 87842317-87842325	TGAAGGCGA	3.0000	NA	-8.9289	Significant
MIR4500HG	chr13 88317101-88317109	TGATGGCGA	3.3219	0.2070	-6.8473	Significant
Intergenic	chr13 88367944-88367952	TCGCCATCA	3.3219	NA	-6.4691	Significant
Intergenic	chr13 89176356-89176364	TGTTGGCGT	5.0000	NA	-7.4701	Significant
Intergenic	chr13 89251116-89251124	TGTAGGCGA	-0.5305	NA	-7.4699	Significant
Intergenic	chr13 91670530-91670538	TGATGGCGT	5.0000	NA	-9.3124	Significant
Intergenic	chr13 91720548-91720556	TGATGGCGT	5.0000	NA	-9.3629	Significant
Intergenic	chr13 91868093-91868101	TCGCCTACA	5.0000	NA	-14.7469	Significant
Intergenic	chr13 91879909-91879917	TGAAGGCGA	0.4475	NA	-7.6831	Significant
GPC5	chr13 92316813-92316821	TGATGGCGT	5.0000	0.0943	-8.7041	Significant
GPC5	chr13 93221679-93221687	TGATGGCGT	5.0000	0.0943	-19.3366	Significant
Intergenic	chr13 95318426-95318434	TCGCCAACA	-1.3219	NA	-4.3961	Significant
ABCC4	chr13 95751133-95751141	TGTTGGCGA	1.5850	0.1318	-5.0847	Significant
ABCC4	chr13 95808213-95808221	TGTTGGCGT	0.0000	0.1318	-7.4699	Significant
CLDN10	chr13 96173043-96173051	TCGCCAACA	5.0000	0.1159	-18.4692	Significant
HS6ST3	chr13 96864500-96864508	TGAAGGCGT	1.0000	0.2864	-5.7407	Significant
HS6ST3	chr13 96881184-96881192	TGTAGGCGT	5.0000	0.2864	-8.8782	Significant
HS6ST3	chr13 97121085-97121093	ACGCCTTCA	5.0000	0.2864	-10.9476	Significant
Intergenic	chr13 98131500-98131508	ACGCCATCA	0.8931	NA	-9.3124	Significant
Intergenic	chr13 98391409-98391417	TGAAGGCGT	3.3219	NA	-9.7560	Significant
FARP1	chr13 98951720-98951728	ACGCCTTCA	5.0000	0.4119	-8.5043	Significant
PCCA	chr13 100759679-100759687	ACGCCTACA	5.0000	0.2155	-9.3124	Significant
FGF14	chr13 102828272-102828280	TGTTGGCGA	5.0000	-0.1375	-6.8473	Significant
TPP2	chr13 103271179-103271187	TGATGGCGA	3.4594	-0.3887	-12.1138	Significant
CCDC168	chr13 103410214-103410222	TGTTGGCGT	5.0000	0.1889	-5.7411	Significant
Intergenic	chr13 103720200-103720208	TGATGGCGA	2.0000	NA	-17.5305	Significant
Intergenic	chr13 103830650-103830658	TGTTGGCGA	5.0000	NA	-7.4927	Significant
Intergenic	chr13 104721805-104721813	TGATGGCGA	2.4594	NA	-5.7407	Significant
Intergenic	chr13 105391537-105391545	TCGCCATCA	5.0000	NA	-7.6316	Significant
Intergenic	chr13 106479581-106479589	ACGCCTACA	0.7776	NA	-5.3906	Significant
Intergenic	chr13 106761112-106761120	TGAAGGCGT	1.3785	NA	-10.2089	Significant
ARGLU1	chr13 107204139-107204147	ACGCCAACA	5.0000	-0.0231	-8.0378	Significant
ARGLU1	chr13 107205113-107205121	TCGCCAACA	3.3219	-0.0231	-6.3297	Significant
Intergenic	chr13 107623828-107623836	TGAAGGCGA	-0.7655	NA	-4.7186	Significant
Intergenic	chr13 108616188-108616196	ACGCCAACA	3.7004	NA	-6.1006	Significant
Intergenic	chr13 108704173-108704179	TGTAGGCGA	1.8074	NA	-11.3976	Significant

Intergenic	chr13 108724907-108724915	TGTTGGCGA	3.7004	NA	-11.1427	Significant
MYO16	chr13 109418916-109418924	TCGCCTTCA	5.0000	0.0661	-17.5104	Significant
Intergenic	chr13 109961764-109961772	ACGCCATCA	0.5146	NA	-8.2836	Significant
Intergenic	chr13 110541634-110541642	TGTTGGCGA	0.7776	NA	-10.2089	Significant
COL4A1	chr13 110946197-110946205	TCGCCAACA	5.0000	-0.1688	-8.0378	Significant
ARHGEF7	chr13 111769548-111769556	TCGCCATCA	5.0000	-2.1265	-9.5736	Significant
ARHGEF7	chr13 111803042-111803050	TGTTGGCGT	3.4594	-2.1265	-7.6316	Significant
Intergenic	chr13 112784556-112784564	TGTTGGCGT	3.1699	NA	-5.7407	Significant
Intergenic	chr13 112815573-112815581	TGAAGGCGA	-0.4475	NA	-6.4691	Significant
RASA3	chr13 114810250-114810258	ACGCCTTCA	0.1255	-2.2608	-12.1138	Significant
CDC16	chr13 115025073-115025081	TGTTGGCGT	5.0000	-1.0882	-12.1138	Significant
Intergenic	chr14 19806681-19806689	TGAAGGCGT	5.0000	NA	-4.4469	Significant
Intergenic	chr14 20222109-20222117	TGTAGGCGT	1.0000	NA	-5.3910	Significant
Intergenic	chr14 20646653-20646661	TGATGGCGA	-0.1255	NA	-5.2534	Significant
RAB2B	chr14 21936605-21936613	ACGCCTACA	0.8480	-0.7997	-7.6316	Significant
Intergenic	chr14 22170728-22170736	ACGCCATCA	1.1375	NA	-6.8473	Significant
Intergenic	chr14 23000674-23000682	TGATGGCGA	5.0000	NA	-8.4534	Significant
Intergenic	chr14 24369906-24369914	ACGCCTACA	5.0000	NA	-5.1303	Significant
DHRS4L2	chr14 24450264-24450272	ACGCCATCA	1.4150	0.7773	-5.3906	Significant
Intergenic	chr14 25005743-25005751	TCGCCAACA	-0.3219	NA	-5.5996	Significant
Intergenic	chr14 28382852-28382860	TCGCCATCA	5.0000	NA	-6.9960	Significant
Intergenic	chr14 29287977-29287985	TCGCCTACA	1.5850	NA	-6.6938	Significant
Intergenic	chr14 29518479-29518487	TCGCCATCA	-1.2630	NA	-8.4534	Significant
Intergenic	chr14 29709202-29709210	ACGCCTTCA	-1.6845	NA	-8.8782	Significant
PRKD1	chr14 30211574-30211582	TCGCCTACA	0.3410	0.0896	-5.1013	Significant
Intergenic	chr14 30490593-30490601	TGAAGGCGA	2.5850	NA	-6.8990	Significant
SCFD1	chr14 31145131-31145139	ACGCCAACA	-0.3479	-0.8860	-5.7407	Significant
Intergenic	chr14 31274005-31274013	TGTTGGCGT	-0.6781	NA	-5.2533	Significant
STRN3	chr14 31380655-31380663	ACGCCAACA	2.8074	-0.8979	-7.8720	Significant
STRN3	chr14 31479171-31479179	TGATGGCGT	2.5025	-0.8979	-7.8722	Significant
STRN3	chr14 31493055-31493063	ACGCCATCA	5.0000	-0.8979	-12.4056	Significant
HEATR5A	chr14 31794270-31794278	TGTAGGCGT	0.4475	-0.9274	-6.8473	Significant
HEATR5A	chr14 31882665-31882673	TGATGGCGA	1.1155	-0.9274	-6.3196	Significant
NUBPL	chr14 32230666-32230674	TGAAGGCGT	5.0000	-0.2285	-9.5736	Significant
NUBPL	chr14 32309163-32309171	TGTTGGCGA	5.0000	-0.2285	-5.0497	Significant
ARHGAP5	chr14 32608691-32608699	TGATGGCGT	-0.1520	-2.3413	-9.7560	Significant
Intergenic	chr14 33397904-33397912	TGTTGGCGA	5.0000	NA	-7.8720	Significant
NPAS3	chr14 33792982-33792990	ACGCCATCA	1.2630	0.0877	-8.2834	Significant
NPAS3	chr14 34202819-34202827	TGTTGGCGT	-0.1520	0.0877	-9.7560	Significant
Intergenic	chr14 34796378-34796386	TGATGGCGA	3.0000	NA	-6.1002	Significant
Intergenic	chr14 35895323-35895331	TCGCCATCA	3.7004	NA	-9.1249	Significant
RALGAPA1	chr14 36090887-36090895	ACGCCATCA	2.3219	-1.8818	-8.8782	Significant
Intergenic	chr14 36427017-36427025	TGTTGGCGT	0.1699	NA	-12.1138	Significant
Intergenic	chr14 36571173-36571181	TCGCCTACA	5.0000	NA	-5.7407	Significant
Intergenic	chr14 36763554-36763562	TGATGGCGA	2.8074	NA	-7.2348	Significant
MBIP	chr14 36789090-36789098	ACGCCAACA	5.0000	-1.5608	-5.1013	Significant
Intergenic	chr14 37068288-37068296	TGTTGGCGT	2.1155	NA	-8.8782	Significant
Intergenic	chr14 37071404-37071412	TCGCCATCA	0.8480	NA	-9.3124	Significant
SLC25A21	chr14 37436444-37436452	TCGCCTTCA	1.2224	0.1269	-7.6316	Significant
MIPOL1	chr14 37844141-37844149	TGATGGCGA	2.3219	-0.6059	-10.5050	Significant
Intergenic	chr14 38218541-38218549	TGTAGGCGT	5.0000	NA	-8.4465	Significant
Intergenic	chr14 38320563-38320571	TGATGGCGA	4.0875	NA	-8.2836	Significant
Intergenic	chr14 39315128-39315136	ACGCCTTCA	5.0000	NA	-9.5736	Significant
Intergenic	chr14 39466669-39466677	TGTAGGCGA	2.3219	NA	-6.5209	Significant
Intergenic	chr14 39577984-39577992	ACGCCTTCA	1.2630	NA	-9.1344	Significant
Intergenic	chr14 40123253-40123261	TGATGGCGA	-0.2895	NA	-7.4699	Significant
Intergenic	chr14 40125052-40125060	TGTAGGCGA	5.0000	NA	-4.9166	Significant
Intergenic	chr14 41076735-41076743	TGAAGGCGT	-0.2410	NA	-8.7041	Significant
Intergenic	chr14 43091425-43091433	ACGCCTTCA	3.1699	NA	-10.6711	Significant
Intergenic	chr14 43693495-43693503	TCGCCTTCA	2.1155	NA	-6.5209	Significant
Intergenic	chr14 44031165-44031173	ACGCCATCA	2.4594	NA	-11.9103	Significant
Intergenic	chr14 46218990-46218998	TGTAGGCGA	-0.7004	NA	-4.7695	Significant
Intergenic	chr14 46248170-46248178	TGATGGCGT	5.0000	NA	-7.6316	Significant
MDGA2	chr14 47708311-47708319	TCGCCATCA	-0.8074	-0.6270	-11.1431	Significant
Intergenic	chr14 49334686-49334694	ACGCCATCA	1.4594	NA	-7.4699	Significant

Intergenic	chr14 49682832-49682840	ACGCCTACA	0.0000	NA	-6.1002	Significant
Intergenic	chr14 49915580-49915588	ACGCCATCA	0.3219	NA	-6.8473	Significant
CDKL1	chr14 50838096-50838104	TCGCCAACA	-0.8074	-0.6441	-4.4469	Significant
ATL1	chr14 51034332-51034340	TCGCCAACA	5.0000	-1.0699	-5.4213	Significant
TRIM9	chr14 51506295-51506303	TGATGGCGA	0.2630	0.2150	-7.4701	Significant
NID2	chr14 52503374-52503382	TCGCCAACA	1.4854	0.1210	-6.3196	Significant
Intergenic	chr14 53288662-53288670	TGTTGGCGA	1.3219	NA	-9.3629	Significant
Intergenic	chr14 53837001-53837009	TGAAGGCGA	0.0000	NA	-7.6316	Significant
Intergenic	chr14 53986033-53986041	TGAAGGCGA	0.0000	NA	-6.8473	Significant
Intergenic	chr14 54061961-54061969	ACGCCTACA	5.0000	NA	-5.4421	Significant
Intergenic	chr14 54486324-54486332	TGTTGGCGT	1.0000	NA	-5.2533	Significant
Intergenic	chr14 57535640-57535648	TGAAGGCGT	3.3219	NA	-11.4243	Significant
Intergenic	chr14 57645214-57645222	TGTTGGCGA	2.3219	NA	-9.1342	Significant
SLC35F4	chr14 58088789-58088797	TCGCCATCA	-0.1926	0.2262	-7.2356	Significant
SLC35F4	chr14 58102578-58102586	ACGCCAACA	-0.8667	0.2262	-4.9165	Significant
Intergenic	chr14 60683107-60683115	TCGCCTTCA	2.4594	NA	-5.9552	Significant
C14orf39	chr14 60951503-60951511	ACGCCAACA	1.2224	0.0676	-8.4534	Significant
Intergenic	chr14 61011562-61011570	ACGCCTACA	-0.3626	NA	-4.5890	Significant
FLJ22447	chr14 62091453-62091461	TCGCCATCA	2.3219	0.0915	-10.6711	Significant
Intergenic	chr14 62981863-62981871	ACGCCAACA	-0.6781	NA	-6.8477	Significant
KCNH5	chr14 63424944-63424952	ACGCCAACA	5.0000	-0.2579	-5.4423	Significant
ESR2	chr14 64725638-64725646	ACGCCTTCA	5.0000	0.1499	-6.1002	Significant
ESR2	chr14 64795748-64795756	ACGCCTACA	5.0000	0.1499	-9.3124	Significant
Intergenic	chr14 65672944-65672952	ACGCCATCA	5.0000	NA	-6.5209	Significant
ATP6V1D	chr14 67817388-67817397	TCGCCATCA	-0.4150	-0.0894	-5.5996	Significant
ZFYVE26	chr14 68266173-68266181	TGAAGGCGT	2.0000	-3.0105	-5.9550	Significant
ERH	chr14 69854139-69854147	TGATGGCGT	3.3219	0.0608	-10.0223	Significant
Intergenic	chr14 70766170-70766178	TGAAGGCGA	5.0000	NA	-7.8720	Significant
RGS6	chr14 72591317-72591325	TGTTGGCGA	-0.7935	0.0876	-6.4691	Significant
RBM25	chr14 73569100-73569108	TGTTGGCGA	-2.1375	-0.6961	-6.4691	Significant
Intergenic	chr14 73590499-73590507	TGAAGGCGA	2.3219	NA	-6.5209	Significant
PTGR2	chr14 74337886-74337894	ACGCCTTCA	1.0000	0.0180	-9.1342	Significant
FLVCR2	chr14 76050649-76050657	TGAAGGCGA	2.1699	-0.2041	-8.4538	Significant
Intergenic	chr14 77199561-77199569	ACGCCAACA	0.0000	NA	-7.2348	Significant
Promoter_ALKBH1	chr14 78174869-78174877	TGTAGGCGT	2.8074	-1.8071	-8.4534	Significant
NRXN3	chr14 79169173-79169181	TCGCCATCA	1.4594	-1.0540	-12.3714	Significant
Intergenic	chr14 80388633-80388640	TGAAGGCGT	5.0000	NA	-5.0497	Significant
Intergenic	chr14 82856276-82856284	TGAAGGCGT	3.7004	NA	-9.1342	Significant
Intergenic	chr14 83881179-83881187	TGTTGGCGT	1.1375	NA	-8.0893	Significant
Intergenic	chr14 84537881-84537889	TGTTGGCGT	0.6781	NA	-5.9549	Significant
Intergenic	chr14 84567571-84567579	TGATGGCGT	2.0000	NA	-8.8782	Significant
Intergenic	chr14 86864365-86864373	ACGCCTACA	1.0000	NA	-6.9960	Significant
Intergenic	chr14 89509656-89509664	TCGCCTTCA	0.3626	NA	-8.0378	Significant
EFCAB11	chr14 90288681-90288689	TGTAGGCGT	5.0000	-0.0935	-8.0891	Significant
Intergenic	chr14 90771324-90771332	TGTAGGCGT	2.9069	NA	-7.0772	Significant
Intergenic	chr14 91544870-91544878	ACGCCTTCA	0.4150	NA	-7.2348	Significant
Intergenic	chr14 91734514-91734522	TCGCCAACA	5.0000	NA	-4.3278	Significant
RIN3	chr14 93016908-93016916	ACGCCATCA	0.8074	-0.9967	-8.8782	Significant
UNC79	chr14 94008839-94008847	ACGCCTACA	2.5850	0.0645	-9.5737	Significant
IFI27L1	chr14 94548836-94548844	ACGCCATCA	-1.6571	0.2242	-9.1249	Significant
Intergenic	chr14 96491727-96491735	ACGCCTTCA	3.1699	NA	-8.4534	Significant
BDKRB1	chr14 96722617-96722625	ACGCCTTCA	-0.7590	-1.3285	-6.1002	Significant
ATG2B	chr14 96795287-96795295	TGTAGGCGA	5.0000	-1.4680	-7.2352	Significant
Intergenic	chr14 97180051-97180059	TGTTGGCGT	1.8074	NA	-11.6709	Significant
VRK1	chr14 97343732-97343740	TGAAGGCGA	2.3219	-1.0580	-21.9155	Significant
Intergenic	chr14 97944247-97944254	ACGCCTTCA	0.1926	NA	-8.4534	Significant
Intergenic	chr14 97993554-97993562	TGAAGGCGA	3.1699	NA	-4.9166	Significant
Intergenic	chr14 98962446-98962454	TCGCCTTCA	0.0000	NA	-9.1342	Significant
Promoter_SNORD114-14	chr14 101437484-101437492	TGATGGCGT	2.9069	-0.0989	-7.0772	Significant
Intergenic	chr14 101585198-101585206	TCGCCTACA	0.8480	NA	-5.3906	Significant
WDR20	chr14 102649542-102649550	TGAAGGCGA	1.4594	-0.9321	-9.7564	Significant
PPP1R13B	chr14 104245039-104245046	ACGCCATCA	2.1699	-2.8317	-13.1671	Significant
Intergenic	chr14 104934899-104934905	TGATGGCGT	3.5850	NA	-11.4244	Significant
Intergenic	chr14 106721875-106721883	TCGCCTTCA	2.0000	NA	-6.8473	Significant
Intergenic	chr14 106802082-106802090	ACGCCTACA	5.0000	NA	-17.2828	Significant

Intergenic	chr14 107045447-107045455	TGATGGCGA	1.0000	NA	-6.4699	Significant
Intergenic	chr15 20267936-20267944	ACGCCATCA	5.0000	NA	-8.2834	Significant
Intergenic	chr15 20457603-20457611	TGATGGCGT	3.5850	NA	-8.4534	Significant
SNRPN	chr15 25153286-25153294	TGATGGCGT	5.0000	0.0732	-6.8990	Significant
Intergenic	chr15 29936135-29936143	TGTAGGCGT	1.7370	NA	-7.0774	Significant
TJP1	chr15 30199762-30199770	TCGCCAACA	5.0000	-1.8348	-8.0378	Significant
TRPM1	chr15 31325022-31325030	TGTAGGCGA	-1.0000	0.1406	-7.4699	Significant
Intergenic	chr15 32020189-32020197	ACGCCAACA	-0.8625	NA	-7.6316	Significant
AVEN	chr15 34169752-34169760	ACGCCTACA	0.0000	-0.3840	-6.1002	Significant
Intergenic	chr15 36278666-36278674	TGTTGGCGT	1.5850	NA	-8.5043	Significant
Intergenic	chr15 37513317-37513325	TCGCCAACA	3.7004	NA	-5.0501	Significant
Intergenic	chr15 39188220-39188228	TGTTGGCGA	1.3219	NA	-6.8990	Significant
Intergenic	chr15 42712724-42712732	ACGCCATCA	1.4854	NA	-10.2585	Significant
Intergenic	chr15 43610815-43610823	TGTAGGCGA	5.0000	NA	-5.3906	Significant
Intergenic	chr15 43612635-43612643	TGATGGCGA	0.5305	NA	-5.9550	Significant
CASC4	chr15 44621962-44621970	TGATGGCGT	-0.7655	0.1209	-6.6938	Significant
Intergenic	chr15 45816450-45816458	TGATGGCGT	5.0000	NA	-6.1522	Significant
Intergenic	chr15 46182260-46182268	TGATGGCGA	5.0000	NA	-5.6140	Significant
Intergenic	chr15 46217066-46217074	TCGCCTACA	2.0000	NA	-4.3961	Significant
Intergenic	chr15 47446512-47446520	TCGCCATCA	2.8074	NA	-8.8782	Significant
SEMA6D	chr15 47747138-47747146	TGTAGGCGT	1.4150	0.0082	-8.9289	Significant
SEMA6D	chr15 47748185-47748193	TGATGGCGA	-0.7370	0.0082	-4.3961	Significant
SEMA6D	chr15 47835659-47835667	TCGCCTTCA	2.1699	0.0082	-4.3961	Significant
SEMA6D	chr15 47986630-47986638	TCGCCTTCA	1.3785	0.0082	-8.4534	Significant
Intergenic	chr15 48098152-48098160	ACGCCATCA	5.0000	NA	-4.5890	Significant
Intergenic	chr15 48309421-48309429	TGTTGGCGA	-1.1155	NA	-7.6316	Significant
AP4E1	chr15 51260218-51260226	TGTTGGCGA	2.5025	-2.2413	-8.2805	Significant
ONECUT1	chr15 53051243-53051251	TGTAGGCGT	1.2630	0.1195	-7.0772	Significant
TEX9	chr15 56658647-56658655	ACGCCTTCA	4.0000	0.1818	-9.7284	Significant
MYZAP	chr15 57963372-57963380	TCGCCTTCA	1.5850	-1.8284	-9.5737	Significant
GCOM1	chr15 57963372-57963380	TCGCCTTCA	3.5850	-1.3241	-8.2834	Significant
Intergenic	chr15 58024760-58024768	TGAAGGCGT	1.0000	NA	-7.0823	Significant
LIPC	chr15 58724898-58724906	TGATGGCGA	2.0000	0.0996	-8.4534	Significant
SLTM	chr15 59180665-59180673	TGTTGGCGA	2.4594	-2.2962	-6.4691	Significant
Intergenic	chr15 60181823-60181831	TGATGGCGT	0.7776	NA	-5.9550	Significant
RORA	chr15 60990182-60990190	ACGCCTACA	5.0000	-2.3131	-13.1673	Significant
Intergenic	chr15 62046850-62046858	ACGCCTTCA	4.0875	NA	-13.1223	Significant
Intergenic	chr15 62836531-62836539	TCGCCAACA	3.1699	NA	-6.1002	Significant
Intergenic	chr15 63199902-63199910	ACGCCTACA	5.0000	NA	-6.8599	Significant
USP3	chr15 63882958-63882966	TGAAGGCGA	2.4594	-2.1800	-6.4691	Significant
HERC1	chr15 63940543-63940551	TGTAGGCGT	5.0000	-1.2627	-7.0772	Significant
SNX1	chr15 64435021-64435029	TCGCCTACA	2.3219	0.1890	-8.0891	Significant
KIAA0101	chr15 64664206-64664214	ACGCCTTCA	5.0000	0.1280	-8.7041	Significant
TRIP4	chr15 64719776-64719784	TGTTGGCGA	-0.1699	-2.5679	-5.2533	Significant
Intergenic	chr15 64774873-64774881	TGTTGGCGT	1.4594	NA	-12.1142	Significant
DPP8	chr15 65772696-65772704	TGTAGGCGA	3.0000	-0.5547	-5.4425	Significant
SLC24A1	chr15 65942636-65942644	ACGCCAACA	1.0000	-0.3869	-6.4695	Significant
Intergenic	chr15 66149805-66149813	TGTAGGCGT	0.5850	NA	-11.1427	Significant
SMAD3	chr15 67440216-67440224	TGTTGGCGT	2.8074	-2.4793	-4.6100	Significant
IQCH	chr15 67608037-67608045	TCGCCTTCA	5.0000	0.0290	-7.4724	Significant
MAP2K5	chr15 67935125-67935133	TGTAGGCGA	5.0000	-1.7268	-6.6938	Significant
PIAS1	chr15 68353293-68353301	TGTAGGCGT	1.1375	-2.1845	-6.6938	Significant
ANP32A	chr15 69074575-69074583	ACGCCTACA	2.4594	-0.1581	-7.0825	Significant
NOX5	chr15 69225412-69225420	TGTTGGCGA	5.0000	0.2247	-7.4699	Significant
NOX5	chr15 69278172-69278180	TGATGGCGA	5.0000	0.2247	-11.4243	Significant
KIF23	chr15 69719666-69719674	TGTAGGCGA	3.3219	-3.0887	-7.6833	Significant
UACA	chr15 71018154-71018162	TGAAGGCGA	5.0000	-0.7413	-7.6316	Significant
Intergenic	chr15 71117745-71117753	TGAAGGCGA	-0.7004	NA	-4.2711	Marginal
Intergenic	chr15 72618968-72618976	TGTTGGCGA	-1.9594	NA	-6.8473	Significant
NEO1	chr15 73575375-73575383	TGTTGGCGT	0.6521	-0.0689	-7.8720	Significant
Intergenic	chr15 73830174-73830182	TCGCCTTCA	2.0000	NA	-7.8720	Significant
Intergenic	chr15 74208222-74208230	ACGCCATCA	4.0000	NA	-14.1685	Significant
ARID3B	chr15 74854820-74854828	ACGCCATCA	3.4594	-3.4006	-11.1427	Significant
Intergenic	chr15 77878723-77878731	ACGCCATCA	0.1520	NA	-8.4534	Significant
CHRNA5	chr15 78859995-78860003	TCGCCAACA	5.0000	-0.7812	-9.1124	Significant

ARNT2	chr15 80839560-80839568	TGATGGCGT	3.3219	-0.0249	-4.9165	Significant
Intergenic	chr15 81250537-81250545	TCGCCTACA	1.5850	NA	-4.9165	Significant
SH3GL3	chr15 84268594-84268602	ACGCCAACA	1.2224	-0.4497	-4.2931	Marginal
ADAMTSL3	chr15 84536906-84536914	TCGCCTACA	5.0000	0.0751	-10.9247	Significant
Intergenic	chr15 84785617-84785625	TGTTGGCGA	5.0000	NA	-10.4609	Significant
AKAP13	chr15 86170130-86170138	TCGCCAACA	1.8745	-4.2603	-6.4695	Significant
AKAP13	chr15 86205732-86205740	ACGCCATCA	3.4594	-4.2603	-10.2089	Significant
NTRK3	chr15 88580909-88580917	TGTAGGCGA	1.7004	0.0072	-4.5891	Significant
Intergenic	chr15 89217901-89217909	TGTTGGCGA	5.0000	NA	-7.2866	Significant
Intergenic	chr15 89614183-89614191	TGAAGGCGT	0.6781	NA	-6.8484	Significant
Intergenic	chr15 90137691-90137699	TGAAGGCGA	5.0000	NA	-6.6938	Significant
SLCO3A1	chr15 92495263-92495271	TCGCCAACA	5.0000	-1.8739	-6.1002	Significant
Intergenic	chr15 92857505-92857512	ACGCCAACA	1.0000	NA	-10.5050	Significant
Intergenic	chr15 94639499-94639507	TGTTGGCGA	3.3219	NA	-5.3906	Significant
Intergenic	chr15 96388749-96388757	TGTTGGCGA	1.0000	NA	-8.0378	Significant
Intergenic	chr15 97147220-97147228	TGAAGGCGT	5.0000	NA	-9.3124	Significant
Intergenic	chr15 97194623-97194631	TGAAGGCGA	5.0000	NA	-18.1569	Significant
Intergenic	chr15 97437687-97437695	TGTAGGCGA	-0.3219	NA	-8.5048	Significant
Intergenic	chr15 97722182-97722190	TCGCCTTCA	1.7370	NA	-6.6939	Significant
SYNM	chr15 99673295-99673303	TGTTGGCGA	1.4594	0.1423	-4.9166	Significant
MEF2A	chr15 100215382-100215390	TGAAGGCGA	5.0000	-3.8524	-6.8473	Significant
LRRK1	chr15 101514756-101514764	TGTAGGCGT	-0.3219	-1.5634	-5.3906	Significant
CHSY1	chr15 101726094-101726102	ACGCCTACA	3.9069	-3.6885	-19.9871	Significant
TM2D3	chr15 102189153-102189161	TCGCCTACA	2.3219	-0.6708	-9.5736	Significant
Promoter_OR4F15	chr15 102357572-102357580	ACGCCTTCA	5.0000	0.0452	-9.7560	Significant
ERVK13-1	chr16 2711320-2711328	TCGCCATCA	1.7370	-0.4368	-9.5737	Significant
CREBBP	chr16 3918605-3918613	TCGCCTTCA	3.3219	-3.1532	-7.8720	Significant
C16orf71	chr16 4792364-4792372	TCGCCAACA	0.0000	0.0867	-8.2834	Significant
GLYR1	chr16 4893303-4893311	ACGCCAACA	0.7370	0.1300	-9.3124	Significant
UBN1	chr16 4929162-4929170	TCGCCAACA	2.4594	NA	-9.3128	Significant
RBFOX1	chr16 7472286-7472294	TGTAGGCGT	5.0000	0.2101	-9.1342	Significant
PMM2	chr16 8910615-8910623	TGTTGGCGA	-1.0875	-1.7280	-7.8724	Significant
GRIN2A	chr16 9852253-9852261	ACGCCAACA	5.0000	0.0849	-10.0223	Significant
Intergenic	chr16 10362995-10363003	ACGCCAACA	5.0000	NA	-14.4802	Significant
SHISA9	chr16 13246817-13246825	TGTAGGCGA	2.3219	-0.0050	-8.2838	Significant
Intergenic	chr16 13542178-13542186	ACGCCTTCA	-1.1520	NA	-13.1223	Significant
PDXDC1	chr16 15120445-15120453	TGAAGGCGT	5.0000	-0.7048	-8.4534	Significant
MYH11	chr16 15869623-15869631	TGAAGGCGT	5.0000	0.0005	-11.6709	Significant
Intergenic	chr16 17127250-17127258	TGTAGGCGT	0.5146	NA	-6.1520	Significant
XYLT1	chr16 17510916-17510924	TGAAGGCGT	2.9069	-0.7673	-7.2348	Significant
XYLT1	chr16 17512807-17512815	TGTAGGCGA	3.4594	-0.7673	-10.4804	Significant
Intergenic	chr16 19293491-19293499	ACGCCTACA	5.0000	NA	-7.2866	Significant
LOC81691	chr16 20834626-20834634	TGTAGGCGT	-0.2630	-0.1288	-6.3196	Significant
DNAH3	chr16 20951657-20951665	TGTAGGCGT	2.1155	-0.0290	-11.9103	Significant
Intergenic	chr16 21572176-21572184	TGTAGGCGT	0.1255	NA	-4.7182	Significant
METTL9	chr16 21666687-21666695	TGAAGGCGA	0.1699	-0.1500	-7.2866	Significant
USP31	chr16 23111353-23111361	TGTTGGCGA	5.0000	-2.0896	-8.5043	Significant
Intergenic	chr16 23819918-23819926	ACGCCAACA	1.5850	NA	-17.2830	Significant
PRKCB	chr16 23855489-23855497	ACGCCTTCA	5.0000	-0.1663	-6.6938	Significant
Intergenic	chr16 24446314-24446322	ACGCCTACA	-0.2224	NA	-8.8782	Significant
Intergenic	chr16 24616954-24616962	TGTTGGCGA	2.5850	NA	-9.1342	Significant
LCMT1	chr16 25163610-25163618	ACGCCTACA	0.5850	-0.3023	-6.1002	Significant
Intergenic	chr16 25384770-25384778	TCGCCTACA	2.3219	NA	-5.3906	Significant
Intergenic	chr16 25566133-25566141	ACGCCTTCA	0.6374	NA	-5.5994	Significant
Intergenic	chr16 26209314-26209322	TCGCCTTCA	5.0000	NA	-7.0823	Significant
Intergenic	chr16 26883845-26883853	TGTAGGCGT	-0.2895	NA	-12.1603	Significant
GSG1L	chr16 27961434-27961442	ACGCCATCA	-0.8931	0.3588	-6.3198	Significant
Intergenic	chr16 31667226-31667234	TCGCCAACA	3.0000	NA	-4.2576	Marginal
Intergenic	chr16 32140750-32140758	TGTAGGCGT	5.0000	NA	-12.9104	Significant
Intergenic	chr16 32574667-32574675	TGTAGGCGT	2.3219	NA	-5.0497	Significant
Intergenic	chr16 33083988-33083996	TGTAGGCGT	1.3219	NA	-6.3297	Significant
Intergenic	chr16 33401126-33401134	ACGCCATCA	4.0000	NA	-13.5543	Significant
Intergenic	chr16 35222973-35222981	ACGCCTACA	0.0000	NA	-5.9550	Significant
PHKB	chr16 47676937-47676945	ACGCCAACA	5.0000	0.1491	-6.1002	Significant
Intergenic	chr16 49442720-49442728	TGTTGGCGT	-0.3219	NA	-5.7926	Significant

Intergenic	chr16 52016855-52016863	TCGCCTTCA	5.0000	NA	-4.9352	Significant
Intergenic	chr16 52447514-52447522	TGATGGCGA	5.0000	NA	-9.7560	Significant
TOX3	chr16 52517064-52517072	ACGCCTTCA	5.0000	-1.5406	-11.1909	Significant
Intergenic	chr16 52673796-52673804	TCGCCATCA	1.1155	NA	-5.9549	Significant
Intergenic	chr16 52998062-52998070	TCGCCTTCA	-0.4854	NA	-7.2348	Significant
FTO	chr16 53804709-53804717	TGAAGGCGA	5.0000	-0.5281	-8.9291	Significant
FTO	chr16 54003317-54003324	ACGCCTTCA	2.5850	-0.5281	-5.1013	Significant
NUP93	chr16 56788901-56788909	TGTTGGCGA	3.7004	-0.6144	-6.1522	Significant
Intergenic	chr16 56960157-56960165	ACGCCTACA	3.0000	NA	-16.7038	Significant
Intergenic	chr16 58456944-58456952	ACGCCTTCA	5.0000	NA	-6.3297	Significant
Intergenic	chr16 59555141-59555149	TGAAGGCGA	-0.3785	NA	-5.0497	Significant
Intergenic	chr16 60402399-60402407	TGAAGGCGA	1.8074	NA	-5.3906	Significant
CDH8	chr16 61721486-61721494	ACGCCTTCA	5.0000	0.0254	-12.6138	Significant
Intergenic	chr16 62744049-62744057	ACGCCTACA	-1.0000	NA	-11.6240	Significant
Intergenic	chr16 64369249-64369257	TCGCCTTCA	1.8074	NA	-6.3198	Significant
CDH11	chr16 64990207-64990215	TGTTGGCGA	1.5850	-3.5818	-5.3914	Significant
Intergenic	chr16 65348031-65348039	TGTTGGCGA	5.0000	NA	-6.3196	Significant
CMTM4	chr16 66661632-66661640	TGAAGGCGT	0.9069	-0.1122	-12.6134	Significant
Intergenic	chr16 66742661-66742669	TGTTGGCGA	0.9069	NA	-12.6134	Significant
CTCF	chr16 67625447-67625455	TGATGGCGT	-1.2016	-2.5241	-7.8720	Significant
ZFP90	chr16 68577026-68577034	TGAAGGCGA	-0.8231	-2.3196	-6.1002	Significant
WWP2	chr16 69873704-69873712	ACGCCATCA	5.0000	-1.5173	-12.6593	Significant
PDXDC2P	chr16 70048117-70048125	ACGCCTTCA	1.5850	-0.9392	-5.5994	Significant
HYDIN	chr16 71063874-71063882	TGATGGCGA	5.0000	0.1232	-5.1013	Significant
HYDIN	chr16 71206968-71206976	ACGCCATCA	5.0000	0.1232	-11.1427	Significant
Intergenic	chr16 72268908-72268916	TGATGGCGT	5.0000	NA	-8.7041	Significant
Intergenic	chr16 72484335-72484343	TGATGGCGT	5.0000	NA	-5.0501	Significant
Intergenic	chr16 73559085-73559093	TGTTGGCGA	3.0000	NA	-4.9350	Significant
Intergenic	chr16 74059291-74059299	TCGCCATCA	2.7004	NA	-6.1522	Significant
Intergenic	chr16 74147620-74147628	TGAAGGCGA	5.0000	NA	-5.9549	Significant
Intergenic	chr16 74234958-74234966	TCGCCTTCA	5.0000	NA	-7.2348	Significant
Intergenic	chr16 74243424-74243432	TCGCCAACA	2.7004	NA	-10.9249	Significant
Intergenic	chr16 75963923-75963931	TCGCCTTCA	1.4150	NA	-8.0378	Significant
CNTNAP4	chr16 76427827-76427835	TCGCCAACA	0.6781	0.0772	-5.3910	Significant
Intergenic	chr16 76972822-76972830	ACGCCAACA	5.0000	NA	-11.6709	Significant
Intergenic	chr16 77543138-77543146	ACGCCAACA	0.1926	NA	-6.6938	Significant
Intergenic	chr16 78123496-78123504	TGTAGGCGT	5.0000	NA	-11.4244	Significant
WWOX	chr16 78150514-78150522	TGATGGCGT	5.0000	0.0373	-11.4243	Significant
WWOX	chr16 78582692-78582700	ACGCCTTCA	5.0000	0.0373	-11.1907	Significant
Intergenic	chr16 79702708-79702716	TGTAGGCGA	2.9069	NA	-12.1138	Significant
Intergenic	chr16 80101070-80101078	TGATGGCGT	0.9069	NA	-13.6409	Significant
Intergenic	chr16 81310761-81310769	ACGCCATCA	2.8074	NA	-6.4695	Significant
Intergenic	chr16 81471821-81471829	ACGCCATCA	5.0000	NA	-9.5736	Significant
PLCG2	chr16 81837806-81837814	TCGCCAACA	5.0000	-0.0873	-9.5736	Significant
Intergenic	chr16 82523913-82523921	TGATGGCGA	0.8931	NA	-7.0772	Significant
CDH13	chr16 83179174-83179182	ACGCCATCA	0.8480	0.0823	-9.5736	Significant
CDH13	chr16 83366848-83366856	TGTTGGCGT	2.1155	0.0823	-8.2834	Significant
USP10	chr16 84793212-84793220	TGTTGGCGA	-0.1255	-0.4987	-5.2533	Significant
Intergenic	chr16 86597638-86597646	ACGCCAACA	0.5850	NA	-9.3124	Significant
BANP	chr16 88068184-88068192	ACGCCAACA	0.8745	-1.8247	-16.1337	Significant
SPIRE2	chr16 89896796-89896804	TGAAGGCGA	5.0000	-1.1470	-12.9102	Significant
Intergenic	chr16 90252650-90252658	TCGCCAACA	1.3785	NA	-4.5893	Significant
NXN	chr17 867859-867867	ACGCCTACA	5.0000	0.0118	-5.4423	Significant
YWHAE	chr17 1277348-1277356	ACGCCTACA	1.7370	0.0947	-7.4699	Significant
RPA1	chr17 1747878-1747886	TGTTGGCGA	3.0000	-0.2289	-11.1907	Significant
SPATA22	chr17 3361622-3361630	TGTTGGCGA	-0.7105	0.2188	-7.8720	Significant
ANKFY1	chr17 4085054-4085062	ACGCCTTCA	0.6781	0.0516	-8.4444	Significant
Intergenic	chr17 6623633-6623641	TGTTGGCGA	1.8074	NA	-4.3961	Significant
Intergenic	chr17 7961750-7961758	TCGCCTTCA	3.7004	NA	-6.4695	Significant
CCDC42	chr17 8636593-8636601	TGTAGGCGA	0.2410	-0.0349	-7.0772	Significant
USP43	chr17 9598088-9598096	ACGCCATCA	-0.6781	-2.3983	-7.2864	Significant
MYH4	chr17 10371238-10371246	TGATGGCGA	3.8074	0.0767	-6.1520	Significant
Intergenic	chr17 10640954-10640962	TGTAGGCGT	0.1255	NA	-6.8990	Significant
SHISA6	chr17 11423078-11423086	ACGCCAACA	0.8074	0.1184	-4.7693	Significant
DNAH9	chr17 11559531-11559539	ACGCCAACA	5.0000	0.0862	-12.6591	Significant

DNAH9	chr17 11688416-11688424	TGATGGCGT	1.6781	0.0862	-4.7695	Significant
DNAH9	chr17 11718722-11718730	ACGCCAACA	3.1699	0.0862	-21.4865	Significant
Intergenic	chr17 12104434-12104442	TGATGGCGT	-0.1926	NA	-6.1522	Significant
Intergenic	chr17 13077296-13077304	TCGCCTTCA	3.0000	NA	-5.1011	Significant
Intergenic	chr17 15297101-15297109	TCGCCTTCA	0.0000	NA	-6.5211	Significant
NCOR1	chr17 15989708-15989716	ACGCCTTCA	-0.9329	-0.5837	-7.0774	Significant
NCOR1	chr17 16029006-16029014	ACGCCATCA	2.1699	-0.5837	-5.9549	Significant
Intergenic	chr17 18496588-18496596	ACGCCATCA	5.0000	NA	-6.8473	Significant
ZNF286B	chr17 18583586-18583594	ACGCCTTCA	5.0000	-1.5024	-8.0346	Significant
Intergenic	chr17 20713622-20713630	TGTTGGCGA	-0.5850	NA	-6.4699	Significant
Intergenic	chr17 21335021-21335029	TGTTGGCGA	5.0000	NA	-7.4699	Significant
Intergenic	chr17 21682751-21682759	TGATGGCGA	3.1699	NA	-8.4534	Significant
Intergenic	chr17 21995551-21995559	ACGCCTTCA	5.0000	NA	-8.0382	Significant
Intergenic	chr17 22027890-22027898	TGTAGGCGT	5.0000	NA	-7.6339	Significant
Intergenic	chr17 22157300-22157308	ACGCCTACA	5.0000	NA	-9.7560	Significant
Intergenic	chr17 22181027-22181035	TGTAGGCGT	2.7004	NA	-5.4423	Significant
Intergenic	chr17 22242878-22242886	TGTAGGCGA	-1.0000	NA	-6.1520	Significant
Intergenic	chr17 25301665-25301673	TGTAGGCGT	3.0000	NA	-8.0891	Significant
Intergenic	chr17 27678407-27678415	TCGCCTACA	2.7004	NA	-14.7469	Significant
TAOK1	chr17 27778568-27778575	TCGCCATCA	5.0000	-2.9373	-13.2817	Significant
NF1	chr17 29683828-29683836	TGTAGGCGA	1.1699	-1.2088	-6.8473	Significant
PSMD11	chr17 30790210-30790218	TGATGGCGT	-0.8931	-0.1943	-6.4691	Significant
Intergenic	chr17 31358596-31358604	TGTTGGCGT	5.0000	NA	-7.4699	Significant
Intergenic	chr17 32004947-32004955	TGAAGGCGT	1.1699	NA	-6.4691	Significant
Intergenic	chr17 32745282-32745290	TGTTGGCGA	2.0000	NA	-23.2025	Significant
ACACA	chr17 35612870-35612878	TGTTGGCGA	0.9069	-0.2320	-7.6831	Significant
Intergenic	chr17 36043511-36043519	TGTTGGCGT	0.1926	NA	-9.1342	Significant
SOCS7	chr17 36517750-36517758	TGTAGGCGT	1.3219	-1.3587	-8.7041	Significant
Intergenic	chr17 36792986-36792994	TGTTGGCGA	-0.5850	NA	-7.2864	Significant
Intergenic	chr17 37202731-37202740	ACGCCATCA	2.1155	NA	-5.3906	Significant
Intergenic	chr17 37202732-37202740	TCGCCATCA	2.1155	NA	-11.5683	Significant
NBR1	chr17 41340920-41340928	ACGCCTTCA	5.0000	NA	-5.9014	Significant
NSF	chr17 44796367-44796375	ACGCCTTCA	-1.0875	-0.4871	-5.5994	Significant
Intergenic	chr17 44837069-44837077	TCGCCATCA	-0.5850	NA	-7.4701	Significant
Intergenic	chr17 45083786-45083794	TCGCCAACA	1.3785	NA	-9.7560	Significant
SKAP1	chr17 46258780-46258788	TCGCCATCA	-0.4150	-0.2192	-5.7407	Significant
Intergenic	chr17 46531521-46531529	TGAAGGCGT	0.0000	NA	-12.1150	Significant
SPOP	chr17 47753033-47753041	ACGCCATCA	-1.7162	-2.0097	-9.5736	Significant
Intergenic	chr17 50772067-50772075	TGTTGGCGA	0.1255	NA	-9.1342	Significant
Intergenic	chr17 50937183-50937191	TGATGGCGA	0.3219	NA	-6.8473	Significant
Intergenic	chr17 52863145-52863153	TGATGGCGA	5.0000	NA	-7.4927	Significant
Intergenic	chr17 53432939-53432947	TGAAGGCGT	-0.6280	NA	-6.6939	Significant
Intergenic	chr17 53766781-53766789	ACGCCATCA	0.0000	NA	-4.7182	Significant
PCTP	chr17 53842888-53842896	TCGCCATCA	3.5850	-0.0890	-6.5209	Significant
MSI2	chr17 55409106-55409114	TGAAGGCGT	0.7776	-0.4112	-5.9549	Significant
MSI2	chr17 55628246-55628254	TGTTGGCGT	3.1699	-0.4112	-6.1002	Significant
Intergenic	chr17 55846339-55846347	TCGCCATCA	0.6781	NA	-10.4803	Significant
CUEDC1	chr17 55965701-55965709	ACGCCATCA	5.0000	-0.3373	-8.0891	Significant
CUEDC1	chr17 56025024-56025032	TGATGGCGT	5.0000	-0.3373	-8.5046	Significant
HSF5	chr17 56503764-56503772	ACGCCTTCA	5.0000	0.1606	-9.7564	Significant
Intergenic	chr17 57369441-57369449	ACGCCATCA	3.5850	NA	-11.1427	Significant
CLTC	chr17 57758763-57758771	TCGCCATCA	2.1155	0.0905	-8.4534	Significant
VMP1	chr17 57819551-57819559	ACGCCTACA	-0.3785	-0.4204	-7.0823	Significant
VMP1	chr17 57859013-57859021	ACGCCATCA	5.0000	-0.4204	-4.5890	Significant
RPS6KB1	chr17 58012787-58012795	TGAAGGCGA	3.7004	-1.0085	-5.5996	Significant
BCAS3	chr17 58935397-58935405	TGAAGGCGA	1.0000	-0.4151	-4.7572	Significant
MED13	chr17 60099156-60099164	TCGCCTACA	3.4594	-4.5144	-19.9576	Significant
10-Mar	chr17 60847048-60847056	TGTAGGCGT	1.8074	0.3717	-5.9550	Significant
SMURF2	chr17 62568515-62568523	TCGCCAACA	0.4854	-0.5096	-5.7407	Significant
Intergenic	chr17 63395589-63395597	TGATGGCGA	1.3785	NA	-10.4803	Significant
CEP112	chr17 63809755-63809763	TCGCCTACA	0.2410	0.0742	-5.3906	Significant
PRKCA	chr17 64339738-64339746	TGATGGCGA	5.0000	-0.5320	-8.0378	Significant
PRKCA	chr17 64517416-64517424	TGATGGCGA	2.8074	-0.5320	-8.2802	Significant
PITPNC1	chr17 65383922-65383930	TCGCCATCA	2.4594	-1.1971	-6.1002	Significant
PITPNC1	chr17 65591792-65591800	TGATGGCGA	1.3219	-1.1971	-7.4701	Significant

BPTF	chr17 65918958-65918966	ACGCCTACA	3.4594	-1.3255	-5.3906	Significant
Intergenic	chr17 66833338-66833346	ACGCCATCA	1.0000	NA	-5.7415	Significant
ABCA9	chr17 67008251-67008259	TGATGGCGA	-0.5850	-0.6056	-4.2929	Marginal
Intergenic	chr17 67058394-67058402	TCGCCAACA	2.7004	NA	-12.9104	Significant
Intergenic	chr17 69245408-69245416	TCGCCATCA	5.0000	NA	-16.7036	Significant
Intergenic	chr17 69325735-69325743	TGAAGGCGT	1.8745	NA	-7.6316	Significant
Intergenic	chr17 69523080-69523088	TGATGGCGA	2.0000	NA	-5.7407	Significant
Intergenic	chr17 69531607-69531615	TGAAGGCGT	2.3219	NA	-8.2834	Significant
Intergenic	chr17 70516043-70516051	TGATGGCGT	-1.3785	NA	-6.6939	Significant
LINC00511	chr17 70604468-70604476	TGTTGGCGA	1.4594	-0.5315	-7.6316	Significant
SEC14L1	chr17 75101317-75101325	ACGCCTTCA	1.1699	-0.5120	-5.2533	Significant
TNRC6C	chr17 76073290-76073298	ACGCCTTCA	2.3219	-1.1212	-5.2533	Significant
RPTOR	chr17 78558525-78558533	ACGCCATCA	2.4594	-2.4962	-8.8786	Significant
Intergenic	chr17 79488921-79488929	TGATGGCGT	-1.3785	NA	-6.6939	Significant
COLEC12	chr18 387556-387564	TGAAGGCGT	5.0000	-0.3294	-7.0774	Significant
Intergenic	chr18 888023-888031	TGTTGGCGT	0.5850	NA	-6.3200	Significant
Intergenic	chr18 2111072-2111080	TGAAGGCGA	5.0000	NA	-12.4057	Significant
CBX3P2	chr18 2654535-2654543	ACGCCTTCA	5.0000	0.2139	-17.8716	Significant
DLGAP1	chr18 3579891-3579899	TCGCCTTCA	1.2479	-0.4202	-6.8990	Significant
DLGAP1	chr18 3835876-3835884	TCGCCATCA	5.0000	-0.4202	-28.8799	Significant
L3MBTL4	chr18 6211044-6211052	ACGCCATCA	5.0000	0.2666	-8.9289	Significant
Intergenic	chr18 7377993-7378001	TGAAGGCGA	2.7004	NA	-7.6316	Significant
Intergenic	chr18 8694863-8694869	TGTTGGCGA	2.8074	NA	-9.7568	Significant
PPP4R1	chr18 9613453-9613461	TCGCCATCA	1.5025	-1.3854	-6.1002	Significant
GNAL	chr18 11824952-11824960	TGAAGGCGT	-0.2224	-0.2205	-6.4691	Significant
Intergenic	chr18 12891581-12891589	ACGCCTACA	5.0000	NA	-15.8464	Significant
Intergenic	chr18 18808334-18808342	TGTAGGCGA	0.4854	NA	-8.2844	Significant
Intergenic	chr18 19890696-19890704	TGATGGCGA	3.5850	NA	-8.5043	Significant
Intergenic	chr18 20210320-20210328	ACGCCAACA	5.0000	NA	-10.6711	Significant
Intergenic	chr18 20238663-20238671	TCGCCTACA	5.0000	NA	-9.5737	Significant
TTC39C	chr18 21636604-21636612	TGTTGGCGA	3.8074	-0.3828	-8.2838	Significant
HRH4	chr18 22043924-22043932	TGTTGGCGT	-1.0000	-0.0844	-7.4701	Significant
Intergenic	chr18 25256590-25256598	ACGCCTTCA	2.5850	NA	-6.3300	Significant
Intergenic	chr18 27447522-27447530	TGATGGCGT	5.0000	NA	-5.5996	Significant
Intergenic	chr18 28514382-28514390	TGTTGGCGT	-0.4695	NA	-16.9461	Significant
DSC3	chr18 28584411-28584419	TGAAGGCGT	3.7004	0.0902	-8.2836	Significant
Intergenic	chr18 30115087-30115095	TGTTGGCGA	0.6781	NA	-10.0066	Significant
Intergenic	chr18 30631318-30631326	TGAAGGCGA	5.0000	NA	-11.4197	Significant
Intergenic	chr18 31953046-31953054	TCGCCTTCA	-1.5025	NA	-12.6138	Significant
DTNA	chr18 32317822-32317830	ACGCCTTCA	1.0000	0.1026	-7.0772	Significant
Intergenic	chr18 32491747-32491755	TCGCCTACA	2.5850	NA	-12.6134	Significant
Intergenic	chr18 33134224-33134232	TGAAGGCGT	-0.4594	NA	-6.6938	Significant
Intergenic	chr18 33670556-33670564	TGTTGGCGA	5.0000	NA	-7.0772	Significant
Intergenic	chr18 35191382-35191390	TCGCCTTCA	2.7004	NA	-5.3906	Significant
Intergenic	chr18 35779485-35779493	TGTTGGCGT	-1.6439	NA	-5.2533	Significant
Intergenic	chr18 35982621-35982629	TGATGGCGT	5.0000	NA	-13.6409	Significant
Intergenic	chr18 36848692-36848700	ACGCCATCA	3.5850	NA	-6.1129	Significant
Intergenic	chr18 37801626-37801634	TGAAGGCGT	3.3219	NA	-8.5043	Significant
Intergenic	chr18 38958302-38958310	TCGCCAACA	1.7370	NA	-9.1342	Significant
Intergenic	chr18 39263746-39263754	TGTTGGCGA	-1.4594	NA	-8.4444	Significant
Intergenic	chr18 40123388-40123396	TGAAGGCGT	1.1699	NA	-5.7407	Significant
RIT2	chr18 40370896-40370904	ACGCCATCA	1.7370	0.2700	-4.9165	Significant
ST8SIA5	chr18 44264626-44264634	ACGCCTTCA	3.1699	0.0970	-7.8722	Significant
SMAD2	chr18 45403179-45403187	TCGCCTACA	5.0000	-1.0348	-8.8782	Significant
Intergenic	chr18 45539639-45539647	TGTTGGCGT	0.7105	NA	-5.5996	Significant
ZBTB7C	chr18 45636908-45636916	TGTTGGCGT	2.0000	-0.9001	-13.9476	Significant
Intergenic	chr18 45848921-45848929	TGTTGGCGT	0.0995	NA	-6.4691	Significant
Intergenic	chr18 45951743-45951751	TCGCCATCA	3.5850	NA	-7.4699	Significant
Intergenic	chr18 47187488-47187496	TCGCCATCA	0.5850	NA	-4.2711	Marginal
Intergenic	chr18 47203217-47203225	TGTAGGCGA	5.0000	NA	-9.3629	Significant
Intergenic	chr18 47267723-47267731	TGATGGCGT	2.5850	NA	-10.9249	Significant
MAPK4	chr18 48132576-48132585	TGATGGCGA	2.4594	0.0916	-9.3132	Significant
MAPK4	chr18 48132577-48132585	TGATGGCGA	2.4594	0.0916	-5.2533	Significant
ELAC1	chr18 48500858-48500866	TGAAGGCGA	0.2895	-0.7778	-6.8990	Significant
Intergenic	chr18 49319953-49319961	TGTTGGCGT	-1.0589	NA	-7.0772	Significant

Intergenic	chr18	49779615-49779623	ACGCCAACA	2.8074	NA	-17.8714	Significant
DCC	chr18	49976547-49976555	TCGCCAACA	-0.1375	-0.0117	-8.7043	Significant
CCDC68	chr18	52588772-52588780	ACGCCTACA	5.0000	-1.1845	-5.9672	Significant
Intergenic	chr18	52744472-52744480	TGATGGCGA	5.0000	NA	-4.7695	Significant
Intergenic	chr18	54261511-54261519	TCGCCAACA	1.4150	NA	-11.1907	Significant
Intergenic	chr18	54938679-54938687	TCGCCTTCA	1.8745	NA	-6.4695	Significant
Intergenic	chr18	55062842-55062850	ACGCCTACA	4.0875	NA	-14.2110	Significant
NEDD4L	chr18	55756229-55756237	TCGCCTACA	1.2224	-1.5152	-5.7407	Significant
ZNF532	chr18	56530174-56530182	TGAAGGCGA	0.2224	-1.5978	-6.1002	Significant
Intergenic	chr18	58177687-58177695	ACGCCAACA	1.8745	NA	-8.0893	Significant
Intergenic	chr18	58567679-58567687	TGTAGGCGA	-0.9175	NA	-6.4695	Significant
Intergenic	chr18	59061239-59061247	TGATGGCGT	0.7370	NA	-6.3200	Significant
BCL2	chr18	60792487-60792495	TGTTGGCGT	1.4150	-1.8157	-4.2708	Marginal
VPS4B	chr18	61057454-61057462	TCGCCATCA	1.7370	-1.7021	-6.3196	Significant
SERPINB2	chr18	61570283-61570291	ACGCCTTCA	3.4594	0.1858	-5.9550	Significant
Intergenic	chr18	62182968-62182976	ACGCCAACA	1.4150	NA	-8.0891	Significant
Intergenic	chr18	62641034-62641042	TGTTGGCGT	0.5850	NA	-5.3906	Significant
Intergenic	chr18	63588817-63588825	TGTAGGCGT	3.4594	NA	-7.6320	Significant
Intergenic	chr18	64149788-64149796	ACGCCAACA	1.8745	NA	-5.0501	Significant
Intergenic	chr18	64377134-64377142	TCGCCAACA	0.2410	NA	-5.7407	Significant
Intergenic	chr18	64426260-64426268	ACGCCTACA	2.7004	NA	-9.3631	Significant
LOC643542	chr18	65243083-65243091	TGAAGGCGT	-0.4406	0.0580	-9.7560	Significant
LOC643542	chr18	65338484-65338492	TCGCCTTCA	0.8480	0.0580	-8.4534	Significant
LOC643542	chr18	65469175-65469183	TCGCCTACA	5.0000	0.0580	-9.1249	Significant
DOK6	chr18	67115338-67115346	TCGCCATCA	-0.2410	0.0622	-9.5613	Significant
Intergenic	chr18	67637026-67637034	TCGCCAACA	-2.8580	NA	-13.4243	Significant
Intergenic	chr18	68045578-68045586	ACGCCTTCA	-0.5850	NA	-6.1002	Significant
Intergenic	chr18	68498717-68498725	ACGCCATCA	2.9069	NA	-10.2089	Significant
Intergenic	chr18	70060091-70060099	TGTTGGCGA	5.0000	NA	-9.1249	Significant
Intergenic	chr18	71132417-71132425	TGTAGGCGA	2.1699	NA	-10.9478	Significant
Intergenic	chr18	71382021-71382029	TCGCCTTCA	-0.6781	NA	-8.5043	Significant
Intergenic	chr18	71401560-71401568	TGTTGGCGT	0.0000	NA	-10.2089	Significant
Intergenic	chr18	71438585-71438593	TCGCCTTCA	-0.5850	NA	-7.8720	Significant
CNDP1	chr18	72242038-72242046	TGATGGCGT	-1.0000	0.0455	-6.1520	Significant
Intergenic	chr18	73541201-73541208	TGTTGGCGT	5.0000	NA	-8.4534	Significant
ZNF516	chr18	74085466-74085474	ACGCCTACA	0.8480	-4.1776	-13.6844	Significant
ZNF236	chr18	74590623-74590631	TGTAGGCGT	5.0000	-1.6422	-7.0823	Significant
ZNF236	chr18	74627606-74627614	TGAAGGCGT	2.4594	-1.6422	-7.6316	Significant
ATP9B	chr18	76909889-76909897	TCGCCAACA	5.0000	0.1557	-11.6711	Significant
Intergenic	chr19	2017475-2017483	TGATGGCGA	5.0000	NA	-9.7560	Significant
SAFB	chr19	5631920-5631928	TGATGGCGT	5.0000	-1.8742	-8.2805	Significant
FUT3	chr19	5849192-5849200	TCGCCTTCA	1.2224	-1.2660	-6.4691	Significant
RANBP3	chr19	5966931-5966939	TCGCCAACA	0.3626	-1.3140	-6.4691	Significant
Intergenic	chr19	9750689-9750697	TGAAGGCGA	2.4594	NA	-8.9291	Significant
Intergenic	chr19	19067753-19067761	TGTTGGCGA	2.5850	NA	-8.8786	Significant
Intergenic	chr19	19091582-19091590	TGTTGGCGT	5.0000	NA	-9.1342	Significant
Intergenic	chr19	21045236-21045244	ACGCCATCA	3.7004	NA	-8.7041	Significant
Intergenic	chr19	22064106-22064114	TCGCCATCA	-0.4150	NA	-9.5737	Significant
Intergenic	chr19	23582019-23582027	ACGCCAACA	1.1699	NA	-10.6715	Significant
ZNF726	chr19	24110965-24110973	TCGCCTTCA	5.0000	-0.8286	-5.3906	Significant
Intergenic	chr19	27754840-27754848	ACGCCTTCA	1.7370	NA	-4.5891	Significant
Intergenic	chr19	28778513-28778521	TCGCCATCA	1.2630	NA	-7.6320	Significant
Intergenic	chr19	28853938-28853946	TGAAGGCGA	-1.0641	NA	-12.9102	Significant
Intergenic	chr19	30141154-30141162	TGTTGGCGT	1.3219	NA	-8.2834	Significant
Intergenic	chr19	30671383-30671391	ACGCCTTCA	1.0000	NA	-7.0772	Significant
Intergenic	chr19	34092513-34092521	TCGCCAACA	1.0000	NA	-5.7407	Significant
Intergenic	chr19	35113058-35113065	ACGCCATCA	2.0000	NA	-6.6938	Significant
Intergenic	chr19	36924623-36924631	TGATGGCGA	0.4150	NA	-5.1013	Significant
ZNF382	chr19	37118323-37118331	TCGCCATCA	5.0000	0.1407	-9.3124	Significant
Intergenic	chr19	37490319-37490327	TGAAGGCGT	5.0000	NA	-4.9165	Significant
Intergenic	chr19	39242347-39242355	TCGCCTTCA	1.8745	NA	-6.8473	Significant
Intergenic	chr19	42173193-42173201	TGAAGGCGA	2.8074	NA	-10.2093	Significant
Intergenic	chr19	42971867-42971875	TGTAGGCGT	0.7004	NA	-5.7411	Significant
Promoter_LOC100289650	chr19	43325653-43325661	TGTTGGCGA	3.1699	0.0772	-7.0772	Significant
Intergenic	chr19	43846225-43846233	TGTTGGCGA	5.0000	NA	-7.8717	Significant

ZNF224	chr19 44611376-44611384	TGTAGGCGA	2.4594	-0.5855	-9.1342	Significant
Intergenic	chr19 44830423-44830431	TCGCCAACA	1.6781	NA	-5.0497	Significant
PPP1R13L	chr19 45892197-45892205	TGTTGGCGA	2.0000	-1.5692	-6.3196	Significant
Intergenic	chr19 46497132-46497140	TGATGGCGT	5.0000	NA	-9.4331	Significant
Intergenic	chr19 46780214-46780222	TGAAGGCGA	5.0000	NA	-6.8473	Significant
Intergenic	chr19 47052137-47052141	TCGCCTACA	1.7370	NA	-7.2352	Significant
ELSPBP1	chr19 48522735-48522743	TGATGGCGA	1.3785	0.1715	-6.5211	Significant
PPP2R1A	chr19 52706462-52706470	TCGCCTACA	5.0000	0.2476	-14.7050	Significant
ZNF83	chr19 53176000-53176008	TCGCCATCA	3.7004	-0.5500	-9.3124	Significant
RPS9	chr19 54709052-54709060	TGAAGGCGT	2.5850	0.5421	-11.1909	Significant
Intergenic	chr19 56267394-56267402	TGTTGGCGA	-1.0000	NA	-6.3196	Significant
Intergenic	chr19 57016325-57016333	TGATGGCGT	5.0000	NA	-13.1671	Significant
ANGPT4	chr20 882796-882804	TGATGGCGT	0.8745	0.2216	-7.0775	Significant
PSMF1	chr20 1106813-1106821	ACGCCATCA	-0.1375	-1.0683	-5.4421	Significant
Intergenic	chr20 1711995-1712003	TGAAGGCGA	1.4594	NA	-5.1011	Significant
Intergenic	chr20 2179173-2179181	ACGCCAACA	-0.6280	NA	-4.9165	Significant
Intergenic	chr20 3409848-3409856	TGAAGGCGT	0.7370	NA	-4.9165	Significant
PRNP	chr20 4676906-4676914	TCGCCATCA	2.2224	0.4786	-7.8722	Significant
Intergenic	chr20 5473931-5473939	TGTTGGCGT	3.8074	NA	-13.9476	Significant
CHGB	chr20 5904038-5904046	ACGCCATCA	5.0000	0.2012	-9.7287	Significant
Intergenic	chr20 7279053-7279061	ACGCCAACA	5.0000	NA	-4.4380	Significant
PLCB1	chr20 8244111-8244119	ACGCCTTCA	2.8074	-0.3493	-10.0224	Significant
PLCB4	chr20 9164832-9164840	TGAAGGCGA	5.0000	-0.4784	-4.5890	Significant
Intergenic	chr20 10191743-10191751	ACGCCATCA	3.7004	NA	-8.0378	Significant
Intergenic	chr20 10368202-10368210	ACGCCTTCA	2.0000	NA	-7.6831	Significant
JAG1	chr20 10639916-10639924	TGATGGCGA	3.1699	-1.4558	-5.7407	Significant
Intergenic	chr20 10661227-10661235	TGTAGGCGT	-0.3219	NA	-7.2348	Significant
Intergenic	chr20 11100447-11100455	TGATGGCGT	5.0000	NA	-10.6301	Significant
Intergenic	chr20 11362853-11362861	ACGCCTACA	1.4594	NA	-9.5736	Significant
Intergenic	chr20 11555803-11555811	ACGCCAACA	1.0000	NA	-5.2534	Significant
Intergenic	chr20 12141388-12141396	ACGCCTTCA	3.7004	NA	-8.7041	Significant
Intergenic	chr20 12271969-12271977	TGTAGGCGA	1.3219	NA	-5.0497	Significant
Intergenic	chr20 12975889-12975897	TCGCCATCA	5.0000	NA	-11.8800	Significant
SEL1L2	chr20 13912759-13912767	TGAAGGCGT	0.8745	0.1024	-6.8473	Significant
MACROD2	chr20 15697550-15697558	TGTTGGCGA	2.0000	-0.1931	-8.0378	Significant
DTD1	chr20 18572982-18572990	TGATGGCGT	5.0000	0.0761	-11.1907	Significant
Intergenic	chr20 19856012-19856020	TGTTGGCGA	-0.6630	NA	-6.6938	Significant
Intergenic	chr20 20274432-20274440	TGTTGGCGT	3.5850	NA	-6.5209	Significant
Intergenic	chr20 22431968-22431976	TCGCCATCA	2.8074	NA	-8.5043	Significant
Intergenic	chr20 22487104-22487112	TCGCCATCA	1.4594	NA	-7.4699	Significant
Intergenic	chr20 24797926-24797934	ACGCCATCA	2.5850	NA	-8.9289	Significant
Intergenic	chr20 24801942-24801950	ACGCCATCA	1.3219	NA	-7.4699	Significant
Intergenic	chr20 29910849-29910857	TCGCCATCA	2.0000	NA	-7.4699	Significant
Intergenic	chr20 29910867-29910875	TCGCCAACA	1.6781	NA	-6.6939	Significant
TM9SF4	chr20 30705535-30705543	TGATGGCGT	0.0000	-0.5178	-6.1006	Significant
KIF3B	chr20 30889845-30889853	TGAAGGCGA	1.5850	-1.1140	-6.9960	Significant
Intergenic	chr20 33272499-33272507	TGAAGGCGT	0.1926	NA	-6.3196	Significant
TTI1	chr20 36636544-36636552	TGATGGCGT	2.7004	-1.5272	-18.0928	Significant
RALGAPB	chr20 37176713-37176721	TGAAGGCGT	5.0000	-2.7785	-4.7693	Significant
RALGAPB	chr20 37200498-37200506	TCGCCAACA	0.5305	-2.7785	-8.2834	Significant
Intergenic	chr20 38419581-38419589	TCGCCAACA	2.4594	NA	-7.8722	Significant
Intergenic	chr20 39271567-39271575	TGAAGGCGA	1.8745	NA	-6.8473	Significant
ZHX3	chr20 39911866-39911874	TCGCCATCA	5.0000	-1.8668	-5.0497	Significant
CHD6	chr20 40203488-40203496	ACGCCTACA	3.8074	-2.8953	-7.0774	Significant
PTPRT	chr20 40762872-40762880	TGAAGGCGT	3.4594	0.0406	-7.6316	Significant
PTPRT	chr20 41063249-41063257	ACGCCTTCA	0.4854	0.0406	-6.6938	Significant
PTPRT	chr20 41403860-41403868	ACGCCTACA	2.5850	0.0406	-5.2533	Significant
PTPRT	chr20 41759635-41759643	ACGCCTACA	0.0000	0.0406	-8.0378	Significant
SERINC3	chr20 43136982-43136990	TGTAGGCGT	0.3219	-0.0804	-6.8473	Significant
STK4	chr20 43676761-43676769	ACGCCAACA	3.4594	-1.4501	-10.6711	Significant
Intergenic	chr20 43746510-43746518	ACGCCTTCA	5.0000	NA	-6.4866	Significant
Intergenic	chr20 44723411-44723419	ACGCCAACA	5.0000	NA	-5.5994	Significant
EYA2	chr20 45806461-45806469	TGATGGCGA	3.8074	-0.0242	-9.1342	Significant
Intergenic	chr20 46808248-46808256	TCGCCATCA	3.3219	NA	-8.0378	Significant
KCNB1	chr20 48028527-48028535	TGTTGGCGA	1.2224	0.2146	-6.1002	Significant

Intergenic	chr20 48638625-48638633	TCGCCAACA	2.7004	NA	-6.3196	Significant
Intergenic	chr20 48950695-48950703	TGAAGGCGA	0.6781	NA	-7.0772	Significant
PTPN1	chr20 49199598-49199606	ACGCCAACA	5.0000	-2.5854	-6.1002	Significant
Intergenic	chr20 49887120-49887128	TGATGGCGT	5.0000	NA	-6.3198	Significant
TSHZ2	chr20 51615375-51615383	TGTAGGCGT	5.0000	0.0204	-13.4241	Significant
Intergenic	chr20 52213275-52213283	TCGCCATCA	5.0000	NA	-5.2533	Significant
CYP24A1	chr20 52773004-52773012	TCGCCTTCA	0.7370	0.4247	-5.9550	Significant
Intergenic	chr20 53809435-53809443	TCGCCATCA	1.1699	NA	-5.4421	Significant
Intergenic	chr20 54374049-54374057	ACGCCTACA	5.0000	NA	-4.9350	Significant
Intergenic	chr20 54403149-54403157	TCGCCAACA	2.5850	NA	-7.6831	Significant
Intergenic	chr20 54840764-54840772	TCGCCATCA	2.0000	NA	-14.4800	Significant
CTCFL	chr20 56073643-56073651	ACGCCTTCA	5.0000	-0.0401	-7.6596	Significant
Intergenic	chr20 56444195-56444203	TGATGGCGA	2.9069	NA	-11.1427	Significant
Intergenic	chr20 59147057-59147065	ACGCCTTCA	1.4594	NA	-7.0772	Significant
Intergenic	chr20 59286659-59286667	TGTTGGCGA	3.3219	NA	-6.6938	Significant
CDH4	chr20 60077765-60077773	TCGCCAACA	2.3219	-0.0901	-13.4241	Significant
Intergenic	chr21 10188434-10188442	TCGCCTTCA	1.5850	NA	-5.7407	Significant
Intergenic	chr21 14388469-14388477	TGATGGCGT	5.0000	NA	-6.1002	Significant
ANKRD30BP2	chr21 14436303-14436311	TGATGGCGA	5.0000	-0.5880	-8.8782	Significant
ANKRD30BP2	chr21 14445482-14445490	TCGCCATCA	1.5850	-0.5880	-12.6591	Significant
Intergenic	chr21 16805833-16805841	TCGCCAACA	-1.2479	NA	-8.7041	Significant
Intergenic	chr21 16860177-16860185	ACGCCAACA	1.0000	NA	-8.8782	Significant
Intergenic	chr21 16885390-16885398	TCGCCAACA	5.0000	NA	-4.7186	Significant
Intergenic	chr21 17063577-17063585	TCGCCATCA	5.0000	NA	-12.9102	Significant
USP25	chr21 17140685-17140693	TGTTGGCGT	2.8074	-1.6853	-10.0223	Significant
Intergenic	chr21 17587489-17587497	TGATGGCGA	0.8745	NA	-9.5736	Significant
Intergenic	chr21 19124808-19124816	ACGCCATCA	3.7004	NA	-6.3196	Significant
CHODL	chr21 19314278-19314286	ACGCCTTCA	5.0000	0.2059	-5.7407	Significant
CHODL	chr21 19420531-19420539	TGATGGCGT	5.0000	0.2059	-8.4534	Significant
Intergenic	chr21 21782762-21782770	ACGCCATCA	5.0000	NA	-6.4691	Significant
Intergenic	chr21 22087830-22087838	ACGCCAACA	1.1844	NA	-9.1342	Significant
NCAM2	chr21 22595871-22595879	TCGCCAACA	5.0000	0.1314	-4.2708	Marginal
NCAM2	chr21 22759201-22759209	TGTTGGCGT	5.0000	0.1314	-5.3906	Significant
Intergenic	chr21 23397229-23397237	TCGCCAACA	2.0000	NA	-6.1006	Significant
Intergenic	chr21 24818384-24818392	ACGCCTTCA	1.0000	NA	-10.4804	Significant
Intergenic	chr21 25318604-25318612	TCGCCAACA	3.4594	NA	-7.0772	Significant
Intergenic	chr21 25820342-25820350	TCGCCAACA	3.5850	NA	-4.7693	Significant
Intergenic	chr21 25916695-25916703	ACGCCTACA	-0.7885	NA	-6.8473	Significant
APP	chr21 27485027-27485035	TGTTGGCGA	-0.4475	0.0586	-8.2834	Significant
CYYR1	chr21 27942069-27942077	TCGCCATCA	5.0000	0.2282	-8.8786	Significant
Intergenic	chr21 30454246-30454254	TGTAGGCGA	0.5850	NA	-6.1002	Significant
LINC00189	chr21 30577049-30577057	TCGCCTTCA	2.4594	0.2609	-8.0378	Significant
LINC00189	chr21 30610481-30610489	ACGCCAACA	1.7370	0.2609	-8.0382	Significant
Intergenic	chr21 31846072-31846080	TCGCCTTCA	1.7370	NA	-8.0382	Significant
Intergenic	chr21 32122776-32122784	TCGCCAACA	1.7370	NA	-5.2534	Significant
TIAM1	chr21 32663045-32663053	TGATGGCGA	2.4594	-0.2072	-6.1526	Significant
HUNK	chr21 33276900-33276908	TGTTGGCGT	2.8074	-0.9893	-6.7019	Significant
Intergenic	chr21 33917188-33917196	ACGCCTTCA	1.7004	NA	-13.9006	Significant
SYNJ1	chr21 34086695-34086703	TGTAGGCGT	1.1699	-0.4299	-7.2348	Significant
Intergenic	chr21 34378601-34378609	TCGCCAACA	2.4594	NA	-12.4056	Significant
Intergenic	chr21 34489540-34489548	TCGCCAACA	1.3219	NA	-5.5996	Significant
ITSN1	chr21 35098063-35098071	TGATGGCGT	1.0995	-0.5364	-8.8782	Significant
LINC00310	chr21 35557014-35557022	TGATGGCGA	0.5146	-0.1052	-8.2836	Significant
RCAN1	chr21 35935225-35935233	ACGCCATCA	5.0000	-1.5731	-32.7321	Significant
RCAN1	chr21 35940778-35940786	TGTTGGCGA	3.5850	-1.5731	-6.8473	Significant
Intergenic	chr21 37378031-37378039	TCGCCAACA	1.4854	NA	-5.3910	Significant
LOC100133286	chr21 37476286-37476294	TCGCCTACA	2.0000	0.3430	-7.6831	Significant
DOPEY2	chr21 37586790-37586798	ACGCCATCA	1.4854	-0.7805	-8.0378	Significant
Intergenic	chr21 39714800-39714807	TGTTGGCGA	5.0000	NA	-6.6938	Significant
Intergenic	chr21 40403232-40403240	ACGCCATCA	5.0000	NA	-11.6236	Significant
Intergenic	chr21 40471060-40471068	TGTTGGCGT	1.3785	NA	-7.8724	Significant
HMGN1	chr21 40719038-40719046	ACGCCAACA	5.0000	0.3211	-4.5890	Significant
Intergenic	chr21 41059063-41059071	TCGCCTTCA	1.3219	NA	-6.8992	Significant
DSCAM	chr21 41675318-41675326	ACGCCTTCA	0.4594	0.1648	-8.0378	Significant
DSCAM	chr21 41806247-41806255	ACGCCAACA	1.7370	0.1648	-8.0382	Significant

FAM3B	chr21	42711573-42711581	TCGCCTTCA	3.3219	0.1478	-6.5209	Significant
Intergenic	chr21	42947308-42947316	TGTTGGCGT	5.0000	NA	-8.4534	Significant
C2CD2	chr21	43315587-43315595	ACGCCAACA	1.1155	-2.2388	-7.0774	Significant
SLC37A1	chr21	43925159-43925167	ACGCCAACA	0.7004	-2.1231	-7.6833	Significant
PDE9A	chr21	44132472-44132480	TGTTGGCGT	-1.4948	-0.9637	-7.8720	Significant
RRP1B	chr21	45092196-45092204	TCGCCAACA	5.0000	-0.7343	-14.7050	Significant
Intergenic	chr21	45118507-45118515	TCGCCATCA	0.8074	NA	-6.4691	Significant
Intergenic	chr21	47366309-47366317	TCGCCAACA	1.8074	NA	-9.5613	Significant
SPECC1L	chr22	24755263-24755271	ACGCCATCA	0.6781	-1.9782	-9.1342	Significant
SGSM1	chr22	25318169-25318177	TGATGGCGT	0.6280	0.1385	-8.4534	Significant
ADRBK2	chr22	25962687-25962695	ACGCCTACA	3.7004	-2.6105	-6.8473	Significant
NF2	chr22	30043416-30043424	TGTAGGCGA	2.5850	-0.9397	-7.8720	Significant
HORMAD2	chr22	30478494-30478502	TGAAGGCGT	5.0000	-0.0718	-16.8079	Significant
OSBP2	chr22	31118303-31118311	TGTTGGCGT	1.7370	-0.3322	-8.0382	Significant
SFI1	chr22	31943016-31943024	TGTTGGCGT	5.0000	-0.3950	-9.5737	Significant
Intergenic	chr22	32757308-32757316	TCGCCATCA	1.8074	NA	-10.2089	Significant
Intergenic	chr22	35326908-35326916	TCGCCATCA	1.7370	NA	-8.0382	Significant
Intergenic	chr22	35522793-35522801	TGAAGGCGT	5.0000	NA	-4.2933	Marginal
HMGXB4	chr22	35679060-35679068	TGAAGGCGT	5.0000	-2.9730	-7.4701	Significant
RBFOX2	chr22	36189375-36189383	ACGCCATCA	-0.5850	-2.0108	-6.8477	Significant
ENTHD1	chr22	40163200-40163208	ACGCCTTCA	2.4594	0.3021	-6.3198	Significant
EFCAB6	chr22	44158158-44158166	TGTAGGCGA	1.7370	0.1206	-8.0382	Significant
ATXN10	chr22	46103980-46103988	TGTAGGCGA	5.0000	-0.1810	-5.1011	Significant
CERK	chr22	47106955-47106963	TGATGGCGA	1.7370	-0.5848	-6.3198	Significant
TBC1D22A	chr22	47464822-47464830	TGAAGGCGA	-0.3626	-0.5689	-6.1002	Significant
Intergenic	chr22	49166707-49166715	TCGCCTTCA	5.0000	NA	-6.5209	Significant
Intergenic	chrX	11708562-11708570	TCGCCATCA	-0.2410	NA	-10.6711	Significant
Intergenic	chrX	30027246-30027254	ACGCCTACA	5.0000	NA	-7.2348	Significant
Intergenic	chrX	41801228-41801236	TGAAGGCGA	3.8074	NA	-7.6339	Significant
