# Supplementary material for: Identification of Binding Targets of a Pyrrole-Imidazole Polyamide KR12 in the LS180 Colorectal Cancer Genome
Source: PLoS One. 2016 Oct 31;11(10):e0165581. doi: 10.1371/journal.pone.0165581 (PMC5087912; doi:10.1371/journal.pone.0165581)
Supplement: S2 Appendix — (GZ) [file pone.0165581.s002.gz › S2 Appendix.pdf]

S2 Appendix. Sequence similarity among KR12-bound lncRNAs and the KRAS transcript. 9-mer sequences in the LS180 genome were counted in regions within 2000 bp of an experimentally determined KR12-binding site in lncRNA stretches and the *KRAS* transcript (chr12: 25,357,722-25,403,865) and their sequence similarities (in turns of k-mer frequencies) compared. "lncRNA" indicates the frequency of a particular 9-mer ("Motif") found within a lncRNA range; similarly, "KRAS" indicates the frequency of the same motif found within the transcript of *KRAS*. Results in "S2 Appendix_lncRNA-masked.txt" indicate 9-mer frequencies determined after filtering for simple tandem repeats by tantan. 138 sites were found within lncRNA regions while none resided within miRNA regions. Between those two sets of 9-mers, 26,222 were identical, with the 9-mer spanning *KRAS* codon 12 in LS180 at the 97.5 percentile; after masking, the KR12 motif was found at the 98.6 percentile among 25,019 identical 9-mers.
