# Supplementary material for: Identification of Binding Targets of a Pyrrole-Imidazole Polyamide KR12 in the LS180 Colorectal Cancer Genome
Source: PLoS One. 2016 Oct 31;11(10):e0165581. doi: 10.1371/journal.pone.0165581 (PMC5087912; doi:10.1371/journal.pone.0165581)
Supplement: S3 Appendix — (PDF) [file pone.0165581.s003.pdf]

S3 Appendix. The list of identified KR12 binding sites in the SW480 genome, organized by RefSeq gene symbols and genomic positions in hg19 coordinates. "Pattern" indicates motif of the binding site on the (+) strand. Fold enrichments ("FE"), changes in gene expressions ("FC") and *p*-values ("p_{BH99}") are listed in $\log_2$ scales. Fold enrichments are defined as the ratio of the maximum per-base coverage within a 1000-bp window centered at the KR12 binding site for the pulldown and input data. Gene expressions are experimentally determined fold changes for KR12 vs. DMSO (see "Expression Microarrays") for sites residing within the transcript or promoter (defined as within 1000 bp upstream of the transcription start site) of a particular gene, and NA otherwise. The 99th percentile p-value, after Benjamini-Hochberg corrections for multiple Kolmogorov-Smirnov comparisons, is listed for a given KR12 binding site. Under "Significance", if $p_{BH99} < 0.05$ for a particular site, it is considered significant; for sites with $0.05 < p_{BH99} < 0.055$, they are considered marginal; sites listed as "Reference" were coordinates from the LS180 genome, and their $\log_2$FE values were determined by computing the differential coverage 1000 bp within their KR12 sites. Sites with $p_{BH99} \geq 0.055$ are deemed statistically insignificant and consequently omitted.

Symbol	Chromosome	Site Position	Pattern	log_2 FE	log_2 FC	log_2 p_{BH99}	Significance
Intergenic	chr4	31884403-31884411	TGTTGGCGT	4.8074	NA	-52.0000	Significant
Intergenic	chr4	43378699-43378707	ACGCCTACA	3.8074	NA	-29.8971	Significant
Intergenic	chr13	32003630-32003638	TCGCCAACA	3.4594	NA	-14.7638	Significant
Intergenic	chr12	84539331-84539339	TGTAGGCGT	3.3219	NA	-28.2385	Significant
GPR39	chr2	133260117-133260125	TGTAGGCGA	3.2928	-0.2397	-14.4650	Significant
Intergenic	chr10	89095007-89095015	TCGCCTACA	3.2479	NA	-23.9133	Significant
Intergenic	chr4	183068092-183068100	TGTAGGCGT	3.1699	NA	-13.0137	Significant
Intergenic	chr2	138627213-138627221	TGTTGGCGT	3.0704	NA	-18.9089	Significant
Intergenic	chr1	79594563-79594571	TCGCCTACA	3.0000	NA	-10.5816	Significant
Intergenic	chr14	43721883-43721891	TCGCCAACA	3.0000	NA	-18.2397	Significant
Intergenic	chr13	65225626-65225634	TGTAGGCGA	2.8826	NA	-16.9357	Significant
Intergenic	chr2	6729531-6729539	TCGCCATCA	2.8804	NA	-8.6142	Significant
CSMD3	chr8	114413733-114413741	TCGCCTACA	2.8745	-0.4165	-11.3666	Significant
Intergenic	chr19	31257777-31257785	TCGCCTTCA	2.8745	NA	-15.3701	Significant
Intergenic	chr2	4492744-4492752	TGTTGGCGA	2.8413	NA	-8.3811	Significant
SESTD1	chr2	180119268-180119276	ACGCCATCA	2.8074	-0.5706	-16.9357	Significant
SP100	chr2	231300572-231300580	TCGCCATCA	2.8074	0.1769	-13.0137	Significant
Intergenic	chr9	105315815-105315823	TGTTGGCGA	2.8074	NA	-16.9357	Significant
VEPH1	chr3	157176106-157176114	TCGCCTTCA	2.7549	0.2563	-6.4122	Significant
LUZP4	chrX	114536640-114536648	TCGCCTTCA	2.7370	-0.0058	-37.4566	Significant
ZRSR2	chrX	15833576-15833584	ACGCCAACA	2.6845	-0.9659	-17.9095	Significant
Intergenic	chr3	109709876-109709884	TCGCCAACA	2.6630	NA	-16.3009	Significant
VSNL1	chr2	17814370-17814378	TGAAGGCGA	2.6323	-0.4137	-7.4774	Significant
Intergenic	chr2	104673890-104673898	TGATGGCGT	2.5850	NA	-12.4532	Significant
Intergenic	chr4	180635831-180635839	TGATGGCGA	2.5850	NA	-8.8501	Significant
NT5DC1	chr6	116476546-116476554	TCGCCAACA	2.5850	0.5000	-11.6339	Significant
TPK1	chr7	144442441-144442449	ACGCCATCA	2.5850	-0.5778	-14.4650	Significant
Intergenic	chr10	105929069-105929077	TCGCCAACA	2.5850	NA	-15.9878	Significant
Intergenic	chr12	115024070-115024078	TGATGGCGA	2.5850	NA	-15.0655	Significant
Intergenic	chr14	46417983-46417991	ACGCCAACA	2.5850	NA	-9.5750	Significant
DOK6	chr18	67382997-67383005	TGTTGGCGT	2.5850	0.0436	-16.3009	Significant
DYTN	chr2	207527628-207527636	TGATGGCGT	2.5361	0.3476	-19.5895	Significant
CDC5L	chr6	44375202-44375210	TGTAGGCGT	2.5146	-0.4484	-13.5857	Significant
LRRC7	chr1	70543311-70543319	TCGCCTACA	2.5025	-0.4462	-14.4650	Significant
Intergenic	chr1	192590029-192590037	TGTTGGCGT	2.5025	NA	-21.3410	Significant
Intergenic	chr13	27007266-27007274	TCGCCATCA	2.4594	NA	-10.0726	Significant
Intergenic	chr14	81407324-81407332	ACGCCTACA	2.4594	NA	-15.6775	Significant
Intergenic	chr19	54753476-54753484	TGTAGGCGT	2.4594	NA	-20.9850	Significant
POLD2	chr7	44161186-44161194	TCGCCATCA	2.4448	0.2788	-10.3257	Significant
Intergenic	chr10	10740746-10740754	ACGCCAACA	2.4330	NA	-15.3701	Significant
Intergenic	chr12	102325013-102325021	TCGCCAACA	2.4330	NA	-11.3666	Significant
NEK7	chr1	198194093-198194101	TGTAGGCGT	2.4150	0.0839	-11.9042	Significant
Intergenic	chr15	58024760-58024768	TGAAGGCGT	2.4150	NA	-13.2983	Significant
DLG2	chr11	84682896-84682904	TGTAGGCGA	2.4021	0.2531	-15.9878	Significant
C4orf19	chr4	37521193-37521201	TGTTGGCGT	2.3923	-0.1464	-11.3666	Significant
RFC1	chr4	39306482-39306490	TCGCCTTCA	2.3923	-0.2046	-14.7638	Significant
Intergenic	chr8	130416076-130416084	TCGCCATCA	2.3923	NA	-16.6169	Significant
PLEKHA5	chr12	19471045-19471053	TGAAGGCGT	2.3923	-1.1084	-7.6991	Significant
AFF1	chr4	87991357-87991365	TGTTGGCGA	2.3785	-0.4536	-11.6339	Significant
CMAHP	chr6	25117110-25117118	TGAAGGCGA	2.3785	-0.0006	-10.8404	Significant
Intergenic	chr6	50537292-50537300	TGATGGCGA	2.3785	NA	-9.3305	Significant
GABRA3	chrX	151411214-151411222	TGTTGGCGA	2.3785	0.2267	-7.2587	Significant
Promoter_MARC2	chr1	220921061-220921069	TGTAGGCGA	2.3692	0.4573	-15.0655	Significant
ADAM12	chr10	127717075-127717083	ACGCCTACA	2.3692	0.0409	-13.8759	Significant
MCC	chr5	112624766-112624774	ACGCCTACA	2.3458	-0.1549	-10.3257	Significant
Intergenic	chr1	74295658-74295666	TGTAGGCGT	2.3219	NA	-9.0889	Significant
Intergenic	chr1	83589044-83589052	TCGCCTTCA	2.3219	NA	-10.3257	Significant
GLUL	chr1	182355559-182355567	ACGCCATCA	2.3219	0.6574	-12.1773	Significant
Intergenic	chr2	189541305-189541313	TGATGGCGA	2.3219	NA	-10.5816	Significant
Intergenic	chr3	103234794-103234802	TGATGGCGA	2.3219	NA	-13.2983	Significant
Intergenic	chr3	116225015-116225023	TGATGGCGA	2.3219	NA	-11.3666	Significant
SLC9B2	chr4	103997179-103997187	ACGCCAACA	2.3219	0.0452	-28.6489	Significant
GALNTL6	chr4	173605376-173605384	TGATGGCGT	2.3219	-0.0446	-11.6339	Significant
Intergenic	chr6	68324735-68324743	TGATGGCGA	2.3219	NA	-13.2983	Significant
Intergenic	chr9	38090205-38090213	ACGCCAACA	2.3219	NA	-11.3666	Significant
PALM2	chr9	112478559-112478567	TGAAGGCGT	2.3219	0.1024	-9.5750	Significant
Intergenic	chr12	80776534-80776542	TGTAGGCGT	2.3219	NA	-8.8501	Significant
MTUS2	chr13	29969471-29969479	TGTTGGCGA	2.3219	-0.1433	-9.5750	Significant
Intergenic	chr14	31971191-31971199	TGAAGGCGT	2.3219	NA	-11.9042	Significant
Intergenic	chr14	54619096-54619104	TGTTGGCGT	2.3219	NA	-15.3701	Significant
Intergenic	chr14	90825072-90825080	ACGCCATCA	2.3219	NA	-6.6195	Significant

Intergenic	chrX 120657200-120657208	TGTTGGCGA	2.3219	NA	-10.3257	Significant
Intergenic	chr5 151358576-151358584	TGATGGCGT	2.2801	NA	-18.9089	Significant
Intergenic	chr12 18046005-18046013	TGTTGGCGA	2.2730	NA	-10.0726	Significant
Intergenic	chr12 19695500-19695508	TGTTGGCGT	2.2730	NA	-13.2983	Significant
Intergenic	chr6 94981478-94981486	TGTTGGCGA	2.2630	NA	-9.5750	Significant
Intergenic	chr7 84172385-84172393	ACGCCAACA	2.2630	NA	-6.6195	Significant
DACH1	chr13 72410819-72410827	TCGCCTTCA	2.2630	-0.8427	-8.1509	Significant
Intergenic	chr13 78854951-78854959	ACGCCTTCA	2.2630	NA	-10.3257	Significant
Intergenic	chr4 118099410-118099418	TGTTGGCGT	2.2479	NA	-10.5816	Significant
Intergenic	chr4 171593132-171593140	TGATGGCGA	2.2479	NA	-15.6775	Significant
Intergenic	chr6 113101966-113101974	ACGCCTTCA	2.2479	NA	-9.5750	Significant
VCPIP1	chr8 67554321-67554329	TCGCCTACA	2.2479	-2.1514	-17.2575	Significant
Intergenic	chr10 55027786-55027794	TGTAGGCGT	2.2479	NA	-8.1509	Significant
Intergenic	chr14 25067242-25067250	TCGCCTTCA	2.2479	NA	-10.8404	Significant
Intergenic	chr14 27181440-27181448	TCGCCATCA	2.2479	NA	-19.5895	Significant
Intergenic	chrX 20836377-20836385	TGTTGGCGT	2.2479	NA	-11.3666	Significant
HTR7	chr10 92569299-92569307	TCGCCTTCA	2.2303	-1.6229	-11.3666	Significant
INADL	chr1 62415937-62415945	TGATGGCGT	2.2224	0.4141	-12.7321	Significant
Intergenic	chr2 105456230-105456238	ACGCCATCA	2.2224	NA	-18.5729	Significant
Intergenic	chr2 209074866-209074874	TGTTGGCGT	2.2224	NA	-19.5895	Significant
Intergenic	chr10 132398260-132398268	TGTAGGCGT	2.2224	NA	-8.3811	Significant
Intergenic	chr13 35496339-35496347	TGTTGGCGA	2.2224	NA	-8.3811	Significant
RERE	chr1 8693850-8693858	TGATGGCGT	2.2130	-0.8813	-26.6257	Significant
Intergenic	chr3 144113739-144113747	TGAAGGCGA	2.2016	NA	-15.0655	Significant
Intergenic	chr5 89659942-89659950	TGTTGGCGA	2.2016	NA	-18.2397	Significant
Intergenic	chr6 88898822-88898830	ACGCCTACA	2.2016	NA	-7.9235	Significant
Intergenic	chrX 102255146-102255154	TCGCCAACA	2.2016	NA	-15.6775	Significant
IQCA1	chr2 237332011-237332019	ACGCCATCA	2.1927	-0.0715	-20.9850	Significant
Intergenic	chr1 247767279-247767287	ACGCCAACA	2.1699	NA	-7.0428	Significant
MAN1A1	chr6 119579091-119579099	TCGCCAACA	2.1699	0.2512	-11.1021	Significant
Intergenic	chr8 120167188-120167196	TGTAGGCGT	2.1699	NA	-9.8224	Significant
Intergenic	chr12 46052350-46052358	TGATGGCGT	2.1699	NA	-10.0726	Significant
ATXN2	chr12 112017900-112017908	ACGCCATCA	2.1699	-1.1047	-16.6169	Significant
Intergenic	chr16 76104838-76104846	TGTTGGCGA	2.1699	NA	-9.3305	Significant
Intergenic	chr5 10102281-10102289	ACGCCTTCA	2.1468	NA	-6.4122	Significant
PPIP5K2	chr5 102507918-102507926	TCGCCTACA	2.1468	0.3971	-10.8404	Significant
CACNB2	chr10 18805205-18805213	TGATGGCGT	2.1468	0.1071	-13.2983	Significant
DLG2	chr11 83544609-83544617	TGATGGCGT	2.1468	0.2531	-25.0586	Significant
C20orf196	chr20 5750919-5750927	TGTAGGCGA	2.1468	-2.6269	-12.7321	Significant
Intergenic	chr2 129673388-129673396	TCGCCAACA	2.1375	NA	-9.3305	Significant
Intergenic	chr2 150967748-150967756	TCGCCTTCA	2.1375	NA	-19.9341	Significant
Intergenic	chr8 129782650-129782658	TGTTGGCGA	2.1375	NA	-17.9095	Significant
Intergenic	chr9 120527843-120527851	ACGCCATCA	2.1293	NA	-6.4122	Significant
Promoter_TRNAU1AP	chr1 28879115-28879123	TGTTGGCGA	2.1155	-0.4174	-12.4532	Significant
ABCD3	chr1 94928395-94928403	TGATGGCGA	2.1155	0.3152	-9.3305	Significant
COL11A1	chr1 103381185-103381193	ACGCCTACA	2.1155	0.1321	-12.4532	Significant
Intergenic	chr5 96387625-96387633	TGTTGGCGA	2.1155	NA	-11.9042	Significant
MLIP	chr6 54110867-54110875	TCGCCTTCA	2.1155	0.1397	-15.6775	Significant
Intergenic	chr11 15806354-15806362	ACGCCAACA	2.1155	NA	-13.8759	Significant
GYS2	chr12 21718777-21718785	ACGCCAACA	2.1155	-0.1713	-23.9133	Significant
NUP160	chr11 47852577-47852585	TGATGGCGT	2.1069	-1.5604	-11.3666	Significant
BIRC6	chr2 32723314-32723322	TGTAGGCGT	2.0875	-0.5994	-8.6142	Significant
Intergenic	chr5 151358373-151358381	ACGCCAACA	2.0875	NA	-18.2397	Significant
Intergenic	chr6 122334903-122334911	TGTTGGCGT	2.0875	NA	-12.4532	Significant
Intergenic	chr12 78677052-78677060	TGAAGGCGA	2.0875	NA	-10.0726	Significant
Intergenic	chr14 38218541-38218549	TGTAGGCGT	2.0875	NA	-9.0889	Significant
Intergenic	chrX 66019877-66019885	TCGCCAACA	2.0875	NA	-10.3257	Significant
BRE	chr2 28115419-28115427	TGTTGGCGA	2.0780	0.2012	-10.3257	Significant
IL23R	chr1 67671120-67671128	ACGCCTACA	2.0704	0.0316	-8.3811	Significant
Intergenic	chr10 65423881-65423889	TGTTGGCGA	2.0704	NA	-12.7321	Significant
GFRA1	chr10 117964895-117964903	TCGCCAACA	2.0704	0.0985	-10.0726	Significant
Intergenic	chr2 180967841-180967849	TGATGGCGT	2.0589	NA	-6.0062	Significant
Intergenic	chr11 107633680-107633688	ACGCCTACA	2.0589	NA	-12.7321	Significant
MDGA2	chr14 48069407-48069415	TCGCCAACA	2.0589	0.0187	-10.3257	Significant
Intergenic	chr16 59874569-59874577	ACGCCTACA	2.0589	NA	-11.6339	Significant
Intergenic	chr2 201063518-201063526	TGATGGCGT	2.0506	NA	-16.6169	Significant
AK3	chr9 4713902-4713910	ACGCCTACA	2.0506	0.6151	-15.3701	Significant
AK3	chr9 4713980-4713988	ACGCCTACA	2.0506	0.6151	-11.6339	Significant
AK3	chr9 4714022-4714030	ACGCCTACA	2.0506	0.6151	-10.0726	Significant
AK3	chr9 4714036-4714044	ACGCCTACA	2.0506	0.6151	-10.3257	Significant
Intergenic	chr2 49750744-49750752	TGTTGGCGA	2.0444	NA	-12.1773	Significant
KCNAB1	chr3 155860811-155860819	TCGCCTACA	2.0444	0.2195	-12.7321	Significant

CDC25C	chr5	137653881-137653889	TGATGGCGT	2.0356	-0.4924	-11.9042	Significant
Intergenic	chr5	175329499-175329507	TGTTGGCGT	2.0356	NA	-17.5820	Significant
GNAL	chr18	11858131-11858139	ACGCCAACA	2.0298	-0.1255	-9.3305	Significant
Intergenic	chr1	24248095-24248103	TGTTGGCGT	2.0000	NA	-7.4774	Significant
Intergenic	chr1	194861514-194861522	TGTTGGCGA	2.0000	NA	-9.3305	Significant
Intergenic	chr3	190177105-190177113	TGTTGGCGA	2.0000	NA	-9.5750	Significant
Intergenic	chr4	33459788-33459796	TGATGGCGT	2.0000	NA	-18.2397	Significant
Intergenic	chr6	98341290-98341298	TCGCCTTCA	2.0000	NA	-11.3666	Significant
Intergenic	chr6	123470326-123470334	TGTTGGCGT	2.0000	NA	-18.9089	Significant
Intergenic	chr7	145636404-145636412	ACGCCTACA	2.0000	NA	-8.8501	Significant
MTUS1	chr8	17563684-17563692	TCGCCATCA	2.0000	-0.1769	-10.5816	Significant
LINC00476	chr9	98630628-98630636	ACGCCTTCA	2.0000	-0.0214	-8.6142	Significant
YME1L1	chr10	27436920-27436928	TCGCCAACA	2.0000	0.4698	-7.9235	Significant
RNLS	chr10	90034735-90034743	TCGCCATCA	2.0000	0.1611	-13.5857	Significant
Intergenic	chr12	56886554-56886562	TGTAGGCGA	2.0000	NA	-8.3811	Significant
Intergenic	chr12	57820812-57820820	ACGCCTACA	2.0000	NA	-11.6339	Significant
ARGLU1	chr13	107204139-107204147	ACGCCAACA	2.0000	0.5308	-7.0428	Significant
Intergenic	chr14	34723282-34723290	TCGCCATCA	2.0000	NA	-10.5816	Significant
Intergenic	chr16	26629580-26629588	TCGCCTACA	2.0000	NA	-9.3305	Significant
Intergenic	chr16	74234958-74234966	TCGCCTTCA	2.0000	NA	-11.1021	Significant
CDH13	chr16	82785584-82785592	ACGCCAACA	2.0000	0.4432	-7.6991	Significant
Intergenic	chr17	16516472-16516480	TCGCCAACA	2.0000	NA	-11.3666	Significant
SSH2	chr17	28230866-28230874	TGAAGGCGA	2.0000	-1.1490	-7.4774	Significant
Intergenic	chrX	53196437-53196445	TCGCCTACA	2.0000	NA	-9.3305	Significant
Intergenic	chr1	238788399-238788407	ACGCCTACA	1.9542	NA	-13.2983	Significant
Intergenic	chr6	120702663-120702671	TGATGGCGT	1.9475	NA	-25.0586	Significant
CYTH3	chr7	6310284-6310292	TGTTGGCGT	1.9434	-1.8503	-8.8501	Significant
Intergenic	chr1	239345489-239345497	TGTTGGCGA	1.9386	NA	-14.7638	Significant
Intergenic	chr3	174295834-174295842	TGAAGGCGA	1.9386	NA	-10.0726	Significant
CLDN10	chr13	96173043-96173051	TCGCCAACA	1.9386	-0.0925	-21.6999	Significant
GRM1	chr6	146459097-146459105	TGTTGGCGT	1.9260	0.0016	-11.1021	Significant
Intergenic	chr12	48327780-48327788	TGTTGGCGA	1.9260	NA	-6.8297	Significant
BTBD11	chr12	107810822-107810830	TGAAGGCGA	1.9260	-0.7511	-10.0726	Significant
DCC	chr18	50474414-50474422	TGTTGGCGT	1.9260	-0.0278	-17.5820	Significant
Intergenic	chrX	35463649-35463657	ACGCCAACA	1.9260	NA	-10.3257	Significant
RAB10	chr2	26294870-26294878	ACGCCAACA	1.9069	0.3930	-13.0137	Significant
LPP	chr3	188547904-188547912	TGATGGCGT	1.9069	0.2581	-22.7937	Significant
GPR98	chr5	89979476-89979484	TCGCCTTCA	1.9069	0.0225	-11.6339	Significant
Intergenic	chr7	16991700-16991708	TGATGGCGT	1.9069	NA	-5.6115	Significant
KIAA1217	chr10	24053238-24053246	TGTTGGCGA	1.9069	-0.5115	-7.6991	Significant
Intergenic	chr11	24198569-24198577	TCGCCTTCA	1.9069	NA	-10.3257	Significant
Intergenic	chr13	42476854-42476862	TCGCCTTCA	1.9069	NA	-13.8759	Significant
Intergenic	chr15	80550910-80550918	TCGCCTTCA	1.9069	NA	-12.1773	Significant
AKAP8L	chr19	15503516-15503524	TCGCCAACA	1.9069	-0.3536	-10.8404	Significant
Intergenic	chrX	115159710-115159718	TGTTGGCGT	1.9069	NA	-21.6999	Significant
ARHGAP8	chr22	45158928-45158936	ACGCCTACA	1.8931	-0.6163	-13.8759	Significant
PRR5-ARHGAP8	chr22	45158928-45158936	ACGCCTACA	1.8931	-0.6715	-13.8759	Significant
TDRD10	chr1	154476219-154476227	TGATGGCGA	1.8745	0.0918	-19.5895	Significant
ZNF385B	chr2	180331822-180331830	TGTTGGCGA	1.8745	0.1151	-13.2983	Significant
ANKMY1	chr2	241472269-241472277	ACGCCATCA	1.8745	-0.8352	-10.3257	Significant
Intergenic	chr5	41544173-41544181	TGTTGGCGA	1.8745	NA	-41.7930	Significant
Intergenic	chr5	126806187-126806195	TGATGGCGT	1.8745	NA	-6.2078	Significant
Intergenic	chr8	117062578-117062586	TGATGGCGT	1.8745	NA	-10.0726	Significant
ITGB1	chr10	33208438-33208446	TCGCCATCA	1.8745	0.2764	-23.1641	Significant
CTNNA3	chr10	68939639-68939647	ACGCCTTCA	1.8745	0.0490	-13.2983	Significant
Intergenic	chr15	97722182-97722190	TCGCCTTCA	1.8745	NA	-22.4263	Significant
Intergenic	chr22	34509470-34509478	TCGCCTTCA	1.8745	NA	-5.8074	Significant
SLC16A4	chr1	110924478-110924486	TGATGGCGA	1.8580	0.2283	-14.1690	Significant
Intergenic	chr11	114155691-114155699	TGTTGGCGA	1.8580	NA	-13.0137	Significant
Intergenic	chr3	126279104-126279112	TGTTGGCGT	1.8480	NA	-6.0062	Significant
AHI1	chr6	135619714-135619722	TCGCCTACA	1.8480	0.3613	-10.3257	Significant
Intergenic	chr7	68586093-68586101	TGTAGGCGT	1.8480	NA	-15.3701	Significant
Intergenic	chr7	125536333-125536341	TGTAGGCGT	1.8480	NA	-11.3666	Significant
Intergenic	chr8	66331871-66331879	ACGCCAACA	1.8480	NA	-9.8224	Significant
RFX3	chr9	3431501-3431509	ACGCCAACA	1.8480	-0.1848	-10.8404	Significant
PCDH15	chr10	55734495-55734503	TGATGGCGA	1.8480	-0.0158	-10.0726	Significant
NUP98	chr11	3805037-3805045	ACGCCTACA	1.8480	-0.4525	-7.6991	Significant
Intergenic	chrX	22537488-22537496	TGTTGGCGT	1.8480	NA	-9.3305	Significant
Intergenic	chrX	40936645-40936653	TGAAGGCGA	1.8480	NA	-9.8224	Significant
Intergenic	chr2	32014633-32014641	ACGCCAACA	1.8365	NA	-8.1509	Significant
Intergenic	chr2	84268919-84268927	TCGCCAACA	1.8365	NA	-14.1690	Significant
Intergenic	chr2	140761940-140761948	ACGCCTACA	1.8365	NA	-15.9878	Significant

ADAM23	chr2	207343263-207343271	TGATGGCGT	1.8365	0.2165	-15.6775	Significant
Intergenic	chr3	187400920-187400928	TGAAGGCGT	1.8365	NA	-15.3701	Significant
CAV1	chr7	116184097-116184105	TGAAGGCGT	1.8365	0.3684	-7.2587	Significant
IMMP1L	chr11	31479548-31479556	ACGCCATCA	1.8365	-0.0972	-14.4650	Significant
ME3	chr11	86222324-86222332	ACGCCTACA	1.8365	0.4500	-8.6142	Significant
FARP1	chr13	98951720-98951728	ACGCCTTCA	1.8301	0.3376	-9.3305	Significant
DPYD	chr1	97963240-97963248	TGTTGGCGA	1.8074	0.1842	-7.9235	Significant
Intergenic	chr1	191017586-191017594	ACGCCAACA	1.8074	NA	-6.8297	Significant
PSMD14	chr2	162240717-162240726	TGTAGGCGA	1.8074	0.2890	-7.4774	Significant
Intergenic	chr5	163505250-163505258	TGATGGCGA	1.8074	NA	-7.4774	Significant
PKHD1	chr6	51901112-51901120	TCGCCATCA	1.8074	0.1929	-10.8404	Significant
WRN	chr8	31022319-31022327	TGAAGGCGT	1.8074	-0.1060	-23.5373	Significant
TRPM3	chr9	73432961-73432969	TGTTGGCGT	1.8074	0.0215	-10.3257	Significant
Intergenic	chr9	107349923-107349931	ACGCCTACA	1.8074	NA	-14.7638	Significant
Intergenic	chr12	9893072-9893080	TGTAGGCGT	1.8074	NA	-10.5816	Significant
Intergenic	chr13	30557501-30557509	TGTAGGCGT	1.8074	NA	-18.9089	Significant
Intergenic	chr13	80145415-80145423	TGTAGGCGT	1.8074	NA	-10.0726	Significant
SCFD1	chr14	31131544-31131552	TGTAGGCGA	1.8074	0.3197	-6.6195	Significant
SHC4	chr15	49121082-49121090	TGTAGGCGT	1.8074	-0.1634	-12.4532	Significant
Intergenic	chr15	88286126-88286134	ACGCCTACA	1.8074	NA	-11.9042	Significant
MMP28	chr17	34104789-34104797	ACGCCTACA	1.8074	-0.3932	-25.0586	Significant
Intergenic	chr18	6814131-6814139	ACGCCTTCA	1.8074	NA	-9.8224	Significant
Intergenic	chr21	23061391-23061399	TCGCCATCA	1.8074	NA	-9.8224	Significant
Intergenic	chrX	44224446-44224454	TGATGGCGA	1.8074	NA	-11.3666	Significant
Intergenic	chr9	86273771-86273779	ACGCCTTCA	1.7843	NA	-7.6991	Significant
Promoter_FBXL12	chr19	9930446-9930454	TGAAGGCGT	1.7843	-0.8043	-11.3666	Significant
Intergenic	chr1	158101391-158101399	ACGCCTTCA	1.7776	NA	-7.2587	Significant
PDE4D	chr5	59746757-59746765	TCGCCTACA	1.7776	-2.4462	-12.1773	Significant
Intergenic	chr17	8290870-8290878	ACGCCATCA	1.7776	NA	-9.8224	Significant
Intergenic	chr2	181965996-181966004	TGATGGCGA	1.7655	NA	-14.4650	Significant
PPARG	chr3	12432946-12432954	TGAAGGCGA	1.7655	-0.2439	-18.5729	Significant
Intergenic	chr3	61431302-61431310	TGTAGGCGT	1.7655	NA	-11.6339	Significant
Intergenic	chr3	106756152-106756160	ACGCCAACA	1.7655	NA	-18.2397	Significant
SOX2-OT	chr3	181086593-181086601	TGAAGGCGA	1.7655	0.1013	-11.9042	Significant
GALNTL6	chr4	173706825-173706833	TGATGGCGA	1.7655	-0.0446	-20.2815	Significant
Intergenic	chr5	98683380-98683388	TGTTGGCGA	1.7655	NA	-12.4532	Significant
EYS	chr6	64859756-64859764	ACGCCTTCA	1.7655	0.2401	-11.6339	Significant
RSBN1L	chr7	77369282-77369290	TGATGGCGA	1.7655	-3.0508	-16.6169	Significant
HAS2	chr8	122648022-122648030	TGAAGGCGT	1.7655	-1.3773	-10.3257	Significant
Intergenic	chr9	129035007-129035015	TGTAGGCGT	1.7655	NA	-9.0889	Significant
Intergenic	chr12	7909761-7909769	TGTAGGCGT	1.7655	NA	-7.4774	Significant
TM9SF2	chr13	100157682-100157690	TGTAGGCGT	1.7655	0.3392	-11.1021	Significant
CACNA2D1	chr7	81599388-81599396	ACGCCTACA	1.7549	0.0695	-9.5750	Significant
PTN	chr7	136925786-136925794	TGAAGGCGA	1.7549	-0.4040	-21.6999	Significant
BICC1	chr10	60587312-60587320	TGATGGCGA	1.7549	-0.1355	-14.4650	Significant
RBFOX1	chr16	7437580-7437588	ACGCCTTCA	1.7549	0.0112	-7.6991	Significant
RBFOX1	chr16	7437818-7437826	TCGCCATCA	1.7549	0.0112	-12.7321	Significant
Intergenic	chr18	13711001-13711009	ACGCCATCA	1.7549	NA	-18.2397	Significant
Intergenic	chr2	67145572-67145580	TGTAGGCGT	1.7500	NA	-10.5816	Significant
Intergenic	chr1	235703670-235703678	TGAAGGCGT	1.7370	NA	-10.5816	Significant
Intergenic	chr2	196960509-196960517	TCGCCAACA	1.7370	NA	-7.9235	Significant
ACOT13	chr6	24697623-24697631	TGTTGGCGT	1.7370	0.3666	-9.0889	Significant
SEL1L	chr14	81979152-81979160	TGATGGCGT	1.7370	-0.5338	-10.8404	Significant
Intergenic	chr20	54933533-54933541	ACGCCAACA	1.7370	NA	-10.8404	Significant
C2orf88	chr2	191004261-191004269	ACGCCTACA	1.7162	-0.1605	-7.2587	Significant
Intergenic	chr5	120247062-120247070	TGATGGCGA	1.7162	NA	-10.8404	Significant
PDSS2	chr6	107609484-107609492	ACGCCTTCA	1.7162	0.3705	-5.0410	Significant
BBS9	chr7	33582722-33582730	ACGCCAACA	1.7162	-0.0368	-8.3811	Significant
ARHGEF9	chrX	62907725-62907733	ACGCCAACA	1.7162	0.1189	-6.0062	Significant
DNM3OS	chr1	172110819-172110827	TGTAGGCGA	1.7004	0.1874	-19.5895	Significant
DNM3	chr1	172110819-172110827	TGTAGGCGA	1.7004	-0.8376	-19.5895	Significant
SMYD3	chr1	246291369-246291377	TGTAGGCGT	1.7004	0.4448	-12.1773	Significant
BRE	chr2	28328498-28328506	TGTTGGCGA	1.7004	0.2012	-12.1773	Significant
Intergenic	chr4	35967647-35967655	TGATGGCGA	1.7004	NA	-16.3009	Significant
Intergenic	chr4	138498459-138498467	ACGCCTACA	1.7004	NA	-15.3701	Significant
Intergenic	chr5	71935527-71935535	TGTTGGCGA	1.7004	NA	-13.8759	Significant
Intergenic	chr6	17379311-17379319	TCGCCATCA	1.7004	NA	-10.8404	Significant
Intergenic	chr6	23744495-23744503	TGTTGGCGA	1.7004	NA	-12.4532	Significant
ELOVL5	chr6	53159743-53159751	ACGCCTTCA	1.7004	-0.0489	-6.6195	Significant
Intergenic	chr8	56969972-56969980	ACGCCATCA	1.7004	NA	-17.5820	Significant
Intergenic	chr10	4241233-4241241	TGTTGGCGT	1.7004	NA	-13.0137	Significant
CELF2	chr10	11074539-11074547	TGATGGCGT	1.7004	0.2191	-11.6339	Significant

Intergenic	chr10	66980146-66980154	TCGCCAACA	1.7004	NA	-15.6775	Significant
ZRANB1	chr10	126639629-126639637	TGTAGGCGT	1.7004	-1.2874	-5.8074	Significant
Promoter_OR4F15	chr15	102357572-102357580	ACGCCTTCA	1.7004	0.1701	-11.6339	Significant
Intergenic	chr17	13261933-13261941	ACGCCTTCA	1.6881	NA	-7.9235	Significant
Intergenic	chr22	42641351-42641359	TGTAGGCGT	1.6881	NA	-6.4122	Significant
Intergenic	chr1	71206974-71206982	TGTTGGCGT	1.6781	NA	-10.5816	Significant
Intergenic	chr3	26631923-26631931	TGATGGCGA	1.6781	NA	-17.5820	Significant
Intergenic	chr5	20610887-20610895	ACGCCAACA	1.6781	NA	-8.8501	Significant
DMRTA1	chr9	22447904-22447912	TCGCCTTCA	1.6781	0.1754	-15.0655	Significant
VTI1A	chr10	114326472-114326480	TGTTGGCGA	1.6781	-0.2900	-7.2587	Significant
OTOGL	chr12	80724230-80724238	TCGCCTACA	1.6781	0.1770	-23.5373	Significant
BCAS3	chr17	59305631-59305639	TGAAGGCGA	1.6781	-0.1306	-10.0726	Significant
ZZZ3	chr1	78095166-78095174	TGATGGCGT	1.6630	-1.6487	-8.6142	Significant
Intergenic	chr2	19346793-19346801	ACGCCTTCA	1.6630	NA	-8.3811	Significant
Intergenic	chr2	37684545-37684553	TCGCCTACA	1.6630	NA	-14.7638	Significant
Intergenic	chr2	72297940-72297948	ACGCCAACA	1.6630	NA	-9.8224	Significant
Intergenic	chr2	108217836-108217844	TCGCCTTCA	1.6630	NA	-10.0726	Significant
ZDHHC11	chr5	807252-807260	ACGCCAACA	1.6630	-0.4928	-8.6142	Significant
SEMA3A	chr7	83637057-83637065	ACGCCTTCA	1.6630	-0.8807	-5.6115	Significant
Intergenic	chr7	89347098-89347106	TCGCCTTCA	1.6630	NA	-6.6195	Significant
Intergenic	chr8	57433530-57433538	TGTAGGCGT	1.6630	NA	-16.9357	Significant
Intergenic	chr11	55284492-55284500	TGTTGGCGT	1.6630	NA	-13.0137	Significant
Intergenic	chr15	46074056-46074064	TGATGGCGA	1.6521	NA	-13.0137	Significant
NCOA1	chr2	24924688-24924696	TCGCCAACA	1.6439	-0.4188	-12.1773	Significant
Intergenic	chr2	124310415-124310423	TCGCCAACA	1.6439	NA	-4.8566	Significant
Intergenic	chr8	43341893-43341901	TGTAGGCGT	1.6439	NA	-6.4122	Significant
SLC4A10	chr2	162696394-162696402	TGTTGGCGT	1.6374	0.2532	-27.0246	Significant
Intergenic	chr3	194728014-194728022	TGTTGGCGT	1.6374	NA	-11.6339	Significant
Intergenic	chr20	4349748-4349756	TCGCCTACA	1.6374	NA	-19.9341	Significant
Intergenic	chr3	149892998-149893006	TGTTGGCGT	1.6323	NA	-10.3257	Significant
Intergenic	chr7	55832504-55832512	TCGCCTACA	1.6323	NA	-6.8297	Significant
TNFRSF9	chr1	7990368-7990376	TGATGGCGA	1.5850	0.0462	-14.7638	Significant
Intergenic	chr1	69026802-69026810	TCGCCAACA	1.5850	NA	-14.4650	Significant
FAM129A	chr1	184916560-184916568	TGTTGGCGA	1.5850	-0.1994	-16.6169	Significant
Intergenic	chr2	36436104-36436112	TGTAGGCGT	1.5850	NA	-8.3811	Significant
Intergenic	chr2	115167482-115167490	TGATGGCGT	1.5850	NA	-7.4774	Significant
Intergenic	chr2	180796520-180796528	TCGCCTACA	1.5850	NA	-8.8501	Significant
FSIP2	chr2	186687977-186687985	ACGCCATCA	1.5850	0.5609	-7.0428	Significant
CADM2	chr3	86039601-86039609	ACGCCATCA	1.5850	0.0611	-6.2078	Significant
Intergenic	chr3	146272510-146272518	ACGCCTTCA	1.5850	NA	-10.0726	Significant
Intergenic	chr3	146295159-146295167	TGAAGGCGA	1.5850	NA	-7.6991	Significant
Intergenic	chr5	8301587-8301595	TCGCCATCA	1.5850	NA	-13.2983	Significant
Intergenic	chr5	24380270-24380278	TGATGGCGT	1.5850	NA	-10.8404	Significant
ARL15	chr5	53365263-53365271	TGATGGCGA	1.5850	0.0527	-10.5816	Significant
MAN2A1	chr5	109131845-109131853	TGTTGGCGA	1.5850	0.2414	-12.1773	Significant
Intergenic	chr5	125374537-125374545	ACGCCAACA	1.5850	NA	-9.3305	Significant
Intergenic	chr5	163537504-163537512	TGTAGGCGA	1.5850	NA	-9.0889	Significant
Intergenic	chr5	165274300-165274308	TCGCCTTCA	1.5850	NA	-13.8759	Significant
RALA	chr7	39711498-39711506	TGATGGCGT	1.5850	-0.4552	-9.8224	Significant
LRGUK	chr7	133899021-133899029	TGTTGGCGA	1.5850	0.0216	-12.1773	Significant
PAXIP1	chr7	154735853-154735861	ACGCCATCA	1.5850	-1.2019	-17.5820	Significant
POTEA	chr8	43214611-43214619	ACGCCAACA	1.5850	0.0776	-11.6339	Significant
BMI1	chr10	22618815-22618823	ACGCCTACA	1.5850	0.3114	-12.7321	Significant
FRMPD2	chr10	49412949-49412957	ACGCCAACA	1.5850	-0.0160	-12.1773	Significant
Intergenic	chr10	114188996-114189004	TCGCCTTCA	1.5850	NA	-14.4650	Significant
SBF2	chr11	10043048-10043056	TGAAGGCGA	1.5850	-0.0601	-9.3305	Significant
COMMD9	chr11	36299409-36299417	TGAAGGCGT	1.5850	0.3252	-9.5750	Significant
Intergenic	chr11	37963591-37963599	TGTAGGCGT	1.5850	NA	-12.1773	Significant
Intergenic	chr11	79406549-79406557	TGATGGCGT	1.5850	NA	-20.2815	Significant
Intergenic	chr11	81266535-81266543	TCGCCAACA	1.5850	NA	-7.9235	Significant
CCDC81	chr11	86129810-86129818	TGAAGGCGT	1.5850	0.1511	-10.5816	Significant
ME3	chr11	86372196-86372204	TGATGGCGA	1.5850	0.4500	-7.0428	Significant
CNTN5	chr11	100033216-100033224	TGAAGGCGT	1.5850	0.0250	-13.5857	Significant
Intergenic	chr11	134703383-134703391	TCGCCATCA	1.5850	NA	-8.6142	Significant
PLEKHA5	chr12	19421610-19421618	TGATGGCGA	1.5850	-1.1084	-6.6195	Significant
GNN	chr12	104317956-104317964	ACGCCTACA	1.5850	0.7163	-14.7638	Significant
Intergenic	chr14	27273853-27273861	ACGCCAACA	1.5850	NA	-14.4650	Significant
Intergenic	chr14	38731521-38731529	ACGCCTTCA	1.5850	NA	-13.2983	Significant
Intergenic	chr14	52714162-52714170	TGTAGGCGT	1.5850	NA	-7.6991	Significant
RTN1	chr14	60096627-60096635	ACGCCATCA	1.5850	0.0283	-8.1509	Significant
Intergenic	chr16	61102030-61102038	TGTAGGCGA	1.5850	NA	-8.3811	Significant
Intergenic	chr16	65731718-65731726	ACGCCAACA	1.5850	NA	-12.1773	Significant

Intergenic	chr22	25745645-25745653	TCGCCATCA	1.5850	NA	-13.5857	Significant
Intergenic	chr22	44875868-44875876	TCGCCATCA	1.5850	NA	-8.6142	Significant
Intergenic	chrX	16066419-16066427	TGAAGGCGA	1.5850	NA	-19.5895	Significant
FBLN7	chr2	112920188-112920196	TCGCCAACA	1.5406	-0.0406	-9.5750	Significant
NEK5	chr13	52660465-52660473	TGTTGGCGT	1.5406	0.0680	-10.5816	Significant
Intergenic	chr3	118529604-118529612	TGAAGGCGA	1.5305	NA	-10.5816	Significant
Intergenic	chr3	162020013-162020021	TCGCCATCA	1.5305	NA	-12.4532	Significant
Intergenic	chr4	1550366-1550374	TGAAGGCGT	1.5305	NA	-12.4532	Significant
Intergenic	chr5	149693210-149693218	TGAAGGCGA	1.5305	NA	-8.8501	Significant
Intergenic	chr17	51497771-51497779	TGTTGGCGT	1.5305	NA	-12.4532	Significant
Intergenic	chr7	85694902-85694910	TCGCCATCA	1.5236	NA	-7.6991	Significant
Intergenic	chr17	52799175-52799183	TCGCCTTCA	1.5236	NA	-17.5820	Significant
LTBP1	chr2	33377488-33377496	TGTTGGCGT	1.5146	0.3106	-11.1021	Significant
Intergenic	chr2	78894229-78894237	ACGCCTACA	1.5146	NA	-7.6991	Significant
PDE1A	chr2	183200184-183200192	ACGCCAACA	1.5146	0.1737	-14.1690	Significant
Intergenic	chr6	82062356-82062364	TCGCCAACA	1.5146	NA	-14.4650	Significant
Intergenic	chr6	169139335-169139343	TGATGGCGA	1.5146	NA	-12.7321	Significant
ZNRF2	chr7	30383739-30383747	TGTTGGCGA	1.5146	-0.7023	-12.4532	Significant
Intergenic	chr7	81138431-81138439	TCGCCAACA	1.5146	NA	-9.3305	Significant
Intergenic	chr8	70315150-70315158	TCGCCTACA	1.5146	NA	-10.3257	Significant
Intergenic	chr9	105339607-105339615	TGATGGCGA	1.5146	NA	-11.1021	Significant
Intergenic	chr10	121932986-121932994	ACGCCTACA	1.5146	NA	-7.6991	Significant
Intergenic	chr12	16935552-16935560	ACGCCATCA	1.5146	NA	-13.0137	Significant
Intergenic	chr14	27517554-27517562	TGTTGGCGA	1.5146	NA	-8.8501	Significant
SYNE2	chr14	64521414-64521422	ACGCCAACA	1.5146	0.3504	-11.6339	Significant
ZNF286B	chr17	18583586-18583594	ACGCCTTCA	1.5146	-1.2427	-8.8501	Significant
Intergenic	chr1	99659412-99659420	TGAAGGCGT	1.5025	NA	-8.6142	Significant
Intergenic	chr2	37426910-37426918	TGTAGGCGT	1.5025	NA	-8.8501	Significant
Intergenic	chr2	102262980-102262988	TGAAGGCGA	1.5025	NA	-5.8074	Significant
ORC4	chr2	148771428-148771436	ACGCCAACA	1.5025	-0.2632	-12.1773	Significant
Intergenic	chr3	64057917-64057925	TCGCCTACA	1.5025	NA	-7.2587	Significant
Intergenic	chr7	120952505-120952513	ACGCCATCA	1.5025	NA	-9.0889	Significant
Intergenic	chr11	98138756-98138764	TCGCCTACA	1.5025	NA	-7.2587	Significant
Intergenic	chr11	123270253-123270261	TCGCCAACA	1.5025	NA	-6.0062	Significant
Intergenic	chr14	83943889-83943897	TGATGGCGT	1.5025	NA	-11.6339	Significant
DOPEY2	chr21	37586790-37586798	ACGCCATCA	1.5025	-0.1824	-7.9235	Significant
YWHAQ	chr2	9768540-9768548	ACGCCATCA	1.4948	1.4954	-6.6195	Significant
Intergenic	chr3	184011762-184011770	TCGCCTTCA	1.4948	NA	-21.6999	Significant
Intergenic	chr1	72958598-72958606	ACGCCAACA	1.4854	NA	-8.6142	Significant
SCN3A	chr2	166020900-166020908	ACGCCATCA	1.4854	0.0228	-10.3257	Significant
ITGA6	chr2	173343881-173343889	ACGCCATCA	1.4854	0.5795	-10.0726	Significant
Intergenic	chr6	56234986-56234994	TCGCCTACA	1.4854	NA	-9.5750	Significant
Intergenic	chr10	25266501-25266509	ACGCCTACA	1.4854	NA	-22.7937	Significant
Intergenic	chr11	98182345-98182353	TCGCCTTCA	1.4854	NA	-9.0889	Significant
MIR4500HG	chr13	88317101-88317109	TGATGGCGA	1.4854	-0.0102	-8.1509	Significant
DIAPH2	chrX	96246261-96246269	ACGCCAACA	1.4854	0.4019	-10.8404	Significant
Intergenic	chr16	52016855-52016863	TCGCCTTCA	1.4739	NA	-7.6991	Significant
RPTOR	chr17	78558525-78558533	ACGCCATCA	1.4739	-1.6122	-10.5816	Significant
Intergenic	chr1	96130699-96130707	TGTTGGCGA	1.4594	NA	-8.1509	Significant
FHIT	chr3	61212903-61212911	ACGCCATCA	1.4594	0.2126	-10.3257	Significant
PRDM9	chr5	23514121-23514129	TCGCCAACA	1.4594	0.4268	-11.9042	Significant
Intergenic	chr5	117190067-117190075	TGTAGGCGT	1.4594	NA	-9.0889	Significant
FBN2	chr5	127681936-127681944	TCGCCTACA	1.4594	0.0292	-13.0137	Significant
Intergenic	chr5	164200275-164200283	TGTAGGCGT	1.4594	NA	-6.0062	Significant
Intergenic	chr6	93561318-93561326	TGTTGGCGT	1.4594	NA	-13.2983	Significant
LAMA2	chr6	129448816-129448824	TCGCCAACA	1.4594	0.1590	-7.9235	Significant
Intergenic	chr7	9729314-9729322	TGTAGGCGT	1.4594	NA	-6.4122	Significant
RGS3	chr9	116316733-116316741	ACGCCATCA	1.4594	-0.9656	-13.0137	Significant
ANK3	chr10	61926372-61926380	TCGCCATCA	1.4594	0.0887	-6.0062	Significant
BBOX1	chr11	27074918-27074926	TCGCCTACA	1.4594	0.0106	-8.3811	Significant
PDZRN4	chr12	41632658-41632666	TGTTGGCGA	1.4594	0.0314	-12.1773	Significant
Intergenic	chr13	80384687-80384695	TGAAGGCGT	1.4594	NA	-6.6195	Significant
GPC6	chr13	94319022-94319030	ACGCCATCA	1.4594	0.1985	-12.1773	Significant
Intergenic	chr14	49723128-49723136	TGTTGGCGA	1.4594	NA	-8.1509	Significant
Intergenic	chr14	98552294-98552302	TCGCCAACA	1.4594	NA	-10.0726	Significant
NETO2	chr16	47170724-47170732	TCGCCTTCA	1.4594	0.1175	-11.3666	Significant
Intergenic	chr16	76034441-76034449	TGATGGCGT	1.4594	NA	-12.7321	Significant
Intergenic	chrX	12909863-12909871	TGAAGGCGA	1.4594	NA	-9.5750	Significant
Intergenic	chr2	206827958-206827966	ACGCCATCA	1.4475	NA	-7.4774	Significant
NUDT5	chr10	12230468-12230476	ACGCCTACA	1.4475	0.3989	-9.8224	Significant
Intergenic	chr5	106276470-106276478	TCGCCTTCA	1.4406	NA	-8.8501	Significant
Intergenic	chr9	36411269-36411277	TGTTGGCGA	1.4406	NA	-9.0889	Significant

Intergenic	chr12	55512635-55512643	TCGCCAACA	1.4406	NA	-11.9042	Significant
Intergenic	chr13	56540071-56540079	TGAAGGCGT	1.4406	NA	-9.8224	Significant
FARP1	chr13	99039417-99039425	TGATGGCGT	1.4406	0.3376	-10.5816	Significant
SPAST	chr2	32298287-32298295	TGATGGCGT	1.4330	-1.5173	-10.8404	Significant
Intergenic	chr3	189288272-189288280	TCGCCATCA	1.4330	NA	-18.2397	Significant
Intergenic	chr1	79077846-79077854	TCGCCATCA	1.4150	NA	-8.1509	Significant
DDR2	chr1	162703650-162703658	TCGCCTACA	1.4150	0.0787	-23.9133	Significant
TIA1	chr2	70452452-70452460	ACGCCAACA	1.4150	-0.2426	-7.9235	Significant
Intergenic	chr2	113611283-113611291	ACGCCATCA	1.4150	NA	-9.0889	Significant
MCTP1	chr5	94082054-94082062	TGAAGGCGT	1.4150	-0.4283	-8.6142	Significant
Intergenic	chr6	117156311-117156319	TGAAGGCGA	1.4150	NA	-7.6991	Significant
Intergenic	chr6	142810834-142810842	TGTTGGCGT	1.4150	NA	-6.4122	Significant
GRM1	chr6	146740210-146740218	TCGCCTACA	1.4150	0.0016	-11.1021	Significant
SNX8	chr7	2336720-2336728	TGTTGGCGT	1.4150	0.0313	-10.0726	Significant
DPY19L2P1	chr7	35125447-35125455	TGATGGCGT	1.4150	0.0105	-10.0726	Significant
DENND2A	chr7	140237741-140237749	TGAAGGCGA	1.4150	0.7503	-9.8224	Significant
TMC1	chr9	75310466-75310474	TCGCCATCA	1.4150	0.2121	-6.2078	Significant
SPATA13	chr13	24623625-24623633	TCGCCTACA	1.4150	-2.1588	-7.6991	Significant
Intergenic	chr13	37699273-37699281	TGTTGGCGA	1.4150	NA	-8.1509	Significant
Intergenic	chr13	49291794-49291802	TCGCCAACA	1.4150	NA	-8.3811	Significant
Intergenic	chr15	61915901-61915909	TCGCCTTCA	1.4150	NA	-12.7321	Significant
FAM154B	chr15	82556342-82556350	ACGCCTTCA	1.4150	0.1941	-13.0137	Significant
SEC11A	chr15	85230518-85230526	TGTAGGCGT	1.4150	0.2806	-8.3811	Significant
NLRC3	chr16	3616163-3616171	TGATGGCGA	1.4150	-0.0956	-7.2587	Significant
CLEC19A	chr16	19316493-19316501	TGAAGGCGA	1.4150	0.2470	-12.1773	Significant
GTF3C1	chr16	27519691-27519699	TGAAGGCGA	1.4150	-0.3765	-12.7321	Significant
Intergenic	chr17	20256150-20256158	TGATGGCGT	1.4150	NA	-11.9042	Significant
Intergenic	chr20	16982569-16982577	TGTAGGCGT	1.4150	NA	-12.7321	Significant
USP25	chr21	17241886-17241894	TCGCCTACA	1.4150	-0.5736	-13.2983	Significant
DMXL1	chr5	118435053-118435061	TCGCCATCA	1.3986	-0.1043	-10.3257	Significant
FNDC3B	chr3	171884149-171884157	TCGCCTTCA	1.3923	-1.7955	-9.0889	Significant
Promoter_ELL2	chr5	95298667-95298675	TGTAGGCGT	1.3923	0.1966	-5.8074	Significant
COL28A1	chr7	7520978-7520986	ACGCCATCA	1.3923	-0.0920	-8.3811	Significant
Intergenic	chr3	110305444-110305452	ACGCCATCA	1.3785	NA	-17.2575	Significant
Intergenic	chr5	32333227-32333235	TCGCCTACA	1.3785	NA	-12.1773	Significant
GFRAL	chr6	55232619-55232627	TGATGGCGT	1.3785	-0.1190	-13.5857	Significant
DYNC1I1	chr7	95716147-95716155	ACGCCAACA	1.3785	-0.1977	-7.2587	Significant
MGAM	chr7	141746648-141746656	TCGCCTTCA	1.3785	0.3288	-10.8404	Significant
TLE1	chr9	84279513-84279521	ACGCCTACA	1.3785	-0.7869	-8.8501	Significant
FAM171A1	chr10	15402812-15402820	ACGCCTTCA	1.3785	-0.5123	-15.6775	Significant
Intergenic	chr12	15208495-15208503	TGAAGGCGT	1.3785	NA	-5.2283	Significant
Intergenic	chr19	51970362-51970370	ACGCCAACA	1.3785	NA	-9.5750	Significant
C2orf43	chr2	21005309-21005317	ACGCCTTCA	1.3626	0.1919	-9.0889	Significant
Intergenic	chr2	23017401-23017409	ACGCCATCA	1.3626	NA	-13.0137	Significant
Intergenic	chr2	64283706-64283714	TGTTGGCGT	1.3626	NA	-13.5857	Significant
Intergenic	chr5	50419056-50419064	ACGCCATCA	1.3626	NA	-12.4532	Significant
Intergenic	chr5	114096166-114096174	TCGCCTACA	1.3626	NA	-9.8224	Significant
Intergenic	chr6	156564361-156564369	TGTTGGCGA	1.3626	NA	-19.2478	Significant
Intergenic	chr7	52358783-52358791	TGTTGGCGT	1.3626	NA	-9.0889	Significant
FOXP2	chr7	113824657-113824665	TCGCCTTCA	1.3626	0.0940	-11.6339	Significant
Intergenic	chr7	154796965-154796973	ACGCCATCA	1.3626	NA	-10.8404	Significant
Intergenic	chr9	80660445-80660453	TGTTGGCGA	1.3626	NA	-14.1690	Significant
Intergenic	chr12	127424941-127424949	TGAAGGCGT	1.3626	NA	-12.4532	Significant
Intergenic	chr13	55987834-55987842	TGATGGCGA	1.3626	NA	-7.4774	Significant
Intergenic	chr13	105920522-105920530	TCGCCAACA	1.3626	NA	-9.3305	Significant
Intergenic	chr16	32423849-32423857	TGATGGCGA	1.3626	NA	-12.4532	Significant
Intergenic	chrX	5220350-5220358	TGTAGGCGA	1.3626	NA	-23.1641	Significant
SNX9	chr6	158335267-158335275	TGATGGCGA	1.3536	-0.2664	-7.0428	Significant
Intergenic	chr9	73908979-73908987	TGTAGGCGA	1.3536	NA	-8.8501	Significant
DGKH	chr13	42776511-42776519	ACGCCTTCA	1.3536	-0.3479	-8.6142	Significant
Intergenic	chr14	21385217-21385225	TCGCCAACA	1.3536	NA	-10.8404	Significant
KIF13A	chr6	17974460-17974468	ACGCCAACA	1.3479	-0.1750	-13.8759	Significant
Intergenic	chr8	49431656-49431664	TCGCCATCA	1.3479	NA	-11.9042	Significant
FAM135B	chr8	139423079-139423087	ACGCCAACA	1.3479	-0.0425	-33.3516	Significant
Intergenic	chr18	10179556-10179564	TGTAGGCGT	1.3479	NA	-12.7321	Significant
CYP24A1	chr20	52773004-52773012	TCGCCTTCA	1.3440	0.2113	-7.9235	Significant
Intergenic	chr1	81055103-81055111	TGATGGCGT	1.3219	NA	-11.6339	Significant
Intergenic	chr1	91955537-91955545	TCGCCAACA	1.3219	NA	-5.6115	Significant
Intergenic	chr1	183524538-183524546	TGTTGGCGA	1.3219	NA	-10.3257	Significant
CTNNA2	chr2	80129577-80129585	TGAAGGCGA	1.3219	0.0868	-10.8404	Significant
Intergenic	chr2	104336774-104336782	ACGCCAACA	1.3219	NA	-6.8297	Significant
NBEAL1	chr2	204022254-204022262	TCGCCATCA	1.3219	-0.2321	-10.8404	Significant

SP140L	chr2	231210831-231210839	ACGCCTTCA	1.3219	0.4075	-10.8404	Significant
Intergenic	chr5	5680387-5680395	TGTTGGCGT	1.3219	NA	-6.6195	Significant
Intergenic	chr5	120697434-120697442	TCGCCTTCA	1.3219	NA	-15.3701	Significant
Intergenic	chr5	124518445-124518453	TGTTGGCGA	1.3219	NA	-6.4122	Significant
MEGF10	chr5	126721166-126721174	TGTTGGCGA	1.3219	-0.0163	-7.2587	Significant
RAPGEF6	chr5	130932914-130932922	TGTTGGCGA	1.3219	-0.6865	-5.0410	Significant
ACOT13	chr6	24697797-24697805	TGTTGGCGT	1.3219	0.3666	-5.0410	Significant
GABRR2	chr6	89998635-89998643	TGTAGGCGT	1.3219	-0.1860	-9.5750	Significant
Intergenic	chr7	17608155-17608163	TCGCCTTCA	1.3219	NA	-8.1509	Significant
Intergenic	chr7	40295377-40295385	TCGCCATCA	1.3219	NA	-11.6339	Significant
Intergenic	chr7	85822738-85822746	TGATGGCGA	1.3219	NA	-6.2078	Significant
Intergenic	chr8	82132417-82132425	ACGCCAACA	1.3219	NA	-5.4185	Significant
Intergenic	chr8	142939552-142939560	TCGCCATCA	1.3219	NA	-6.8297	Significant
Intergenic	chr11	32093189-32093197	TGTTGGCGT	1.3219	NA	-8.8501	Significant
CD44	chr11	35201976-35201984	TGTTGGCGT	1.3219	0.3519	-9.0889	Significant
Intergenic	chr12	10548834-10548842	ACGCCTACA	1.3219	NA	-8.6142	Significant
ST8SIA1	chr12	22370317-22370325	TGATGGCGT	1.3219	-0.2700	-5.6115	Significant
IKBIP	chr12	99029440-99029448	TGTAGGCGA	1.3219	0.1171	-9.5750	Significant
Intergenic	chr14	38550850-38550858	TGTTGGCGA	1.3219	NA	-5.8074	Significant
Intergenic	chr14	99125279-99125287	TGATGGCGT	1.3219	NA	-12.7321	Significant
UNC13C	chr15	54882798-54882806	TGTTGGCGT	1.3219	0.0971	-10.0726	Significant
Intergenic	chr17	6861612-6861620	ACGCCAACA	1.3219	NA	-7.0428	Significant
TTI1	chr20	36636544-36636552	TGATGGCGT	1.3219	-1.0348	-6.8297	Significant
DRP2	chrX	100483994-100484002	TGATGGCGA	1.3219	-0.2891	-16.3009	Significant
Intergenic	chr5	3685489-3685497	ACGCCATCA	1.2955	NA	-9.5750	Significant
Intergenic	chr3	81010856-81010864	TGTAGGCGT	1.2895	NA	-11.3666	Significant
AZIN1	chr8	103874339-103874347	TGATGGCGA	1.2895	0.1021	-6.2078	Significant
Intergenic	chr15	40808235-40808243	TGTTGGCGA	1.2895	NA	-11.3666	Significant
Intergenic	chr20	50494565-50494573	TGATGGCGA	1.2895	NA	-10.0726	Significant
RAB1A	chr2	65317038-65317046	TGTTGGCGA	1.2801	0.3344	-9.3305	Significant
Intergenic	chr2	184248930-184248938	TGTTGGCGT	1.2801	NA	-9.3305	Significant
Intergenic	chr2	188942125-188942133	TCGCCAACA	1.2801	NA	-6.4122	Significant
Intergenic	chr5	106033038-106033046	TCGCCTACA	1.2801	NA	-10.5816	Significant
Intergenic	chr6	147054959-147054967	TGAAGGCGT	1.2801	NA	-7.2587	Significant
GTF2I	chr7	74121328-74121336	ACGCCATCA	1.2801	-0.3730	-10.0726	Significant
ATP6V1B2	chr8	20077387-20077395	TCGCCTTCA	1.2801	-0.3556	-13.0137	Significant
ZFAT	chr8	135564844-135564852	TGATGGCGT	1.2801	-1.1029	-6.4122	Significant
LIN52	chr14	74559428-74559436	TCGCCAACA	1.2801	-1.4663	-12.1773	Significant
Intergenic	chr17	47365280-47365288	TGATGGCGT	1.2801	NA	-7.2587	Significant
Intergenic	chr17	52914754-52914762	TGTTGGCGT	1.2801	NA	-8.3811	Significant
MED13	chr17	60082775-60082783	TCGCCAACA	1.2801	-1.9008	-12.4532	Significant
ZNF146	chr19	36722728-36722736	TGTAGGCGA	1.2801	-1.4208	-9.0889	Significant
Intergenic	chr21	31552095-31552103	ACGCCTTCA	1.2801	NA	-12.1773	Significant
Intergenic	chr2	35052899-35052907	TGATGGCGT	1.2630	NA	-9.3305	Significant
SRBD1	chr2	45813830-45813838	ACGCCATCA	1.2630	-0.0112	-12.1773	Significant
FGF5	chr4	81191810-81191818	ACGCCTTCA	1.2630	-0.0773	-15.9878	Significant
SDK1	chr7	3838668-3838676	TCGCCTTCA	1.2630	-0.0218	-6.2078	Significant
Intergenic	chr7	21103726-21103734	ACGCCAACA	1.2630	NA	-10.8404	Significant
Intergenic	chr7	51898374-51898382	TGAAGGCGA	1.2630	NA	-9.0889	Significant
Intergenic	chr12	28248593-28248601	TCGCCATCA	1.2630	NA	-11.9042	Significant
ZMYM2	chr13	20594628-20594636	TGTTGGCGT	1.2630	-0.8828	-7.6991	Significant
PCDH9	chr13	67178882-67178890	ACGCCATCA	1.2630	0.0723	-13.2983	Significant
Intergenic	chr13	88367944-88367952	TCGCCATCA	1.2630	NA	-6.6195	Significant
Intergenic	chr14	46902868-46902876	ACGCCTACA	1.2630	NA	-10.0726	Significant
Intergenic	chr19	34519444-34519452	ACGCCATCA	1.2630	NA	-8.8501	Significant
Intergenic	chrX	124186649-124186657	TCGCCAACA	1.2630	NA	-6.4122	Significant
METAP1D	chr2	172885038-172885046	ACGCCATCA	1.2479	-0.7302	-10.3257	Significant
NXPH1	chr7	8679475-8679483	TCGCCTTCA	1.2479	-0.0158	-7.0428	Significant
LOC493754	chr7	66050974-66050982	ACGCCATCA	1.2479	NA	-7.0428	Significant
PRKCH	chr14	61885629-61885637	TGATGGCGA	1.2479	-0.4984	-9.0889	Significant
TM2D3	chr15	102189153-102189161	TCGCCTACA	1.2479	-0.6008	-7.6991	Significant
SOCS7	chr17	36548403-36548411	TGTTGGCGT	1.2479	-0.9360	-12.7321	Significant
Intergenic	chr2	5967726-5967734	TCGCCATCA	1.2410	NA	-8.1509	Significant
Intergenic	chr7	77269684-77269692	TGTTGGCGA	1.2410	NA	-15.9878	Significant
EIF4G3	chr1	21322812-21322820	TGATGGCGA	1.2224	0.4140	-12.4532	Significant
PPIEL	chr1	40000306-40000314	ACGCCATCA	1.2224	0.0148	-8.3811	Significant
NTNG1	chr1	107991381-107991389	ACGCCATCA	1.2224	0.1868	-4.3204	Marginal
RGL1	chr1	183883986-183883994	TGAAGGCGA	1.2224	-3.0584	-11.3666	Significant
PLA2G4A	chr1	186861806-186861814	TGTTGGCGT	1.2224	0.0552	-9.0889	Significant
Intergenic	chr2	188721944-188721952	TGTTGGCGT	1.2224	NA	-10.8404	Significant
LOC100506085	chr4	170876923-170876931	TGTTGGCGA	1.2224	0.1199	-11.6339	Significant
LOC339975	chr4	188236948-188236956	TGTTGGCGT	1.2224	-0.0447	-7.6991	Significant

Intergenic	chr5	148276504-148276512	TGTTGGCGT	1.2224	NA	-7.2587	Significant
RANBP9	chr6	13655566-13655574	TGATGGCGT	1.2224	-0.5946	-9.5750	Significant
Intergenic	chr7	152575944-152575952	TGTAGGCGT	1.2224	NA	-8.3811	Significant
HERC4	chr10	69791157-69791165	TCGCCAACA	1.2224	-0.1803	-18.2397	Significant
Intergenic	chr11	48000297-48000305	ACGCCATCA	1.2224	NA	-7.9235	Significant
DENND5B	chr12	31648850-31648858	TCGCCTACA	1.2224	-1.6568	-7.0428	Significant
KPNA3	chr13	50344631-50344639	TGATGGCGT	1.2224	-0.1523	-9.8224	Significant
Intergenic	chr13	105950100-105950108	TGATGGCGA	1.2224	NA	-14.7638	Significant
Intergenic	chr14	102073753-102073761	ACGCCATCA	1.2224	NA	-10.8404	Significant
SYN3	chr22	33033826-33033834	TGTTGGCGA	1.2224	-0.0804	-10.5816	Significant
Intergenic	chr22	49166707-49166715	TCGCCTTCA	1.2224	NA	-9.0889	Significant
Intergenic	chrX	5805118-5805126	TCGCCATCA	1.2224	NA	-14.7638	Significant
PCMTD2	chr20	62904511-62904519	TGTAGGCGT	1.2065	0.2551	-6.0062	Significant
SNX2	chr5	122113992-122114000	TCGCCAACA	1.2016	0.2730	-10.8404	Significant
Intergenic	chr6	81229710-81229718	ACGCCATCA	1.2016	NA	-6.6195	Significant
Intergenic	chr7	14891507-14891515	ACGCCTTCA	1.2016	NA	-7.0428	Significant
Intergenic	chr7	65472200-65472208	TGTAGGCGT	1.2016	NA	-7.9235	Significant
Intergenic	chr8	20369494-20369502	TGTAGGCGA	1.2016	NA	-10.8404	Significant
UNC5D	chr8	35151233-35151241	TGATGGCGT	1.2016	-0.1505	-6.8297	Significant
GLB1L2	chr11	134222123-134222131	TGTTGGCGT	1.2016	0.3868	-9.0889	Significant
Intergenic	chr13	32019811-32019819	ACGCCAACA	1.2016	NA	-8.3811	Significant
FREM2	chr13	39388141-39388149	TCGCCTACA	1.2016	0.1013	-7.0428	Significant
LOC339260	chr17	20879858-20879866	ACGCCAACA	1.2016	NA	-19.9341	Significant
Intergenic	chr1	165892480-165892488	TGAAGGCGA	1.1927	NA	-7.0428	Significant
Intergenic	chr2	34757485-34757493	TGATGGCGT	1.1927	NA	-16.3009	Significant
KIAA2018	chr3	113393693-113393701	TGTAGGCGT	1.1927	-1.5973	-9.3305	Significant
NLGN1	chr3	173248408-173248416	TGTTGGCGT	1.1927	-0.3248	-7.6991	Significant
Intergenic	chr6	137882483-137882491	TCGCCTTCA	1.1927	NA	-11.6339	Significant
TRIM35	chr8	27142645-27142653	TCGCCATCA	1.1927	-1.8044	-7.0428	Significant
Intergenic	chr9	94888052-94888060	TCGCCATCA	1.1927	NA	-13.0137	Significant
NELL1	chr11	20716118-20716126	TGTAGGCGT	1.1927	-0.0272	-7.4774	Significant
Intergenic	chr11	49471323-49471331	TGAAGGCGA	1.1927	NA	-10.0726	Significant
CNTN5	chr11	99828412-99828420	TGAAGGCGT	1.1927	0.0250	-6.8297	Significant
Intergenic	chr12	22678999-22679007	ACGCCATCA	1.1927	NA	-9.0889	Significant
Intergenic	chr15	73830174-73830182	TCGCCTTCA	1.1927	NA	-6.0062	Significant
Intergenic	chr15	93840279-93840287	ACGCCAACA	1.1927	NA	-23.5373	Significant
ZNF221	chr19	44470783-44470791	TGTAGGCGA	1.1927	0.1232	-8.8501	Significant
PPIEL	chr1	40015911-40015919	TGAAGGCGT	1.1844	0.0148	-16.9357	Significant
CMAS	chr12	22208507-22208515	ACGCCATCA	1.1806	0.0195	-10.0726	Significant
KIT	chr4	55585681-55585689	TGTTGGCGA	-1.1699	-0.0873	-20.6318	Reference
Intergenic	chr3	125392837-125392845	TGTTGGCGT	1.1699	NA	-9.8224	Significant
Intergenic	chr5	95560749-95560757	TGATGGCGT	1.1699	NA	-8.8501	Significant
FBXL17	chr5	107605978-107605986	TGAAGGCGA	1.1699	0.1326	-15.9878	Significant
Intergenic	chr5	165824589-165824597	TGAAGGCGT	1.1699	NA	-5.4185	Significant
PDSS2	chr6	107641261-107641269	TCGCCTTCA	1.1699	0.3705	-10.3257	Significant
RPS6KA2	chr6	166829703-166829711	TCGCCATCA	1.1699	-0.3066	-10.0726	Significant
POU6F2	chr7	39487802-39487810	TGAAGGCGA	1.1699	0.0475	-8.6142	Significant
Intergenic	chr7	77412496-77412504	ACGCCAACA	1.1699	NA	-8.1509	Significant
Intergenic	chr13	55423098-55423106	TCGCCATCA	1.1699	NA	-9.3305	Significant
Intergenic	chr17	661157-661165	ACGCCAACA	1.1699	NA	-7.2587	Significant
Intergenic	chr19	57016325-57016333	TGATGGCGT	1.1699	NA	-11.9042	Significant
Intergenic	chr21	21038655-21038663	TGATGGCGA	1.1699	NA	-6.6195	Significant
OPRM1	chr6	154401434-154401442	ACGCCTTCA	1.1575	-0.0628	-23.1641	Significant
RBM25	chr14	73569100-73569108	TGTTGGCGA	1.1575	-0.3928	-6.8297	Significant
Intergenic	chr2	126805855-126805863	ACGCCATCA	1.1520	NA	-10.0726	Significant
SPATS2L	chr2	201182064-201182072	ACGCCTACA	1.1520	0.0209	-10.8404	Significant
Intergenic	chr3	136953910-136953918	TGTTGGCGA	1.1520	NA	-8.6142	Significant
Intergenic	chr5	8684633-8684641	ACGCCATCA	1.1520	NA	-8.3811	Significant
Intergenic	chr5	24380522-24380530	TGAAGGCGT	1.1520	NA	-11.9042	Significant
Intergenic	chr5	100489462-100489470	TGTTGGCGT	1.1520	NA	-11.9042	Significant
FBXL17	chr5	107334664-107334672	TGAAGGCGA	1.1520	0.1326	-8.8501	Significant
BBS9	chr7	33631475-33631483	TCGCCATCA	1.1520	-0.0368	-7.9235	Significant
Intergenic	chr7	141974022-141974030	TGAAGGCGA	1.1520	NA	-6.0062	Significant
ANK3	chr10	61968887-61968895	TGATGGCGA	1.1520	0.0887	-10.8404	Significant
FAM149B1	chr10	74974980-74974988	TGTTGGCGA	1.1520	0.1764	-18.2397	Significant
Intergenic	chr12	125667715-125667723	TGTTGGCGT	1.1520	NA	-13.8759	Significant
NBEA	chr13	35842728-35842736	ACGCCAACA	1.1520	-0.1613	-10.3257	Significant
Intergenic	chr13	62087482-62087490	ACGCCTACA	1.1520	NA	-12.4532	Significant
ANKFY1	chr17	4096869-4096877	TCGCCATCA	1.1520	0.4010	-8.3811	Significant
DLGAP1	chr18	3687424-3687432	ACGCCAACA	1.1520	0.0757	-6.4122	Significant
DNAH14	chr1	225226640-225226648	TCGCCAACA	1.1375	0.2379	-8.6142	Significant
Intergenic	chr2	103941667-103941675	ACGCCATCA	1.1375	NA	-8.3811	Significant

Intergenic	chr3	88604949-88604957	TGTTGGCGT	1.1375	NA	-13.2983	Significant
TRIM61	chr4	165879342-165879350	ACGCCATCA	1.1375	-0.0499	-11.1021	Significant
Intergenic	chr6	132918679-132918687	TCGCCTTCA	1.1375	NA	-19.2478	Significant
RGS17	chr6	153340387-153340395	ACGCCATCA	1.1375	0.0815	-9.8224	Significant
Intergenic	chr9	42523636-42523644	TGAAGGCGT	1.1375	NA	-16.9357	Significant
Intergenic	chr9	120462782-120462790	TGTAGGCGA	1.1375	NA	-11.6339	Significant
Intergenic	chr11	131154756-131154764	ACGCCATCA	1.1375	NA	-7.0428	Significant
PRIM2	chr6	57445258-57445266	TCGCCTACA	1.1255	0.1547	-9.5750	Significant
Promoter_ZC3HC1	chr7	129692150-129692158	TCGCCAACA	1.1255	-1.2833	-8.1509	Significant
Intergenic	chr16	53081126-53081134	TGTTGGCGA	1.1255	NA	-7.2587	Significant
Intergenic	chr3	166683978-166683986	TGTAGGCGT	1.1155	NA	-10.5816	Significant
AHI1	chr6	135749017-135749025	TGAAGGCGA	1.1155	0.3613	-7.0428	Significant
MAGI2	chr7	78605429-78605437	TGAAGGCGT	1.1155	-0.4728	-9.0889	Significant
Intergenic	chr9	98417249-98417257	TGTTGGCGT	1.1155	NA	-6.4122	Significant
Intergenic	chr10	45689611-45689619	TCGCCATCA	1.1155	NA	-6.8297	Significant
PTEN	chr10	89653828-89653836	TGAAGGCGT	1.1155	-0.9384	-16.3009	Significant
PCF11	chr11	82874137-82874145	ACGCCATCA	1.1155	-4.2396	-7.9235	Significant
PPHLN1	chr12	42817887-42817895	TGATGGCGT	1.1155	-0.0120	-12.4532	Significant
Intergenic	chr13	79521740-79521748	TGTAGGCGT	1.1155	NA	-9.3305	Significant
FANCM	chr14	45648598-45648606	TCGCCATCA	1.1155	-2.6395	-11.6339	Significant
C17orf85	chr17	3721622-3721630	TGAAGGCGT	1.1155	-0.5489	-7.9235	Significant
Intergenic	chr19	42173193-42173201	TGAAGGCGA	1.1155	NA	-13.5857	Significant
OSBP2	chr22	31118303-31118311	TGTTGGCGT	1.1155	0.2721	-13.2983	Significant
LOC400927	chr22	38778163-38778171	ACGCCAACA	1.1155	0.1734	-7.4774	Significant
LOC400927-CSNK1E	chr22	38778163-38778171	ACGCCAACA	1.1155	NA	-7.4774	Significant
Intergenic	chr2	189521259-189521267	TGTTGGCGA	1.0995	NA	-9.3305	Significant
EGFEM1P	chr3	168428124-168428132	TGTAGGCGA	1.0995	0.2298	-8.3811	Significant
Intergenic	chr5	13408367-13408375	TGTTGGCGT	1.0995	NA	-8.6142	Significant
MMD2	chr7	4932027-4932035	TCGCCTACA	1.0995	0.0046	-13.2983	Significant
Intergenic	chr7	25592201-25592209	TGATGGCGA	1.0995	NA	-16.6169	Significant
Intergenic	chr8	59368756-59368764	ACGCCTACA	1.0995	NA	-10.5816	Significant
Intergenic	chr9	78397984-78397992	TCGCCAACA	1.0995	NA	-13.0137	Significant
Intergenic	chr10	129291781-129291789	TCGCCAACA	1.0995	NA	-9.5750	Significant
RSF1	chr11	77521806-77521814	TGTTGGCGA	1.0995	-1.9220	-11.9042	Significant
ZMYM2	chr13	20600537-20600545	TGAAGGCGA	1.0995	-0.8828	-10.5816	Significant
ATP8A2	chr13	26569936-26569944	TGATGGCGT	1.0995	0.2258	-6.8297	Significant
Intergenic	chr14	77199561-77199569	ACGCCAACA	1.0995	NA	-5.8074	Significant
Intergenic	chr17	50462178-50462186	TGTTGGCGT	1.0995	NA	-6.2078	Significant
Intergenic	chr20	7279053-7279061	ACGCCAACA	1.0995	NA	-7.4774	Significant
Intergenic	chr20	57675941-57675949	TGTTGGCGT	1.0995	NA	-29.0621	Significant
Intergenic	chrX	127951959-127951967	TGTAGGCGA	1.0995	NA	-11.1021	Significant
ELTD1	chr1	79382061-79382069	ACGCCATCA	1.0875	-0.2606	-11.3666	Significant
MAP4K3	chr2	39597468-39597476	TGATGGCGA	1.0875	-0.4952	-14.7638	Significant
Intergenic	chr2	106945585-106945593	ACGCCTTCA	1.0875	NA	-11.3666	Significant
KIAA1715	chr2	176821518-176821526	TCGCCTACA	1.0875	0.2888	-6.6195	Significant
RELN	chr7	103534896-103534904	ACGCCTTCA	1.0875	0.0457	-11.3666	Significant
Intergenic	chr7	117771535-117771543	TGTAGGCGT	1.0875	NA	-11.3666	Significant
Intergenic	chr7	123949807-123949815	TGATGGCGA	1.0875	NA	-19.2478	Significant
Intergenic	chr8	96120924-96120932	TGTTGGCGT	1.0875	NA	-15.6775	Significant
Intergenic	chr9	81419797-81419805	ACGCCAACA	1.0875	NA	-12.4532	Significant
Intergenic	chr9	89435102-89435110	TCGCCAACA	1.0875	NA	-7.6991	Significant
Intergenic	chr20	10940296-10940304	TCGCCATCA	1.0875	NA	-9.8224	Significant
PROKR1	chr2	68876022-68876030	TCGCCTACA	1.0780	0.3128	-9.8224	Significant
Intergenic	chr2	128805488-128805496	ACGCCAACA	1.0780	NA	-8.6142	Significant
IKZF2	chr2	213884797-213884805	ACGCCATCA	1.0780	-0.7298	-9.8224	Significant
STAG1	chr3	136458772-136458780	ACGCCAACA	1.0780	0.1546	-12.4532	Significant
NLGN1	chr3	173310292-173310300	ACGCCTACA	1.0780	-0.3248	-9.5750	Significant
AUTS2	chr7	69561391-69561399	TCGCCAACA	1.0780	-1.3668	-7.0428	Significant
FAT3	chr11	92449558-92449566	TCGCCATCA	1.0780	0.8027	-6.0062	Significant
Intergenic	chr13	61184866-61184874	TGAAGGCGT	1.0780	NA	-7.4774	Significant
Intergenic	chr20	22343672-22343680	ACGCCATCA	1.0780	NA	-11.6339	Significant
PCBP3	chr21	47342622-47342630	TGTAGGCGA	1.0780	0.1365	-7.0428	Significant
Intergenic	chrX	141174933-141174941	ACGCCAACA	1.0780	NA	-8.6142	Significant
Intergenic	chr3	150637632-150637640	TGAAGGCGT	1.0704	NA	-10.8404	Significant
MAD1L1	chr7	2021991-2021999	ACGCCATCA	1.0704	-0.6269	-10.8404	Significant
Intergenic	chr7	53803784-53803792	TGATGGCGT	1.0704	NA	-13.8759	Significant
Intergenic	chr7	79504685-79504693	TGTTGGCGT	1.0704	NA	-16.6169	Significant
Intergenic	chr8	49664479-49664487	TCGCCTTCA	1.0704	NA	-15.0655	Significant
Intergenic	chr11	93332643-93332651	TCGCCTACA	1.0704	NA	-8.6142	Significant
HEATR4	chr14	74003639-74003647	TCGCCATCA	1.0704	0.2109	-8.8501	Significant
Promoter_ACOT1	chr14	74003639-74003647	TCGCCATCA	1.0704	-0.0977	-8.8501	Significant
VPS53	chr17	554961-554969	ACGCCTTCA	1.0704	0.0633	-8.6142	Significant

SMARCE1	chr17	38798754-38798762	TGTAGGCGA	1.0704	0.6442	-7.6991	Significant
LINC00189	chr21	30577049-30577057	TCGCCTTCA	1.0704	0.1293	-9.3305	Significant
Intergenic	chrX	33808315-33808323	TCGCCAACA	1.0704	NA	-6.8297	Significant
MTA3	chr2	42743911-42743919	TGTAGGCGT	1.0641	-0.0556	-9.8224	Significant
SEMA3C	chr7	80391213-80391221	TCGCCAACA	1.0641	0.1718	-10.0726	Significant
Intergenic	chr9	79630738-79630746	TCGCCAACA	1.0589	NA	-13.0137	Significant
SMG6	chr17	2070834-2070842	ACGCCTTCA	1.0589	-0.8214	-13.8759	Significant
LOC100506393	chr12	20224040-20224048	ACGCCTACA	1.0545	0.2184	-10.3257	Significant
Intergenic	chr2	159572865-159572873	TCGCCTTCA	1.0506	NA	-10.5816	Significant
ROR1	chr1	64329312-64329320	TGATGGCGT	1.0310	-0.7008	-33.7963	Significant
Intergenic	chr8	129606269-129606277	TCGCCAACA	1.0231	NA	-5.2283	Significant
ST3GAL3	chr1	44253425-44253433	TGAAGGCGA	1.0000	-0.4125	-8.3811	Significant
Intergenic	chr1	55791667-55791675	TCGCCAACA	1.0000	NA	-15.0655	Significant
Intergenic	chr1	87576415-87576423	TGAAGGCGT	1.0000	NA	-20.9850	Significant
Intergenic	chr2	21604001-21604009	ACGCCATCA	1.0000	NA	-24.2922	Significant
CDKL4	chr2	39406329-39406337	TGTTGGCGT	1.0000	0.2756	-9.0889	Significant
Intergenic	chr2	76209036-76209044	TGTAGGCGA	1.0000	NA	-13.8759	Significant
Intergenic	chr2	82676574-82676582	TCGCCTTCA	1.0000	NA	-9.8224	Significant
KCNIP3	chr2	96032829-96032837	TCGCCAACA	1.0000	0.2402	-9.5750	Significant
Intergenic	chr2	124168310-124168318	TGTAGGCGT	1.0000	NA	-9.8224	Significant
ZNF385B	chr2	180602862-180602870	ACGCCTTCA	1.0000	0.1151	-9.8224	Significant
Intergenic	chr2	186361898-186361906	TGATGGCGT	1.0000	NA	-18.5729	Significant
DOCK10	chr2	225830837-225830845	ACGCCTTCA	1.0000	-0.0208	-11.3666	Significant
Intergenic	chr3	42401760-42401768	TGTTGGCGA	1.0000	NA	-9.8224	Significant
Intergenic	chr3	44329360-44329368	TGATGGCGA	1.0000	NA	-6.2078	Significant
Intergenic	chr3	111201221-111201229	TCGCCTTCA	1.0000	NA	-8.6142	Significant
Intergenic	chr3	136994116-136994124	TGTTGGCGT	1.0000	NA	-13.5857	Significant
Intergenic	chr3	145598555-145598563	TCGCCATCA	1.0000	NA	-11.9042	Significant
Intergenic	chr5	6436651-6436659	ACGCCAACA	1.0000	NA	-9.8224	Significant
Intergenic	chr5	18113510-18113518	TCGCCTTCA	1.0000	NA	-8.8501	Significant
CDH18	chr5	20304304-20304312	TGTTGGCGT	1.0000	0.0324	-7.0428	Significant
SCAMP1	chr5	77715110-77715118	TGTAGGCGT	1.0000	-0.0408	-9.8224	Significant
Intergenic	chr5	94647200-94647208	TCGCCAACA	1.0000	NA	-9.0889	Significant
Intergenic	chr5	98155028-98155036	ACGCCTACA	1.0000	NA	-9.8224	Significant
Intergenic	chr5	109529314-109529322	TGTAGGCGT	1.0000	NA	-13.0137	Significant
Intergenic	chr5	131500175-131500183	TCGCCAACA	1.0000	NA	-5.6115	Significant
ARHGAP26	chr5	142440466-142440474	TGTAGGCGA	1.0000	-0.3846	-8.8501	Significant
LARS	chr5	145552284-145552292	TGAAGGCGT	1.0000	0.6752	-9.5750	Significant
LARS	chr5	145552765-145552773	TGATGGCGT	1.0000	0.6752	-9.3305	Significant
Intergenic	chr6	9426634-9426642	TCGCCTTCA	1.0000	NA	-16.9357	Significant
Intergenic	chr6	92294574-92294582	ACGCCATCA	1.0000	NA	-16.6169	Significant
Intergenic	chr6	106435979-106435987	TCGCCATCA	1.0000	NA	-15.0655	Significant
Intergenic	chr7	10068881-10068889	TCGCCATCA	1.0000	NA	-6.6195	Significant
Intergenic	chr7	10235400-10235408	ACGCCTACA	1.0000	NA	-7.2587	Significant
RSBN1L	chr7	77342141-77342149	TGTAGGCGT	1.0000	-3.0508	-9.0889	Significant
Intergenic	chr7	130445568-130445576	TCGCCAACA	1.0000	NA	-7.4774	Significant
TEK	chr9	27182620-27182628	TCGCCTACA	1.0000	0.4177	-17.2575	Reference
Intergenic	chr9	80893270-80893278	TCGCCTACA	1.0000	NA	-6.0062	Significant
Intergenic	chr9	91884794-91884802	TGATGGCGA	1.0000	NA	-13.8759	Significant
GAPVD1	chr9	128036498-128036506	TGTTGGCGA	1.0000	-0.4604	-9.5750	Significant
Intergenic	chr10	88694763-88694771	ACGCCAACA	1.0000	NA	-4.8566	Significant
Intergenic	chr10	122710417-122710425	TCGCCTTCA	1.0000	NA	-10.0726	Significant
Intergenic	chr11	22958908-22958916	ACGCCTTCA	1.0000	NA	-7.6991	Significant
Intergenic	chr11	110941709-110941717	TGAAGGCGT	1.0000	NA	-9.5750	Significant
ETS1	chr11	128364113-128364121	ACGCCAACA	1.0000	0.0927	-12.4532	Significant
Intergenic	chr12	9976113-9976121	ACGCCTACA	1.0000	NA	-10.3257	Significant
OVCH1	chr12	29614917-29614925	TGTAGGCGA	1.0000	-0.0139	-7.6991	Significant
Intergenic	chr12	51943326-51943334	TGATGGCGT	1.0000	NA	-7.9235	Significant
SACS	chr13	23929918-23929926	TGTAGGCGA	1.0000	-1.3469	-11.3666	Significant
Intergenic	chr13	37309839-37309847	TGAAGGCGA	1.0000	NA	-10.3257	Significant
ENOX1	chr13	44016175-44016183	ACGCCTACA	1.0000	-0.5524	-8.6142	Significant
Intergenic	chr13	87602475-87602483	TGTAGGCGT	1.0000	NA	-15.9878	Significant
CLYBL	chr13	100275507-100275515	TCGCCAACA	1.0000	0.1182	-19.9341	Significant
ATL1	chr14	51034332-51034340	TCGCCATCA	1.0000	-0.8694	-10.0726	Significant
RAD51B	chr14	68354075-68354083	TCGCCATCA	1.0000	0.1567	-12.7321	Significant
DPF3	chr14	73206948-73206956	TGTTGGCGT	1.0000	-0.2185	-10.8404	Significant
MOCOS	chr18	33782029-33782037	ACGCCAACA	1.0000	0.1790	-15.3701	Significant
XRN2	chr20	21354480-21354488	TGTAGGCGT	1.0000	0.1401	-7.9235	Significant
Intergenic	chrX	109150326-109150334	ACGCCAACA	1.0000	NA	-4.8566	Significant
Intergenic	chr20	22487104-22487112	TCGCCATCA	0.9594	NA	-6.8297	Significant
Intergenic	chr4	152265822-152265830	ACGCCATCA	0.9475	NA	-23.9133	Significant
SAAL1	chr11	18123757-18123765	TCGCCAACA	0.9475	0.5510	-16.3009	Significant

STK4	chr20	43617902-43617910	TCGCCAACA	0.9475	-0.3530	-13.2983	Significant
USP34	chr2	61502621-61502629	TGATGGCGT	0.9434	-0.4724	-13.2983	Significant
Intergenic	chr5	3485081-3485089	TCGCCTTCA	0.9386	NA	-7.9235	Significant
Intergenic	chr7	89909203-89909211	TGAAGGCGA	0.9386	NA	-13.5857	Significant
TBXAS1	chr7	139654871-139654879	TGTAGGCGT	0.9386	0.3051	-7.4774	Significant
HYDIN	chr16	71059396-71059404	TGATGGCGT	0.9386	-0.0531	-12.7321	Significant
PTPRT	chr20	40762872-40762880	TGAAGGCGT	0.9386	0.0375	-9.0889	Significant
Intergenic	chr2	153844114-153844122	ACGCCATCA	0.9329	NA	-9.5750	Significant
EXT2	chr11	44144012-44144020	TCGCCATCA	0.9329	0.1042	-7.6991	Significant
Intergenic	chr19	54570175-54570183	TCGCCAACA	0.9329	NA	-10.0726	Significant
Intergenic	chr2	151227816-151227824	TGATGGCGT	0.9260	NA	-13.8759	Significant
Intergenic	chr3	195647019-195647027	TCGCCAACA	0.9260	NA	-7.6991	Significant
SOBP	chr6	107957902-107957910	TGATGGCGT	0.9260	-1.3875	-6.4122	Significant
PPP1R9A	chr7	94688240-94688248	TGTAGGCGT	0.9260	-0.0935	-7.6991	Significant
PDK4	chr7	95224405-95224413	TGTTGGCGA	0.9260	-1.0074	-6.0062	Significant
ANGPT1	chr8	108440181-108440189	TGAAGGCGT	0.9260	0.1273	-10.3257	Significant
Intergenic	chr9	74066254-74066262	TGTAGGCGT	0.9260	NA	-8.1509	Significant
Intergenic	chr12	25112087-25112095	TGTTGGCGA	0.9260	NA	-8.8501	Significant
CHN1	chr2	175751860-175751868	TGATGGCGA	0.9175	0.1904	-7.4774	Significant
Intergenic	chr3	166264298-166264306	TGAAGGCGT	0.9175	NA	-9.3305	Significant
Intergenic	chr5	88362672-88362680	TCGCCTTCA	0.9175	NA	-6.6195	Significant
RELN	chr7	103366749-103366757	TCGCCATCA	0.9175	0.0457	-6.6195	Significant
UBR5	chr8	103353764-103353772	TCGCCTTCA	0.9175	-0.4529	-7.2587	Significant
Intergenic	chr9	83102093-83102101	TGTTGGCGA	0.9175	NA	-11.1021	Significant
Intergenic	chr10	120437457-120437465	ACGCCAACA	0.9175	NA	-12.7321	Significant
Intergenic	chr12	34438167-34438175	TGTAGGCGT	0.9175	NA	-9.3305	Significant
Intergenic	chr13	19610585-19610593	TCGCCAACA	0.9175	NA	-13.5857	Significant
Intergenic	chr13	44528813-44528821	TGTTGGCGT	0.9175	NA	-19.5895	Significant
Intergenic	chr13	65396570-65396578	TGTTGGCGT	0.9175	NA	-23.5373	Significant
NRXN3	chr14	78818292-78818300	TGTTGGCGA	0.9175	-0.0589	-11.3666	Significant
Intergenic	chrX	54739062-54739070	TGTAGGCGT	0.9175	NA	-9.0889	Significant
TMEM132B	chr12	126039431-126039439	TGTTGGCGA	0.9125	-0.2807	-14.1690	Significant
DPY19L2P1	chr7	35223055-35223063	TGATGGCGA	0.9069	0.0105	-11.3666	Significant
Intergenic	chr7	73852126-73852134	TGATGGCGT	0.9069	NA	-10.0726	Significant
Intergenic	chr8	120407604-120407612	TGATGGCGA	0.9069	NA	-10.3257	Significant
PCGF5	chr10	92924541-92924549	TCGCCTTCA	0.9069	0.3945	-12.4532	Significant
Intergenic	chr11	103427562-103427570	TGTAGGCGT	0.9069	NA	-11.9042	Significant
Intergenic	chr12	102987543-102987551	TGATGGCGA	0.9069	NA	-9.5750	Significant
Intergenic	chr13	66119787-66119795	ACGCCTTCA	0.9069	NA	-10.0726	Significant
Intergenic	chr14	41620652-41620660	TGTTGGCGA	0.9069	NA	-8.6142	Significant
NKX2-4	chr20	21376222-21376230	ACGCCAACA	0.9069	-0.1176	-9.5750	Significant
NRD1	chr1	52268328-52268336	TGATGGCGT	0.8931	0.2767	-9.8224	Significant
Intergenic	chr1	154603660-154603668	TGTAGGCGT	0.8931	NA	-9.8224	Significant
MECOM	chr3	168860249-168860257	TGATGGCGA	0.8931	-0.1809	-8.3811	Significant
Intergenic	chr4	182532326-182532334	TGTAGGCGA	0.8931	NA	-6.2078	Significant
PPP2R2B	chr5	146216548-146216556	ACGCCAACA	0.8931	-0.1538	-9.0889	Significant
Intergenic	chr6	21409900-21409908	TGTTGGCGA	0.8931	NA	-10.8404	Significant
USP49	chr6	41800451-41800459	TCGCCTACA	0.8931	-0.3873	-10.8404	Significant
RIMS1	chr6	72764279-72764287	TCGCCAACA	0.8931	0.0175	-18.5729	Significant
Intergenic	chr6	164487420-164487428	ACGCCTACA	0.8931	NA	-8.1509	Significant
Intergenic	chr8	74254675-74254683	TGTAGGCGT	0.8931	NA	-11.6339	Significant
NBEA	chr13	35645839-35645847	TGATGGCGA	0.8931	-0.1613	-18.2397	Significant
DLEU1	chr13	50694694-50694702	TCGCCTTCA	0.8931	-0.7258	-20.2815	Significant
MYH10	chr17	8481708-8481716	ACGCCAACA	0.8931	0.2557	-10.3257	Significant
PHACTR3	chr20	58419103-58419111	TCGCCAACA	0.8931	-0.3202	-7.6991	Significant
Intergenic	chrX	14527758-14527766	TGAAGGCGT	0.8931	NA	-12.7321	Significant
Intergenic	chrX	64570037-64570045	ACGCCATCA	0.8931	NA	-13.8759	Significant
RNF2	chr1	185060543-185060551	TGTAGGCGA	-0.8745	-0.2888	-17.2575	Significant
Intergenic	chr12	4337463-4337471	ACGCCAACA	-0.8745	NA	-10.5816	Significant
Intergenic	chr2	16444919-16444927	TGAAGGCGA	0.8745	NA	-9.0889	Significant
Intergenic	chr2	104034402-104034410	TGTTGGCGT	0.8745	NA	-8.3811	Significant
Intergenic	chr2	176365422-176365430	TCGCCTTCA	0.8745	NA	-11.6339	Significant
LSAMP	chr3	115918472-115918480	TGTAGGCGA	0.8745	-0.1001	-10.5816	Significant
RSRC1	chr3	157981948-157981956	TGTAGGCGT	0.8745	0.4650	-14.4650	Significant
USO1	chr4	76698849-76698857	TGATGGCGA	0.8745	0.3617	-8.8501	Significant
GFM2	chr5	74018027-74018035	TCGCCAACA	0.8745	0.1657	-16.6169	Significant
Intergenic	chr6	31180167-31180175	TCGCCAACA	0.8745	NA	-10.8404	Significant
Intergenic	chr7	127277813-127277821	TGTTGGCGA	0.8745	NA	-9.0889	Significant
Intergenic	chr10	78476540-78476548	TGTTGGCGT	0.8745	NA	-9.5750	Significant
Intergenic	chr11	21819183-21819191	ACGCCATCA	0.8745	NA	-25.8364	Significant
Intergenic	chr14	39458749-39458757	TGATGGCGA	0.8745	NA	-8.6142	Significant
Intergenic	chr18	8483544-8483552	TGTTGGCGA	0.8745	NA	-13.2983	Significant

Intergenic	chr5	164863286-164863294	TGTAGGCGT	0.8625	NA	-12.7321	Significant
Intergenic	chr6	168389815-168389823	TCGCCATCA	0.8625	NA	-9.5750	Significant
Intergenic	chr1	95131606-95131614	TGTTGGCGA	0.8480	NA	-13.5857	Significant
Intergenic	chr2	68345010-68345018	TCGCCAACA	0.8480	NA	-7.4774	Significant
CACNA2D3	chr3	54415783-54415791	TCGCCTTCA	0.8480	0.1439	-11.9042	Significant
RYK	chr3	133876903-133876911	TGTTGGCGT	0.8480	-0.1709	-6.0062	Significant
Intergenic	chr4	190195010-190195018	TGAAGGCGT	0.8480	NA	-52.0000	Significant
SPATA9	chr5	95000195-95000203	TGAAGGCGA	0.8480	0.2258	-14.4650	Significant
Intergenic	chr7	28870246-28870254	TGTTGGCGT	0.8480	NA	-11.6339	Significant
Intergenic	chr7	91531347-91531355	ACGCCAACA	0.8480	NA	-6.2078	Significant
CADM1	chr11	115235572-115235580	TGTTGGCGT	0.8480	0.2176	-8.1509	Significant
SOX5	chr12	23717965-23717973	TGTAGGCGA	0.8480	0.7601	-14.1690	Significant
Intergenic	chr14	29215804-29215812	TGATGGCGT	0.8480	NA	-13.5857	Significant
KIAA0430	chr16	15727265-15727273	TGATGGCGT	0.8480	0.0443	-10.8404	Significant
PIP5K1C	chr19	3651161-3651169	ACGCCTTCA	0.8480	-0.4409	-12.7321	Significant
Intergenic	chr8	128145384-128145392	TGTTGGCGA	0.8340	NA	-6.0062	Significant
SPTBN1	chr2	54731700-54731708	TGTAGGCGT	-0.8301	0.1455	-4.8566	Reference
Intergenic	chr2	73132694-73132702	ACGCCATCA	0.8301	NA	-7.0428	Significant
Intergenic	chr6	99575432-99575440	TGATGGCGT	0.8301	NA	-12.4532	Significant
Intergenic	chr7	40712558-40712566	TGTAGGCGA	0.8301	NA	-11.1021	Significant
EZH2	chr7	148523589-148523597	TCGCCTACA	0.8301	-0.6766	-11.6339	Significant
Intergenic	chr9	85477810-85477818	ACGCCTACA	0.8301	NA	-13.8759	Significant
Intergenic	chr11	34410007-34410015	ACGCCATCA	0.8301	NA	-7.6991	Significant
Intergenic	chr11	37418162-37418170	TGTTGGCGT	0.8301	NA	-10.5816	Significant
Intergenic	chr11	55303111-55303119	TGAAGGCGT	0.8301	NA	-12.1773	Significant
Intergenic	chr13	23691398-23691406	TCGCCATCA	0.8301	NA	-9.8224	Significant
PAFAH1B1	chr17	2532512-2532520	TGTAGGCGT	0.8301	-1.7089	-10.0726	Significant
Intergenic	chr20	54374049-54374057	ACGCCTACA	0.8301	NA	-11.6339	Significant
Intergenic	chr20	51422764-51422772	TGAAGGCGA	0.8194	NA	-10.0726	Significant
Intergenic	chr2	181028542-181028550	TGTTGGCGT	0.8074	NA	-8.1509	Significant
DNAH5	chr5	13914133-13914141	TGAAGGCGA	0.8074	-0.4461	-8.3811	Significant
CRISP2	chr6	49677213-49677221	ACGCCATCA	0.8074	-0.3704	-9.0889	Significant
Intergenic	chr7	135342156-135342164	TGTTGGCGA	0.8074	NA	-10.8404	Significant
SMARCA2	chr9	2076428-2076436	ACGCCTACA	0.8074	0.2143	-11.1021	Significant
Intergenic	chr10	30370329-30370337	TGAAGGCGT	0.8074	NA	-8.3811	Significant
Intergenic	chr10	93422308-93422316	ACGCCTTCA	0.8074	NA	-10.5816	Significant
Intergenic	chr13	58040162-58040170	TCGCCTACA	0.8074	NA	-6.6195	Significant
ZNF234	chr19	44653142-44653150	TCGCCTACA	0.8074	-1.6160	-8.1509	Significant
Intergenic	chr20	11362853-11362861	ACGCCTACA	0.8074	NA	-9.0889	Significant
Intergenic	chr4	45730418-45730426	ACGCCTACA	0.7885	NA	-7.0428	Significant
Intergenic	chr5	145040507-145040515	TCGCCTACA	0.7885	NA	-11.1021	Significant
Intergenic	chr7	19563895-19563903	TGTTGGCGT	0.7885	NA	-7.2587	Significant
LHFPL3	chr7	103979558-103979566	TGATGGCGA	0.7885	0.6609	-6.6195	Significant
SPTBN1	chr2	54882314-54882322	TGTTGGCGA	-0.7776	0.1455	-7.0428	Reference
FAM69A	chr1	93378394-93378402	ACGCCTACA	0.7776	-1.0579	-9.5750	Significant
PLD1	chr3	171333667-171333675	TGTAGGCGT	0.7776	-0.2451	-10.8404	Significant
Intergenic	chr6	9255083-9255091	TGTTGGCGT	0.7776	NA	-17.9095	Significant
PCLO	chr7	82405973-82405981	TGATGGCGT	0.7776	0.1243	-8.3811	Significant
Intergenic	chr7	110086887-110086895	TGTTGGCGA	0.7776	NA	-14.7638	Significant
Intergenic	chr7	151996918-151996926	TCGCCTTCA	0.7776	NA	-7.6991	Significant
DOCK8	chr9	328085-328093	ACGCCTTCA	0.7776	0.3242	-13.0137	Significant
PCDH15	chr10	56146579-56146587	TGTTGGCGA	0.7776	-0.0158	-10.3257	Significant
Intergenic	chr11	10970808-10970816	TCGCCTTCA	0.7776	NA	-8.3811	Significant
Intergenic	chr13	46802667-46802675	ACGCCTTCA	0.7776	NA	-10.0726	Significant
Intergenic	chr1	111571659-111571667	ACGCCTTCA	0.7655	NA	-12.1773	Significant
NCOA1	chr2	24856375-24856383	TGAAGGCGA	0.7655	-0.4188	-10.8404	Significant
SULT1C2P1	chr2	108944109-108944117	TGATGGCGA	0.7655	-0.0635	-6.8297	Significant
LEKR1	chr3	156745858-156745866	TCGCCTACA	0.7655	0.0329	-9.5750	Significant
Intergenic	chr7	29777669-29777677	ACGCCATCA	0.7655	NA	-7.9235	Significant
Intergenic	chr11	74207542-74207550	ACGCCAACA	0.7655	NA	-15.6775	Significant
Intergenic	chr11	74207968-74207976	ACGCCAACA	0.7655	NA	-12.1773	Significant
Intergenic	chr18	8536428-8536436	TGAAGGCGA	0.7655	NA	-11.1021	Significant
ACN9	chr7	96746179-96746187	ACGCCATCA	0.7590	0.1060	-5.4185	Significant
Intergenic	chr8	129407302-129407310	TGTTGGCGT	0.7590	NA	-6.4122	Significant
SLC38A1	chr12	46624586-46624594	TGTTGGCGA	0.7590	-0.5396	-9.3305	Significant
YTHDC2	chr5	112907662-112907670	TCGCCTTCA	0.7521	-0.0392	-6.2078	Significant
CNIH4	chr1	224563736-224563744	ACGCCAACA	0.7370	0.2342	-23.1641	Significant
LRP1B	chr2	141906215-141906223	ACGCCAACA	0.7370	0.0880	-10.8404	Significant
Intergenic	chr2	150413062-150413070	TCGCCAACA	0.7370	NA	-11.6339	Significant
OLA1	chr2	175053040-175053048	ACGCCTACA	0.7370	0.6017	-17.5820	Significant
Intergenic	chr5	97903139-97903147	ACGCCATCA	0.7370	NA	-8.1509	Significant
FBXL17	chr5	107679064-107679072	TGAAGGCGA	0.7370	0.1326	-14.1690	Significant

ATXN1	chr6	16580982-16580990	ACGCCTACA	0.7370	-1.9921	-8.1509	Significant
Intergenic	chr6	101654042-101654050	TGAAGGCGT	0.7370	NA	-7.6991	Significant
Intergenic	chr6	154986260-154986268	TGTTGGCGA	0.7370	NA	-9.3305	Significant
PDE1C	chr7	31822121-31822129	TCGCCTTCA	0.7370	0.0682	-9.3305	Significant
FRMD4A	chr10	14268802-14268810	TGTTGGCGT	0.7370	-0.1383	-12.7321	Significant
GBF1	chr10	104069904-104069912	TCGCCTTCA	0.7370	-0.3318	-7.6991	Significant
Intergenic	chr12	23140500-23140508	TGAAGGCGA	0.7370	NA	-10.3257	Significant
SOX5	chr12	24447093-24447101	TCGCCTTCA	0.7370	0.7601	-9.3305	Significant
ENOX1	chr13	44218493-44218501	ACGCCTACA	0.7370	-0.5524	-7.6991	Significant
Intergenic	chr14	60954612-60954620	TGTTGGCGA	0.7370	NA	-6.6195	Significant
SLC24A4	chr14	92962335-92962343	TGATGGCGT	0.7370	-0.2421	-10.5816	Significant
RYR3	chr15	33848480-33848488	TGATGGCGT	0.7370	-0.1685	-10.8404	Significant
Intergenic	chr16	77017430-77017438	TCGCCAACA	0.7370	NA	-8.6142	Significant
CEP112	chr17	63834993-63835001	ACGCCAACA	0.7370	-0.0144	-15.6775	Significant
Intergenic	chr18	4785035-4785043	TCGCCAACA	0.7370	NA	-12.1773	Significant
TTC39C	chr18	21636604-21636612	TGTTGGCGA	0.7370	0.3413	-7.2587	Significant
ARFGAP3	chr22	43221132-43221140	TGATGGCGT	0.7370	0.1326	-8.1509	Significant
Intergenic	chrX	100440904-100440912	TGTAGGCGA	0.7370	NA	-12.4532	Significant
Intergenic	chr20	46808248-46808256	TCGCCATCA	0.7199	NA	-10.0726	Significant
Intergenic	chr2	55733715-55733723	ACGCCATCA	0.7105	NA	-13.2983	Significant
SDPR	chr2	192706736-192706744	TGAAGGCGT	0.7105	-0.1474	-6.4122	Significant
AGMO	chr7	15283113-15283121	TGTTGGCGT	0.7105	0.0549	-8.1509	Significant
Intergenic	chr7	41899943-41899951	TGTTGGCGA	0.7105	NA	-12.1773	Significant
Intergenic	chr7	77143509-77143517	TCGCCTACA	0.7105	NA	-8.8501	Significant
FKBP15	chr9	115952702-115952710	TGATGGCGA	0.7105	-2.0068	-5.2283	Significant
Intergenic	chr11	119336375-119336383	TGTAGGCGA	0.7105	NA	-12.1773	Significant
DOCK9	chr13	99646415-99646423	TGTTGGCGT	0.7105	-0.3366	-12.1773	Significant
MYT1L	chr2	1909905-1909913	TCGCCTACA	0.7004	0.2176	-20.9850	Significant
RNF144A	chr2	7106946-7106954	TGAAGGCGA	0.7004	0.2233	-13.0137	Significant
Intergenic	chr2	35023705-35023713	TCGCCTACA	0.7004	NA	-14.7638	Significant
OLA1	chr2	175039413-175039421	TCGCCTTCA	0.7004	0.6017	-5.6115	Significant
Intergenic	chr5	46121278-46121286	TGAAGGCGT	0.7004	NA	-11.3666	Significant
Intergenic	chr5	164802325-164802333	TGAAGGCGA	0.7004	NA	-8.8501	Significant
BACH2	chr6	90654745-90654753	TGTTGGCGA	0.7004	-0.1164	-12.7321	Significant
STEAP1B	chr7	22536513-22536521	ACGCCAACA	0.7004	-0.1446	-8.1509	Significant
Intergenic	chr14	82172854-82172862	TGTTGGCGT	0.7004	NA	-6.0062	Significant
WWOX	chr16	79150375-79150383	TCGCCTTCA	0.7004	0.2967	-8.6142	Significant
Intergenic	chr17	25301665-25301673	TGTAGGCGT	0.7004	NA	-15.3701	Significant
Intergenic	chrX	15360182-15360190	ACGCCATCA	0.7004	NA	-10.0726	Significant
AFTPH	chr2	64772017-64772025	TCGCCATCA	0.6919	-2.1122	-9.3305	Significant
Intergenic	chr5	116252530-116252538	TGATGGCGT	0.6919	NA	-9.3305	Significant
Intergenic	chr16	32140750-32140758	TGTAGGCGT	0.6881	NA	-32.0347	Significant
KRAS	chr12	25398279-25398286	TGWWGGCGW	-0.6781	-0.2893	-13.2983	Reference
RAB31	chr18	9795735-9795743	TGTTGGCGT	-0.6781	0.6015	-14.4650	Significant
Intergenic	chr2	14445417-14445425	TCGCCATCA	0.6781	NA	-8.3811	Significant
Intergenic	chr2	107712953-107712961	TGTTGGCGT	0.6781	NA	-9.3305	Significant
LRP1B	chr2	142012066-142012074	TGATGGCGT	0.6781	0.0880	-13.8759	Significant
PTPRG	chr3	61766562-61766570	TGATGGCGT	0.6781	-0.1811	-10.5816	Significant
Intergenic	chr7	54340851-54340859	ACGCCAACA	0.6781	NA	-5.4185	Significant
MAGI2	chr7	78987503-78987511	ACGCCAACA	0.6781	-0.4728	-7.2587	Significant
ZCCHC6	chr9	88931500-88931508	ACGCCAACA	0.6781	-0.7066	-8.6142	Significant
Intergenic	chr9	104987364-104987372	TGTAGGCGA	0.6781	NA	-10.3257	Significant
PCDH9	chr13	67119800-67119808	TGTTGGCGT	0.6781	0.0723	-10.0726	Significant
Intergenic	chr16	62657350-62657358	TCGCCAACA	0.6781	NA	-10.8404	Significant
Intergenic	chr18	1827019-1827027	TGATGGCGT	0.6781	NA	-9.5750	Significant
Intergenic	chr21	20646682-20646690	TCGCCATCA	0.6781	NA	-12.1773	Significant
SLC27A4	chr9	131117094-131117102	TGTTGGCGT	0.6674	-0.4429	-7.6991	Significant
MIER1	chr1	67416950-67416958	TGTAGGCGT	0.6630	-0.4871	-7.0428	Significant
BIRC6	chr2	32612641-32612649	TGATGGCGA	0.6630	-0.5994	-10.5816	Significant
Intergenic	chr2	123781507-123781515	TCGCCTTCA	0.6630	NA	-7.9235	Significant
MAP2	chr2	210349045-210349053	TGATGGCGA	0.6630	-0.1998	-20.6318	Significant
MAP2	chr2	210349130-210349138	TGATGGCGA	0.6630	-0.1998	-16.3009	Significant
CDH6	chr5	31301340-31301348	ACGCCTACA	0.6630	0.1019	-21.6999	Significant
Intergenic	chr7	1268653-1268661	TCGCCTTCA	0.6590	NA	-8.6142	Significant
Intergenic	chr1	213792299-213792307	TGATGGCGA	0.6521	NA	-12.4532	Significant
SDCCAG8	chr1	243455517-243455525	TGTTGGCGA	0.6521	-0.2042	-10.8404	Significant
Intergenic	chr2	45363290-45363298	TCGCCAACA	0.6521	NA	-10.3257	Significant
MAGI2	chr7	78563838-78563846	TGATGGCGA	0.6521	-0.4728	-8.6142	Significant
DPY19L2P2	chr7	102819965-102819973	TGATGGCGT	0.6521	-0.0127	-6.8297	Significant
CHD7	chr8	61687713-61687721	ACGCCTTCA	0.6521	-3.2927	-6.6195	Significant
CPA6	chr8	68364678-68364686	TGAAGGCGA	0.6521	-1.1668	-13.0137	Significant
Intergenic	chr11	110622193-110622201	TGTTGGCGA	0.6521	NA	-21.3410	Significant

BICD1	chr12	32349081-32349089	TGTTGGCGA	0.6521	-0.6934	-5.2283	Significant
TMEM117	chr12	44310632-44310640	TGTTGGCGT	0.6521	-1.3378	-6.2078	Significant
Intergenic	chr12	102975461-102975469	TCGCCTTCA	0.6521	NA	-11.9042	Significant
ZNF473	chr19	50537725-50537733	TGAAGGCGA	-0.6374	-1.9179	-11.1021	Significant
PLSCR5	chr3	146310371-146310379	TCGCCATCA	0.6374	0.4659	-15.0655	Significant
Intergenic	chr7	54130354-54130362	ACGCCTACA	0.6374	NA	-13.5857	Significant
RABGEF1	chr7	66176009-66176017	TGATGGCGT	0.6374	-1.4486	-8.1509	Significant
XPO7	chr8	21786055-21786063	TCGCCAACA	0.6374	-0.0523	-5.0410	Significant
TLE4	chr9	82336354-82336362	TGTAGGCGT	0.6374	-2.2614	-13.2983	Significant
Intergenic	chr12	20114519-20114527	TGAAGGCGA	0.6374	NA	-16.9357	Significant
Intergenic	chr17	48621890-48621898	TGATGGCGT	0.6374	NA	-7.6991	Significant
Intergenic	chr17	55846339-55846347	TCGCCATCA	0.6374	NA	-11.3666	Significant
Intergenic	chr20	20103558-20103566	ACGCCAACA	0.6374	NA	-13.5857	Significant
Intergenic	chr22	25692743-25692751	TGTAGGCGT	0.6374	NA	-7.9235	Significant
CBY1	chr22	39066837-39066845	TGAAGGCGT	0.6374	-0.0385	-9.8224	Significant
VEPH1	chr3	157062909-157062917	ACGCCTTCA	0.6215	0.2563	-22.7937	Significant
ZNF800	chr7	127012963-127012971	TCGCCTTCA	0.6215	-0.9839	-10.3257	Significant
KDM2A	chr11	66982664-66982672	TCGCCAACA	0.6215	-1.3328	-10.0726	Significant
Intergenic	chr6	156424916-156424924	TCGCCTACA	-0.5850	NA	-8.8501	Significant
BCL2	chr18	60947699-60947707	ACGCCTACA	-0.5850	-0.4310	-20.9850	Reference
KCNK1	chr1	233803155-233803163	TGTAGGCGT	0.5850	-0.7245	-17.2575	Significant
Intergenic	chr2	9829282-9829290	ACGCCAACA	0.5850	NA	-11.3666	Significant
EHBP1	chr2	63252342-63252350	TGATGGCGA	0.5850	-0.5562	-10.0726	Significant
Intergenic	chr2	76912645-76912653	ACGCCATCA	0.5850	NA	-8.1509	Significant
GALNT13	chr2	155179049-155179057	TGAAGGCGT	0.5850	0.2557	-15.9878	Significant
Intergenic	chr2	177936250-177936258	TGAAGGCGT	0.5850	NA	-12.7321	Significant
PLCL1	chr2	198994481-198994489	TCGCCTACA	0.5850	-0.1240	-13.0137	Significant
CISD2	chr4	103806570-103806578	TGTTGGCGT	0.5850	0.2928	-18.5729	Significant
SLC9B1	chr4	103806570-103806578	TGTTGGCGT	0.5850	-0.0923	-18.5729	Significant
Intergenic	chr4	185005372-185005380	ACGCCATCA	0.5850	NA	-25.4461	Significant
Intergenic	chr5	104835655-104835663	ACGCCATCA	0.5850	NA	-6.6195	Significant
ZNF346	chr5	176476597-176476605	TGAAGGCGT	0.5850	-3.8093	-11.6339	Significant
MOG	chr6	29633576-29633584	TGTAGGCGA	0.5850	0.1128	-13.0137	Significant
Intergenic	chr7	48110090-48110098	TGATGGCGA	0.5850	NA	-17.5820	Significant
Intergenic	chr8	20291898-20291906	TGTTGGCGT	0.5850	NA	-10.3257	Significant
Intergenic	chr9	82415067-82415075	TCGCCTACA	0.5850	NA	-8.8501	Significant
Intergenic	chr9	85229782-85229790	ACGCCATCA	0.5850	NA	-13.8759	Significant
SLC28A3	chr9	86934748-86934756	ACGCCTTCA	0.5850	0.1983	-7.9235	Significant
SVIL	chr10	29820828-29820836	TCGCCAACA	0.5850	-0.4986	-11.1021	Significant
Intergenic	chr11	23173619-23173627	TGTAGGCGT	0.5850	NA	-12.7321	Significant
Intergenic	chr11	37419078-37419086	ACGCCTTCA	0.5850	NA	-9.8224	Significant
Intergenic	chr11	44084581-44084589	TCGCCTTCA	0.5850	NA	-7.2587	Significant
CNTN5	chr11	99987070-99987078	ACGCCTTCA	0.5850	0.0250	-13.8759	Significant
CNTN5	chr11	99987070-99987078	TCGCCTTCA	0.5850	0.0250	-13.8759	Significant
Intergenic	chr11	109265773-109265781	ACGCCAACA	0.5850	NA	-9.8224	Significant
WNK1	chr12	890361-890369	ACGCCTACA	0.5850	-0.1781	-13.8759	Significant
Intergenic	chr12	21271991-21271999	TGTAGGCGT	0.5850	NA	-12.4532	Significant
SERTM1	chr13	37262497-37262505	ACGCCATCA	0.5850	-0.0852	-5.4185	Significant
Intergenic	chr13	52865401-52865409	TCGCCATCA	0.5850	NA	-12.7321	Significant
GPC6	chr13	94833526-94833534	TCGCCATCA	0.5850	0.1985	-7.9235	Significant
Intergenic	chr14	106721875-106721883	TCGCCTTCA	0.5850	NA	-13.5857	Significant
Intergenic	chr17	18612941-18612949	ACGCCATCA	0.5850	NA	-10.8404	Significant
PITPNC1	chr17	65591792-65591800	TGATGGCGA	0.5850	0.1275	-9.0889	Significant
BPTF	chr17	65930496-65930504	TGATGGCGA	0.5850	-0.8145	-14.1690	Significant
Intergenic	chr20	43838788-43838796	ACGCCTACA	0.5850	NA	-6.8297	Significant
Intergenic	chrX	139327694-139327702	ACGCCAACA	0.5850	NA	-14.7638	Significant
Promoter_CDC27	chr17	45266858-45266866	ACGCCTTCA	-0.5795	-0.4545	-52.0000	Significant
CYP3A4	chr7	99355203-99355211	ACGCCAACA	0.5619	-0.2544	-8.8501	Significant
Intergenic	chr7	149438188-149438196	TGAAGGCGT	-0.5525	NA	-10.3257	Significant
Intergenic	chr20	22167431-22167439	TGATGGCGA	0.5525	NA	-8.3811	Significant
Intergenic	chr20	55499844-55499852	TGAAGGCGT	0.5525	NA	-9.5750	Significant
Intergenic	chr20	22076259-22076267	ACGCCAACA	-0.5475	NA	-12.1773	Significant
Intergenic	chr14	62227756-62227764	TGAAGGCGT	0.5475	NA	-7.4774	Significant
Intergenic	chr16	24712219-24712227	ACGCCTTCA	0.5475	NA	-8.6142	Significant
Intergenic	chr21	32264562-32264570	TGATGGCGA	0.5475	NA	-8.1509	Significant
Intergenic	chr21	37254690-37254698	TGATGGCGT	0.5475	NA	-8.8501	Significant
ZBBX	chr3	166972410-166972418	ACGCCAACA	0.5406	0.1322	-15.0655	Significant
ELMO1	chr7	37048437-37048445	TGATGGCGA	0.5406	-0.0560	-25.0586	Significant
CACNA2D1	chr7	81735143-81735151	ACGCCTACA	0.5406	0.0695	-10.5816	Significant
Intergenic	chr7	108594808-108594816	ACGCCATCA	0.5406	NA	-13.8759	Significant
CNTNAP2	chr7	147487757-147487765	TCGCCATCA	0.5406	0.0521	-9.5750	Significant
PAPPA	chr9	118937330-118937338	TCGCCAACA	0.5406	-0.0968	-13.2983	Significant

Intergenic	chr10	77030143-77030151	ACGCCAACA	0.5406	NA	-10.5816	Significant
CRTAC1	chr10	99773815-99773823	TGATGGCGT	0.5406	-0.1257	-5.8074	Significant
ANKFY1	chr17	4085054-4085062	ACGCCTTCA	0.5406	0.4010	-7.0428	Significant
BCAS3	chr17	58923822-58923830	TGATGGCGT	0.5406	-0.1306	-9.0889	Significant
SGCD	chr5	156024278-156024286	TGTAGGCGA	0.5305	-0.0429	-8.1509	Significant
NRG1	chr8	31904815-31904823	TGTAGGCGA	0.5305	0.1984	-11.3666	Significant
Intergenic	chr9	95456175-95456183	TGTAGGCGT	0.5305	NA	-10.3257	Significant
CWF19L2	chr11	107205498-107205506	TCGCCTTCA	0.5305	-0.2574	-6.6195	Significant
Intergenic	chr12	55381046-55381054	TCGCCAACA	0.5305	NA	-7.2587	Significant
Intergenic	chr13	40612871-40612879	TGAAGGCGT	0.5305	NA	-8.8501	Significant
Intergenic	chr14	65167999-65168007	TGATGGCGA	0.5305	NA	-7.4774	Significant
EIF4G3	chr1	21170791-21170799	TCGCCAACA	0.5146	0.4140	-7.4774	Significant
SULT6B1	chr2	37402312-37402320	TCGCCATCA	0.5146	0.2362	-9.8224	Significant
PSME4	chr2	54127042-54127050	TGTTGGCGT	0.5146	-1.3197	-6.8297	Significant
USO1	chr4	76665619-76665627	TCGCCAACA	0.5146	0.3617	-11.1021	Significant
TBXAS1	chr7	139536961-139536969	TGTTGGCGA	0.5146	0.3051	-12.1773	Significant
Intergenic	chr10	78592093-78592101	TGAAGGCGA	0.5146	NA	-16.9357	Significant
ATRNL1	chr10	117494649-117494657	TCGCCTTCA	0.5146	0.1957	-15.9878	Significant
Intergenic	chr11	87189274-87189282	ACGCCAACA	0.5146	NA	-7.2587	Significant
GUCY1A2	chr11	106685679-106685687	ACGCCAACA	0.5146	-0.0035	-26.6257	Significant
Intergenic	chr17	13697204-13697212	TGAAGGCGT	0.5146	NA	-6.4122	Significant
Intergenic	chr21	43097064-43097072	TCGCCAACA	0.5146	NA	-13.8759	Significant
NEB	chr2	152385738-152385746	TGTAGGCGT	-0.5025	-0.0993	-19.5895	Significant
NECAB1	chr8	91929328-91929336	TGATGGCGT	-0.5025	-0.3033	-10.8404	Significant
Intergenic	chr13	91670530-91670538	TGATGGCGT	-0.5025	NA	-13.0137	Significant
Intergenic	chr6	84831226-84831234	TGATGGCGT	0.5025	NA	-11.3666	Significant
Intergenic	chr7	76430180-76430188	TGTTGGCGA	0.5025	NA	-6.4122	Significant
CNOT4	chr7	135073037-135073045	TCGCCAACA	0.5025	-1.4137	-10.5816	Significant
BCL2	chr18	60792487-60792495	TGTTGGCGT	-0.4854	-0.4310	0.0000	Reference
Intergenic	chr5	99862405-99862413	TCGCCAACA	0.4854	NA	-13.8759	Significant
BBS9	chr7	33294548-33294556	TGTTGGCGA	0.4854	-0.0368	-12.1773	Significant
Intergenic	chr10	31501182-31501190	TGTTGGCGA	0.4854	NA	-8.1509	Significant
SOX5	chr12	23841797-23841805	TGTAGGCGT	0.4854	0.7601	-8.6142	Significant
ANGPT4	chr20	882796-882804	TGATGGCGT	0.4854	-0.0080	-8.6142	Significant
ACSL4	chrX	108921613-108921621	TGAAGGCGT	0.4854	0.4076	-11.6339	Significant
ENOX2	chrX	129967645-129967653	TGAAGGCGA	0.4854	-0.2358	-9.5750	Significant
Intergenic	chr7	64960955-64960963	ACGCCTACA	0.4792	NA	-46.8915	Significant
Intergenic	chr13	60089552-60089560	TCGCCTACA	-0.4739	NA	-45.8401	Significant
Promoter_LOC100289650	chr19	43325653-43325661	TGTTGGCGA	0.4739	0.1301	-9.0889	Significant
ZNRF2	chr7	30355369-30355377	ACGCCTACA	0.4695	-0.7023	-7.9235	Significant
Intergenic	chr7	87860344-87860352	TCGCCTACA	0.4695	NA	-11.6339	Significant
PPP1R9A	chr7	94638733-94638741	TCGCCATCA	0.4695	-0.0935	-7.2587	Significant
ACN9	chr7	96782133-96782141	TGTAGGCGT	0.4695	0.1060	-8.6142	Significant
Intergenic	chr7	98614515-98614523	TGTTGGCGT	0.4695	NA	-8.1509	Significant
Intergenic	chr7	115835379-115835387	TGTTGGCGA	0.4695	NA	-8.1509	Significant
Intergenic	chr2	105036-105044	TGATGGCGT	0.4594	NA	-14.4650	Significant
Intergenic	chr2	6918486-6918494	TGAAGGCGA	0.4594	NA	-8.8501	Significant
PROM1	chr4	15998356-15998364	TGATGGCGA	0.4594	-0.1748	-13.2983	Significant
MAST4	chr5	66213739-66213747	TGAAGGCGT	0.4594	-0.5121	-20.2815	Significant
CNTNAP2	chr7	146636519-146636527	TGAAGGCGA	0.4594	0.0521	-9.5750	Significant
Intergenic	chr8	63130168-63130176	TGAAGGCGA	0.4594	NA	-8.8501	Significant
GRIK4	chr11	120739776-120739784	ACGCCATCA	0.4594	0.0131	-5.4185	Significant
FRMD5	chr15	44303939-44303947	ACGCCATCA	0.4594	-0.7395	-7.6991	Significant
DCAF7	chr17	61657813-61657821	TGATGGCGA	0.4594	-0.2056	-10.0726	Significant
Intergenic	chr18	4623308-4623316	TGTAGGCGT	0.4594	NA	-5.0410	Significant
Intergenic	chrX	20499263-20499271	TGTAGGCGT	0.4594	NA	-8.1509	Significant
Intergenic	chr2	118468406-118468414	TGAAGGCGA	-0.4475	NA	-16.6169	Significant
PKP4	chr2	159377528-159377536	TCGCCTTCA	0.4475	0.3216	-9.8224	Significant
Intergenic	chr6	86121459-86121467	ACGCCAACA	0.4475	NA	-18.2397	Significant
SEMA3A	chr7	83701261-83701269	TCGCCTTCA	0.4475	-0.8807	-9.5750	Significant
LMTK2	chr7	97800860-97800868	TGAAGGCGT	0.4475	-2.0397	-13.0137	Significant
KIF27	chr9	86518424-86518432	TGTTGGCGT	0.4475	0.0157	-10.5816	Significant
STIM1	chr11	3943668-3943676	TGTAGGCGT	0.4475	-0.1578	-9.0889	Significant
Intergenic	chr11	46159408-46159416	TGTAGGCGT	0.4475	NA	-8.1509	Significant
Intergenic	chr3	66558706-66558714	TCGCCAACA	0.4406	NA	-7.4774	Significant
Intergenic	chr5	113478164-113478172	ACGCCATCA	0.4406	NA	-18.9089	Significant
Intergenic	chr22	33584931-33584939	TCGCCAACA	0.4361	NA	-9.3305	Significant
Intergenic	chr9	93167096-93167104	TCGCCTACA	-0.4150	NA	-10.0726	Significant
Intergenic	chr1	737261-737269	TCGCCTACA	0.4150	NA	-11.3666	Significant
ZBTB20	chr3	114615851-114615859	TGATGGCGA	0.4150	0.0225	-14.1690	Significant
FAM81B	chr5	94757725-94757733	ACGCCATCA	0.4150	0.0057	-7.4774	Significant
IMMP2L	chr7	111195927-111195935	TGTTGGCGA	0.4150	0.4922	-9.5750	Significant

Intergenic	chr7 121295603-121295611	TGTTGGCGT	0.4150	NA	-9.3305	Significant
C8orf76	chr8 124241726-124241734	ACGCCAACA	0.4150	-1.2061	-10.3257	Significant
ZHX1-C8orf76	chr8 124241726-124241734	ACGCCAACA	0.4150	-1.2116	-10.3257	Significant
LOC100499484	chr9 100012452-100012460	ACGCCTACA	0.4150	-0.4078	-7.4774	Significant
Intergenic	chr11 81488677-81488685	TGTAGGCGA	0.4150	NA	-5.4185	Significant
Intergenic	chr12 47306950-47306958	ACGCCAACA	0.4150	NA	-10.3257	Significant
MTUS2	chr13 29672437-29672445	TGAAGGCGT	0.4150	-0.1433	-11.3666	Significant
Intergenic	chr16 24446314-24446322	ACGCCTACA	0.4150	NA	-7.0428	Significant
GFOD2	chr16 67728045-67728053	ACGCCATCA	0.4150	-2.6903	-9.8224	Significant
Intergenic	chr19 32019806-32019814	ACGCCATCA	0.4150	NA	-14.4650	Significant
Intergenic	chrX 23536434-23536442	TGTTGGCGT	0.4150	NA	-12.4532	Significant
OSBPL1A	chr18 21977368-21977376	ACGCCAACA	0.3923	0.3146	-20.9850	Significant
Intergenic	chr7 55939025-55939033	TCGCCATCA	0.3870	NA	-9.8224	Significant
FBXL13	chr7 102697256-102697264	ACGCCAACA	0.3870	0.4283	-8.1509	Significant
Intergenic	chr2 3979970-3979978	ACGCCAACA	-0.3785	NA	-13.2983	Significant
Intergenic	chr11 56507632-56507640	TGTAGGCGT	-0.3785	NA	-10.0726	Significant
TMEM131	chr2 98406127-98406135	ACGCCATCA	0.3785	0.2923	-10.3257	Significant
FRAS1	chr4 79281675-79281683	TGTAGGCGT	0.3785	-0.4685	-7.6991	Significant
Intergenic	chr5 162443258-162443266	TGTAGGCGA	0.3785	NA	-7.6991	Significant
Intergenic	chr6 108284907-108284915	ACGCCTACA	0.3785	NA	-9.8224	Significant
Intergenic	chr7 33156314-33156322	TCGCCAACA	0.3785	NA	-6.0062	Significant
Intergenic	chr7 36857673-36857681	TCGCCAACA	0.3785	NA	-13.8759	Significant
Intergenic	chr7 45556736-45556744	ACGCCAACA	0.3720	NA	-10.0726	Significant
Intergenic	chr17 32489568-32489576	TCGCCTACA	-0.3626	NA	-8.1509	Significant
RAP1A	chr1 112257901-112257909	TGTTGGCGA	0.3626	0.3870	-8.8501	Significant
Intergenic	chr1 200235249-200235257	TCGCCTTCA	0.3626	NA	-11.3666	Significant
XRCC5	chr2 217023073-217023081	ACGCCATCA	0.3626	0.2337	-13.8759	Significant
Intergenic	chr3 193443438-193443446	TGTAGGCGA	0.3626	NA	-7.6991	Significant
Intergenic	chr7 142363576-142363584	TGAAGGCGT	0.3626	NA	-9.0889	Significant
Intergenic	chr13 55589627-55589635	TGATGGCGT	0.3626	NA	-9.5750	Significant
Intergenic	chr15 36007159-36007167	TGATGGCGT	0.3626	NA	-12.1773	Significant
NXN	chr17 867859-867867	ACGCCTACA	0.3536	-0.3339	-9.0889	Significant
CNTNAP2	chr7 147361623-147361631	TCGCCTTCA	-0.3479	0.0521	-9.5750	Significant
Intergenic	chr2 103548808-103548816	TCGCCAACA	0.3479	NA	-9.5750	Significant
Intergenic	chr5 18376308-18376316	TGAAGGCGT	0.3479	NA	-13.5857	Significant
Intergenic	chr9 111703629-111703637	TGAAGGCGT	0.3479	NA	-10.5816	Significant
Intergenic	chr21 26866788-26866796	TGAAGGCGA	0.3479	NA	-9.0889	Significant
Intergenic	chr7 155359911-155359919	TGTTGGCGA	0.3410	NA	-10.0726	Significant
C9orf3	chr9 97648653-97648661	ACGCCTACA	0.3410	0.0741	-9.3305	Significant
Intergenic	chr2 126318923-126318931	ACGCCATCA	-0.3219	NA	-8.3811	Significant
TMTC1	chr12 29664386-29664394	TGATGGCGA	-0.3219	-0.5363	-25.8364	Significant
Intergenic	chr2 36262806-36262814	TCGCCATCA	0.3219	NA	-9.3305	Significant
Intergenic	chr2 184359749-184359757	TCGCCATCA	0.3219	NA	-14.1690	Significant
Intergenic	chr3 136526772-136526780	TGTTGGCGA	0.3219	NA	-11.6339	Significant
TYW1B	chr7 72059368-72059376	ACGCCAACA	0.3219	NA	-14.1690	Significant
OXR1	chr8 107525197-107525205	TGTTGGCGT	0.3219	-0.4905	-14.1690	Significant
C9orf3	chr9 97493245-97493253	TGAAGGCGA	0.3219	0.0741	-6.2078	Significant
Intergenic	chr11 130627011-130627019	TGAAGGCGA	0.3219	NA	-6.8297	Significant
Intergenic	chr14 54061961-54061969	ACGCCTACA	0.3219	NA	-13.0137	Significant
Intergenic	chr20 56550111-56550119	TCGCCATCA	0.3219	NA	-8.1509	Significant
GDAP1L1	chr20 42880715-42880723	TCGCCTACA	-0.3049	-0.0677	-4.8566	Significant
FBXO31	chr16 87383642-87383650	TGTTGGCGA	0.3049	-2.6107	-16.6169	Significant
SPTBN1	chr2 54833043-54833051	TCGCCATCA	0.2996	0.1455	0.0000	Reference
Intergenic	chr2 161974195-161974203	TGAAGGCGA	0.2996	NA	-12.7321	Significant
ABL1	chr9 133701269-133701277	TCGCCATCA	0.2996	-1.4226	-12.1773	Significant
Intergenic	chr11 38411226-38411234	TCGCCAACA	0.2996	NA	-9.3305	Significant
PTPRT	chr20 41759635-41759643	ACGCCTACA	0.2996	0.0375	-7.4774	Significant
Intergenic	chr2 220643340-220643348	TCGCCATCA	-0.2895	NA	-15.9878	Significant
Intergenic	chr3 144040797-144040805	ACGCCAACA	0.2895	NA	-8.8501	Significant
Intergenic	chr7 107458215-107458223	TGAAGGCGT	0.2895	NA	-6.4122	Significant
Intergenic	chr7 111254541-111254549	ACGCCATCA	0.2895	NA	-9.5750	Significant
Intergenic	chr11 28432746-28432754	TCGCCATCA	0.2895	NA	-8.8501	Significant
Intergenic	chr12 115610540-115610548	TGTTGGCGT	0.2895	NA	-13.2983	Significant
TM9SF4	chr20 30710410-30710418	TGATGGCGT	0.2895	-0.2973	-52.0000	Significant
ZBTB20	chr3 114109670-114109678	TCGCCATCA	0.2801	0.0225	-12.4532	Significant
Intergenic	chr5 95803440-95803448	TGTTGGCGT	0.2801	NA	-10.5816	Significant
Intergenic	chr20 346595-346603	TCGCCATCA	0.2801	NA	-5.8074	Significant
Intergenic	chr3 88431190-88431198	TGTAGGCGT	-0.2630	NA	-8.8501	Significant
Intergenic	chr10 27157986-27157994	TGTTGGCGA	-0.2630	NA	-22.0617	Significant
CD59	chr11 33734610-33734618	ACGCCATCA	-0.2630	0.3253	-5.2283	Significant
Intergenic	chr1 4485398-4485406	TGAAGGCGT	0.2630	NA	-13.0137	Significant
SPTBN1	chr2 54898020-54898028	TCGCCATCA	0.2630	0.1455	-9.0889	Reference

ARMC2	chr6	109226422-109226430	TGTTGGCGT	0.2630	0.0404	-12.4532	Significant
BBS9	chr7	33606657-33606665	TCGCCTTCA	0.2630	-0.0368	-9.0889	Significant
MAGI2	chr7	78546175-78546183	TGAAGGCGA	0.2630	-0.4728	-7.6991	Significant
CADPS2	chr7	122485982-122485990	TCGCCAACA	0.2630	0.0601	-11.3666	Significant
DAPK1	chr9	90201065-90201073	TGATGGCGT	0.2630	-0.0433	-8.6142	Significant
KIAA0368	chr9	114163613-114163621	TGATGGCGA	0.2630	0.6200	-9.3305	Significant
ZNF816-ZNF321P	chr19	53451781-53451789	ACGCCAACA	0.2630	-0.1457	-19.2478	Significant
Intergenic	chr1	59208062-59208070	TGAAGGCGA	-0.2410	NA	-14.4650	Significant
Intergenic	chr6	164245618-164245626	ACGCCAACA	-0.2410	NA	-17.5820	Significant
Intergenic	chr5	96173617-96173625	ACGCCATCA	0.2410	NA	-12.1773	Significant
Intergenic	chr5	124413786-124413794	TGTTGGCGA	0.2410	NA	-6.8297	Significant
Intergenic	chr6	81241580-81241588	TGTTGGCGT	0.2410	NA	-7.0428	Significant
Intergenic	chr7	54207630-54207638	TGATGGCGA	0.2410	NA	-6.2078	Significant
Intergenic	chr12	48824436-48824444	TGTTGGCGT	0.2410	NA	-13.2983	Significant
Intergenic	chr17	17097318-17097326	ACGCCAACA	-0.2345	NA	-8.1509	Significant
Intergenic	chr7	106324912-106324920	TGTTGGCGT	0.2345	NA	-8.1509	Significant
Intergenic	chr8	94044026-94044034	TGTTGGCGT	-0.2224	NA	-23.9133	Significant
Intergenic	chr12	110795810-110795818	TGAAGGCGA	-0.2224	NA	-10.0726	Significant
Intergenic	chr8	127311767-127311775	TGTAGGCGT	0.2224	NA	-14.1690	Significant
Intergenic	chr2	133034297-133034305	ACGCCTACA	0.2145	NA	-28.2385	Significant
COL28A1	chr7	7566413-7566421	ACGCCAACA	-0.2065	-0.0920	-19.2478	Significant
Intergenic	chr11	6999636-6999644	TGTTGGCGA	-0.2065	NA	-12.4532	Significant
FCGBP	chr19	40436693-40436701	ACGCCATCA	-0.2065	0.2800	-8.6142	Significant
PCDH9	chr13	67691398-67691406	TGAAGGCGA	0.2065	0.0723	-35.1474	Significant
COL21A1	chr6	56025709-56025717	ACGCCTTCA	0.1927	0.1771	-10.8404	Significant
Intergenic	chr1	20685423-20685431	ACGCCTACA	0.1844	NA	-26.2296	Significant
Intergenic	chr2	375379-375387	ACGCCTTCA	0.1806	NA	-10.5816	Significant
MBD5	chr2	149063719-149063727	ACGCCTACA	-0.1699	0.3670	-27.4264	Significant
Intergenic	chr3	148056048-148056056	TGTTGGCGA	-0.1699	NA	-13.2983	Significant
Intergenic	chr7	66964948-66964956	TGTTGGCGA	-0.1699	NA	-9.5750	Significant
HYDIN	chr16	71063874-71063882	TGATGGCGA	0.1699	-0.0531	-5.4185	Significant
CTPS2	chrX	16626070-16626078	ACGCCTACA	0.1699	0.1782	-8.6142	Significant
TMEM169	chr2	216947983-216947991	ACGCCATCA	0.1605	-0.1244	-14.7638	Significant
Intergenic	chr5	131840736-131840744	TCGCCTACA	0.1605	NA	-8.8501	Significant
BCKDHB	chr6	80926471-80926479	TGATGGCGT	-0.1520	0.4939	-8.8501	Significant
Intergenic	chr6	163800934-163800942	TGATGGCGA	-0.1520	NA	-9.8224	Significant
Intergenic	chr5	166522654-166522662	ACGCCTACA	0.1520	NA	-14.1690	Significant
Intergenic	chr14	105298138-105298146	TGTAGGCGT	0.1520	NA	-11.6339	Significant
Intergenic	chr18	1751017-1751025	TGAAGGCGA	0.1520	NA	-17.9095	Significant
Intergenic	chr3	44558595-44558603	TCGCCTACA	-0.1375	NA	-18.9089	Significant
Intergenic	chr9	109173767-109173775	TGAAGGCGA	-0.1375	NA	-9.0889	Significant
Intergenic	chr17	38870493-38870501	TGTTGGCGA	0.1375	NA	-10.0726	Significant
Intergenic	chr7	135574887-135574895	TGTTGGCGT	-0.1255	NA	-9.5750	Significant
PPP1R13L	chr19	45892197-45892205	TGTTGGCGA	-0.1255	-0.9693	-9.5750	Significant
PIK3CA	chr3	178943684-178943692	TGATGGCGT	0.1255	-0.6009	-9.8224	Reference
DLGAP1	chr18	3920961-3920969	TCGCCATCA	0.1255	0.0757	-6.8297	Significant
USP14	chr18	179726-179734	TGTAGGCGT	-0.1203	0.4851	-10.5816	Significant
DICER1	chr14	95609580-95609588	ACGCCTACA	-0.1155	-1.0892	-11.6339	Significant
ANKH	chr5	14764569-14764577	TCGCCAACA	0.1155	0.4402	-11.1021	Significant
Intergenic	chr7	110020628-110020636	TCGCCATCA	0.1155	NA	-10.3257	Significant
BTRC	chr10	103252403-103252411	TGAAGGCGT	0.1155	-0.7855	-11.3666	Significant
KLHL28	chr14	45415040-45415048	TGTTGGCGA	0.1155	-0.6758	-14.1690	Significant
MYT1L	chr2	2219065-2219073	TCGCCTACA	-0.1069	0.2176	-13.8759	Significant
PRKAG3	chr2	219696170-219696178	ACGCCTACA	-0.1069	0.2977	-15.3701	Significant
Intergenic	chr22	20719288-20719296	TGTAGGCGT	-0.1069	NA	-8.1509	Significant
Intergenic	chr6	92154217-92154225	TGTTGGCGT	0.1069	NA	-10.5816	Significant
PHF21A	chr11	45979546-45979554	ACGCCAACA	0.1069	-0.7153	-16.3009	Significant
SPATS2	chr12	49874350-49874358	ACGCCTACA	0.1069	-0.1338	-8.1509	Significant
ZC3H14	chr14	89067621-89067629	TGATGGCGT	0.1069	-0.7994	-10.5816	Significant
Intergenic	chr12	16274854-16274862	TGAAGGCGT	-0.0995	NA	-8.8501	Significant
THSD4	chr15	71533689-71533697	TGAAGGCGA	0.0995	0.0970	-11.6339	Significant
Intergenic	chr22	23780358-23780366	TGTTGGCGT	0.0931	NA	-7.4774	Significant
GARS	chr7	30657593-30657601	TCGCCTACA	0.0875	-0.0443	-12.1773	Significant
Intergenic	chr1	12592450-12592458	TGAAGGCGA	0.0825	NA	-8.8501	Significant
SPATA13	chr13	24623379-24623387	TCGCCTACA	-0.0740	-2.1588	-9.3305	Significant
Intergenic	chr2	8701180-8701188	ACGCCTACA	0.0000	NA	-11.9042	Significant
SPTBN1	chr2	54813966-54813974	TGTTGGCGA	0.0000	0.1455	-10.5816	Reference
TTC21B	chr2	166788276-166788284	TGAAGGCGA	0.0000	-0.1869	-50.0000	Significant
Intergenic	chr4	185235896-185235904	TGTAGGCGA	0.0000	NA	-8.6142	Significant
Intergenic	chr7	1349395-1349403	TGATGGCGA	0.0000	NA	-9.0889	Significant
Intergenic	chr7	1349428-1349436	TGATGGCGA	0.0000	NA	-9.8224	Significant
Intergenic	chr7	1349446-1349454	TGATGGCGA	0.0000	NA	-7.2587	Significant

Intergenic	chr7	9815048-9815056	TGTAGGCGA	0.0000	NA	-19.5895	Significant
DGKB	chr7	14635704-14635712	TGATGGCGA	0.0000	-0.0576	-7.9235	Significant
ANLN	chr7	36438708-36438716	TCGCCATCA	0.0000	0.2820	-8.6142	Significant
COBL	chr7	51364111-51364119	TGAAGGCGT	0.0000	0.4096	-9.3305	Significant
Intergenic	chr7	68586330-68586338	TGAAGGCGA	0.0000	NA	-10.5816	Significant
TRIM24	chr7	138233274-138233282	TGAAGGCGT	0.0000	-1.6288	-8.6142	Significant
PRKDC	chr8	48789084-48789092	TGTTGGCGT	0.0000	0.7535	-6.8297	Significant
MS4A3	chr11	59838320-59838328	TGTTGGCGA	0.0000	0.5055	-10.8404	Significant
Intergenic	chr11	71003451-71003459	TCGCCTTCA	0.0000	NA	-12.7321	Significant
Intergenic	chr13	107379908-107379916	TCGCCTTCA	0.0000	NA	-10.5816	Significant
AKAP6	chr14	32897861-32897869	TGATGGCGA	0.0000	-0.0139	-7.4774	Significant
Intergenic	chr15	60729733-60729741	TGTTGGCGA	0.0000	NA	-15.9878	Significant
Intergenic	chr16	8222966-8222974	TGATGGCGA	0.0000	NA	-9.3305	Significant
UBE2O	chr17	74445864-74445872	TGATGGCGT	0.0000	-2.0896	-8.3811	Significant
Intergenic	chr18	19890696-19890704	TGATGGCGA	0.0000	NA	-13.5857	Significant
Intergenic	chr18	20210320-20210328	ACGCCAACA	0.0000	NA	-7.2587	Significant
BCL2	chr18	60896144-60896152	TGTTGGCGA	0.0000	-0.4310	-15.9878	Reference
EBF4	chr20	2696542-2696550	ACGCCATCA	0.0000	0.4422	-6.6195	Significant
Intergenic	chr20	60535369-60535377	TGATGGCGA	0.0000	NA	-19.5895	Significant
