# Supplementary material for: Identification of Binding Targets of a Pyrrole-Imidazole Polyamide KR12 in the LS180 Colorectal Cancer Genome
Source: PLoS One. 2016 Oct 31;11(10):e0165581. doi: 10.1371/journal.pone.0165581 (PMC5087912; doi:10.1371/journal.pone.0165581)
Supplement: S4 Appendix — (PDF) [file pone.0165581.s004.pdf]

S4 Appendix. The list of predicted KR12 binding sites in the hg19 genome found within the transcripts of common transcription factors (161 TF). “Pattern” indicates motif of the binding site on the (+) strand. “Intron” indicates whether a particular site is within an intron of the transcription factor (TRUE) or otherwise (FALSE) based on RefSeq hg19 annotations.

TF	Chromosome	Predicted Site Position	Pattern	Intron
ATF3	chr1	212762236-212762244	TCGCCTTCA	TRUE
ATF3	chr1	212782315-212782323	TCGCCTTCA	TRUE
RFX5	chr1	151317822-151317830	ACGCCTACA	TRUE
RUNX3	chr1	25228698-25228706	ACGCCATCA	FALSE
RUNX3	chr1	25256080-25256088	TGAAGGCGA	FALSE
SETDB1	chr1	150936151-150936159	TGATGGCGA	FALSE
TAL1	chr1	47688305-47688313	TCGCCTACA	TRUE
TAL1	chr1	47693967-47693975	TGTTGGCGA	TRUE
ZZZ3	chr1	78046464-78046472	ACGCCATCA	TRUE
ZZZ3	chr1	78095166-78095174	TGATGGCGT	TRUE
BCL11A	chr2	60734116-60734124	TGTTGGCGT	TRUE
FOSL2	chr2	28617606-28617614	TGATGGCGT	TRUE
FOSL2	chr2	28627026-28627034	ACGCCATCA	FALSE
GTF3C2	chr2	27564339-27564347	TCGCCTACA	TRUE
MTA3	chr2	42743911-42743919	TGTAGGCGT	TRUE
MTA3	chr2	42869986-42869994	TGTTGGCGA	TRUE
MTA3	chr2	42935500-42935508	TGAAGGCGT	TRUE
STAT1	chr2	191840422-191840430	TGATGGCGA	TRUE
NR2C2	chr3	15036653-15036661	ACGCCATCA	TRUE
NR2C2	chr3	15043821-15043829	TCGCCATCA	TRUE
NR2C2	chr3	15056511-15056519	ACGCCAACA	TRUE
SMARCC1	chr3	47634014-47634022	TGTAGGCGA	TRUE
SMARCC1	chr3	47778746-47778754	TGATGGCGT	TRUE
SMARCC1	chr3	47787074-47787082	ACGCCTACA	TRUE
SMARCC1	chr3	47791309-47791317	ACGCCTACA	TRUE
REST	chr4	57786881-57786889	ACGCCATCA	TRUE
CHD1	chr5	98203654-98203662	TCGCCAACA	TRUE
EBF1	chr5	158128845-158128853	TCGCCTTCA	TRUE
EBF1	chr5	158145275-158145283	TCGCCATCA	TRUE
EBF1	chr5	158166633-158166641	TGATGGCGT	TRUE
EBF1	chr5	158281750-158281758	ACGCCATCA	TRUE
EBF1	chr5	158287842-158287850	TCGCCATCA	TRUE
EBF1	chr5	158526366-158526374	TGTTGGCGT	FALSE
MEF2C	chr5	88036232-88036240	TGTTGGCGA	TRUE
MEF2C	chr5	88156732-88156740	TCGCCTACA	TRUE
NR3C1	chr5	142712351-142712359	TGATGGCGT	TRUE
ESR1	chr6	152043648-152043656	TGTTGGCGT	TRUE
ESR1	chr6	152116024-152116032	ACGCCTTCA	TRUE
ESR1	chr6	152161134-152161142	TGTAGGCGT	TRUE
ESR1	chr6	152170113-152170121	ACGCCAACA	TRUE
ESR1	chr6	152197067-152197075	TGATGGCGT	TRUE
ESR1	chr6	152211989-152211997	TCGCCTTCA	TRUE
ESR1	chr6	152368207-152368215	ACGCCTTCA	TRUE
PRDM1	chr6	106543584-106543592	ACGCCAACA	FALSE
TBP	chr6	170865774-170865782	TGTTGGCGA	TRUE
EZH2	chr7	148523589-148523597	TCGCCTACA	FALSE
EZH2	chr7	148554705-148554713	TGATGGCGA	TRUE
EZH2	chr7	148561059-148561067	TCGCCTTCA	TRUE
EZH2	chr7	148581108-148581116	ACGCCTACA	TRUE
FOXP2	chr7	113792661-113792669	TCGCCTACA	TRUE
FOXP2	chr7	113824657-113824665	TCGCCTTCA	TRUE
FOXP2	chr7	113836122-113836130	TGATGGCGT	TRUE
FOXP2	chr7	114116482-114116490	TGTAGGCGT	TRUE
FOXP2	chr7	114290041-114290049	TCGCCTTCA	TRUE
FOXP2	chr7	114296103-114296111	ACGCCTACA	TRUE
IKZF1	chr7	50430094-50430102	TGAAGGCGT	TRUE
IKZF1	chr7	50452078-50452086	TCGCCATCA	TRUE

IKZF1	chr7 50467715-50467723	ACGCCATCA	FALSE
IKZF1	chr7 50471056-50471064	TGATGGCGT	FALSE
SP4	chr7 21488870-21488878	TCGCCAACA	TRUE
SP4	chr7 21527313-21527321	TGTAGGCGT	TRUE
ZKSCAN1	chr7 99636596-99636604	TGATGGCGT	FALSE
HSF1	chr8 145530630-145530638	ACGCCTTCA	TRUE
PAX5	chr9 36912487-36912495	ACGCCTTCA	TRUE
PAX5	chr9 37034302-37034310	TCGCCAACA	FALSE
PBX3	chr9 128719154-128719162	TCGCCTTCA	TRUE
RXRA	chr9 137246231-137246239	TGTTGGCGT	TRUE
RXRA	chr9 137269112-137269120	TGATGGCGA	TRUE
RXRA	chr9 137270180-137270188	TGAAGGCGT	TRUE
RXRA	chr9 137295746-137295754	TGTTGGCGT	TRUE
RXRA	chr9 137330540-137330548	ACGCCAACA	FALSE
CTBP2	chr10 126740648-126740656	ACGCCAACA	TRUE
CTBP2	chr10 126744917-126744925	TGAAGGCGT	TRUE
CTBP2	chr10 126825450-126825458	TGATGGCGT	TRUE
CTBP2	chr10 126843870-126843878	TGAAGGCGA	TRUE
GATA3	chr10 8116511-8116519	TGTAGGCGA	FALSE
TCF7L2	chr10 114768272-114768280	ACGCCTTCA	TRUE
TCF7L2	chr10 114898142-114898150	TCGCCTTCA	TRUE
TCF7L2	chr10 114917422-114917430	ACGCCATCA	TRUE
ZEB1	chr10 31631471-31631479	TGTAGGCGT	TRUE
ZEB1	chr10 31786613-31786621	TCGCCATCA	TRUE
ETS1	chr11 128347949-128347957	TCGCCATCA	TRUE
ETS1	chr11 128364113-128364121	ACGCCAACA	TRUE
ETS1	chr11 128392698-128392706	TGAAGGCGA	TRUE
ATF1	chr12 51201050-51201058	TGATGGCGT	TRUE
FOXM1	chr12 2977827-2977835	TGATGGCGA	FALSE
SP1	chr12 53802817-53802825	TCGCCAACA	TRUE
STAT2	chr12 56739147-56739155	TGATGGCGT	TRUE
TEAD4	chr12 3093609-3093617	TGTAGGCGA	TRUE
ELF1	chr13 41576321-41576329	ACGCCTTCA	TRUE
ELF1	chr13 41581741-41581749	ACGCCTTCA	TRUE
BRF1	chr14 105676907-105676915	TCGCCATCA	FALSE
RCOR1	chr14 103100024-103100032	TCGCCAACA	TRUE
MEF2A	chr15 100111035-100111043	TGAAGGCGT	TRUE
MEF2A	chr15 100117237-100117245	TGTTGGCGT	TRUE
MEF2A	chr15 100215382-100215390	TGAAGGCGA	TRUE
TCF12	chr15 57467741-57467749	TCGCCAACA	TRUE
CTCF	chr16 67625447-67625455	TGATGGCGT	TRUE
CTCF	chr16 67672979-67672987	ACGCCAACA	FALSE
BRCA1	chr17 41230953-41230961	ACGCCTACA	TRUE
NFATC1	chr18 77183199-77183207	ACGCCATCA	TRUE
NFATC1	chr18 77193704-77193712	TGAAGGCGT	FALSE
ARID3A	chr19 957706-957714	TGATGGCGA	TRUE
IRF3	chr19 50165258-50165266	TCGCCATCA	TRUE
JUNB	chr19 12902889-12902897	ACGCCTACA	FALSE
NFIC	chr19 3381759-3381767	TCGCCTACA	FALSE
NFIC	chr19 3423772-3423780	TCGCCATCA	TRUE
NFIC	chr19 3442279-3442287	TGAAGGCGA	TRUE
NFIC	chr19 3453405-3453413	TGATGGCGT	TRUE
SIRT6	chr19 4182296-4182304	TGTTGGCGT	TRUE
SMARCA4	chr19 11086258-11086266	TGTAGGCGT	TRUE
SMARCA4	chr19 11133447-11133455	TGATGGCGA	TRUE
SMARCA4	chr19 11170503-11170511	TGAAGGCGA	FALSE
TCF3	chr19 1624633-1624641	TCGCCTACA	TRUE
ZBTB7A	chr19 4054951-4054959	TGTAGGCGA	FALSE

ZNF274	chr19	58715749-58715757	TGTAGGCGT	TRUE
CTCFL	chr20	56073643-56073651	ACGCCTTCA	FALSE
FOXA2	chr20	22562745-22562753	TGAAGGCGT	FALSE
FOXA2	chr20	22563474-22563482	TGTTGGCGT	FALSE
HNF4A	chr20	43038938-43038946	TGATGGCGA	TRUE
TFAP2C	chr20	55210158-55210166	TGATGGCGT	TRUE
MAFF	chr22	38604581-38604589	TGATGGCGT	TRUE
SMARCB1	chr22	24145586-24145594	ACGCCTTCA	FALSE
SMARCB1	chr22	24167593-24167601	ACGCCTTCA	FALSE
HDAC6	chrX	48673817-48673825	TGAAGGCGT	FALSE
HDAC6	chrX	48678443-48678451	TCGCCATCA	TRUE
HDAC8	chrX	71620291-71620299	ACGCCATCA	TRUE
PHF8	chrX	53971262-53971270	TCGCCAACA	TRUE
PHF8	chrX	53991880-53991888	TCGCCAACA	TRUE
PHF8	chrX	54044125-54044133	TGATGGCGA	FALSE
TAF1	chrX	70621709-70621717	TGATGGCGT	TRUE
