# Supplementary figures and images for: Identification of Binding Targets of a Pyrrole-Imidazole Polyamide KR12 in the LS180 Colorectal Cancer Genome
Source: PLoS One. 2016 Oct 31;11(10):e0165581. doi: 10.1371/journal.pone.0165581 (PMC5087912; doi:10.1371/journal.pone.0165581)

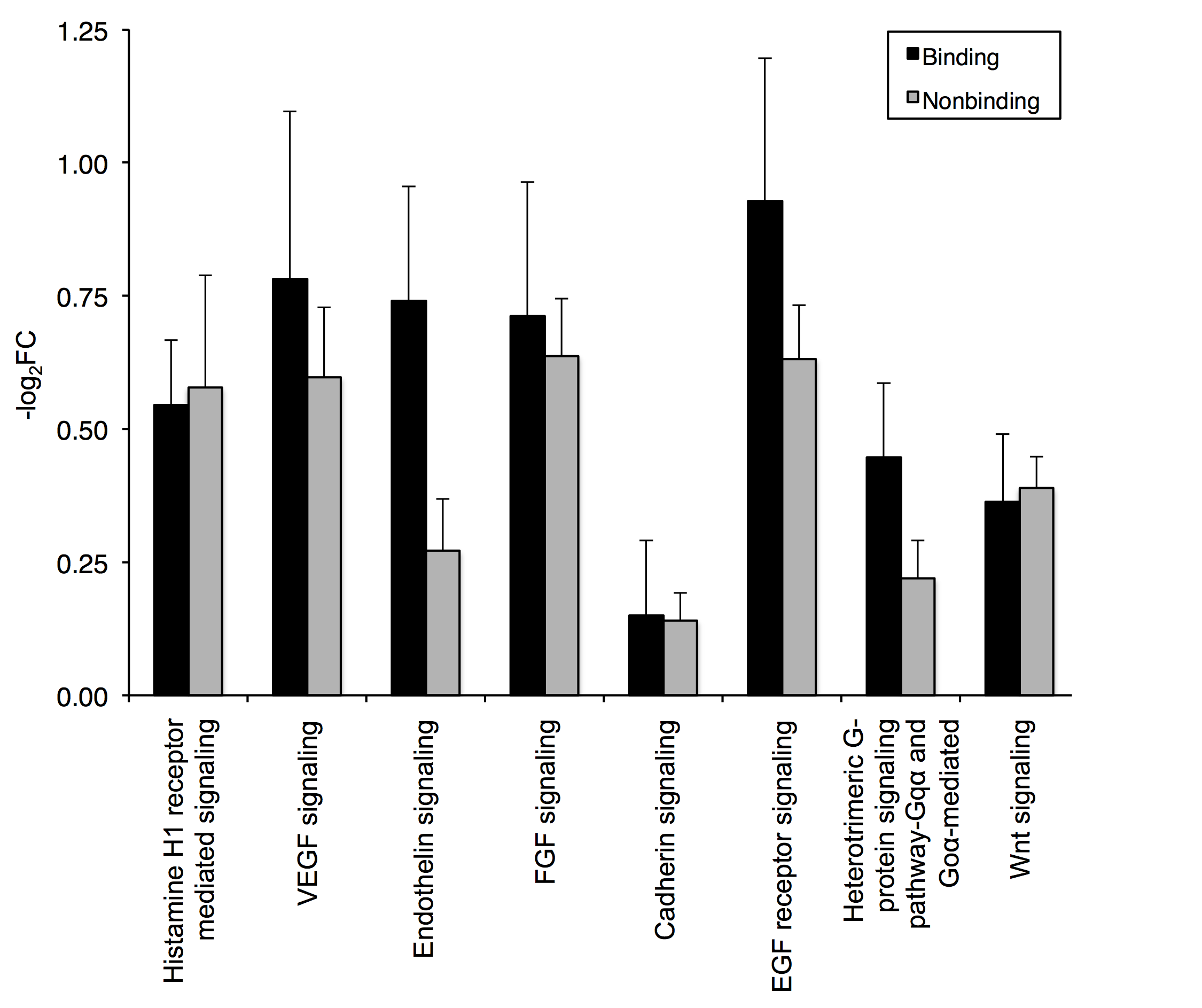

Supplement: S1 Fig — (TIFF) [file pone.0165581.s005.tiff]
