# Supplementary material for: Identification of Binding Targets of a Pyrrole-Imidazole Polyamide KR12 in the LS180 Colorectal Cancer Genome
Source: PLoS One. 2016 Oct 31;11(10):e0165581. doi: 10.1371/journal.pone.0165581 (PMC5087912; doi:10.1371/journal.pone.0165581)
Supplement: S1 Table — (DOCX) [file pone.0165581.s007.docx]

**S1 Table. List of Enriched peaks containing KR12 motifs in the LS180 dataset, using MACS 1.4.2 under default parameters for peak calling and cross-referenced to predicted KR12 binding sites in the hg19 genome.**

| **Coordinate** | **Symbol** | **Coordinate** | **Symbol** |
| --- | --- | --- | --- |
| chr1:142603549-142603557 | Intergenic | chr10:42453151-42453159 | Intergenic |
| chr1:142866428-142866436 | Intergenic | chr11:50770064-50770072 | Intergenic |
| chr2:89102401-89102409 | ANKRD36BP2 | chr12:28428063-28428071 | CCDC91 |
| chr3:75777023-75777031 | Intergenic | chr16:32140750-32140758 | Intergenic |
| chr3:90497250-90497258 | Intergenic | chr16:46454413-46454421 | Intergenic |
| chr6:57445258-57445266 | PRIM2 | chr16:71063874-71063882 | HYDIN |
| chr6:57457961-57457969 | PRIM2 | chr17:21682751-21682759 | Intergenic |
| chr7:64960955-64960963 | Intergenic | chr17:25301665-25301673 | Intergenic |
| chr7:97096675-97096683 | Intergenic | chr19:27754840-27754848 | Intergenic |
| chr9:68435874-68435882 | LOC642236 | chr21:9451625-9451633 | Intergenic |
