# Supplementary material for: Identification of Binding Targets of a Pyrrole-Imidazole Polyamide KR12 in the LS180 Colorectal Cancer Genome
Source: PLoS One. 2016 Oct 31;11(10):e0165581. doi: 10.1371/journal.pone.0165581 (PMC5087912; doi:10.1371/journal.pone.0165581)
Supplement: S2 Table — (DOCX) [file pone.0165581.s008.docx]

**S2 Table. List of KEGG human pathways in which KR12 binding significantly affects the expression and has observable specific effect compared to KR12-bound genes otherwise**.

|  |  | **Significance in Pathway Regulation** | | | **Effect of KR12 Binding in a Pathway** | | | **Specificity of Effect of KR12 Binding on a Pathway** | | |
| --- | --- | --- | --- | --- | --- | --- | --- | --- | --- | --- |
| **ID** | **Pathway** | ***p-value*** | **PT** | **NP** | ***p-value*** | **PT+** | **PT-** | ***p-value*** | **PT+** | **NP+** |
| 00410 | β-alanine metabolism | 4.47×10^-12^ | 0.169 | -0.363 | 2.16×10^-2^ | 0.040 | 0.176 | 4.29×10^-84^ | 0.040 | -0.659 |
| 00830 | Retinol metabolism | 2.05×10^-7^ | 0.106 | -0.363 | 4.55×10^-2^ | 0.607 | 0.067 | 4.96×10^-3^ | 0.607 | -0.661 |
| 04068 | FoxO signaling | 8.49×10^-3^ | -0.597 | -0.360 | 1.01×10^-2^ | -1.487 | -0.488 | 2.46×10^-2^ | -1.487 | -0.649 |
| 04120 | Ubiquitin-mediated  proteolysis | 1.30×10^-10^ | -0.968 | -0.357 | 3.42×10^-2^ | -1.441 | -0.889 | 2.44×10^-3^ | -1.441 | -0.647 |
| 04144 | Endocytosis | 1.72×10^-5^ | -0.636 | -0.358 | 1.11×10^-3^ | -1.307 | -0.546 | 3.10×10^-3^ | -1.307 | -0.643 |
| 04910 | Insulin signaling pathway | 3.51×10^-2^ | -0.519 | -0.361 | 3.75×10^-3^ | -1.249 | -0.400 | 2.93×10^-2^ | -1.249 | -0.650 |
| 05010 | Alzheimer's disease | 1.70×10^-10^ | 0.004 | -0.365 | 3.19×10^-2^ | -0.227 | 0.026 | 3.87×10^-4^ | -0.227 | -0.663 |
| 05143 | African trypano-somiasis | 1.07×10^-2^ | -0.091 | -0.362 | 1.51×10^-2^ | -0.392 | -0.032 | 1.50×10^-2^ | -0.392 | -0.659 |
| 05166 | HTLVI infection | 2.93×10^-2^ | -0.499 | -0.360 | 2.26×10^-3^ | -1.475 | -0.431 | 1.10×10^-2^ | -1.475 | -0.648 |
| 05200 | Pathways in cancer | 3.17×10^-4^ | -0.529 | -0.358 | 5.51×10^-4^ | -1.083 | -0.442 | 1.30×10^-2^ | -1.083 | -0.641 |
| 05206 | MicroRNAs in cancer | 4.76×10^-5^ | -0.696 | -0.359 | 1.49×10^-2^ | -1.403 | -0.608 | 1.78×10^-2^ | -1.403 | -0.648 |
| 05212 | Pancreatic cancer | 2.10×10^-2^ | -0.632 | -0.361 | 1.15×10^-2^ | -1.669 | -0.440 | 2.81×10^-2^ | -1.669 | -0.650 |
| 05215 | Prostate cancer | 4.66×10^-3^ | -0.670 | -0.360 | 1.07×10^-2^ | -1.619 | -0.493 | 2.25×10^-2^ | -1.619 | -0.648 |
| 05220 | Chronic myeloid leukemia | 1.28×10^-4^ | -0.866 | -0.360 | 2.37×10^-3^ | -1.975 | -0.628 | 2.38×10^-3^ | -1.975 | -0.645 |
| 05414 | Dilated cardiomyopathy | 1.23×10^-8^ | -0.006 | -0.363 | 2.30×10^-2^ | 0.214 | -0.029 | 2.89×10^-7^ | 0.214 | -0.664 |

Listed p-values are obtained from comparing mean expressions within each category by two-sample Welch *t*-test. Mean expressions of genes within a group are expressed in log_2_FC values; “PT” and “NP” indicate genes either belonging to the particular pathway or otherwise; “+” and “-“ indicate KR12-binding and non-binding, respectively.
