# Supplementary material for: Identification of Binding Targets of a Pyrrole-Imidazole Polyamide KR12 in the LS180 Colorectal Cancer Genome
Source: PLoS One. 2016 Oct 31;11(10):e0165581. doi: 10.1371/journal.pone.0165581 (PMC5087912; doi:10.1371/journal.pone.0165581)
Supplement: S3 Table — (DOCX) [file pone.0165581.s009.docx]

**S3 Table. List of KR12-bound genes that participate in one or more KEGG pathways deemed to have statistically significant changes (from Table S2) upon KR12 binding, and their relations to *KRAS* via the frequency of co-occurrence in *KRAS*-implicated pathways.**

| **Symbol** | **KEGG ID** | **Frequency** | **Symbol** | **KEGG ID** | **Frequency** |
| --- | --- | --- | --- | --- | --- |
| TGFBR2 | hsa:7048 | 8 | CREBBP | hsa:1387 | 11 |
| SMAD2 | hsa:4087 | 8 | ATM | hsa:472 | 4 |
| SMAD3 | hsa:4088 | 9 | ADCY8 | hsa:114 | 15 |
| CBL | hsa:867 | 6 | PRKCA | hsa:5578 | 30 |
| CBLB | hsa:868 | 6 | PRKCB | hsa:5579 | 28 |
| NEDD4L | hsa:23327 | 1 | KIT | hsa:3815 | 8 |
| SMURF2 | hsa:64750 | 0 | STK4 | hsa:6789 | 5 |
| PIK3R1 | hsa:5295 | 47 | E2F3 | hsa:1871 | 11 |
| PIK3R3 | hsa:8503 | 47 | BCL2 | hsa:596 | 11 |
| PIK3CA | hsa:5290 | 48 | RPTOR | hsa:57521 | 5 |
| PIK3CD | hsa:5293 | 47 | PLCG2 | hsa:5336 | 16 |
| PRKAG2 | hsa:51422 | 5 | RALA | hsa:5898 | 5 |
| FOXO1 | hsa:2308 | 8 | FGFR1 | hsa:2260 | 11 |
| MAPK10 | hsa:5602 | 19 | TCF7L1 | hsa:83439 | 7 |
| PLCB1 | hsa:23236 | 16 | MECOM | hsa:2122 | 3 |
| PLCB4 | hsa:5332 | 16 | CTBP1 | hsa:1487 | 2 |
| CDC16 | hsa:8881 | 2 |  |  |  |

“Frequency” indicates the frequency of which a certain gene appears in a *KRAS*-implicated pathway based on KEGG classifications.
