# Supplementary material for: Identification of Binding Targets of a Pyrrole-Imidazole Polyamide KR12 in the LS180 Colorectal Cancer Genome
Source: PLoS One. 2016 Oct 31;11(10):e0165581. doi: 10.1371/journal.pone.0165581 (PMC5087912; doi:10.1371/journal.pone.0165581)
Supplement: S4 Table — (DOCX) [file pone.0165581.s010.docx]

**S4 Table. List of Scrambled KR12 Motifs.**

| TCGGGGWWW | TGGCWGGWW | TGWCGWGGW | TWCWGGGWG | TWGWGGGCW |
| --- | --- | --- | --- | --- |
| TCGGWWGWG | TGGCWWGWG | TGWCWGGGW | TWCWGGWGG | TWGWGGGWC |
| TCGWGGGWW | TGGGCGWWW | TGWCWWGGG | TWCWGWGGG | TWGWGGWGC |
| TCGWGGWWG | TGGGCWGWW | TGWGCGGWW | TWCWWGGGG | TWGWGWCGG |
| TCGWGWGWG | TGGGCWWGW | TGWGCWGGW | TWGCGWGWG | TWGWWCGGG |
| TCGWWGGGW | TGGGWGWWC | TGWGGCGWW | TWGGCGWGW | TWGWWGGGC |
| TCGWWGWGG | TGGGWWCGW | TGWGGCWGW | TWGGCWWGG | TWWCGGGWG |
| TCWGGGWWG | TGGGWWCWG | TGWGGGWWC | TWGGGCWWG | TWWCGGWGG |
| TCWGGWGGW | TGGGWWGCW | TGWGGWCWG | TWGGGWCGW | TWWCGWGGG |
| TCWGGWGWG | TGGGWWGWC | TGWGGWGWC | TWGGGWCWG | TWWCWGGGG |
| TCWGWGGGW | TGGWCWGGW | TGWGWCGWG | TWGGWCGWG | TWWGCGGGW |
| TCWGWGGWG | TGGWCWWGG | TGWGWGCGW | TWGGWGCGW | TWWGCGGWG |
| TCWGWGWGG | TGGWGCWGW | TGWGWGGCW | TWGGWGCWG | TWWGCGWGG |
| TCWWGGGGW | TGGWGGCWW | TGWGWGGWC | TWGGWGGCW | TWWGGCGGW |
| TGCGGWWGW | TGGWGWCGW | TGWGWWCGG | TWGGWGGWC | TWWGGGGCW |
| TGCGWGGWW | TGGWGWCWG | TGWWCWGGG | TWGGWWGGC | TWWGGWCGG |
| TGCGWWGWG | TGGWGWGCW | TGWWGWGGC | TWGWCGWGG | TWWGWCGGG |
| TGCWGWGWG | TGGWWGGWC | TGWWWGGCG | TWGWCWGGG | TWWWCGGGG |
| TGCWWGGWG | TGGWWGWGC | TWCGGGGWW | TWGWGCWGG | TWWWGCGGG |
| TGGCGWGWW | TGGWWWGGC | TWCGGWGGW | TWGWGGCWG | TWWWGGGCG |

All motifs are listed beginning at the 5’ (+) terminus, with the first base being a fixed T to simulate functionalization with CBI to be consistent with KR12.
